# Electronic cigarettes and health outcomes: umbrella and systematic review of the global evidence

**DOI:** 10.5694/mja2.51890

**Published:** 2023-03-20

**Authors:** Emily Banks, Amelia Yazidjoglou, Sinan Brown, Mai Nguyen, Melonie Martin, Katie Beckwith, Amanda Daluwatta, Sai Campbell, Grace Joshy

**Affiliations:** ^1^ National Centre for Epidemiology and Population Health Australian National University Canberra ACT

**Keywords:** Electronic cigarettes, Systematic review, Public health

## Abstract

**Objective:**

To review and synthesise the global evidence regarding the health effects of electronic cigarettes (e‐cigarettes, vapes).

**Study design:**

Umbrella review (based on major independent reviews, including the 2018 United States National Academies of Sciences, Engineering, and Medicine [NASEM] report) and top‐up systematic review of published, peer‐reviewed studies in humans examining the relationship of e‐cigarette use to health outcomes published since the NASEM report.

**Data sources:**

Umbrella review: eight major independent reviews published 2017–2021. Systematic review: PubMed, MEDLINE, Scopus, Web of Science, the Cochrane Library, and PsycINFO (articles published July 2017 – July 2020 and not included in NASEM review).

**Data synthesis:**

Four hundred eligible publications were included in our synthesis: 112 from the NASEM review, 189 from our top‐up review search, and 99 further publications cited by other reviews. There is conclusive evidence linking e‐cigarette use with poisoning, immediate inhalation toxicity (including seizures), and e‐cigarette or vaping product use‐associated lung injury (EVALI; largely but not exclusively for e‐liquids containing tetrahydrocannabinol and vitamin E acetate), as well as for malfunctioning devices causing injuries and burns. Environmental effects include waste, fires, and generation of indoor airborne particulate matter (substantial to conclusive evidence). There is substantial evidence that nicotine e‐cigarettes can cause dependence or addiction in non‐smokers, and strong evidence that young non‐smokers who use e‐cigarettes are more likely than non‐users to initiate smoking and to become regular smokers. There is limited evidence that freebase nicotine e‐cigarettes used with clinical support are efficacious aids for smoking cessation. Evidence regarding effects on other clinical outcomes, including cardiovascular disease, cancer, development, and mental and reproductive health, is insufficient or unavailable.

**Conclusion:**

E‐cigarettes can be harmful to health, particularly for non‐smokers and children, adolescents, and young adults. Their effects on many important health outcomes are uncertain. E‐cigarettes may be beneficial for smokers who use them to completely and promptly quit smoking, but they are not currently approved smoking cessation aids. Better quality evidence is needed regarding the health impact of e‐cigarette use, their safety and efficacy for smoking cessation, and effective regulation.

**Registration:**

Systematic review: PROSPERO, CRD42020200673 (prospective).

Electronic cigarettes (e‐cigarettes, vapes) are devices that aerosolise an “e‐liquid” for inhalation.^1^, ^2^, ^3^ Devices range from older low power “cigalikes” to refillable pen and larger tank devices, to more recent small high concentration nicotine salt pods and disposable products.^2^, ^3^, ^4^, ^5^ E‐cigarettes are used by millions of people around the world, particularly by younger people.^6^, ^7^ In the 2019 Australian National Drug Strategy Household Survey, 26% of 18–24‐year‐old respondents and 10% of those aged 40–49‐years reported having used e‐cigarettes at least once.^8^

The contemporary evidence on e‐cigarettes, including information about their direct effects on health and indirect effects (eg, impact on smoking behaviour), must be integrated to inform evidence‐based policy and practice. Several major reviews of the health effects of e‐cigarettes have been published,^2^, ^3^, ^9^, ^10^, ^11^, ^12^ but no contemporary comprehensive systematic reviews or major reports with systematic quality assessment were identified prior to our review for the Australian Department of Health.^13^ We provide a contemporary overview of the evidence regarding the health effects of nicotine and non‐nicotine e‐cigarettes.

## Methods

Our review integrates an umbrella review of evidence from major independent reviews with a top‐up systematic review, following PRISMA guidelines,^14^ using the same search terms and eligibility criteria. It is based on work commissioned by the Australian Department of Health,^13^, ^15^ which also provided evidence for the 2021 Royal Australian College of General Practitioners (RACGP) update to smoking cessation guidelines^16^ and the 2022 statement of the National Health and Medical Research Council (NHMRC) Chief Executive Officer.^17^

### Umbrella review

As our starting point, we used the most recent large scale review, the 2018 United States National Academies of Sciences, Engineering, and Medicine (NASEM) review,^3^ and we searched for other major reviews in the grey literature to 31 December 2021. Studies included in the NASEM and other major reviews but not already identified by our top‐up review (see below) were evaluated using the same eligibility criteria as the top‐up review, summarised, and integrated into our narrative synthesis. We identified major reviews published after our 2021 search (to the end of November 2022) using rapid search methods (Supporting Information, part 1); these reviews are not included in our results synthesis but are considered in our discussion.

### Top‐up review

We searched PubMed, MEDLINE, Scopus, Web of Science, the Cochrane Library, and PsycINFO in July 2020 for relevant publications during July 2017 – July 2020 not included in the NASEM review (details: Supporting Information, part 1; protocol published in PROSPERO: CRD42020200673, 31 August 2020). We included evidence regarding e‐cigarettes and smoking initiation and cessation reported by the current authors in previous systematic reviews in our analysis.^15^, ^18^, ^19^

#### Inclusion and exclusion criteria

We included peer‐reviewed original research articles on the health effects of e‐cigarettes. When possible, evidence related to e‐cigarettes delivering tetrahydrocannabinol (THC) was excluded as being outside the scope of the review, as were animal, *in vitro*, and biomarker studies (Supporting Information, part 1).

#### Eligibility screening

Article details were imported into EndNote, exported to Covidence (https://www.covidence.org), and duplicates removed. Two authors (two of AY, SB, KB, MM, MN, AD, SC) independently screened all titles, abstracts, and articles according to the eligibility criteria; disagreements were resolved by consensus or by a third author.

#### Data extraction

One of the authors independently extracted data using a pre‐specified piloted data extraction template; data were checked by a second author. Outcomes were classified by clinical disease endpoints (eg, stroke, myocardial infarction, cancer), subclinical outcomes (eg, coronary artery calcification, lung function), and other health outcomes. Missing data, competing interests, and funding were noted.

#### Risk of bias assessment

The methodological quality of studies included in the top‐up review was independently assessed by two authors using Joanna Briggs Institute critical appraisal tools (https://jbi.global/critical‐appraisal‐tools). Disagreements were resolved by consensus discussions or by consultation with a third author. Three otherwise eligible studies were excluded because of low quality scores.^20^, ^21^, ^22^

### Integrated data analysis

#### GRADE assessment

The certainty of the integrated body of evidence for each health outcome derived from the umbrella and top‐up reviews was assessed using the GRADE approach^23^, ^24^ (Supporting Information, part 2).

#### Data synthesis

We prioritised study designs most useful for assessing causal effects with respect to the health outcome (as applicable), in the order: randomised controlled trials; prospective cohort studies; case–control studies; and non‐randomised intervention studies. Published meta‐analyses were included when available. For the smoking cessation analyses, we included only randomised controlled trials with biochemically verified cessation outcomes and at least four months of follow‐up. When epidemiological studies were unavailable or not relevant, other potentially informative evidence was examined, including cross‐sectional surveys, case reports and case series (particularly for exposure‐dependent outcomes, such as burns and injuries), and findings from surveillance systems.

We separately summarised the study characteristics and main findings for the umbrella and top‐up reviews, and then integrated them in a narrative synthesis, drawing conclusions on the certainty of the evidence (NASEM framework), by exposure groups and comparators. We conducted meta‐analyses when possible (Supporting Information, part 2).

## Results

We identified 6558 potentially eligible publications for the top‐up review. After screening, 227 were eligible for our analysis. A further ten items were identified by forward and backward citation searching, and one was identified in the grey literature. Eighty‐six publications were excluded because the study designs precluded drawing causal inferences about links between e‐cigarette use and health outcomes. A further 37 relevant publications were identified in two earlier reviews of smoking uptake and cessation by the authors.^15^, ^18^ In total, 189 studies met our selection criteria for the top‐up review (Box 1).

Box 1 Selection of publications for inclusion in the top‐up systematic review*

**Figure:**
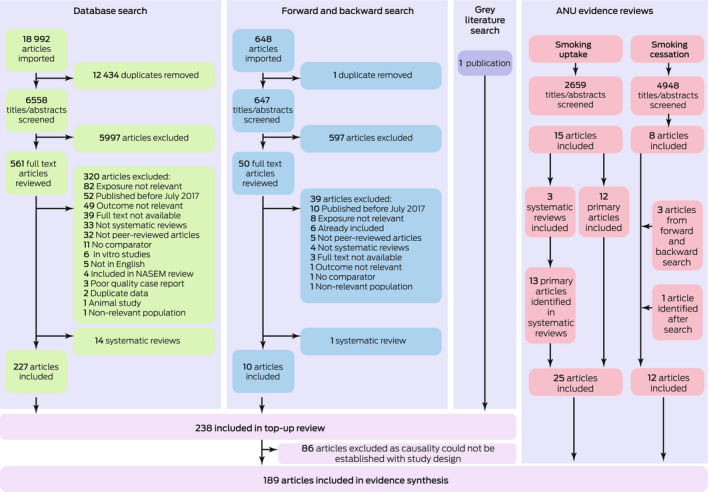
ANU = Australian National University. * Figure modified from our larger e‐cigarette health outcomes review report, with permission.^13^

Eight relevant major independent reviews were identified: those prepared for Public Health England (2018, 2020, 2021),^11^, ^25^, ^26^ the Commonwealth Scientific and Industrial Research Organisation (Australia, 2018),^9^ NASEM (United States, 2018),^3^ the Irish Health Research Board (2020),^10^ the European Union Scientific Committee on Health, Environmental, and Emerging Risks (2021),^2^ and the United States Preventive Services Task Force (2021).^12^ A total of 112 relevant studies were identified in the NASEM review, as well as 99 additional studies cited by other reviews and not captured in the top‐up review.

A total of 400 publications were therefore included in the final evidence synthesis (Supporting Information, parts 3 and 4; Box 2).^13^ Information on nicotine content was generally not provided; however, as almost all e‐cigarette products sold probably contain nicotine,^32^ we assumed that reported health effects were related to nicotine‐containing e‐cigarettes, unless otherwise noted.

Box 2 Overview of the 400 publications included in our systematic review evidence synthesis, by health outcome category and study design*

**Table:** 

Health outcome	Meta‐analysis	Randomised controlled trial	Cohort study	Non‐randomised intervention study	Case–control study	Surveillance report	Cross‐sectional survey	Case series	Case report
Dependence and abuse liability		13^†^	1	17			21		
Cardiovascular disease	1	11	1	7					1
Cancer			1						
Respiratory disease		10^‡^	6^‡^	8		18		7	9
Oral health			2	2					
Developmental/ reproductive			2				1		
Burns and injuries						7		26	34
Poisoning						28		4	27
Mental health			3						
Environmental hazards^§^				17		3		5	
Neurological						3		2	8
Sleep									
Less serious adverse events	1	24	17	4		1			
Ocular health				1					
Wound healing									
Olfactory							1		
Endocrine							2		
Allergy diseases								1	3
Haematological									
Smoking uptake	3	2	23						
Smoking cessation		11							

* Figure modified from our larger e‐cigarette health outcomes review report, with permission.^13^ In two cases, the listed study design is different to the design stated by the study authors. For example, a trial described by study authors as a “randomised crossover trial” without reference to randomisation did not meet the requirements to be classified a randomised trial.

† One article described two separate randomised controlled trials,^27^ both of which are included in the count.

‡ Two article pairs that reported the same data (a cohort study^28^, ^29^ and a randomised controlled trial^30^, ^31^) were each combined for evidence synthesis.

§ Characterisation of studies with environmental outcomes differ from those for other outcomes; in this table, “non‐randomised intervention studies” refer to controlled experimental studies, “case series” to natural experiments.

### Health outcomes

Evidence regarding the health effects of e‐cigarettes is very limited. The risks of several adverse health outcomes are higher in e‐cigarette users. There is conclusive evidence that nicotine e‐cigarettes can cause poisoning and immediate inhalation toxicity (including seizures), particularly in children and adolescents, and that malfunctioning devices can cause injuries and burns; there is substantial evidence that nicotine e‐cigarettes can cause dependence or addiction in non‐smokers. There is conclusive evidence that e‐cigarettes cause e‐cigarette or vaping product use‐associated lung injury (EVALI), largely for e‐liquids containing THC (and the additive vitamin E acetate, identified in many, but not all, THC‐containing products); however, 14% of EVALI cases in the largest relevant study were linked to nicotine‐containing e‐liquids without these constituents.^33^ There is moderate evidence that nicotine e‐cigarettes can cause less serious adverse events, such as headache, cough, throat irritation, dizziness, and nausea. Identified environmental effects include waste, fires, and generation of indoor airborne particulate matter (substantial to conclusive evidence) (Box 3).

Box 3 Summary of evidence synthesis on relationships between use of e‐cigarettes and health outcomes*

**Table:** 

Health outcome type (number of studies)	Summary of conclusions from evidence review
Dependence and abuse liability (52)	Non‐smokers: substantial evidence that nicotine e‐cigarette use leads to dependence on e‐cigarettes. Smokers: limited evidence that nicotine e‐cigarette use leads to dependence on e‐cigarettes; limited evidence that the abuse liability of nicotine e‐cigarettes is lower than for cigarettes or that abuse liability of nicotine e‐cigarettes is higher than for nicotine replacement therapy products. Smokers: insufficient evidence that abuse liability is influenced by flavour and nicotine content.
Cardiovascular disease (21)	Risks of clinical cardiovascular disease outcomes (eg, myocardial infarction, stroke or cardiovascular mortality): no available evidence. Risks of subclinical atherosclerosis‐related outcomes (eg, carotid intima media thickness, coronary artery calcification): no available evidence. Other cardiovascular outcomes in non‐smokers (increased blood pressure, heart rate, autonomic control and arterial stiffness; reduced endothelial function, hand microcirculation and cardiac function/geometry; cardiac device interference): insufficient evidence. Other cardiovascular outcomes in smokers: moderate evidence that nicotine e‐cigarettes acutely increase heart rate, systolic blood pressure, diastolic blood pressure and arterial stiffness; limited evidence that they increase endothelial dysfunction, and that long term nicotine e‐cigarette use after switching from cigarette smoking reduces blood pressure.
Cancer (1)	Invasive cancer risk: no available evidence. Risks of pre‐cancer/subclinical cancer outcomes: no available evidence.
Respiratory disease (58)	Respiratory disease (EVALI): conclusive evidence of a causal link in smokers and non‐smokers; largely for use of THC‐containing products, but in the largest relevant study 14% of cases involved people who reported exclusive use of nicotine‐only products and 2% reported use of non‐nicotine products (no THC or nicotine).^33^ Other clinical respiratory outcomes (asthma, bronchitis, chronic obstructive pulmonary disease): insufficient (smokers)^†^ or no available evidence (non‐smokers). Reduced respiratory exacerbations, disease progression in adult smokers (healthy, asthmatic, chronic obstructive pulmonary disease) who switch to exclusive or dual use of e‐cigarettes: insufficient evidence.^†^ Acute effects (up to two hours after use) on spirometry parameters after use of nicotine or non‐nicotine e‐cigarettes: limited (non‐smokers) or insufficient evidence (smokers). Increased respiratory resistance and impedance in healthy and asthmatic smokers (up to 30 minutes after use) with use of nicotine e‐cigarettes: limited evidence. Exhaled breath outcomes for smokers and non‐smokers (healthy, asthmatic) with nicotine e‐cigarette use: insufficient evidence. Other respiratory measures (sinonasal symptoms, airway hyperresponsiveness): insufficient (smokers) for nicotine e‐cigarette use or no available evidence (non‐smokers).
Oral health (4)	Clinical or intermediate/subclinical oral health outcomes for exclusive e‐cigarette users (independent of smoking): no available evidence. Reduced plaque, gingival and papillary bleeding in smokers switching to nicotine e‐cigarette use: insufficient evidence. Increased gum disease, bone loss around teeth, any periodontal disease in exclusive nicotine e‐cigarette users, dual users, or non‐smokers (never and former smokers): insufficient evidence.
Developmental and reproductive (3)	Development of children or adolescents: no available evidence. Pregnancy and foetal outcomes (eg, low birthweight, pre‐term birth, Apgar score, small‐for‐gestational‐age birth) for exclusive generally nicotine e‐cigarette users and dual users: insufficient evidence. Other reproductive outcomes: no available evidence.
Burns and injuries (67)	Potentially fatal or severe burns and injuries: conclusive evidence of causal link.
Poisoning (59)	Intentional or accidental exposure to nicotine e‐liquids causing potentially severe poisoning, which can be fatal: conclusive evidence. A considerable number of accidental poisonings are in children under six years of age.^34^ Potential for nicotine toxicity: conclusive evidence.
Mental health (3)	Clinical mental health outcomes: no available evidence. Depressive symptoms: insufficient evidence;^†^ alternative subclinical mental health measures: no available evidence.
Environmental hazards with health implications (25)	Increased airborne particulate matter in indoor environments: conclusive evidence. Increased concentrations of airborne nicotine and of nicotine and cotinine on indoor surfaces: limited evidence. Increased air levels of carbon dioxide, carbon monoxide, propylene glycol, volatile organic compounds, carbonyls: insufficient evidence. As cause of fires and environmental waste: substantial evidence; of a level hazardous to human health: insufficient evidence.
Neurological (13)	Seizures with use of nicotine e‐cigarettes: conclusive evidence. Injuries from e‐cigarette explosions causing nerve damage: limited evidence. Risks of other clinical neurological outcomes: no available evidence.
Sleep (0)	No available evidence.
Less serious adverse events (47)	Throat irritation, cough, dizziness, headache, nausea with use of generally nicotine e‐cigarettes: moderate evidence.
Ocular health (1)	Clinical outcomes: no available evidence. Corneal epithelial thickness, pre‐corneal tear film stability with use of nicotine e‐cigarettes: insufficient evidence. Other ocular outcomes: no available evidence.
Wound healing (0)	Clinical or subclinical wound healing outcomes: no available evidence.
Olfactory (1)	Clinical olfactory outcomes: no available evidence. Subclinical olfactory measures with use of nicotine e‐cigarettes: insufficient evidence.
Endocrine (2)	Clinical endocrine outcomes: no available evidence. Subclinical endocrine outcomes (prediabetes, insulin resistance): insufficient evidence.^†^
Allergic diseases (4)	Contact dermatitis: limited evidence.^†^ Other clinical allergy outcomes: no available evidence.
Haematological (0)	No available evidence.

EVALI = e‐cigarette or vaping product use‐associated lung injury; THC = tetrahydrocannabinol.

* Table modified from our larger e‐cigarette health outcomes review report, with permission.^13^

† Nicotine content not reported in original studies. Many studies did not provide data on whether products were nicotine or non‐nicotine e‐cigarettes; in such cases, use was assumed to be largely of nicotine e‐cigarettes. When the information was provided, the product types are specified.

There is insufficient evidence regarding changes in respiratory symptoms, exacerbations of respiratory disease, lung function, and other respiratory measures in smokers who switch to exclusively using nicotine e‐cigarettes. There is limited or insufficient evidence that nicotine e‐cigarette use by non‐smokers (mostly people who had never smoked) leads to acute reductions in lung function or other respiratory measures. There is moderate evidence that nicotine e‐cigarettes immediately increase heart rate, systolic and diastolic blood pressure, and arterial stiffness acutely after use, by smokers.

Evidence is insufficient or unavailable regarding the effects of nicotine and non‐nicotine e‐cigarette use on cardiovascular disease, cancer, respiratory conditions other than lung injury, mental health, development in children and adolescents, reproduction, sleep, wound healing, neurological conditions (other than seizures), and endocrine, olfactory, ocular, allergic, and haematological conditions (Box 2, Box 3).

We rated the certainty of evidence for clinical and subclinical outcomes as low or very low, largely because of limitations in sample size and study design, and an overall paucity of evidence that caused serious concerns about inconsistency, imprecision, and indirectness (Supporting Information, part 5).

### Smoking behaviour

Meta‐analyses of 25 longitudinal studies found strong evidence that young never‐smokers and non‐smokers who use e‐cigarettes are about three times as likely as non‐users to start smoking tobacco and to become regular smokers.^18^ Significant elevations in risk were identified by all studies included in the meta‐analyses. Potential residual confounding cannot be excluded, but the findings were similar after adjusting for a variety of factors, including risk‐taking. The relationship between e‐cigarette use and smoking was deemed likely to be causal according to the Bradford–Hill criteria.^35^

There is limited evidence from eleven randomised controlled trials that freebase nicotine e‐cigarettes used with clinical support are as efficacious as smoking cessation aids as approved nicotine replacement therapy or usual care/no intervention; evidence comparing their efficacy with that of non‐nicotine e‐cigarettes is insufficient.^15^, ^19^ Evidence that non‐nicotine e‐cigarettes are efficacious compared with counselling or approved nicotine replacement therapy for smoking cessation is insufficient. There is limited evidence that the likelihood of relapse is about twice as high for ex‐smokers who use e‐cigarettes than for those who do not^18^ (Box 4).

Box 4 Summary of evidence synthesis on relationships between e‐cigarette use and smoking behaviour*

**Table:** 

Smoking behaviour (number of studies)	Summary of conclusions from evidence review
Smoking uptake (28)	Young never‐smokers who use e‐cigarettes are more likely than people who do not to initiate cigarette smoking: strong evidence Young non‐smokers who use e‐cigarettes are more likely than non‐smokers who do not to become current smokers: strong evidence. Former smokers who use e‐cigarettes are more likely to resume smoking than those who have not used e‐cigarettes: limited evidence.
Smoking and nicotine cessation (11)	Use of freebase nicotine e‐cigarettes with clinical support is more efficacious for assisting smoking cessation than nicotine replacement therapy, or than no intervention or usual care: limited evidence. Trials in which efficacy was found used products with freebase nicotine concentrations of no more than 20 mg/mL. Efficacy of nicotine e‐cigarettes for smoking cessation, compared with non‐nicotine e‐cigarettes: insufficient evidence. Efficacy of non‐nicotine e‐cigarettes for smoking cessation, compared with counselling or nicotine replacement therapy: insufficient evidence. Efficacy of non‐clinical use of freebase nicotine e‐cigarettes for assisting smoking cessation: insufficient evidence. Efficacy of nicotine salt products for assisting smoking cessation: no available evidence. Use of nicotine e‐cigarettes for assisting smoking cessation results in greater exposure to nicotine (smoking, exclusive e‐cigarette use or dual smoking/e‐cigarette use) than nicotine replacement therapy: limited evidence.

* Table modified from our larger e‐cigarette health outcomes review report, with permission. ^13^

Many studies did not provide data on whether products were nicotine or non‐nicotine e‐cigarettes; in these cases, use was assumed to be largely of nicotine e‐cigarettes. When the information was provided, the product types are specified.

## Discussion

The published evidence indicates that use of nicotine e‐cigarettes increases the risks of adverse health outcomes, including addiction, poisoning, toxicity from inhalation (including seizures), and lung injury (largely but not exclusively attributable to THC/vitamin E acetate‐containing products). Devices can cause trauma and burns, largely because of exploding lithium batteries. There is evidence for adverse effects on cardiovascular health measures (including blood pressure and heart rate) and lung function. Non‐smoking young people who use e‐cigarettes are about three times as likely as non‐users to start tobacco smoking and to become regular smokers.^18^ Bearing in mind caveats regarding inferences based on observational data and the possibility of residual confounding, this relationship was deemed probably causal. Non‐smokers and young people are most vulnerable to e‐cigarette adverse events, as they are disproportionately affected by risks such as addiction, poisoning, toxicity from inhalation, and increased smoking uptake, with little or no potential benefit through increased smoking cessation.^13^ The impact of e‐cigarettes on the environment includes indoor air pollution, waste, and fires.

Evidence for the efficacy of freebase nicotine e‐cigarettes as smoking cessation aids was largely from studies incorporating clinical support and was limited. This conclusion is consistent with those of other major independent reviews (2018–2021) that evidence for the efficacy of e‐cigarettes as smoking cessation aids is limited,^3^ unavailable,^9^ similar to that of other smoking cessation therapies,^36^ inadequate,^37^ of increasing strength that they may be helpful in the short term,^26^ weak,^2^ or insufficient.^38^ The 2022 Cochrane review of e‐cigarettes as smoking cessation aids^39^ included major reviews published since our umbrella review search. We rated it less independent than other reviews because four of its authors were also investigators in the included trials. The review found high certainty evidence that nicotine e‐cigarettes were more efficacious as smoking cessation aids than standard nicotine replacement therapy (six studies), moderate certainty evidence that they were more efficacious than non‐nicotine e‐cigarettes, and very low certainty evidence that they were more efficacious than usual care or behavioural support.^39^ The review inclusion criteria were broader than ours, including self‐reported cessation outcomes and trials not published as peer‐reviewed articles, and the authors generally rated risk of bias in individual trials lower than we did.

A key finding of our synthesis was the general paucity of evidence for the effects of nicotine and non‐nicotine e‐cigarettes on many major clinical outcomes, including cancer, cardiovascular, metabolic, mental health, developmental, reproductive, and neurological outcomes (other than seizures). The evidence chiefly concerns nicotine e‐cigarettes and outcomes that can be detected within months or years of commencing use (including effects on smoking behaviour) and acute outcomes for which links with e‐cigarette use may be apparent at the individual or group level, such as addiction, poisoning, burns, nicotine toxicity, and EVALI. An English review we identified after our umbrella review search noted the lack of direct evidence with regard to major clinical outcomes and that most serious adverse events were infrequent; the authors focused on comparing the effects of e‐cigarette use with those of smoking and on biomarkers, and generally found more favourable results for e‐cigarettes than for tobacco cigarettes.^40^

Overall, our findings add to and concur broadly with those of other major reviews, including our conclusion that the direct impact of e‐cigarettes on clinical disease outcomes are largely unknown.

E‐cigarette users inhale a complex mixture of chemicals, including nicotine, solvent carriers, flavourings, tobacco‐specific nitrosamines, volatile organic compounds, phenolic compounds, tobacco alkaloids, aldehydes, free radicals, reactive oxygen species, furans, and metals; many are associated with adverse health effects.^13^, ^41^ As well as being highly addictive, human and animal studies indicate that nicotine has adverse effects on cardiovascular markers, lung function, and brain development and function in adolescents.^3^, ^42^, ^43^, ^44^, ^45^, ^46^, ^47^, ^48^, ^49^ Certain harms identified here, including those related to devices, probably also apply to non‐nicotine e‐cigarettes, as does the uncertainty regarding other effects.

We identified a range of health harms, and no benefits, for non‐smokers who use e‐cigarettes. The World Health Organization recognises addiction *per se* as a harmful outcome.^50^ Nicotine addiction and aggressive marketing underpin the widespread and increasing use of e‐cigarettes by young people.^51^ The direct health risks, the association of e‐cigarette use with taking up tobacco smoking, and the uncertainty about their effects on major health outcomes mean that e‐cigarette use by non‐smokers, especially children and adolescents, is an important public health problem. The health impact of e‐cigarettes in ex‐smokers is reduced if they use other means to quit or if any e‐cigarette use is short‐term; there is limited evidence that relapse to smoking is more likely for e‐cigarette users. For non‐nicotine e‐cigarettes, we found no benefits in terms of smoking cessation, harms related to devices, and uncertainty regarding health effects, indicating overall harm.

Appropriate therapeutic use of a product requires evidence of acceptable safety and efficacy, a favourable risk–benefit balance, and quality.^52^, ^53^, ^54^ Most smokers who successfully quit do so without cessation aids,^55^, ^56^ and many approved smoking cessation aids of established safety, quality, and efficacy are available.^16^ In many countries, dual tobacco smoking and e‐cigarette use is the most frequent pattern of e‐cigarette use.^8^, ^57^, ^58^ The direct health impact of dual use is unknown, and e‐cigarettes may facilitate continued smoking, increasing risks.^59^ Smokers are subject to the adverse health effects of e‐cigarettes, while other consequences (poisoning, environmental impact, use by non‐smokers) can affect family and community members. E‐cigarettes may be beneficial for smokers who use them to completely and promptly quit smoking, but the limited evidence in this regard, their risks, uncertainty about their effects on major clinical outcomes, and continued smoking by most users render their overall safety and efficacy unclear. They are not registered therapeutic goods in Australia or overseas.^12^ The United States Preventive Services Task Force noted that “the current evidence is insufficient to assess the balance of benefits and harms of electronic cigarettes (e‐cigarettes) for tobacco cessation in adults, including pregnant persons”^38^ and they are not currently recommended for this purpose in the United States.^60^ The RACGP, in light of the evidence limitations, recommends that e‐cigarettes be prescribed only for fully informed people who have unsuccessfully tried other smoking cessation methods.^16^

Risks related to e‐cigarettes are likely to be increased by certain product attributes, as well as by factors that lead to greater use by people not using them for smoking cessation, including higher e‐liquid nicotine concentration; greater e‐liquid volume; “at home” dilution and other e‐liquid preparation; adulteration of e‐liquids; poor labelling and non‐child‐resistant packaging; high concentration nicotine salt products; flavourings and other characteristics or branding that appeal to children, adolescents and non‐smokers; availability, advertising and promotion; low cost; inadequate enforcement of legal restrictions on the supply and advertising of e‐cigarettes; tobacco and nicotine industry influence; and misinformation about their effects on health.^2^, ^13^, ^61^, ^62^

### Limitations

Methodological challenges contribute to the paucity of reliable evidence. E‐cigarettes are relatively new, diverse, and changing rapidly.^2^ Consequently, long term evidence is unavailable and products examined in specific studies do not necessarily reflect current options, as indicated, for example, by the limited information on more recent nicotine salt products. Further evaluation of the effects of e‐cigarettes on health must consider product diversity and changes over time. Identified risks should be assumed to apply to e‐cigarettes in general, unless robust evidence indicates otherwise.

Most publications did not include information on e‐cigarette type or e‐liquid constituents, and e‐cigarettes labelled as nicotine‐free often include nicotine, undermining the reliability of some data.^2^ While the use of non‐nicotine e‐cigarettes is likely to be uncommon, their inclusion in some studies would reduce the apparent magnitude of nicotine‐related effects and our findings would consequently be conservative.

Reliable evidence on clinical health outcomes requires appropriate short and long term, large scale, high quality studies and statistical analyses. Moreover, the effects of e‐cigarettes and tobacco smoking must be reliably distinguished. Tobacco smoking causes a wide range of health harms that vary according to the intensity, duration, and recentness of smoking.^63^, ^64^ Older e‐cigarette users are generally established and continuing smokers (dual users), smokers aiming to quit, and ex‐smokers, but younger users are often not established smokers, including people who have never smoked, have tried smoking in the past, or smoke infrequently. Confounding between the effects of e‐cigarette use and smoking is therefore a major problem, and statistical adjustment is unlikely to fully overcome this difficulty, particularly for the health conditions most affected by smoking. Consequently, people who have never smoked comprise the most reliable group in which to quantify the effects of e‐cigarettes, and people neither smoking nor using e‐cigarettes should be the main comparison group.^3^ Statements that e‐cigarettes are “safer than smoking” and terms such as “tobacco harm reduction” may be useful when discussing the minority of smokers who use e‐cigarettes to quit completely, but are not applicable to non‐smokers. Comparisons with the effects of smoking do not permit the reliable ascertainment of the direct health impact of e‐cigarettes.

For completeness, we also discussed evidence related to physiological and other outcome types and study designs. Findings from these study types should be interpreted cautiously as they do not provide reliable evidence about the causal relationships between e‐cigarette use and clinical disease outcomes.

The certainty of evidence was rated very low for most clinical and subclinical outcomes. As the tobacco and related industries can influence research conduct and distort study findings,^65^ it is important to ensure that research is free of competing interests. Risk of bias assessment was based on methodological quality and potential competing interests, such as financial support from the tobacco or pharmaceutical industries. Although risks of bias were noted across health outcomes, significant concerns in other domains meant that they were unlikely to have large effects on our interpretation of their findings or the overall certainty of evidence. Application of the precautionary principle, whereby exposure to an agent with uncertain effects should be avoided,^66^ is appropriate when serious adverse impacts are scientifically plausible, particularly those affecting future generations.

## Conclusions

There is strong or conclusive evidence that nicotine e‐cigarettes can be harmful to health, particularly for non‐smokers and children and adolescents. Evidence for their effects, and those of non‐nicotine e‐cigarettes, on most important health outcomes is uncertain. Smokers who quit by switching completely and promptly to e‐cigarettes may benefit from their use, but they are not currently approved as medical smoking cessation aids. The evidence we have synthesised supports policy and regulatory efforts to reduce e‐cigarette use in the general population, particularly by non‐smokers and children, adolescents, and young adults, and for purposes other than smoking cessation. Better quality evidence is required regarding e‐cigarette health effects, their safety and efficacy as smoking cessation aids, and effective regulatory options.

## Open access

Open access publishing facilitated by Australian National University, as part of the Wiley ‐ Australian National University agreement via the Council of Australian University Librarians.

## Competing interests

No relevant disclosures.

## Supporting information


**Data S1** Supporting Information
