# Supplementary material for: Electronic cigarettes and health outcomes: umbrella and systematic review of the global evidence
Source: Med J Aust. 2023 Mar 20;218(6):267–75. doi: 10.5694/mja2.51890 (PMC10952413; doi:10.5694/mja2.51890)
Supplement: Supplementary file 1 — Data S1 Supporting Information [file MJA2-218-267-s001.pdf]

The Medical Journal of Australia

Supporting Information

Supplementary results

This appendix was part of the submitted manuscript and has been peer reviewed.
It is posted as supplied by the authors.

Appendix to: Banks E, Yazidjoglou A, Brown S, et al. Electronic cigarettes and health outcomes: umbrella and systematic review of the global evidence. *Med J Aust* 2023; doi: 10.5694/mja2.51890.

Electronic cigarettes and health outcomes: umbrella and systematic review of evidence

Appendix

Contents

1. Methods 4
Search strategy 4
Search terms: PubMed 4
General Search – 22 July 2020 4
Search Terms: Dependence – 24 July 2020 4
Search Terms: Injuries, burns, poisoning – 24 July 2020 4
Rapid search of additional major international reviews 5
General search – 14 December 2022 5
Inclusion and exclusion criteria 6
2. Tools and methods for evaluating evidence 7
3. Characteristics of the study publications included in the top-up review 8
1. Dependence and abuse liability 9
2. Cardiovascular health outcomes 30
3. Respiratory disease 42
4. Oral Health 61
5. Developmental and reproductive 62
6. Burns and injuries 65
7. Poisonings 73
8. Mental health effects 83
9. Environmental hazards with health implications 84
10. Neurological outcomes 93
11. Less serious adverse events 94
12. Optical health 101
13. Olfactory outcomes 102
4. Studies in combined evidence synthesis - systematic umbrella and top-up review 103
Dependence and Abuse liability (52 studies) 103
Cardiovascular health outcomes (21 studies) 107
Cancer (1 study) 109
Respiratory disease (58 studies) 110
Oral Health (4 studies) 114
Developmental and reproductive (3 studies) 115
Burns and injuries (67 studies) 116

Poisonings (59 studies) 120
Mental Health (3 studies) 124
Environmental hazards with health implications (25 studies) 125
Neurological outcomes (13 studies) 127
Sleep (0 studies) 128
Less serious adverse events (47 studies) 129
Optical Health (1 study) 133
Wound healing (0 studies) 134
Olfactory outcomes (1 study) 135
Endocrine outcomes (2 studies) 136
Allergic diseases (4 studies) 137
Haematological outcomes (0 studies) 138
Smoking uptake (28 studies) 139
Smoking cessation (11 studies) 141
5. GRADE table (combined evidence from umbrella and top-up review) 143
References 147

Tables

Table 1.1. Study details: dependence and abuse liability – randomised controlled trials, cohort studies, non-randomised intervention studies, cross-sectional surveys 9
Table 2.1. Study details: cardiovascular health outcomes – meta-analyses 30
Table 2.2. Study details: cardiovascular health outcomes – randomised controlled trials, cohort and non-randomised intervention studies 31
Table 2.3. Study details: cardiovascular health outcomes – case reports 41
Table 3.1. Study details: respiratory health outcomes – randomised controlled trials, cohort studies and non-randomised intervention studies 42
Table 3.2. Study details: respiratory health outcomes – surveillance reports 51
Table 3.3. Study details: respiratory health outcomes - case reports and case series 56
Table 4.1. Study details: oral health – cohort studies 61
Table 5.1. Study details: developmental and reproductive outcomes – cohort studies and cross-sectional surveys 62
Table 6.1. Study details: burns and injuries – surveillance reports 65
Table 6.2. Study details: burns and injuries – case reports and case series 68
Table 7.1. Study details: poisoning – surveillance reports 73
Table 7.2. Study details: poisoning – case reports and case series 80
Table 8.1. Study details: mental health effects – cohort studies 83
Table 9.1 Study details: environmental hazards with health implications – controlled and natural experiments 84
Table 9.2 Study details: environmental hazards with health implications – surveillance reports 92
Table 10.1. Study details: neurological outcomes – surveillance reports 93
Table 11.1. Study details: less serious adverse events – randomised controlled trials and cohort studies 94
Table 11.2. Study details: less serious adverse events - surveillance reports 100
Table 12.1. Study details: optical health – non-randomised intervention studies 101
Table 13.1. Study details: olfactory outcomes – cross-sectional surveys 102

1. Methods

Search strategy

In addition to the database search for original research articles, a secondary search for systematic reviews/meta-analyses of relevant health outcomes was conducted using the same search terms as the primary articles. Studies found in these reviews were screened using the same eligibility criteria as the original articles.

Forward and backward citation searching was performed on included studies. Using Web of Science, Scopus and Google Scholar, references cited within included studies (backward search) and papers that cited the included study (forward search) were exported into Covidence. Studies that had previously been screened in the original database search and other duplicates were removed. Remaining studies underwent title and abstract and full text screening, data extraction, quality assessment and data synthesis as per the procedure outlined in the methods of the main manuscript.

Search terms were informed by the NASEM review[1] search strategy with additional guidance provided by librarians.

E-cigarettes delivering tetrahydrocannabinol were excluded as not within the scope of evidence required by stakeholders, including the Australian Department of Health, the National Health and Medical Research Council, and the Royal Australian College of General Practitioners. Moreover, in synthesising evidence regarding the health effects of nicotine and non-nicotine e-cigarettes, it was important to distinguish these effects from those of tetrahydrocannabinol.

The search was limited to studies published in English as there were insufficient time and resources for professional translation of non-English studies; 25 studies were excluded using this criterion during title and abstract screening and five during full text screening. It is unclear whether they would otherwise have been included in the final evidence synthesis, but the small volume renders it unlikely that our findings were significantly affected by their omission.

Search terms: PubMed

General Search – 22 July 2020

e-cigarette OR e-cigarettes OR "Electronic cigarette" OR "Electronic cigarettes" OR "Electronic nicotine de*" OR e-liquid OR "Electronic nicotine delivery system" OR vape OR vaping OR "Electronic inhalant device" OR "Electronic Nicotine Delivery Systems"[Mesh] AND ((humans[Filter]) AND (2017:2020[pdat])) AND (humans[Filter]) Filters: Humans

Search Terms: Dependence – 24 July 2020

((e-cigarette OR e-cigarettes OR "Electronic cigarette" OR "Electronic cigarettes" OR "Electronic nicotine de*" OR "Electronic nicotine delivery system" OR vape OR vaping OR e-liquid OR "Electronic inhalant device" OR "Electronic Nicotine Delivery Systems"[Mesh]) AND ("Tobacco Use Disorder" [MeSH] OR "Substance Withdrawal Syndrome" [MeSH] OR "Craving" [MeSH] OR dependence or withdrawal or craving OR appeal or addiction OR "abuse liability" OR "subjective effects" OR "smoking urge" OR "urge to smoke" OR "smoking desire" OR "desire to smoke")) AND (2017:2020[pdat])

Search Terms: Injuries, burns, poisoning – 24 July 2020

(e-cigarette OR e-cigarettes OR "Electronic cigarette" OR "Electronic cigarettes" OR "Electronic nicotine de*" OR "Electronic nicotine delivery system" OR vape OR vaping OR e-liquid OR "Electronic inhalant device" OR "Electronic Nicotine Delivery Systems"[Mesh]) AND ("Poisoning"[MeSH] OR dermal OR injury OR injuries OR explosi* OR explod* OR ingestion OR poison OR poisoning OR ingest OR burn*) AND (2017:2020[pdat])

Rapid search of additional major international reviews

A further search was conducted (at the request of one reviewer of our manuscript) to identify additional major reviews published to November 2022. PubMed and Google were searched to capture both peer reviewed articles and grey literature. The PubMed search terms are listed below and the Google search included terms such as: “electronic cigarettes’; “e-cigarettes”; “ENDS” and “vaping”. This was a rapid review rather than a systematic review update as only two databases were searched and screening was performed by only one reviewer.

General search – 14 December 2022

("electronic nicotine delivery systems"[MeSH Terms] OR ("electronic"[All Fields] AND "nicotine"[All Fields] AND "delivery"[All Fields] AND "systems"[All Fields]) OR "electronic nicotine delivery systems"[All Fields] OR "e cigarette"[All Fields] OR "ENDS"[All Fields] OR ("vaping"[MeSH Terms] OR "vaping"[All Fields] OR "vape"[All Fields] OR "electronic nicotine delivery systems"[MeSH Terms] OR ("electronic"[All Fields] AND "nicotine"[All Fields] AND "delivery"[All Fields] AND "systems"[All Fields]) OR "electronic nicotine delivery systems"[All Fields])) AND (systematicreview[Filter]

Inclusion and exclusion criteria

PICO category	Inclusion Criteria	Exclusion Criteria
Population	General population<br>Priority subgroups:<br>- Non-smoking populations<br>- Children and youth<br>- Aboriginal and Torres Strait Islander communities<br>- Current smokers	Animals<br>In vitro<br>In vivo
Intervention	Exposure to nicotine-containing or non-nicotine-containing e-cigarettes or e-liquids	Heat-not-burn and other tobacco containing products<br>Passive exposure or second- or third- hand exposure
Comparison	Never smokers (no e-cigarette or combustible tobacco products ever)<br>Former combustible tobacco smokers<br>Former e-cigarette users<br>Former dual-user<br>For some outcomes where no other comparator is possible, smoker populations will be considered	Current combustible tobacco smokers<br>Dual users
Outcomes	Primary outcomes are clinical disease endpoints, such as myocardial infarction, stroke and cancer.<br>Measures of physiological response or biological effect – such as intermediate markers of disease or health outcome (e.g., atherosclerosis, high blood pressure, lung damage), will be considered if they are likely to be specifically informative.<br>Health outcomes include:<br>Dependence<br>Abuse liability<br>Cardiovascular disease<br>Cancer<br>Respiratory disease<br>Oral disease<br>Development and reproductive effects<br>Injuries, burns and poisonings<br>Mental health<br>Environmental impacts relevant to human health e.g., fire<br>Any other health outcomes derived from the search (e.g., neurological, sleep, adverse events, optical health, wound healing, olfactory, endocrine, allergic diseases and haematological outcomes)	Studies that measure the suppression of withdrawal and craving related to combustible tobacco smoking only
Study type	Human studies<br>Published, peer-reviewed original research<br>The highest quality data will be prioritised, in the following order and dependent on the health outcome under investigation:<br>- Randomised controlled trials (including randomised crossover trials)<br>- Prospective cohort studies<br>- Case-control studies<br>- Non-randomised intervention studies (with comparison group or compared to baseline)<br>For health outcomes where epidemiological studies are not available or are not relevant, and where these types of evidence are likely to be informative, other forms of evidence listed below will be considered.<br>- Cross-sectional surveys<br>- Case reports and case series (particularly for exposure-dependent health outcomes, e.g., burns/injuries, poisonings)<br>- Grey literature/reports from passive surveillance systems	Primary evidence included in the NASEM review,[1] PHE review[2] and CSIRO review.[3]<br>Qualitative studies<br>Conference abstracts, letters, editorials, correspondence, opinion pieces, position statements<br>Case reports/series of poor quality
Follow-up period	No restrictions	
Setting	Any country	No exclusion criteria
Time period	From 2017 to July 2020 (date of search) to capture evidence published since the NASEM review.[1] As searches cannot be limited by month of year, studies published prior to July 2017 will be manually excluded.	Published before July 2017 and included in the NASEM review[1]
Language	English only	Not available in English
Other		Duplicated data<br>Unavailable full text<br>Focus on e-cigarette ingredients/toxicology (with no health outcome)<br>Focus on factors associated with e-cigarette uptake, not health outcomes<br>Prevalence study on e-cigarette use<br>Focus on perceptions of e-cigarette safety<br>Focus on e-cigarette particle distribution<br>Studies otherwise inappropriate for this section

CSIRO = Commonwealth Scientific and Industrial Research Organisation; NASEM = National Academies of Sciences, Engineering, and Medicine; PHE = Public Health England; PICO = population, intervention, comparison, outcomes.

2. Tools and methods for evaluating evidence

Assessing the evidence

Individual studies

Assess	Quality of studies
Tool	Joanna Briggs Institute (JBI) critical appraisal of study methodology

Possible ratings	Definition
High	80-100% criteria met
Moderate	50-79% criteria met
Low	<50% criteria met

Elements appraised vary by study design and include the following:

- Clear temporal relationship of variables
- Representativeness
- Comparator
- Group allocation
- Selection criteria
- Blinding
- Measurement of exposure/condition
- Management of confounding factors
- Assessment of outcomes
- Clinical detail
- Exposure/follow-up period
- Management of and accounting for follow-up
- Statistical analysis
- Trial design

Synthesised evidence

Assess	Certainty of evidence
Tool	GRADE appraisal for systematic reviews and evidence syntheses

Possible ratings	Definition
High	Confident in the evidence
Moderate	Moderately confident
Low	Limited confidence
Very low	Very little confidence

Initial certainty rated based on study design:

High (randomised controlled/crossover trial)
Moderate (case-control, cohort, NR intervention)
Low (case report/series, surveillance report)

Certainty rated down due to:

	Assessing	*Example*
Risk of bias	Methodological limitations	Low JBI ratings, conflicts of interest, small N studies
Inconsistency	Effect across studies	Contradicting outcomes
Indirectness	Addressing the research question	Lack of evidence on primary outcomes
Imprecision	Number of events	Small number of small studies
Publication bias	Evidence of bias	Only small positive studies

Assess	Conclusions based on evidence
Tool	NASEM framework for assessing levels of evidence for conclusions

Possible ratings	Definition
Conclusive evidence	High confidence, no limitations
Substantial evidence	High confidence, minor limitations
Moderate evidence	Moderate confidence, limitations
Limited evidence	Limited confidence, significant limitations
Insufficient evidence	Very little confidence, substantial uncertainty
No available evidence	No conclusion, no evidence

Rating	Supportive findings	Opposing findings	Type of studies
Conclusive	Many	None	Good-quality controlled
Substantial	Several	Few or none	Good-quality observational
			Controlled trials
Moderate	Several	Few or none	Fair-quality studies
Limited	Few	None	Fair-quality studies
	Most	Some	Any
Insufficient	Few	Some	Any
	One	NA	
No available	None	NA	NA

GRADE = Grading of Recommendations Assessment, Development and Evaluation; JBI = Joanna Briggs Institute; N = number of studies; NASEM = National Academies of Sciences, Engineering, and Medicine; NR = non-randomised.

Notes: JBI critical appraisal checklists[4] assessed methodological quality for individual studies identified in the top-up review only.
GRADE[5] and the NASEM framework[1] were applied to synthesised evidence from all sources (top-up, NASEM review[1] and other).

3. Characteristics of the study publications included in the top-up review

No studies were identified for the top-up review with the following health outcomes:

- cancer
- sleep outcomes
- wound healing
- endocrine
- allergies
- haematological

The tables in this section are modified from our larger e-cigarette health outcomes review report, with permission:

Banks E, Yazidjoglou A, Brown S, et al. Electronic cigarettes and health outcomes: systematic review of global evidence. Report for the Australian Department of Health. Canberra, Australia: National Centre for Epidemiology and Population Health, 2022. https://openresearch-repository.anu.edu.au/handle/1885/262914 (viewed April 2022).

Full references, for studies identified in both the top-up review and umbrella review, are provided in Appendix 4, grouped under relevant health outcome and presented by study type.

1. Dependence and abuse liability

Table 1.1. Study details: dependence and abuse liability – randomised controlled trials, cohort studies, non-randomised intervention studies, cross-sectional surveys

Study details (author, year, location, study type, [data source, time frame])	Sample characteristics	Intervention/exposure and comparator	Outcome measure	Results	Quality assessment, study size, conflict of interest and funding
Randomised controlled trials					
De La Garza et al., 2019<br><br>US<br><br>Randomised, double-blinded, placebo-controlled experimental trial<br><br>Study date not reported	Study size<br>15 participants<br><br>Sample<br>Tobacco dependent e-cigarette naïve smokers<br><br>Gender - n (%)<br>Male: 10/15 (66%)<br>Female: 5/15 (33%)<br><br>Age - mean (SD) years<br>50.6 (7.6)	Intervention 1<br>ENDS: 18mg/mL nicotine<br><br>Intervention 2<br>ENDS: 36mg/mL nicotine<br><br>Comparator<br>ENNDS: 0mg/mL<br><br>Materials<br>eGo devices with a 3.3V e-cigarette battery attached to a 1.5Ω dual-coil cartomizer<br><br>Virginia Pure tobacco flavoured, containing 0, 18, or 36mg/mL nicotine loaded with 1mL of a 70% propylene glycol/30%vegetable glycerin<br><br>Pattern of exposure<br>4 sessions: 10 puffs, twice with 30-minute washout. Abstinent night before	E-cigarette perception questionnaire<br>How rewarding (satisfying) is this e-cigarette dose compared to own? (mean (SD))<br><br>Which would you rather smoke-this e-cigarette dose or own cigarette? (ratio)	E-cigarette perception questionnaire<br><br>(ENNDS / 18mg/mL ENDS / 36mg/mL ENDS)<br>How rewarding (satisfying) is this e-cigarette dose compared to own? (mean (SD)): 3.1 (1.9) / 3.0 (1.8) / 2.7 (1.7)<br>Which would you rather smoke-this e-cigarette dose or own cigarette? (ratio): 3:11 / 4:11 / 4:11	Moderate methodological quality<br><br>Very small study size<br><br>Conflicts of interest<br>None declared<br><br>Funding<br>Supported by National Cancer Institute

Study details (author, year, location, study type, [data source, time frame])	Sample characteristics	Intervention/exposure and comparator	Outcome measure	Results	Quality assessment, study size, conflict of interest and funding
O'Connell et al., 2019<br><br>US<br><br>Randomised, open-label, crossover clinical trial<br><br>Study date not reported	Study size<br>15 e-cigarette naïve smokers<br><br>Sample<br>Smoke ≥10 CPD, no previous use of e-cigarettes<br><br>Gender - n (%)<br>Male: 9/15 (60%)<br>Female: 6/15 (40%)<br><br>Age - mean (SD) years<br>42.3 (12.41)	Materials<br>(1) myblu pod-system: 25mg nicotine ('freebase') tobacco flavour<br>(2) myblu pod-system: 16mg nicotine lactate tobacco flavour<br>(3) myblu pod-system: 25mg nicotine lactate tobacco flavour<br>(4) myblu pod-system: 40mg nicotine lactate tobacco flavour<br>(5) blu PRO open system: 48mg nicotine lactate tobacco flavour<br><br>Pattern of exposure<br>10 inhalations every 30s for 3s in duration	Subjective measures<br>Did you enjoy it?	Did you enjoy it? - mean (SD)<br>Mean (SD)<br>Conventional cigarette: 4.9 (1.44)<br>Myblu 40mg: 4.0 (1.36)<br>Myblu 25mg: 3.5 (1.98)<br>Myblu 16mg: 3.5 (1.46)<br>Blu PRO 48mg: 3.2 (1.81)<br>Blu PRO 25mg (freebase): 3.5 (1.87)<br>Scale: 1, not at all; 2, very little; 3, a little; 4, modestly; 5, a lot; 6, quite a lot; 7, extremely<br><br>No significant difference between the six products	Moderate methodological quality<br><br>Very small study size<br><br>Conflicts of interest<br>Full time employees of the Imperial Brands Group or Celerion. Celerion has received funding from several e-cigarette/tobacco manufacturers<br><br>Funding<br>Supported by Imperial Brands
Adriaens et al., 2018<br><br>Belgium<br><br>Randomised, crossover within-subjects trial<br><br>Study date not reported	Study size<br>30 participants<br><br>Sample<br>Smokers for at least three years (at least 10 CPD), unwilling to quit, never used e-cigarettes or heat-not-burn tobacco products<br><br>Gender - n (%)<br>Male: 20/30 (67%)<br>Female: 10/30 (33%)<br><br>Age - mean (SD) years<br>22 (3.09)	Intervention<br>ENDS: 18mg/mL nicotine, tobacco or menthol flavour<br><br>Comparator<br>Own combustible tobacco cigarette and IQOS™ (heat-not-burn product) regular flavour<br><br>Materials<br>Own tobacco cigarette, e-cigarette, IQOS™ (heat-not-burn product)<br><br>Pattern of use<br>Laboratory sessions on three consecutive days, 70-80 minutes each session. Five minutes ad lib use for each product	Modified Cigarette Evaluation Questionnaire (mCEQ)<br>Smoking satisfaction<br>Psychological reward<br>Aversion<br>Enjoyment of respiratory tract sensations<br>Craving reduction<br><br>Additional questions (visual analogue scale and open-ended questions)<br>Willing to use the product for another five minutes<br><br>Willing to keep trying or start using the product<br><br>Desire/intention to go and buy the product<br><br>Willing to consider using the product to (try to) quit smoking<br><br>Aspects missed when using the e-cigarette compared to tobacco cigarettes	Modified Cigarette Evaluation Questionnaire (mCEQ)<br>(Highest rating → Lowest rating)<br>Satisfaction: Cigarette, IQOS™, ENDS<br>Psychological reward: Cigarette, IQOS™, ENDS<br>Aversion: Cigarette, ENDS, IQOS™<br>Enjoyment of respiratory tract sensations: Cigarette, IQOS™, ENDS<br>Craving reduction: Cigarette, IQOS™, ENDS<br><br>Between-group comparisons (mCEQ)<br>*Cigarette and ENDS*<br>p<0.001: satisfaction, psychological reward, respiratory tract sensations, craving reduction<br><br>Additional questions<br>Significantly (p<0.05) higher willingness to use IQOS™ for another five minutes compared to the e-cigarette. No difference found for all other items.<br><br>Reported aspects missed when using the e-cigarette compared to tobacco cigarettes (frequency %)<br>ENDS<br>Taste, aroma, flavour, smell: 63%<br>Psychophysiological effects e.g. relaxing effects: 43%<br>Feeling/sensations of inhalation in throat and lungs: 27%<br>Nicotine and throat hit: 23%<br>Handling/gesture of smoking: 17%<br>Six participants (20%) reported no missing aspects for the e-cigarette	Low methodological quality<br><br>Very small study size<br><br>Conflicts of interest<br>None declared, but authors are Tobacco Harm Reduction (THR) advocates<br><br>Funding<br>No external funding received

<table>
<tr><th>Study details (author, year, location, study type, [data source, time frame])</th><th>Sample characteristics</th><th>Intervention/exposure and comparator</th><th>Outcome measure</th><th>Results</th><th>Quality assessment, study size, conflict of interest and funding</th></tr>
<tr>
<td>Palmer & Brandon, 2018

US

Randomised, double-blinded, balanced-placebo experimental crossover trial

Study date not reported</td>
<td><u>Study size</u>
128 participants

<u>Sample</u>
Current daily ENDS users: daily nicotine solution use for ≥30 days. Includes dual users (n=52) and former smokers (n=76)

<u>Gender - n (%)</u>
Male: 79/128 (62%)
Female: 49/128 (38%)

<u>Age - mean (SD) years</u>
36.4 (13.79)</td>
<td><u>Intervention</u>
ENDS: 12mg/mL nicotine, 50% vegetable glycerin, 50% propylene glycol, tobacco, menthol, or fruit flavour

<u>Comparator</u>
ENNDS: 0mg/mL, 50% vegetable glycerin, 50% propylene glycol, tobacco, menthol, or fruit flavour

<u>Materials</u>
eGo-style 3.6-4.2 Volt, 1100 mAh battery, 2.8-Ohm, 510-style clearomiser

<u>Pattern of exposure</u>
At least 10 puffs in 10 minutes, survey re-administered</td>
<td><u>Craving to vape/smoke (mean)</u>
Questionnaire of Smoking Urges (smoking and modified e-cigarette version)</td>
<td><u>Condition means - drug content and instructional set (nicotine or non-nicotine)</u>
<table>
<tr><th></th><th>True positive</th><th>False positive (placebo)</th><th>False negative (anti-placebo)</th><th>True negative</th></tr>
<tr><td>Craving to smoke</td><td>7.75</td><td>8.08</td><td>3.93</td><td>4.57</td></tr>
<tr><td>Craving to vape</td><td>8.00[a,b]</td><td>3.68[a]</td><td>3.84[b]</td><td>4.82</td></tr>
</table>

<u>Marginal means</u>
<table>
<tr><th></th><th>Drug Content Nicotine</th><th></th><th>Instructional Set Told Nicotine</th><th></th><th></th><th></th><th></th></tr>
<tr><th></th><th>Yes</th><th>No</th><th>Yes</th><th>No</th><th>F (N)</th><th>F (I)</th><th>F (N X I)</th></tr>
<tr><td>Craving to smoke</td><td>5.69</td><td>6.19</td><td>7.92[a]</td><td>4.25[a]</td><td>0.15</td><td>4.21*</td><td>0.02</td></tr>
<tr><td>Craving to vape</td><td>5.92</td><td>4.26</td><td>5.87</td><td>4.34</td><td>1.73</td><td>1.31</td><td>5.56*</td></tr>
</table>
N=nicotine; I=instruction
Positive difference scores represent reductions in value from pre- to post-tests
*p<0.05
Shared superscripts indicate significant differences in cell means: a: p<0.05, b: p<0.01

<u>Nicotine Dosing Estimate</u>
Smokers: higher nicotine dose estimates were associated with greater cigarette craving reduction; r (50)=0.37, p=0.007
Full sample: nicotine dose estimate was not associated with e-cigarette craving reduction; r (126)=0.15, ns</td>
<td>High methodological quality

Small study size

<u>Conflicts of interest</u>
None declared

<u>Funding</u>
University of South Florida, the National Institute on Drug Abuse, and Cancer Center & Research Institute</td>
</tr>
</table>

Study details (author, year, location, study type, [data source, time frame])	Sample characteristics	Intervention/exposure and comparator	Outcome measure	Results	Quality assessment, study size, conflict of interest and funding
Stiles et al., 2018<br><br>US<br><br>Randomised, open-label, crossover trial<br><br>Study date not reported	Study size<br>71 participants<br><br>Sample<br>E-cigarette naïve current combustible cigarette smokers (10+ menthol king size (83-85mm) or 100mm cigarettes (filtered) per day for at least last 6 months; usually smoke within 30 min of waking)<br><br>Gender - n (%)<br>Male: 44/71 (62%)<br>Female: 27/71 (38%)<br><br>Age - mean (SD) years<br>34.3 (10.2)	Intervention 1<br>ENDS: 14mg, 29mg or 36mg, menthol flavour<br><br>Intervention 2<br>Cigarettes (high-abuse liability)<br><br>Comparator<br>Nicotine gum (low abuse liability)<br><br>Materials<br>ENDS: Vuse Solo<br>Cigarettes: own<br>Gum: Nicorette White Ice Mint 4mg nicotine polacrilex<br><br>Patter of exposure<br>Home use (approx. 10 to 30 minutes ad libitum) at least 6 out of 7 days prior to laboratory visit. 12 hours abstinence prior to laboratory visit. At visit, 10 min ab libitum ENDS or cigarette, 30 minutes gum, measured up to 6 hours post-exposure	Subjective effects (overall and maximum effect (E_{max}) - mean (95% CI))<br>Product liking<br><br>Intent to use again<br><br>Liking of positive effects<br><br>Disliking of negative effects	Subjective effects - mean (95% CI)	Moderate methodological quality<br><br>Very small study size<br><br>Conflicts of interest<br>Authors full time employees of tobacco company subsidiary. Consultant services for pharmaceutical and tobacco companies<br><br>Funding<br>RJ Reynolds Vapor Company through its affiliate RJ Reynolds Tobacco Company

	ENDS				
	14mg	29mg	36mg	Cigarette	Gum
Product liking	1521.63[†§] (1314.14, 1729.12)	1426.20[†§] (1204.32, 1648.08)	1256.89[†§] (1035.52, 1478.27)	3148.10 (2933.18, 3363.02)	907.29 (692.69, 1121.89)
E_{max}	5.08[†§] (4.46, 5.70)	4.51[†] (3.86, 5.16)	4.53[†] (3.86, 5.19)	9.29 (8.65, 9.93)	3.25 (2.61, 3.89)
Intent to use again	1489.01[†§] (1346.90, 1631.12)	1534.54[†§] (1383.20, 1685.87)	1412.88[†§] (1261.88, 1563.89)	2403.50 (2256.57, 2550.43)	1143.37 (996.69, 1290.05)
E_{max}	4.40[†§] (3.99, 4.80)	4.49[†§] (4.06, 4.91)	4.25[†] (3.82, 4.68)	6.93 (6.52, 7.35)	3.32 (3.82, 4.68)
Liking of positive effects	766.72[†] (475.9, 1057.54)	1003.47[†] (709.08, 1297.87)	704.70[†] (400.05, 1009.36)	1388.31 (1102.92, 1673.70)	842.96 (542.72, 1143.21)
E_{max}	6.45[†] (5.79, 7.11)	6.44[†] (5.76, 7.12)	6.74[†] (6.01, 7.47)	8.63 (8.00, 9.27)	6.02 (5.32, 6.72)
Disliking of negative effects	596.25 (297.04, 895.46)	822.23 (512.69, 1131.77)	491.65 (207.8, 775.51)	787.93 (462.74, 1113.12)	771.89 (498.84, 1044.94)
E_{max}	5.16 (4.15, 6.17)	6.16 (5.10, 7.21)	5.17 (4.23, 6.11)	6.06 (4.94, 7.17)	6.24 (5.34, 7.13)

† Significantly different from cigarettes; p<0.05
§ Significantly different from gum; p<0.05

<table>
<tr>
<td>Hiler et al., 2017

US

Randomised, double-blinded trial

Study date not reported</td>
<td><u>Study size</u>
64 participants; 31 ENDS naïve smokers
33 ENDS experienced

<u>Sample</u>
ENDS experienced individuals: ≥3 months use, using ≥1mL of ≥8mg/mL nicotine e-liquid daily; ≤5 CPD.
ENDS naïve cigarette smokers: ≥10 CPD, <5 ENDS lifetime use

<u>Gender - n (%)</u>
Male: 45/64 (70%)
Female: 19/64 (30%)

<u>Age - mean (SD) years</u>
30.6 (9.1)</td>
<td><u>Intervention</u>
ENDS: 8, 18, 36mg/mL nicotine

<u>Comparator</u>
ENNDS: 0mg/mL nicotine

<u>Materials</u>
“eGo” 3.3-V, 1,000- mAh battery with a 1.5-Ω, dual-coil, 510-style “cartomizer”; tobacco or menthol flavoured e-liquid

<u>Patter of exposure</u>
Four sessions (order randomised), separated by 48 hours. 12 hours abstinence prior to session. Session was two 10 puffs bouts (30 second break in between puffs)</td>
<td><u>Fagerström Test for Nicotine Dependence (FTND)</u>
Modified e-cigarette appearance for ENDS experienced individuals

<u>Penn State Dependence Index (PSDI)</u>
ENDS experienced: Electronic Cigarette Dependence Index
ENDS naïve: Cigarette Dependence Index

<u>Subjective questionnaire</u>
Modified version of Hughes-Hatsukami Withdrawal Scale, Tiffany-Drobes Questionnaire of Smoking Urges (QSU; factor 1: intention to use; factor 2: anticipation of relief from withdrawal symptoms); modified for ENDS experienced individuals such that whenever the word cigarette appeared in the original, the word e-cigarette appeared instead.</td>
<td><u>Dependence scores - mean (SD)</u>
<table>
<tr><th></th><th>ENDS experienced</th><th>ENDS naïve</th><th>T statistic</th><th>P</th></tr>
<tr><td>FTND</td><td>4.3 (2.0)</td><td>4.7 (1.9)</td><td>-0.8</td><td>NS</td></tr>
<tr><td>PSDI</td><td>9.9 (3.4)</td><td>12.2 (4.0)</td><td>-2.0</td><td><0.05</td></tr>
</table>
<u>Subjective effects</u>
<table>
<tr><th></th><th colspan="2">Condition</th><th colspan="2">Group</th><th colspan="2">Condition x Group</th></tr>
<tr><th></th><th>F</th><th>P</th><th>F</th><th>P</th><th>F</th><th>P</th></tr>
<tr><td colspan="7">Hughes-Hatsukami</td></tr>
<tr><td>Anxious</td><td>5.0</td><td><0.01</td><td>10.5</td><td><0.01</td><td>0.6</td><td>NS</td></tr>
<tr><td>Craving</td><td>19.0</td><td><0.01</td><td>1.7</td><td>NS</td><td>3.6</td><td><0.05</td></tr>
<tr><td>Depression</td><td>7.7</td><td><0.01</td><td>6.0</td><td><0.05</td><td>4.7</td><td><0.01</td></tr>
<tr><td>Difficulty concentrating</td><td>8.6</td><td><0.01</td><td>3.3</td><td>NS</td><td>1.7</td><td>NS</td></tr>
<tr><td>Drowsy</td><td>6.8</td><td><0.01</td><td>0.8</td><td>NS</td><td>4.9</td><td><0.01</td></tr>
<tr><td>Hunger</td><td>0.7</td><td>NS</td><td>1.4</td><td>NS</td><td>1.7</td><td>NS</td></tr>
<tr><td>Impatient</td><td>6.2</td><td><0.01</td><td>8.4</td><td><0.05</td><td>0.4</td><td>NS</td></tr>
<tr><td>Irritable</td><td>8.5</td><td><0.01</td><td>12.1</td><td><0.01</td><td>0.0</td><td>NS</td></tr>
<tr><td>Restless</td><td>5.6</td><td><0.01</td><td>6.5</td><td><0.05</td><td>0.2</td><td>NS</td></tr>
<tr><td>Sweets</td><td>0.4</td><td>NS</td><td>1.4</td><td>NS</td><td>1.8</td><td>NS</td></tr>
<tr><td>Urge</td><td>20.8</td><td><0.01</td><td>1.7</td><td>NS</td><td>4.4</td><td><0.01</td></tr>
<tr><td colspan="7">Tiffany-Drobes QSU</td></tr>
<tr><td>Factor 1</td><td>17.5</td><td><0.01</td><td>0.74</td><td>NS</td><td>3.7</td><td><0.05</td></tr>
<tr><td>Factor 2</td><td>12.4</td><td><0.01</td><td>10.9</td><td><0.01</td><td>0.8</td><td>NS</td></tr>
<tr><td colspan="7">Direct effects</td></tr>
<tr><td>Awake</td><td>6.2</td><td><0.01</td><td>1.3</td><td>NS</td><td>3.0</td><td><0.05</td></tr>
<tr><td>Calm</td><td>10.2</td><td><0.01</td><td>1.9</td><td>NS</td><td>2.9</td><td>NS</td></tr>
<tr><td>Concentrate</td><td>5.9</td><td><0.01</td><td>3.9</td><td>NS</td><td>1.7</td><td>NS</td></tr>
<tr><td>Dizzy</td><td>7.6</td><td><0.01</td><td>0.3</td><td>NS</td><td>0.7</td><td>NS</td></tr>
<tr><td>Pleasant</td><td>4.0</td><td><0.05</td><td>1.5</td><td>NS</td><td>3.7</td><td><0.05</td></tr>
<tr><td>Reduced hunger</td><td>6.4</td><td><0.01</td><td>1.0</td><td>NS</td><td>0.7</td><td>NS</td></tr>
<tr><td>Right now</td><td>8.9</td><td><0.01</td><td>6.8</td><td><0.01</td><td>2.4</td><td>NS</td></tr>
<tr><td>Satisfy</td><td>10.4</td><td><0.01</td><td>1.1</td><td>NS</td><td>5.9</td><td><0.01</td></tr>
<tr><td>Sick</td><td>3.6</td><td><0.05</td><td>0.5</td><td>NS</td><td>0.3</td><td>NS</td></tr>
<tr><td>Taste good</td><td>4.0</td><td><0.01</td><td>1.1</td><td>NS</td><td>1.4</td><td>NS</td></tr>
</table>
</td>
<td>Moderate methodological quality

Small study size

<u>Conflicts of interest</u>
Paid consultants in litigation against tobacco industry

<u>Funding</u>
Supported by NIH</td>
</tr>
<tr><td colspan="6">Cohort studies</td></tr>
<tr>
<td>Du et al., 2019</td>
<td><u>Study size</u>
494 participants</td>
<td><u>Exposure</u></td>
<td>PSECDI</td>
<td><u>Exclusive e-cigarette users (n=412)</u></td>
<td>Low methodological quality</td>
</tr>
</table>

<table>
<tr><th>Study details (author, year, location, study type, [data source, time frame])</th><th>Sample characteristics</th><th>Intervention/exposure and comparator</th><th>Outcome measure</th><th>Results</th><th>Quality assessment, study size, conflict of interest and funding</th></tr>
<tr>
<td>US

Longitudinal cohort study

2012-2017

Online e-cigarette survey</td>
<td>Exclusive e-cigarette: 412
Poly users: 59

<u>Sample</u>
Exclusive e-cigarette: past 7-day use
Poly users: e-cigarette and any other tobacco product

<u>Gender (%)</u>
E-cigarette
Male: 278/412 (67.5%)
Female: 134/412 (32.5%)
Poly
Male: 38/59 (64.4%)
Female: 21/59 (35.6%)

<u>Mean age (SD) years</u>
E-cigarette: 41.2 (11.9)
Poly: 36.5 (11.9)</td>
<td>E-cigarette: any nicotine concentration

<u>Comparator</u>
Within participants, baseline and follow-up

<u>Materials</u>
Own brand e-cigarette

<u>Follow-up</u>
6 years
Baseline: 2012-2014
Follow-up: 2017-2018</td>
<td>E-cigarette use times per day

Time to first e-cigarette use after waking

Awaken at night to use e-cigarette

Nights per week awakened to use e-cigarette

Hard to quit e-cigarette

Strong cravings to use e-cigarette

Strong urges to use e-cigarette

Hard to keep from using e-cigarette

Felt irritable if couldn't use e-cigarette

Felt nervous, restless, or anxious if couldn't use e-cigarette</td>
<td>
<table>
<tr><th>Outcome</th><th>Baseline</th><th>Follow-up</th><th>P</th></tr>
<tr><td>PSECDI-mean (SD)</td><td>8.5 (3.4)</td><td>8.4 (3.8)</td><td>0.33</td></tr>
<tr><td>Times per day-mean (SD)</td><td>23.9 (24.7)</td><td>21.8 (23.9)</td><td>0.14</td></tr>
<tr><td>Time to first e-cigarette, mins-mean (SD)</td><td>44.5 (77.5)</td><td>41.7 (73.3)</td><td>0.54</td></tr>
<tr><td>Awaken to use e-cigarette - n (%)</td><td>29 (7.1%)</td><td>39 (9.5%)</td><td>0.10</td></tr>
<tr><td>Nights per week awaken to use e-cigarette - mean (SD)</td><td>0.3 (1.2)</td><td>0.4 (1.3)</td><td>0.22</td></tr>
<tr><td>Hard quit e-cigarette - n (%)</td><td>133 (32.4%)</td><td>83 (20.2%)</td><td><0.0001</td></tr>
<tr><td>Craving to use e-cigarette - n (%)</td><td>176 (42.8%)</td><td>182 (44.3%)</td><td>0.60</td></tr>
<tr><td>Urge to use e-cigarette - n (%)</td><td>59 (14.3%)</td><td>59 (14.3%)</td><td>1.00</td></tr>
<tr><td>Hard to keep from using e-cigarette - n (%)</td><td>44 (10.7%)</td><td>61 (14.8%)</td><td>0.04</td></tr>
<tr><td>Irritable if can't use e-cigarette - n (%)</td><td>131 (31.8%)</td><td>120 (29.1%)</td><td>0.34</td></tr>
<tr><td>Anxious if can't use e-cigarette - n (%)</td><td>137 (33.3%)</td><td>130 (31.6%)</td><td>0.53</td></tr>
</table>

<u>Poly users: e-cigarette and any tobacco product (n=59)</u>
<table>
<tr><th>Outcomes</th><th>Baseline</th><th>Follow-up</th><th>P</th><th>P (e-cigarette vs. poly)</th></tr>
<tr><td>PSECDI-mean (SD)</td><td>7.5 (3.8)</td><td>8.0 (3.9)</td><td>0.46</td><td>0.46</td></tr>
<tr><td>Times per day-mean (SD)</td><td>16.2 (14.6)</td><td>15.9 (22.9)</td><td>0.95</td><td>0.08</td></tr>
<tr><td>Time to first e-cigarette, mins-mean (SD)</td><td>64.9 (105.4)</td><td>59.0 (109.3)</td><td>0.75</td><td>0.12</td></tr>
<tr><td>Awaken to use e-cigarette - n (%)</td><td>6 (10.2%)</td><td>9 (15.3%)</td><td>0.32</td><td>0.17</td></tr>
<tr><td>Nights per week awaken to use e-cigarette - mean (SD)</td><td>0.5 (1.5)</td><td>0.5 (1.5)</td><td>0.84</td><td>0.43</td></tr>
<tr><td>Hard quit e-cigarette - n (%)</td><td>20 (33.9%)</td><td>13 (22.0%)</td><td>0.14</td><td>0.74</td></tr>
<tr><td>Craving to use e-cigarette - n (%)</td><td>21 (35.6%)</td><td>33 (55.9%)</td><td>0.003</td><td>0.09</td></tr>
<tr><td>Urge to use e-cigarette - n (%)</td><td>10 (17.0%)</td><td>10 (17.0%)</td><td>1.00</td><td>0.59</td></tr>
<tr><td>Hard to keep from using e-cigarette - n (%)</td><td>9 (15.3%)</td><td>15 (25.4%)</td><td>0.11</td><td>0.04</td></tr>
<tr><td>Irritable if can't use e-cigarette - n (%)</td><td>20 (33.9%)</td><td>23 (39.0%)</td><td>0.47</td><td>0.12</td></tr>
<tr><td>Anxious if can't use e-cigarette - n (%)</td><td>20 (33.9%)</td><td>26 (44.1%)</td><td>0.22</td><td>0.06</td></tr>
</table>
</td>
<td>Moderate study size

<u>Conflicts of interest</u>
Consultant fees and grants from pharmaceutical companies

<u>Funding</u>
Supported by the National Institute on Drug Abuse of NIH and the Center for Tobacco Products of the U.S. Food and Drug Administration</td>
</tr>
<tr><td colspan="6">Non-randomised intervention studies</td></tr>
</table>

<table>
<tr><th>Study details (author, year, location, study type, [data source, time frame])</th><th>Sample characteristics</th><th>Intervention/exposure and comparator</th><th>Outcome measure</th><th>Results</th><th>Quality assessment, study size, conflict of interest and funding</th></tr>
<tr>
<td>Hughes et al., 2020

US

Non-randomised, unblinded, within-participants pre-post clinical study

Study date not reported</td>
<td><u>Study size</u>
109 participants enrolled, 59 used in analysis (compliant)

<u>Sample</u>
Former smoker using ENDS daily history of cigarette use for at least 1 year and <6 cigarettes in last month; daily ENDS use >2 months

<u>Gender (compliant) - n (%)</u>
Male: 48/59 (81%)
Female: 11/59 (19%)

<u>Age (compliant) - mean (SD) years</u>
32 (10)</td>
<td><u>Intervention</u>
ENDS: high nicotine concentration, exact concentration unknown

<u>Comparator</u>
Pre and post

<u>Materials</u>
Own ENDS

<u>Pattern of use</u>
7 days continuous ENDS use, 6 days biologically confirmed abstinence</td>
<td><u>DSM-5 withdrawal criteria</u>
Overall and individual items: angry, anxious/nervous, increased appetite, difficulty concentrating, depressed/sad, insomnia and restlessness

<u>E-cigarette craving measures</u>
How much of the time felt urge, and now strong urge

<u>Potential withdrawal symptoms</u>
Impatient/impulsive, enjoy pleasant events less, less positive outlook, and mood swings

<u>Control symptoms</u>
Diarrhea, headache and, tremor</td>
<td>
<table>
<tr><th></th><th>Vaping
Mean</th><th>Abstinent
Mean</th><th>Increase
Mean</th><th>t</th></tr>
<tr><td><u>Withdrawal - mean</u></td><td></td><td></td><td></td><td></td></tr>
<tr><td>Overall</td><td>0.16</td><td>0.57</td><td>0.41</td><td>6.5***</td></tr>
<tr><td>Angry</td><td>0.21</td><td>0.88</td><td>0.67</td><td>6.1***</td></tr>
<tr><td>Anxious</td><td>0.14</td><td>0.59</td><td>0.45</td><td>4.1***</td></tr>
<tr><td>Increased appetite</td><td>0.13</td><td>0.62</td><td>0.49</td><td>5.1***</td></tr>
<tr><td>Difficulty concentrating</td><td>0.10</td><td>0.52</td><td>0.41</td><td>4.6***</td></tr>
<tr><td>Depressed</td><td>0.08</td><td>0.28</td><td>0.21</td><td>3.6***</td></tr>
<tr><td>Insomnia</td><td>0.26</td><td>0.38</td><td>0.12</td><td>2.1*</td></tr>
<tr><td>Restlessness</td><td>0.17</td><td>0.71</td><td>0.53</td><td>5.1***</td></tr>
<tr><td><u>E-cigarette craving - mean</u></td><td></td><td></td><td></td><td></td></tr>
<tr><td>How much of time felt urge</td><td>1.97</td><td>2.47</td><td>0.49</td><td>3.7***</td></tr>
<tr><td>How strong urge</td><td>1.94</td><td>2.62</td><td>0.68</td><td>4.9***</td></tr>
<tr><td><u>Potential withdrawal - mean</u></td><td></td><td></td><td></td><td></td></tr>
<tr><td>Impatient, impulsive</td><td>0.10</td><td>0.57</td><td>0.47</td><td>4.5***</td></tr>
<tr><td>Enjoy pleasant events less</td><td>0.03</td><td>0.31</td><td>0.28</td><td>3.1**</td></tr>
<tr><td>Less positive outlook</td><td>0.04</td><td>0.27</td><td>0.22</td><td>2.7**</td></tr>
<tr><td>Mood swings</td><td>0.05</td><td>0.41</td><td>0.36</td><td>3.9***</td></tr>
<tr><td><u>Control - mean</u></td><td></td><td></td><td></td><td></td></tr>
<tr><td>Diarrhea</td><td>0.04</td><td>0.07</td><td>0.03</td><td>0.6</td></tr>
<tr><td>Headache</td><td>0.19</td><td>0.33</td><td>0.14</td><td>1.9</td></tr>
<tr><td>Tremors</td><td>0.00</td><td>0.15</td><td>0.15</td><td>3.4**</td></tr>
</table>
*p<0.05, **p<0.01, ***p<0.001

<u>Symptoms interfered with functioning</u>
<table>
<tr><th>Vaping</th><th>Abstinent</th></tr>
<tr><td>12%</td><td>38%</td></tr>
</table>
</td>
<td>Moderate methodological quality

Small study size

<u>Conflicts of interest</u>
Consultant fees and grants from pharmaceutical companies and tobacco industry

<u>Funding</u>
National Cancer Institute</td>
</tr>
</table>

<table>
<tr><th>Study details (author, year, location, study type, [data source, time frame])</th><th>Sample characteristics</th><th>Intervention/exposure and comparator</th><th>Outcome measure</th><th>Results</th><th>Quality assessment, study size, conflict of interest and funding</th></tr>
<tr>
<td>Hughes et al., 2020

US

Non-randomised, unblinded, within-participants pre-post clinical study

Study date not reported</td>
<td><u>Study size</u>
30 participants enrolled, 18 used in analysis (compliant)

<u>Sample</u>
Never smoker using ENDS daily: <100 life cigarette use and no current "regular" use of other nicotine/tobacco products; daily ENDS use >2 months

<u>Gender (compliant) - n (%)</u>
Male: 11/18 (61%)
Female: 7/18 (39%)

<u>Age (compliant) - mean (SD) years</u>
22 (4)</td>
<td><u>Intervention</u>
ENDS: nicotine concentration unknown

<u>Comparator</u>
Pre and post

<u>Materials</u>
Own ENDS

<u>Pattern of use</u>
7 days continuous e-cigarette use, 6 days biologically confirmed abstinence</td>
<td><u>DSM-5 withdrawal criteria</u>
Overall and individual items: angry, anxious/nervous, increased appetite, difficulty concentrating, depressed/sad, insomnia and restlessness

<u>E-cigarette craving measures</u>
How much of the time felt urge, and now strong urge

<u>Potential withdrawal symptoms</u>
Impatient/impulsive, enjoy pleasant events less, less positive outlook, and mood swings

<u>Control symptoms</u>
Diarrhea, headache and, tremor</td>
<td>
<table>
<tr><th></th><th>Vaping</th><th>Abstinent</th><th>Increase</th><th></th><th></th></tr>
<tr><th></th><th>Mean</th><th>Mean</th><th>Mean</th><th>t</th><th>P</th></tr>
<tr><td><u>Withdrawal - mean</u></td><td></td><td></td><td></td><td></td><td></td></tr>
<tr><td>Overall</td><td>0.10</td><td>0.33</td><td>0.23 (0.28)</td><td>3.4</td><td>0.003</td></tr>
<tr><td>Angry</td><td>0.06</td><td>0.44</td><td>0.39 (0.53)</td><td>3.1</td><td>0.006</td></tr>
<tr><td>Anxious</td><td>0.14</td><td>0.42</td><td>0.28 (0.65)</td><td>1.8</td><td>0.09</td></tr>
<tr><td>Increased appetite</td><td>0.06</td><td>0.33</td><td>0.28 (0.71)</td><td>1.7</td><td>0.12</td></tr>
<tr><td>Difficulty concentrating</td><td>0.06</td><td>0.33</td><td>0.28 (0.52)</td><td>2.3</td><td>0.04</td></tr>
<tr><td>Depressed</td><td>0.14</td><td>0.25</td><td>0.11 (0.63)</td><td>0.7</td><td>0.47</td></tr>
<tr><td>Insomnia</td><td>0.14</td><td>0.25</td><td>0.11 (0.27)</td><td>1.7</td><td>0.10</td></tr>
<tr><td>Restlessness</td><td>0.14</td><td>0.31</td><td>0.17 (0.34)</td><td>2.1</td><td>0.05</td></tr>
<tr><td><u>E-cigarette craving - mean</u></td><td></td><td></td><td></td><td></td><td></td></tr>
<tr><td>How much of time felt urge</td><td>1.44</td><td>2.08</td><td>0.64 (0.97)</td><td>2.8</td><td>0.01</td></tr>
<tr><td>How strong urge</td><td>1.47</td><td>2.19</td><td>0.72 (1.00)</td><td>3.1</td><td>0.007</td></tr>
<tr><td><u>Potential withdrawal - mean</u></td><td></td><td></td><td></td><td></td><td></td></tr>
<tr><td>Impatient, impulsive</td><td>0.08</td><td>0.33</td><td>0.25 (0.39)</td><td>2.7</td><td>0.02</td></tr>
<tr><td>Enjoy pleasant events less</td><td>0.03</td><td>0.06</td><td>0.03 (0.27)</td><td>0.4</td><td>0.67</td></tr>
<tr><td>Less positive outlook</td><td>0.06</td><td>0.06</td><td>0.00 (0.17)</td><td>0.0</td><td>1.00</td></tr>
<tr><td>Mood swings</td><td>0.00</td><td>0.14</td><td>0.14 (0.29)</td><td>2.1</td><td>0.06</td></tr>
<tr><td><u>Control - mean</u></td><td></td><td></td><td></td><td></td><td></td></tr>
<tr><td>Diarrhea</td><td>0.08</td><td>0.19</td><td>0.11 (0.61)</td><td>0.8</td><td>0.45</td></tr>
<tr><td>Headache</td><td>0.11</td><td>0.42</td><td>0.31 (0.82)</td><td>1.6</td><td>0.13</td></tr>
<tr><td>Tremors</td><td>0.00</td><td>0.03</td><td>0.03 (0.12)</td><td>1.0</td><td>0.33</td></tr>
</table>
*Based on paired t-test (17 df)

Symptoms interfered with functioning
<table>
<tr><th>Vaping</th><th>Abstinent</th></tr>
<tr><td>11%</td><td>33%</td></tr>
</table>
</td>
<td>Moderate methodological quality

Very small study size

<u>Conflicts of interest</u>
Consultant fees and grants from pharmaceutical companies and tobacco industry

<u>Funding</u>
National Cancer Institute</td>
</tr>
</table>

<table>
<tr>
<th>Study details (author, year, location, study type, [data source, time frame])</th>
<th>Sample characteristics</th>
<th>Intervention/exposure and comparator</th>
<th>Outcome measure</th>
<th>Results</th>
<th>Quality assessment, study size, conflict of interest and funding</th>
</tr>
<tr>
<td>Cobb et al., 2019

US

Non-randomised intervention study (7 Latin-square ordered conditions)

Study date not reported</td>
<td><u>Study size</u>
20 participants

<u>Sample</u>
Healthy young adult (18-21 years) smokers (at least 5 CPD for past three months), unwilling to quit, have not regularly used e-cigarettes (using weekly or greater for one month or longer)

<u>Gender - n (%)</u>
Male: 10/20 (50%)
Female: 10/20 (50%)

<u>Age - mean (SD) years</u>
19.9 (1.1)</td>
<td><u>Intervention 1</u>
ENDS: eGo device 36mg/mL nicotine concentration, in one of three flavours

<u>Intervention 2</u>
ENNDS: eGo device 0mg/mL nicotine concentration, in one of three flavours

<u>Comparator</u>
Own brand (OB) cigarette

<u>Materials</u>
ENDS, ENNDS and own brand cigarette

<u>Pattern of use</u>
10-puff (30s interpuff interval) product administration at baseline (bout 1) and 60 minutes (bout 2)</td>
<td><u>Drug Effects Scale (visual analogue scale)</u>
“Do you feel a rush?”

“Do you like the drug effects?”

“Do you dislike the drug effects?”

“Do you feel any good drug effects?”

“Do you feel any bad drug effects?”

<u>Direct Effects of Nicotine Scale (DENS) (visual analogue scale)</u></td>
<td>
<u>Drug Effects Scale</u>
<table>
<tr><th></th><th>Condition (C)</th><th></th><th>Bout (B)</th><th></th><th>Time (T)</th><th></th></tr>
<tr><td></td><td>F</td><td>p</td><td>F</td><td>p</td><td>F</td><td>p</td></tr>
<tr><td>Rush</td><td>11.3</td><td><.0001</td><td>0.5</td><td>0.464</td><td>36.1</td><td><.0001</td></tr>
<tr><td>Like effects</td><td>5.8</td><td><.0001</td><td>0.0</td><td>0.885</td><td>16.3</td><td><.0001</td></tr>
<tr><td>Dislike effects</td><td>1.5</td><td>0.182</td><td>0.4</td><td>0.519</td><td>3.4</td><td>0.009</td></tr>
<tr><td>Feel good</td><td>9.5</td><td><.0001</td><td>0.1</td><td>0.809</td><td>20.2</td><td><.0001</td></tr>
<tr><td>Feel bad</td><td>3.5</td><td>0.002</td><td>0.2</td><td>0.621</td><td>3.6</td><td>0.006</td></tr>
</table>
<u>Drug Effects Scale (e-cigarette conditions only and bout 1)</u>
<table>
<tr><th></th><th>Flavour (F)</th><th></th><th>Nicotine (N)</th><th></th></tr>
<tr><td></td><td>F</td><td>p</td><td>F</td><td>p</td></tr>
<tr><td>Rush</td><td>4.66</td><td>0.010</td><td>35.21</td><td><.001</td></tr>
<tr><td>Like effects</td><td>2.34</td><td>0.097</td><td>16.07</td><td><.001</td></tr>
<tr><td>Dislike effects</td><td>2.06</td><td>0.128</td><td>2.46</td><td>0.117</td></tr>
<tr><td>Feel good</td><td>0.73</td><td>0.484</td><td>24.76</td><td><.001</td></tr>
<tr><td>Feel bad</td><td>3.86</td><td>0.022</td><td>8.15</td><td>0.004</td></tr>
</table>
<u>Direct Effects of Tobacco Scale</u>
<table>
<tr><th></th><th>Condition (C)</th><th></th><th>Bout (B)</th><th></th><th>Time (T)</th><th></th></tr>
<tr><td></td><td>F</td><td>p</td><td>F</td><td>p</td><td>F</td><td>p</td></tr>
<tr><td>Satisfy</td><td>42.6</td><td><.0001</td><td>17.7</td><td><.0001</td><td>26.8</td><td><.0001</td></tr>
<tr><td>Pleasant</td><td>50.0</td><td><.0001</td><td>29.8</td><td><.0001</td><td>36.6</td><td><.0001</td></tr>
<tr><td>Taste good</td><td>27.2</td><td><.0001</td><td>24.1</td><td><.0001</td><td>36.6</td><td><.0001</td></tr>
<tr><td>Calm</td><td>12.0</td><td><.0001</td><td>1.3</td><td>0.261</td><td>22.0</td><td><.0001</td></tr>
<tr><td>Like to use another</td><td>5.3</td><td><.0001</td><td>0.1</td><td>0.742</td><td>5.0</td><td>0.001</td></tr>
</table>
<u>Direct Effects of Tobacco Scale (e-cigarette conditions only and bout 1)</u>
<table>
<tr><th></th><th>Flavour (F)</th><th></th><th>Nicotine (N)</th><th></th></tr>
<tr><td></td><td>F</td><td>p</td><td>F</td><td>p</td></tr>
<tr><td>Satisfy</td><td>4.46</td><td>0.012</td><td>0.08</td><td>0.773</td></tr>
<tr><td>Pleasant</td><td>2.69</td><td>0.069</td><td>49.72</td><td><.001</td></tr>
<tr><td>Taste good</td><td>16.32</td><td><.001</td><td>29.30</td><td><.001</td></tr>
<tr><td>Calm</td><td>0.23</td><td>0.796</td><td>18.82</td><td><.001</td></tr>
<tr><td>Like to use another</td><td>5.75</td><td>0.003</td><td>10.84</td><td>0.001</td></tr>
</table>
</td>
<td>Moderate methodological quality

Very small study size

<u>Conflicts of interest</u>
Paid consultant in litigation against the tobacco industry

<u>Funding</u>
Virginia Foundation for Healthy Youth, National Cancer Institute, National Institute on Drug Abuse, Center for Tobacco Products of the US FDA</td>
</tr>
</table>

<table>
<tr><th>Study details (author, year, location, study type, [data source, time frame])</th><th>Sample characteristics</th><th>Intervention/exposure and comparator</th><th>Outcome measure</th><th>Results</th><th>Quality assessment, study size, conflict of interest and funding</th></tr>
<tr>
<td>Dowd & Tiffany, 2019

US

Non-randomised, crossover study

Study date not reported</td>
<td><u>Study size</u>
54 participants

<u>Sample</u>
Dual users: 30+ cigarettes and at least 3mL nicotine e-liquid per week for past 3 months

<u>Gender - n (%)</u>
Male: 44/54 (81%)
Female: 10/54 (19%)

<u>Age - mean (SD) years</u>
27.8 (10.2)</td>
<td><u>Intervention 1/cue 1</u>
ENDS: unknown nicotine concentration but not intentionally using non-nicotine e-liquid

<u>Intervention 2/cue 2</u>
Combustible tobacco cigarette

<u>Comparator/control cue</u>
Water

<u>Materials</u>
Own ENDS and cigarettes

<u>Pattern of use</u>
Cue in box, 8 second delay, questionnaire, sampling or not of cue (box locked or unlocked depending on computer), questionnaire

36 trials (12 trials of each cue), 30 seconds between trials</td>
<td><u>Choice behaviours under cued conditions</u>
E-cigarette craving

Tobacco cigarette craving

Spending choice time

Money spent

Latency to access cue

Puff duration

Water consumed</td>
<td><u>Behaviours under cued conditions - mean (SD)</u>
<table>
<tr><th></th><th>ENDS</th><th>Cigarette</th><th>Water</th></tr>
<tr><td>E-cigarette craving</td><td>3.5 (1.4)*</td><td>2.9 (1.3)*</td><td>3.1 (1.4)</td></tr>
<tr><td>Cigarette craving</td><td>4.0 (1.3)</td><td>4.5 (1.2)*</td><td>4.0 (1.2)</td></tr>
<tr><td>Spending choice time (ms)</td><td>4,309 (2484)*†</td><td>4,243 (1763)*</td><td>3,070 (1518)</td></tr>
<tr><td>Money spent ($)</td><td>0.09 (0.06)*†</td><td>0.13 (0.06)*</td><td>0.04 (0.04)</td></tr>
<tr><td>Latency to access cue (ms)</td><td>3,167.5 (2400.4)</td><td>3,222.7 (2504.2)</td><td>2,869.4 (1606.8)</td></tr>
<tr><td>Puff duration (ms)</td><td>5,450.0 (5241.6)</td><td>4,401.9 (3922.6)</td><td>–</td></tr>
<tr><td>Water consumed (mL)</td><td>–</td><td>–</td><td>9.8 (8.8)</td></tr>
</table>
* Significantly different compared to water trials (p<0.0001)
† Significantly different compared to combustible cigarette trials (p<0.0001)</td>
<td>High methodological quality

Very small study size

<u>Conflicts of interest</u>
None declared

<u>Funding</u>
None received</td>
</tr>
</table>

<table>
<tr><th>Study details (author, year, location, study type, [data source, time frame])</th><th>Sample characteristics</th><th>Intervention/exposure and comparator</th><th>Outcome measure</th><th>Results</th><th>Quality assessment, study size, conflict of interest and funding</th></tr>
<tr>
<td>Maloney et al., 2019

US

Non-randomised crossover study (Latin-square ordered)

Study date not reported</td>
<td><u>Study size</u>
24 participants

<u>Sample</u>
Smokers (10 or more CPD for at least a year) aged between 18 and 55 years, who were e-cigarette naïve (used <20 times in life)

<u>Gender - n (%)</u>
Male: 18/24 (75%)
Female: 6/24 (25%)

<u>Age - mean (SD) years</u>
30.9 (9.5)</td>
<td><u>Intervention 1</u>
ENDS: eGo device 36mg/mL nicotine, in one of two flavours

<u>Intervention 2</u>
ENNDS: eGo device 0mg/mL nicotine, in one of two flavours

<u>Comparator</u>
FDA-approved nicotine inhaler, own brand cigarette

<u>Materials</u>
ENDS, ENNDS, nicotine inhaler, own brand cigarette

<u>Pattern of use</u>
Four separate laboratory sessions of approx. five hours each, separated by a minimum of 48 hours. In each session, one of four study products was used</td>
<td><u>Direct Effects of Product Use Questionnaire (visual analogue scale)</u>

<u>Multiple-Choice Procedure (MCP)</u>
Eleven choices between increasing amounts of money or 10 puffs from study product used in that session

Crossover point</td>
<td>
<table>
<tr><td>Outcome measure</td><td>Condition</td><td></td><td></td><td>Time</td><td></td><td></td></tr>
<tr><td></td><td>F</td><td>P</td><td>n^2_p</td><td>F</td><td>P</td><td>n^2_p</td></tr>
<tr><td>MCP</td><td>9.75</td><td><.001</td><td>.30</td><td>1.96</td><td>ns</td><td>.08</td></tr>
<tr><td><u>Direct Effects of Product Use</u></td><td></td><td></td><td></td><td></td><td></td><td></td></tr>
<tr><td>Calm</td><td>14.86</td><td><.001</td><td>.41</td><td>11.43</td><td><.001</td><td>.35</td></tr>
<tr><td>Pleasant</td><td>34.26</td><td><.001</td><td>.62</td><td>4.59</td><td><.05</td><td>.18</td></tr>
<tr><td>Satisfy</td><td>44.20</td><td><.001</td><td>.68</td><td>2.54</td><td>ns</td><td>.11</td></tr>
<tr><td>Taste good</td><td>40.48</td><td><.001</td><td>.66</td><td>3.87</td><td><.05</td><td>.16</td></tr>
</table>
<u>MCP crossover point</u>
<table>
<tr><td>Product</td><td>Crossover point (mean (SD))</td></tr>
<tr><td>ENDS</td><td>$0.87 (1.0)</td></tr>
<tr><td>ENNDS</td><td>$0.96 (1.2)</td></tr>
<tr><td>Nicotine inhaler</td><td>$0.32 (0.6)</td></tr>
<tr><td>Own brand cigarette</td><td>$1.42 (1.4)</td></tr>
</table>
The mean MCP crossover point for the cigarette condition was significantly higher than the mean of the ENDS condition [t(23) = 3.27, p<0.01].

No significant difference between the mean crossover point in the cigarette condition and the ENNDS condition.

The mean MCP crossover point for the nicotine inhaler was significantly lower than means for the ENDS condition and the ENNDS condition [ts(23) > 2.71, ps<0.025; Bonferroni-corrected P].
</td>
<td>Moderate methodological quality

Very small study size

<u>Conflicts of interest</u>
Paid consultant in litigation against the tobacco industry

<u>Funding</u>
National Institute on Drug Abuse of the National Institutes of Health and the Center for Tobacco Products of the U.S. Food and Drug Administration</td>
</tr>
</table>

<table>
<tr><th>Study details (author, year, location, study type, [data source, time frame])</th><th>Sample characteristics</th><th>Intervention/exposure and comparator</th><th>Outcome measure</th><th>Results</th><th>Quality assessment, study size, conflict of interest and funding</th></tr>
<tr>
<td>St Helen et al., 2019

US

Non-randomised two-arm counterbalanced crossover study

Study date not reported</td>
<td><u>Study size</u>
36 participants

<u>Sample</u>
Healthy dual-users aged 21 or over, smoke at least 5 CPD over past 30 days, use the same e-cigarette device at least once daily on 15 of past 30 days, no intention to quit smoking or ENDS over next three months

<u>Gender - n (%)</u>
Male: 28/36 (78%)
Female: 8/36 (22%)

<u>Age - mean (SD) years</u>
35.4 (11.7)</td>
<td><u>Intervention</u>
ENDS: usual brand, ranging in concentration from labelled 6mg/mL to 50mg/mL (actual measured ranged from 4.5ug/mg to 52.2ug/mg)

<u>Comparator</u>
Tobacco cigarette: usual brand

<u>Materials</u>
Usual brand ENDS and cigarettes - provided by study

<u>Pattern of use</u>
Two sessions, one week apart. One puff every 30 seconds (15 puffs for cigalike, 10 for tanks), puff duration not controlled

Cigarette arm - smoked until cigarette complete</td>
<td><u>Modified Cigarette Evaluation Scale (mCES)</u>
Satisfaction
Reward
Aversive effects
Enjoyment of sensation at the back of the throat and chest
Craving reduction

<u>Questionnaire for Smoking Urges (QSU-Brief) and QSU-Brief modified for e-cigarettes</u>

Factor 1 - positive reinforcement aspects of smoking or vaping

Factor 2 - negative reinforcing aspects of smoking or vaping</td>
<td><u>mCES (mean (SD)) - administered five minutes after last puff</u>
<table>
<tr><th></th><th>ENDS</th><th>Tobacco cigarette</th><th>P</th></tr>
<tr><td>Enjoyment of sensation</td><td>4.1 (1.5)</td><td>4.6 (1.6)</td><td>0.05</td></tr>
<tr><td>Craving reduction</td><td>4.2 (1.7)</td><td>5.6 (1.7)</td><td><0.001</td></tr>
<tr><td>Satisfaction</td><td>14.3 (4.3)</td><td>16.6 (3.3)</td><td>0.001</td></tr>
<tr><td>Psychological reward</td><td>19.7 (7.6)</td><td>23.2 (6.7)</td><td>0.006</td></tr>
<tr><td>Aversion</td><td>5.1 (3.3)</td><td>5.5 (2.9)</td><td>0.44</td></tr>
</table>
<u>Subjective effects QSU - ENDS types (ENDS arm)</u>
<table>
<tr><th></th><th>QSU Factor 1 (P)</th><th>QSU Factor 2 (P)</th></tr>
<tr><td>Urge to smoke</td><td>0.035</td><td>0.009</td></tr>
<tr><td>Urge to vape</td><td>Not reported</td><td>0.004</td></tr>
</table></td>
<td>High methodological quality

Very small study size

<u>Conflicts of interest</u>
Consultant to pharmaceutical companies and has been paid expert witness in litigation against tobacco companies

<u>Funding</u>
Supported by grants from the National Institute on Drug Abuse, National Cancer Institute</td>
</tr>
</table>

<table>
<tr><th>Study details (author, year, location, study type, [data source, time frame])</th><th>Sample characteristics</th><th>Intervention/exposure and comparator</th><th>Outcome measure</th><th>Results</th><th>Quality assessment, study size, conflict of interest and funding</th></tr>
<tr>
<td>Ruther et al., 2018

Germany

Non-randomised pre-post within-subjects and between-subjects study

Study date not reported</td>
<td><u>Study size</u>
20 participants (9 in ENDS groups, 11 in cigarette group)

<u>Sample</u>
Healthy males aged over 18 years

ENDS groups: routine ENDS users for three months, not smoked cigarette for more than one month

Cigarette group: smoking cigarette for at least three years and at least 5 CPD

<u>Gender - n (%)</u>
Male: 20/20 (100%)
Female: 0/20 (0%)

<u>Age - mean (SD) years</u>
ENDS: 28.5 (8.9)
Cigarette: 26.2 (6.9)</td>
<td><u>Intervention</u>
ENDS: Three cigalike (disposable) and one tank model ENDS, 18 (1) mg/mL nicotine, industrial brand

<u>Comparator</u>
Tobacco cigarette

<u>Materials</u>
3 Cigalike models
1 tank model
Marlboro Red cigarette

<u>Pattern of use</u>
ENDS groups: four study visits at one-week intervals- different type of ENDS at each visit (non-randomised order). Duration of inhalation was four seconds, 26s interpuff interval

Cigarette group: one study visit, smoked cigarette. Duration of inhalation was two seconds, 28s interpuff interval</td>
<td><u>Craving for smoking - German version of Questionnaire on Smoking Urges (QSU-G)</u>
Two factor-specific dimensions of subjective craving for smoking on seven-level rating scale. ‘Cigarette’ and ‘smoking’ replaced with ‘e-cigarette’ and ‘vaping’ for ENDS groups

Factor 1 - intention to smoke and anticipation of positive effects from smoking (positive reinforcement)

Factor 2 - craving for smoking and anticipation of relief from negative effects of nicotine withdrawal (negative reinforcement)

<u>Fagerström Test for Nicotine Dependence (FTND)</u></td>
<td><u>QSU-G (German version of the Questionnaire on Smoking Urges) before and after consumption</u>
<table>
<tr><th>Product</th><th>Factor 1 (positive reinforcement)</th><th></th><th>Factor 2 (negative reinforcement)</th><th></th></tr>
<tr><td></td><td>Before</td><td>After</td><td>Before</td><td>After</td></tr>
<tr><td>Tobacco cigarette</td><td>4.93</td><td>2.6**</td><td>2.68</td><td>1.74*</td></tr>
<tr><td>Cigalikes</td><td>5.54</td><td>4.51</td><td>3.34</td><td>2.79</td></tr>
<tr><td>Tank model</td><td>5.56</td><td>3.45**</td><td>3.21</td><td>1.98*</td></tr>
</table>
Within-group pre-post comparisons: * Significant (p<0.05) ** Highly significant (p<0.001)

<u>Between-group comparisons - cigalike compared to tank devices</u>
<table>
<tr><th></th><th>Cigalike vs. Tank</th><th>Tank vs. Cigarettes</th></tr>
<tr><td>Factor 1</td><td>p=0.015</td><td>Non-significant</td></tr>
<tr><td>Factor 2</td><td>p=0.044</td><td>Non-significant</td></tr>
</table>
<u>FTND</u>
<table>
<tr><th></th><th>ENDS</th><th>Smoker</th></tr>
<tr><td>Mean (SD; range)</td><td>2.67 (2.18; 0-6)</td><td>2.73 (2.41; 0-8)</td></tr>
<tr><td>Physical dependence (n)</td><td></td><td></td></tr>
<tr><td>Mild</td><td>3</td><td>6</td></tr>
<tr><td>Moderate</td><td>5</td><td>4</td></tr>
<tr><td>Severe</td><td>1</td><td>1</td></tr>
</table></td>
<td>Moderate methodological quality

Very small study size

<u>Conflicts of interest</u>
None declared

<u>Funding</u>
Not reported</td>
</tr>
</table>

Spindle et al., 2018<br><br>US<br><br>Non-randomised intervention study<br><br>Study date not reported	<u>Study size</u><br>30 participants<br><br><u>Sample</u><br>Used <5 CPD, used ≥1mL of e-cigarette liquid daily, used ≥6mg/mL nicotine concentration,<br>and had used their e-cigarette ≥3 months<br><br><u>Gender - n (%)</u><br>Male: 29/30 (97%)<br>Female: 1/30 (3%)<br><br><u>Age - mean (SD) years</u><br>26.9 (7.1)	<u>Intervention</u><br>ENDS: 18mg/mL, PG:VG ratios: 100:0, 70:30, 30:70, and 0:100<br><br><u>Materials</u><br>"eGo" (3.3 V) battery with a 1.5 ohm (Ω), dual-coil, 510 "cartomizer"; Virginia Pure" tobacco flavour) 18mg/mL nicotine<br><br><u>Pattern of use</u><br>12-hour abstinence, 4 sessions. Each session, 2 bouts (60 washout) consisting of 10 puffs with 30s inter-puff-interval each	<u>Fagerström Test for Nicotine Dependence (FTND)</u><br>Modified e-cigarette appearance for ENDS experienced individuals<br><br><u>Penn State Dependence Index (PSDI)</u><br><br><u>Subjective questionnaire</u><br>Hughes-Hatsukami Withdrawal Scale<br>Tiffany-Drobes Questionnaire of Smoking Urges (QSU; factor 1: intention to use; factor 2: anticipation of relief from withdrawal symptoms);<br>general labeled magnitude scale	<u>Dependence scores - mean (SD)</u><br>FTND: 3.7 (2.4)<br>PSDI: 8.8 (4.8)<br><br><u>Subjective effects</u>	High methodological quality<br><br>Very small study size<br><br><u>Conflicts of interest</u><br>Paid consultant in litigation against the tobacco industry<br><br><u>Funding</u><br>Supported by National Institute on Drug Abuse of the National Institutes of Health and the Center for Tobacco Products of the U.S. Food and Drug Administration

	Condition		Time		Condition x Time	
	F	P	F	P	F	P
Hughes-Hatsukami						
Anxious	0.28	NS	7.87	<0.01	1.18	NS
Craving	0.34	NS	16.15	<0.001	0.97	NS
Depression	0.69	NS	3.06	NS	0.96	NS
Concentrating	0.32	NS	8.12	<0.001	0.89	NS
Drowsy	0.52	NS	9.90	<0.001	1.32	NS
Hunger	2.73	NS	6.83	<0.01	0.68	NS
Impatient	0.59	NS	5.43	<0.01	1.04	NS
Irritable	0.42	NS	3.73	<0.05	0.85	NS
Restless	0.73	NS	2.89	<0.05	1.00	NS
Sweets	0.58	NS	1.88	NS	2.04	NS
Urge	0.70	NS	15.97	<0.001	0.71	NS
Tiffany-Drobes QSU						
Factor 1	0.74	NS	19.65	<0.001	1.15	NS
Factor 2	3.04	<0.05	9.71	<0.001	1.11	NS
Direct effects						
Awake	5.53	<0.01	3.77	<0.01	2.25	<0.05
Calm	3.26	<0.05	7.32	<0.001	1.09	NS
Concentrate	5.03	<0.01	1.49	NS	1.58	NS
Dizzy	2.90	NS	5.00	<0.01	1.00	NS
Pleasant	6.94	<0.01	2.80	<0.05	0.71	NS
Reduced hunger	2.09	NS	3.68	<0.01	0.66	NS
Right now	0.11	NS	14.65	<0.001	0.41	NS
Satisfy	3.98	<0.05	4.70	<0.01	0.56	NS
Sick	0.49	NS	0.16	NS	0.81	NS
Taste good	3.14	<0.05	0.93	NS	0.69	NS
General labeled magnitude						
Flavour	1.86	NS	0.56	NS	0.02	NS
Harshness	4.74	<0.01	0.92	NS	0.03	NS

Study details (author, year, location, study type, [data source, time frame])	Sample characteristics	Intervention/exposure and comparator	Outcome measure	Results	Quality assessment, study size, conflict of interest and funding
				Throat hit 11.47 <0.001 1.53 NS 0.05 NS	
Cross-sectional surveys					
Camara-Medeiros et al., 2020<br><br>Canada<br><br>Online survey<br><br>March 2018	<u>Study size</u><br>578 participants<br><br><u>Sample - n (%)</u><br>Regular e-cigarette users<br>Never smokers: 356 (62.0%)<br>Former smokers: 101 (17.6%)<br>Current smokers (dual users): 117 (20.4%)<br><br><u>Gender - n (%)</u><br>Male: 439/578 (76.0%)<br>Female: 139/578 (24.0%)<br><br><u>Age - mean (SD) years</u><br>18.7 (2.23)	<u>Exposure</u><br>Length of time since starting vaping ≤ 1 year ago or > 1 year ago<br>Daily vaping (reported currently vaping 'daily or almost daily', number of times vaped per weekday and weekend day (<10 times per day/≥ times per day)<br><br><u>Comparator</u><br>Various<br><br><u>Materials</u><br>Own brand e-cigarette	<u>Self-perceived addiction</u><br>"Would you say that you are 'very addicted to vaping,' 'somewhat addicted to vaping,' 'not at all addicted to vaping,' or 'I don't know'"<br><br>Very addicted<br>Somewhat addicted<br>Not addicted	<u>Daily vaping</u><br>Adjusted OR (95% CI) / P<br>No: 1.00<br>Yes: 7.51 (4.55 to 12.42) / <0.0001<br><br><u>Nicotine strength</u><br>Adjusted OR (95% CI) / P<br>0 mg/mL: 1.00<br>1-8 mg/mL: 0.94 (0.47 to 1.85) / 0.0298<br>9+ mg/mL: 2.35 (1.10 to 5.03) / 0.0011<br><br><u>Time since initiating vaping</u><br>Adjusted OR (95% CI) / P<br>Less than 1 year: 1.00<br>More than 1 year: 1.62 (1.06 to 2.47) / 0.026<br><br><u># Times vaped per weekday</u><br>Adjusted OR (95% CI) / P<br><10: 1.00<br>10+: 1.17 (0.65 to 2.10) / 0.594<br><br><u># Times vaped per weekend day</u><br>Adjusted OR (95% CI) / P<br><10: 1.00<br>10+: 0.64 (0.35 to 1.18) / 0.157	Moderate methodological quality<br><br>Moderate study size<br><br><u>Conflicts of interest</u><br>None declared<br><br><u>Funding</u><br>Funded by the Ontario Ministry of Health and Long-Term Care

<table>
<tr><th>Study details (author, year, location, study type, [data source, time frame])</th><th>Sample characteristics</th><th>Intervention/exposure and comparator</th><th>Outcome measure</th><th>Results</th><th>Quality assessment, study size, conflict of interest and funding</th></tr>
<tr>
<td>Leavens et al., 2020

US

Online survey

January-March 2019</td>
<td><u>Study size</u>
593 ever JUUL users

<u>Sample</u>
Ever JUUL users (may also use other e-cigarette devices)

<u>Gender - n (%)</u>
Male: 351/584 (60.1%)
Female: 233/584 (39.9%)

<u>Age - mean (SD) years</u>
25.9 (3.1)

<u>Ethnicity - n (%)</u>
White: 454/584 (77.7%)
Black: 50/584 (8.6%)
Asian: 43/584 (7.4%)
Other: 37/584 (6.3%)</td>
<td><u>Exposure</u>
Never smokers: denied smoking in the past 3 months and smoked <100 cigarettes in their lifetime

<u>Comparator 1</u>
Former smokers: denied smoking in the past 3 months and reported smoking at least 100 cigarettes in their lifetime

<u>Comparator 2</u>
Dual users: reported smoking cigarettes at least five times per month for the past 3 months and smoking at least 100 cigarettes in their lifetime

<u>Materials</u>
Own brand e-cigarette</td>
<td><u>Penn State Electronic Cigarette Dependence Index - all e-cigarettes</u>
Score out of 20:
0-3: not dependent
4-8: low dependence
9-12: medium dependence
13+: high dependence

<u>E-cigarette demand (abuse liability) - JUUL specific</u>
If JUUL were free, how many times would you use JUUL in a single day? (One “time” consists of 15 puffs or 10 min)

What is the maximum amount you would be willing to spend for a single day’s worth of JUULing (in dollars)?

What is the max you would be willing to pay to use a JUUL for 10 minutes?</td>
<td><u>E-cigarette dependence and demand by group - mean (SD)</u>
<table>
<tr><th></th><th>Dual (n=232)</th><th>Former (n=187)</th><th>Never (n=174)</th><th>F</th><th>P</th></tr>
<tr><td>Penn State E-cigarette Dependence</td><td>8.0 (4.1)*</td><td>7.6 (4.0)*$^{+}$</td><td>7.0 (4.2) $^{+}$</td><td>3.2</td><td>0.043</td></tr>
<tr><td>Time use if free</td><td>9.6 (10.8)*</td><td>8.9 (8.4)*</td><td>6.4 (6.2)$^{+}$</td><td>6.5</td><td>0.002</td></tr>
<tr><td>Max. for day of use ($)</td><td>11.7 (12.3)*</td><td>7.9 (8.3)$^{+}$</td><td>10.6 (13.2)*$^{+}$</td><td>5.6</td><td>0.004</td></tr>
<tr><td>Max. spent for 10 minutes of use ($)</td><td>5.7 (8.0)*</td><td>2.9 (4.6)$^{+}$</td><td>4.3 (5.7)*$^{+}$</td><td>9.4</td><td><0.001</td></tr>
</table>
Symbols within each row indicate significant pairwise comparisons. Bolded values indicate significant omnibus tests.</td>
<td>Low methodological quality

Moderate study size

<u>Conflicts of interest</u>
Not reported

<u>Funding</u>
Supported by Oklahoma State University and National Institute on Drug Abuse</td>
</tr>
<tr>
<td>Shiffman & Sembower, 2020

US

Nationally representative cross-sectional survey

The Population Assessment of Tobacco and Health (PATH) Wave 1-3

2013-2016</td>
<td><u>Study size</u>
1,144 ever e-cigarette users

<u>Sample</u>
Ever used e-cigarettes “fairly regularly” and now use them every day or some days, no other tobacco product use

No demographic information reported</td>
<td><u>Exposure</u>
Exclusive e-cigarette use

<u>Comparator</u>
Daily (n=720): Reports using at least 27 days in past 30 days

Non-daily (n=431): Reports using less than 27 days in past 30 days

<u>Materials</u>
Own brand e-cigarette</td>
<td><u>PATH dependence scale</u>
Consists of 16 items (15 using a 1-5 scale ranging from “not at all true of me” to “extremely true of me”; one dichotomous item was scored 1 or 5)</td>
<td><u>E-cigarette only dependence - exclusive e-cigarette users</u>
<table>
<tr><th></th><th>Respondents</th><th>Observations</th><th>Mean</th><th>SE</th></tr>
<tr><td>Current exclusive e-cigarette</td><td>1,114</td><td>1,586</td><td>1.98</td><td>0.06</td></tr>
<tr><td>Daily e-cigarette</td><td>720</td><td>1,082</td><td>2.17</td><td>0.08</td></tr>
<tr><td>Non-daily e-cigarette</td><td>431</td><td>493</td><td>1.37</td><td>0.04</td></tr>
</table>
Adjusted analyses control for PATH wave of data collection, age, sex, ethnicity, and education</td>
<td>Low methodological quality

Moderate study size

<u>Conflicts of interest</u>
Consultants to tobacco industry

<u>Funding</u>
Supported by RAI Services Company</td>
</tr>
</table>

Study details (author, year, location, study type, [data source, time frame])	Sample characteristics	Intervention/exposure and comparator	Outcome measure	Results	Quality assessment, study size, conflict of interest and funding
Boykan et al., 2019<br><br>US<br><br>Three Stony Brook Children's outpatient offices<br><br>April 2017-April 2018	<u>Study size</u><br>42 current e-cigarette users<br><br><u>Sample</u><br>Past week exclusive users of pod and non-pod devices<br><br><u>Gender</u><br>Not reported<br><br><u>Age - (%) years</u><br>Pod / Non-pod<br>12-14: 60.0 / 40.0<br>15-17: 56.0 / 44.0<br>18-21: 22.2 / 77.8	<u>Exposure</u><br>Exclusive e-cigarette pod users<br><br><u>Comparator</u><br>Non-pod users<br><br><u>Materials</u><br>Own brand e-cigarette	If I go too long without vaping, the desire to vape interrupts my thinking<br><br>If I go too long without vaping, the desire to vape is so great that I need to vape again<br><br>If I go too long without vaping, I get angry or irritable<br><br>If I go too long without vaping, I get stressed<br><br>I need to vape when I awaken in the morning	<u>E-cigarette dependence (past-week users) - affirmative response - n (%)</u><br>(Total (n=42) / Pod (n=20) / Non-pod (n=22) / P)<br>Desire interrupts thinking: 3 (7%) / 3 (15%) / 0 (0%) / 0.060<br>Desire so great, I need to use again: 2 (5%) / 2 (10%) / 0 (0%) / 0.130<br>I get angry or irritable: 5 (12%) / 4 (20%) / 1 (5%) / 0.122<br>I get stressed: 6 (14%) / 4 (20%) / 2 (9%) / 0.320<br>Use upon waking: 6 (14%) / 6 (29%) / 0 (0%) / 0.006<br>Not all respondents answered all questions.	Low methodological quality<br><br>Small sample size<br><br><u>Conflict s of interest</u><br>Consultant fees and grants from pharmaceutical companies<br><br><u>Funding</u><br>Stony Brook University

<table>
<tr><th>Study details (author, year, location, study type, [data source, time frame])</th><th>Sample characteristics</th><th>Intervention/exposure and comparator</th><th>Outcome measure</th><th>Results</th><th>Quality assessment, study size, conflict of interest and funding</th></tr>
<tr>
<td>Hughes & Callas, 2019

US

The Population Assessment of Tobacco and Health (PATH) Wave 2

2014-2015</td>
<td><u>Study size</u>
3,210 ENDS or cigarette abstainers

<u>Sample</u>
Current or past established daily or some-day ENDS or cigarettes that had a successful or unsuccessful attempt to stop vaping or smoking completely or an attempt to reduce ENDS or cigarette use

<u>Gender (female) - n (%)</u>
ENDS: 33%
Cigarette: 53%
Dual/ENDS: 65%
Dual/cigarette: 59%
Dual/both: 60%

<u>Age - n (%) years</u>
<table>
<tr><th></th><th>18-24</th><th>25-54</th><th>55+</th></tr>
<tr><td>ENDS</td><td>13</td><td>73</td><td>14</td></tr>
<tr><td>Cigarette</td><td>7</td><td>63</td><td>31</td></tr>
<tr><td>Dual/ENDS</td><td>6</td><td>70</td><td>24</td></tr>
<tr><td>Dual/cigarette</td><td>8</td><td>70</td><td>21</td></tr>
<tr><td>Dual/ both</td><td>10</td><td>66</td><td>23</td></tr>
</table></td>
<td><u>Exposure</u>
ENDS abstinence in exclusive (ENDS) or dual users (Dual/e-cigarette)

<u>Comparator</u>
Cigarette abstinence in exclusive smokers or dual users (Dual/cigarette)

Dual ENDS and cigarette who quit both (Dual/both)

<u>Materials</u>
Own brand e-cigarette</td>
<td><u>DSM-5 criteria for tobacco withdrawal</u>
Angry, anxious, depressed, difficulty concentrating (diff conc.), eating more, insomnia, and restlessness</td>
<td><u>Prevalence of withdrawal symptoms on most recent quit attempt</u>
<table>
<tr><th>Symptom</th><th>ENDS only, quit ENDS (n=25)</th><th>Cigarette only, quit cigarette (n=2,528)</th><th>Dual, quit ENDS not cigarette (n=60)</th><th>Dual, quit cigarette not ENDS (n=355)</th><th colspan="2">Within Dual, quit ENDS & cigarette (n=242)</th></tr>
<tr><th></th><th></th><th></th><th></th><th></th><th>ENDS</th><th>Cigarette</th></tr>
<tr><td>Any (%)</td><td>40%</td><td>71%**</td><td>30%</td><td>80%***</td><td>50%</td><td>74%***</td></tr>
<tr><td>4+ (%)</td><td>25%</td><td>33%</td><td>12%</td><td>45%***</td><td>12%</td><td>43%***</td></tr>
<tr><td>No. [M (SD)]</td><td>1.7 (2.3)</td><td>2.5 (2.3)*</td><td>0.9 (1.9)</td><td>3.1 (2.4)***</td><td>1.8 (2.2)</td><td>3.0 (2.4)***</td></tr>
</table>
* <0.05, **<0.01, ***<0.001

Dual users who stopped ENDS and continued cigarette reported non-significantly less withdrawal than ENDS-only users who stopped ENDS (first vs. third columns) suggesting continuing cigarette use abated ENDS withdrawal. In contrast, dual users who stopped cigarette and continued ENDS reported more, not less, withdrawal than exclusive cigarette users who stopped cigarette (second vs. fourth columns, p<0.001 for all three withdrawal measures).

<u>Prevalence of individual symptoms on most recent quit attempt - (%)</u>
<table>
<tr><th></th><th>ENDS only, quit ENDS (n=25)</th><th>Cigarette only, quit cigarette (n=2,528)</th><th>Dual, quit ENDS not cigarette (n=60)</th><th>Dual, quit cigarette not ENDS (n=355)</th><th colspan="2">Within Dual, quit ENDS & cigarette (n=242)</th></tr>
<tr><th></th><th></th><th></th><th></th><th></th><th>ENDS</th><th>Cigarette</th></tr>
<tr><td>Angry</td><td>30%</td><td>49%</td><td>21%</td><td>62%</td><td>34%</td><td>61%</td></tr>
<tr><td>Anxious</td><td>23%</td><td>45%</td><td>14%</td><td>48%</td><td>35%</td><td>52%</td></tr>
<tr><td>Depressed</td><td>22%</td><td>19%</td><td>11%</td><td>24%</td><td>10%</td><td>19%</td></tr>
<tr><td>Diff con</td><td>12%</td><td>25%</td><td>10%</td><td>36%</td><td>21%</td><td>35%</td></tr>
<tr><td>Eat more</td><td>40%</td><td>43%</td><td>12%</td><td>49%</td><td>28%</td><td>49%</td></tr>
<tr><td>Insomnia</td><td>13%</td><td>26%</td><td>10%</td><td>33%</td><td>18%</td><td>35%</td></tr>
<tr><td>Restless</td><td>25%</td><td>43%</td><td>16%</td><td>51%</td><td>30%</td><td>53%</td></tr>
</table></td>
<td>Low methodological quality

Large sample size

<u>Conflicts of interest</u>
Consultant fees and grants from pharmaceutical companies and tobacco industry

<u>Funding</u>
National Cancer Institute</td>
</tr>
</table>

<table>
<tr>
<td>Jankowski et al., 2019

Poland

YoUng People E-Smoking Study (YUPESS)

January-March 2019</td>
<td><u>Sample size</u>
90 participants

<u>Sample</u>
Exclusive ENDS users, smokers and dual users

<u>Gender - n (%)</u>
Male: 54/90 (60%)
Female: 36/90 (40%)

<u>Age - mean (SD) years</u>
22.4 (2.2)</td>
<td><u>Exposure (n=30)</u>
Exclusive e-cigarette users, mean (SD) duration of e-cigarette use was 29.0 (24.1) months

<u>Comparator 1 (n=30)</u>
Smokers, mean (SD) smoking duration was 50.0 (32.0) months

<u>Comparator 2 (n=30)</u>
Dual users, mean (SD) smoking duration was 67.3 (30.5) months and mean (SD) duration of e-cigarette use was 27.7 (17.4) months among dual users

<u>Materials</u>
Own brand e-cigarette</td>
<td><u>Fagerström Test for Nicotine Dependence (FTND)</u>
Scored out of 10:
1-2: low dependence
3-4: low/moderate dependence
5-7: moderate dependence
8+: high dependence</td>
<td><u>Aspects of cigarette and e-cigarette dependence based on FTND (% (95% CI))</u>
<table>
<tr><td></td><td>Smokers (n=30)</td><td>Exclusive e-cigarette users (n=30)</td><td>Dual users (n=30) E-cigarette</td><td>Dual users (n=30) Smoking</td><td>P (Cigarette vs. Dual)</td><td>P (E-cigarette vs. Dual)</td></tr>
<tr><td colspan="7">How soon after waking up do you reach for a (e-) cigarette?</td></tr>
<tr><td>Within 30 min</td><td>17.9% (7.9-35.6)</td><td>53.9% (35.5-71.2)</td><td>57.1% (39.1-73.5)</td><td>42.3% (25.5-61.1)</td><td rowspan="2">0.04</td><td rowspan="2">0.8</td></tr>
<tr><td>After 30 mins</td><td>82.1% (64.4-92.1)</td><td>46.1% (28.8-64.5)</td><td>42.9% (26.5-60.9)</td><td>57.7% (38.9-74.5)</td></tr>
<tr><td colspan="7">Do you find it difficult to refrain from smoking/vaping in places where it is forbidden?</td></tr>
<tr><td>Yes</td><td>10.7% (3.7-27.2)</td><td>34.6% (19.4-53.8)</td><td>42.9% (26.5-60.9)</td><td>19.2% (8.5-37.9)</td><td rowspan="2">0.4</td><td rowspan="2">0.5</td></tr>
<tr><td>No</td><td>89.3% (72.8-96.3)</td><td>65.4% (46.2-80.6)</td><td>57.1% (39.1-73.5)</td><td>80.8% (62.1-91.5)</td></tr>
<tr><td colspan="7">Which (e-)cigarette would you hate most to give up?</td></tr>
<tr><td>First one</td><td>57.1% (39.1-73.5)</td><td>30.8% (16.5-50.0)</td><td>35.7% (20.7-54.2)</td><td>73.1% (53.9-86.3)</td><td rowspan="2">0.2</td><td rowspan="2">0.7</td></tr>
<tr><td>Any other</td><td>42.9% (26.5-60.9)</td><td>69.2% (50.0-83.5)</td><td>64.3% (45.8-79.3)</td><td>26.9% (13.7-46.1)</td></tr>
<tr><td colspan="7">How many (e-)cigarettes per day do you smoke?</td></tr>
<tr><td>10 or less</td><td>85.7% (68.5-94.3)</td><td>38.5% (22.4-57.5)</td><td>32.1% (17.9-50.7)</td><td>69.2% (50.0-83.5)</td><td rowspan="4">0.2</td><td rowspan="4">0.8</td></tr>
<tr><td>11-20</td><td>14.3% (5.7-31.5)</td><td>38.5% (22.4-57.5)</td><td>35.7% (20.7-54.2)</td><td>23.1% (11.0-42.1)</td></tr>
<tr><td>21-30</td><td>0.0% (0.0-11.3)</td><td>11.5% (4.0-28.9)</td><td>10.7% (3.7-27.2)</td><td>7.7% (2.1-24.1)</td></tr>
<tr><td>31+</td><td>0.0% (0.0-11.3)</td><td>11.5% (4.0-28.9)</td><td>21.4% (10.2-39.5)</td><td>0.0% (0.0-11.3)</td></tr>
<tr><td colspan="7">Do you smoke/vape more frequently during the first hours after waking than during the rest of the day?</td></tr>
<tr><td>Yes</td><td>14.3% (5.7-31.5)</td><td>15.4% (6.2-33.5)</td><td>39.3% (23.6-57.6)</td><td>34.6% (19.4-53.8)</td><td rowspan="2">0.8</td><td rowspan="2">0.05</td></tr>
<tr><td>No</td><td>85.7% (68.5-94.3)</td><td>84.6% (28.8-64.5)</td><td>60.7% (42.4-76.4)</td><td>65.4% (46.2-80.6)</td></tr>
<tr><td colspan="7">Do you smoke/vape if you are so ill that you are in bed most of the day?</td></tr>
<tr><td>Yes</td><td>21.4% (10.2-39.5)</td><td>34.6% (19.4-53.8)</td><td>67.9% (49.3-82.1)</td><td>42.3% (25.5-61.1)</td><td rowspan="2">0.09</td><td rowspan="2">0.01</td></tr>
<tr><td>No</td><td>78.6% (60.5-89.8)</td><td>65.4% (46.2-80.6)</td><td>67.9% (49.3-82.1)</td><td>57.7% (40.0-74.5)</td></tr>
<tr><td>FTND score mean (SD)</td><td>1.6 (1.6)</td><td>3.5 (2.6)</td><td>4.7 (2.6)</td><td>3.2 (2.2)</td><td>0.002</td><td>0.03</td></tr>
</table>
The average FTND score among exclusive e-cigarette users was over twice as high (mean 3.5 vs. 1.6) as among traditional cigarette smokers (p=0.002). The mean nicotine dependence level from e-cigarettes (mean 4.7) was higher than that from traditional cigarettes (mean 3.2; p=0.03) among dual users.</td>
<td>Moderate methodological quality

Small sample size

<u>Conflict of interest</u>
None declared

<u>Funding</u>
Medical University Silesia</td>
</tr>
<tr>
<td>Case et al., 2018

US</td>
<td><u>Study size</u>
132 participants</td>
<td><u>Exposure (n=91)</u>
Exclusive e-cigarette users</td>
<td><u>Adapted from Hooked on Nicotine Checklist</u></td>
<td><u>Cessation-related items - % (95% CI)</u>
<table>
<tr><td></td><td>Want to quit</td><td>Quit attempt</td></tr>
<tr><td>Dual user</td><td>24.2% (10.0, 48.0)</td><td>22.9% (9.1, 46.9)</td></tr>
</table></td>
<td>Low methodological quality</td>
</tr>
</table>

Study details (author, year, location, study type, [data source, time frame])	Sample characteristics	Intervention/exposure and comparator	Outcome measure	Results	Quality assessment, study size, conflict of interest and funding
Wave 4 Texas Adolescent Tobacco and Marketing Surveillance System (TATAMS)<br><br>April-June 2016	Sample<br>Past 30-day exclusive or dual users<br><br>Gender - n (%)<br>Male: 68/132 (52%)<br>Female: 64/132 (48%)<br><br>Age - mean (years)<br>15.1<br><br>Ethnicity (%)<br>White: 34.3%	Comparator 1 (n=41)<br>Dual users<br><br>Materials<br>Own e-cigarette	Fagerström Tolerance Questionnaire<br><br>Adapted Population Assessment of Tobacco and Health (PATH) Survey	E-cigarette — 53.3% (37.6, 68.4) — 45.7% (30.2, 62.1)<br><br>Symptoms of e-cigarette dependence - % (95% CI)<br>— Really need — ≤30 mins — Strong urge<br>Dual user — 32.7% (16.9, 53.9) — 16.4% (7.3, 32.7) — 35.7% (18.3, 57.8)<br>E-cigarette — 5.0% (2.2, 10.9) — 5.7% (2.5, 11.9) — 5.6% (2.5, 11.9)<br><br>When you have not used an e-cigarette, vape pen, or e-hookah for a while, do you….- % (95% CI)<br>— Find it difficult to concentrate — Feel irritable — Feel anxious<br>Dual user — 19.2% (9.1, 36.0) — 29.0% (12.8, 53.1) — 15.4% (6.9, 30.9)<br>E-cigarette — 1.6% (0.4, 5.7) — 4.7% (2.1, 10.3) — 2.8% (1.1, 7.4)<br><br>E-cigarette-specific symptoms of nicotine dependence<br>— AOR (95% CI)<br>E-cigarette — Ref<br>Dual user — 0.22 (0.07, 0.70)*<br><br>Dependence symptoms — 0.61 (0.41, 0.92)*<br><br>Past-year quit attempt<br>E-cigarette — Ref<br>Dual user — 0.25 (0.07, 0.91)*<br><br>Dependence symptoms — 0.52 (0.30, 0.92)*<br><br>*<0.05	Small study size<br><br>Conflicts of interest<br>None declared<br><br>Funding<br>Supported by a grant from the National Cancer Institute and the FDA Center for Tobacco Products (CTP)
Morean et al., 2018<br><br>US<br><br>School-based survey, pencil and paper<br><br>2017	Study size<br>520 participants<br><br>Sample<br>High school current e-cigarette users, 21.8% were also using tobacco cigarettes<br><br>Gender (%)<br>Male: 49.5%<br>Female: 50.5%<br><br>Age - mean (SD) years<br>16.22 (1.19)<br><br>Ethnicity (%)<br>White: 84.8%	Exposure<br>Past-month e-cigarettes<br><br>Comparator<br>None<br><br>Materials<br>Own e-cigarette	E-cigarette dependence scale<br>Response options included:<br>0 (never)<br>1 (rarely)<br>2 (sometimes)<br>3 (often)<br>4 (almost always)	E-cigarette dependence<br>— Mean (SD)<br>Total — 2.27 (3.84)<br>When I haven't been able to vape for a few hours, the craving gets intolerable. — 0.50 (1.00)<br>I drop everything to go out and get e-cigarettes or e-juice. — 0.30 (0.93)<br>I vape more before going into a situation where vaping is not allowed. — 0.74 (1.22)<br>I find myself reaching for e-cigarettes without thinking about it. — 0.73 (1.22)<br><br>Stronger nicotine dependence was associated with being in a higher grade (r=0.13), vaping at an earlier age (r=−0.31), vaping more frequently (r=0.47), and using higher nicotine concentrations (r=0.46), p's<.01. E-cigarette nicotine dependence also was significantly associated with using nicotine e-liquid (nicotine 0.36[0.40], nicotine-free 0.07[0.19], t=9.90) and past-month cigarette smoking (smokers 0.51[0.41], non-smokers 0.24[0.36], t=6.00), p's<.001<br><br>More than half of the sample (55.6%) endorsed experiencing some level of e-cigarette nicotine dependence	Low methodological quality<br><br>Moderate study size<br><br>Conflicts of interest<br>Previously received donate study medication from pharmaceutical companies<br><br>Funding<br>Supported in part by the FDA Center for Tobacco Products.

Study details (author, year, location, study type, [data source, time frame])	Sample characteristics	Intervention/exposure and comparator	Outcome measure	Results	Quality assessment, study size, conflict of interest and funding
Browne et al., 2017<br><br>Multiple countries<br><br>Online survey<br><br>Study date not reported	Sample size<br>436 respondents<br><br>Sample<br>Current e-cigarette users (no definition provided), 22 dual users<br><br>Gender - n (%)<br>Male: 350/436 (80.3%)<br>Female: 86/436 (19.7%)<br><br>Age - mean (SD) years<br>41.4 (13.1)	Exposure<br>Current e-cigarette use<br><br>Comparator<br>Former tobacco smoking<br><br>Materials<br>Own e-cigarette	Fagerström Test for Nicotine Dependence<br>Retrospective smoking (FTND-R) or current vaping (FTND-V)	Wilcoxon non-parametric t-tests confirmed that mean responses on all FTND-V probes were significantly less than their FTND-R counterparts (p<0.001), with the largest effect size observed for 'did/do you smoke/vape more during the first hours after waking than during the rest of the day?"	Low methodological quality<br><br>Moderate sample size<br><br>Conflict of interest<br>None declared<br><br>Funding<br>Supported by Central Queensland University

Percentages and P-values are presented as reported in original studies.
AOR = adjusted odds ratio; CI = confidence interval; CPD = cigarette(s) per day; CTP = Center for Tobacco Products; DENS = Direct Effects of Nicotine Scale; DSM = Diagnostic and Statistical Manual of Mental Disorders; E_{max} = maximum effect; ENDS = electronic nicotine delivery system; ENNDS = electronic non-nicotine delivery system; FDA = Food and Drug Administration (US); FTND = Fagerström Test for Nicotine Dependence; FTND-R = Fagerström Test for Nicotine Dependence (retrospective smoking); FTND-V = Fagerström Test for Nicotine Dependence (current vaping); max = maximum; mCEQ = Modified Cigarette Evaluation Questionnaire; mCES = Modified Cigarette Evaluation Scale; MCP = multiple-choice procedure; NIH = National Institutes of Health; NS = not significant; OB = own brand; OR = odds ratio; PATH = Population Assessment of Tobacco and Health; PG = propylene glycol; PSDI = Penn State Dependence Index; PSECDI = Penn State Electronic Cigarette Dependence Index; QSU = Questionnaire of Smoking Urges; RAI = Reynolds American Inc.; ref = reference; SD = standard deviation; SE = standard error; TATAMS = Texas Adolescent Tobacco and Marketing Surveillance System; THR = tobacco harm reduction; US = United States; VG = vegetable glycerin; YUPESS = YoUng People E-smoking Study.

2. Cardiovascular health outcomes

Table 2.1. Study details: cardiovascular health outcomes – meta-analyses

<table>
<tr><th>Study details (author, year, study type)</th><th>Inclusion and exclusion criteria</th><th>Outcome measure</th><th>Results</th><th>Quality assessment, study size, conflict of interest and funding</th></tr>
<tr>
<td>Skotsimara et al., 2019

Systematic review and meta-analysis</td>
<td>Not reported</td>
<td><u>Acute effects of ENDS</u>
Heart rate (beats/min)

Systolic blood pressure (mm Hg)

Diastolic blood pressure (mm Hg)

<u>Effects of switching to ENDS</u>
Heart rate (beats/min)

Systolic blood pressure (mm Hg)

Diastolic blood pressure (mm Hg)</td>
<td><u>Acute effects of ENDS - 5-30-minutes follow-up</u>
<table>
<tr><th></th><th>Number of studies</th><th>Number of Participants</th><th>Pooled Mean Difference (95% CI)</th><th>Heterogeneity</th></tr>
<tr><td>Heart rate</td><td>11</td><td>273</td><td>2.27 (1.64-2.89)</td><td>70%</td></tr>
<tr><td>Systolic blood pressure</td><td>7</td><td>175</td><td>2.02 (0.07-3.97)</td><td>0%</td></tr>
<tr><td>Diastolic blood pressure</td><td>7</td><td>175</td><td>2.01 (0.62-3.39)</td><td>15.7%</td></tr>
</table>
<u>Non-acute effects of ENDS - 5 days to 1-year follow-up</u>
<table>
<tr><th></th><th>Number of studies</th><th>Number of Participants</th><th>Pooled Mean Difference (95% CI)</th><th>Heterogeneity</th></tr>
<tr><td>Heart rate</td><td>3</td><td>173</td><td>-0.03 (-2.57 – 2.52)</td><td>60.7%</td></tr>
<tr><td>Systolic blood pressure</td><td>3</td><td>173</td><td>-7.00 (-9.63 – -4.37)</td><td>0%</td></tr>
<tr><td>Diastolic blood pressure</td><td>3</td><td>173</td><td>-3.65 (-5.71 – -1.59)</td><td>0%</td></tr>
</table>
</td>
<td>Moderate methodological quality

Moderate study size

<u>Conflict of interest</u>
None declared

<u>Funding</u>
No specific funding</td>
</tr>
</table>

Percentages and p-values are presented as reported in original studies.
CI = confidence interval; ENDS = electronic nicotine delivery system.

Table 2.2. Study details: cardiovascular health outcomes – randomised controlled trials, cohort and non-randomised intervention studies

<table>
<tr><th>Study details (author, year, location, study type time frame, [data source])</th><th>Sample characteristics</th><th>Intervention/exposure and comparator</th><th>Outcome measure</th><th>Results</th><th>Quality assessment, study size conflicts of interest, funding</th></tr>
<tr><td colspan="6">Randomised controlled trials</td></tr>
<tr>
<td>Cossio et al., 2020

US

Randomised, single-blinded, crossover study

Study date not reported</td>
<td><u>Study size</u>
16 participants

<u>Sample</u>
Naïve to regular tobacco products

<u>Gender - n (%)</u>
Male: 9/16 (56%)
Female: 7/16 (44%)

<u>Age - mean (SD) years</u>
24 (3)</td>
<td><u>Intervention 1</u>
ENDS: 5.4% nicotine

<u>Intervention 2</u>
ENNDS: 0% nicotine

<u>Comparator</u>
Menthol-flavoured cigarette-like pipe (Harmless Cigarette Quit Smoking Aid)

<u>Materials</u>
1. ENDS: battery (Cirrus 3, White Cloud Cigarette) and cartridge (Menthol Flavour Clear Draw Max)
2. ENNDS: battery (Cirrus 3) and cartridge (Menthol Flavour Clear Draw Max)

<u>Pattern of exposure</u>
6 minutes: 4-second inhalations every 20 seconds (18 puffs). >48-hour break between sessions. Order randomised.</td>
<td><u>Cardio-ankle vascular index</u>

<u>Flow-mediated dilation (%)</u>

<u>Haemodynamics</u>
Systolic blood pressure (mm Hg)

Diastolic blood pressure (mm Hg)</td>
<td><u>Cardio-ankle vascular index</u>
<table>
<tr><th></th><th>Control</th><th>ENNDS</th><th>ENDS</th></tr>
<tr><td>Baseline</td><td>5.7 (0.6)</td><td>5.9 (0.6)</td><td>5.8 (0.7)</td></tr>
<tr><td>Immediately post</td><td>5.9 (0.9)</td><td>6.0 (0.7)</td><td>6.2 (0.8)</td></tr>
<tr><td>1 hour post</td><td>6.0 (0.8)</td><td>6.0 (0.5)</td><td>6.0 (0.9)</td></tr>
<tr><td>2 hours post</td><td>6.0 (0.8)</td><td>6.1 (0.7)</td><td>5.9 (0.8)</td></tr>
</table>
No statistical difference in any condition

<u>Flow-mediated dilation</u>
<table>
<tr><th></th><th>Control</th><th>ENNDS</th><th>ENDS</th></tr>
<tr><td>Baseline</td><td>5.6% (2.5)</td><td>5.7% (2.8)</td><td>5.6% (1.8)</td></tr>
<tr><td>Immediately post</td><td>5.6% (2.4)</td><td>5.0% (2.0)</td><td>5.3% (1.7)</td></tr>
<tr><td>1 hour post</td><td>5.6% (2.0)</td><td>5.0% (2.2)</td><td>6.1% (2.1)</td></tr>
<tr><td>2 hours post</td><td>5.2% (3.2)</td><td>5.2% (2.5)</td><td>5.6% (2.6)</td></tr>
</table>
No statistical difference in any condition

<u>Systolic blood pressure</u>
<table>
<tr><th></th><th>Control</th><th>ENNDS</th><th>ENDS</th></tr>
<tr><td>Baseline</td><td>117 (6)</td><td>115 (8)</td><td>119 (10)</td></tr>
<tr><td>Immediately post</td><td>119 (8)</td><td>118 (10)</td><td>124 (10)</td></tr>
<tr><td>1 hour post</td><td>120 (7)</td><td>120 (8)</td><td>121 (10)</td></tr>
<tr><td>2 hours post</td><td>120 (7)</td><td>119 (10)</td><td>121 (9)</td></tr>
</table>
<u>Diastolic blood pressure</u>
<table>
<tr><th></th><th>Control</th><th>ENNDS</th><th>ENDS</th></tr>
<tr><td>Baseline</td><td>68 (3)</td><td>66 (4)</td><td>69 (4)</td></tr>
<tr><td>Immediately post</td><td>68 (6)</td><td>68 (5)</td><td>73 (5)</td></tr>
<tr><td>1 hour post</td><td>71 (6)</td><td>70 (5)</td><td>71 (6)</td></tr>
<tr><td>2 hours post</td><td>69 (5)</td><td>68 (5)</td><td>70 (5)</td></tr>
</table>
</td>
<td>Moderate methodological quality

Very small study size

<u>Conflicts of interest</u>
None declared

<u>Funding</u>
Not reported</td>
</tr>
</table>

<table>
<tr><th>Study details (author, year, location, study type time frame, [data source])</th><th>Sample characteristics</th><th>Intervention/exposure and comparator</th><th>Outcome measure</th><th>Results</th><th>Quality assessment, study size conflicts of interest, funding</th></tr>
<tr>
<td>Ikonodimis et al., 2020

Greece

Randomised controlled trial, unblinded

Study date not reported</td>
<td><u>Study size</u>
40 participants

<u>Sample</u>
Current smokers without cardiovascular disease

<u>Gender - n (%)</u>
Male: 8/40 (20%)
Female: 32/40 (80%)

<u>Age - mean (SD) years</u>
44.8 (11.3)</td>
<td><u>Intervention (n=20)</u>
ENDS: 12mg/mL nicotine

<u>Comparator (n=20)</u>
Conventional cigarette

<u>Materials</u>
ENDS: NOBACCO eGo Epsilon BDC 1100, eGo battery, 1100 mAh, operating at 3.9V
Conventional cigarette: participant's own type

<u>Pattern of exposure</u>
Complete switch to ENDS (biochemically verified) for four months</td>
<td><u>Haemodynamics</u>
Systolic blood pressure (mm Hg)

Diastolic blood pressure (mm Hg)

<u>Arterial stiffness</u>
Pulse wave velocity (m/s)

Systolic blood pressure assessed by Complior device (mm Hg)

Diastolic blood pressure assessed by Complior device (mm Hg)</td>
<td><u>Systolic blood pressure</u>
<table>
<tr><th></th><th>Pre</th><th>Post</th><th>P</th></tr>
<tr><td>ENDS</td><td>129.3 (19.1)</td><td>128.7 (19.9)</td><td>0.949</td></tr>
<tr><td>Cigarette</td><td>124.3 (19.8)</td><td>123.5 (15.1)</td><td>0.855</td></tr>
</table>
<u>Diastolic blood pressure</u>
<table>
<tr><th></th><th>Pre</th><th>Post</th><th>P</th></tr>
<tr><td>ENDS</td><td>80.5 (12.5)</td><td>79.3 (12.5)</td><td>0.641</td></tr>
<tr><td>Cigarette</td><td>75 (10.6)</td><td>72.4 (10.6)</td><td>0.267</td></tr>
</table>
<u>Pulse wave velocity</u>
<table>
<tr><th></th><th>Pre</th><th>Post</th><th>P</th></tr>
<tr><td>ENDS</td><td>10.9 (1.9)</td><td>10.1 (1.7)</td><td>0.047</td></tr>
<tr><td>Cigarette</td><td>9.5 (2.8)</td><td>10.3 (2.9)</td><td>0.028</td></tr>
</table>
<u>Systolic blood pressure assessed by Complior device</u>
<table>
<tr><th></th><th>Pre</th><th>Post</th><th>P</th></tr>
<tr><td>ENDS</td><td>119.2 (18.5)</td><td>121.2 (20.6)</td><td>0.517</td></tr>
<tr><td>Cigarette</td><td>117.5 (17.2)</td><td>115.3 (14.5)</td><td>0.484</td></tr>
</table>
<u>Diastolic blood pressure assessed by Complior device</u>
<table>
<tr><th></th><th>Pre</th><th>Post</th><th>P</th></tr>
<tr><td>ENDS</td><td>78.9 (12.5)</td><td>79.3 (11.7)</td><td>0.843</td></tr>
<tr><td>Cigarette</td><td>77.1 (13.9)</td><td>73.3 (9.9)</td><td>0.244</td></tr>
</table>
</td>
<td>Moderate methodological quality

Small study size

<u>Conflict of interest</u>
None declared

<u>Funding</u>
None received</td>
</tr>
</table>

<table>
<tr>
<td>Antoniewicz et al., 2019

Sweden

Randomised, double-blinded, crossover study

Study date not reported</td>
<td><u>Study size</u>
15 participants

<u>Sample</u>
Occasional users of tobacco products (max 10 cigarettes/month), healthy

<u>Gender - n (%)</u>
Male: 6/15 (40%)
Female: 9/15 (60%)

<u>Age - mean (SD) years</u>
26 (3)</td>
<td><u>Intervention 1</u>
ENDS: 19mg/mL nicotine

<u>Intervention 2</u>
ENNDS: 0mg/mL nicotine

<u>Comparator</u>
Before and after

<u>Materials</u>
Variable mod third generation e-cigarette (eVic-VT, Shenzhen Joyetech Co., Ltd., China) with e-liquid base primarily 49.4% propylene glycol, 44.4% vegetable glycerin, 5% ethanol, without any added flavourings

<u>Pattern of exposure</u>
30 puffs from ENDS for 30 min, with each puff lasting approximately three seconds; measurements up to 6 hours following exposure</td>
<td><u>Haemodynamics</u>
Heart rate (beats/min)

Blood pressure (mm Hg)

<u>Arterial stiffness</u>
Pulse wave velocity (m/s)

Heart-rate corrected augmentation index (%)</td>
<td><u>Heart rate</u>
<table>
<tr><th></th><th>ENDS</th><th>ENNDS</th><th>P (time)</th><th>P (time x exposure)</th></tr>
<tr><td>Baseline</td><td>65.4 (8.5)</td><td>63.8 (9.7)</td><td></td><td></td></tr>
<tr><td>0 mins</td><td>71.7 (11.3*)</td><td>64 (10.7)</td><td></td><td></td></tr>
<tr><td>10 mins</td><td>70 (12.4*)</td><td>63.3 (12.2)</td><td></td><td></td></tr>
<tr><td>20 mins</td><td>69.7 (12.9*)</td><td>62.7 (8.4)</td><td>0.015</td><td>0.001</td></tr>
<tr><td>30 mins</td><td>65.7 (10.7)</td><td>62.3 (9.2)</td><td></td><td></td></tr>
<tr><td>2 hours</td><td>64 (9.9)</td><td>61.5 (9.4)</td><td></td><td></td></tr>
<tr><td>4 hours</td><td>67.6 (10.9)</td><td>64.1 (9.9)</td><td></td><td></td></tr>
</table>
<u>Systolic blood pressure</u>
<table>
<tr><th></th><th>ENDS</th><th>ENNDS</th><th>P (time)</th><th>P (time x exposure)</th></tr>
<tr><td>Baseline</td><td>109.4 (9.5)</td><td>109.3 (10.3)</td><td></td><td></td></tr>
<tr><td>0 mins</td><td>119.3 (9.5†)</td><td>114.5 (13.2†)</td><td></td><td></td></tr>
<tr><td>10 mins</td><td>117.4 (13†)</td><td>111.2 (16.1†)</td><td></td><td></td></tr>
<tr><td>20 mins</td><td>113.7 (10.3)</td><td>109.3 (15.5)</td><td><0.001</td><td>0.227</td></tr>
<tr><td>30 mins</td><td>114.5 (12)</td><td>108.8 (15.4)</td><td></td><td></td></tr>
<tr><td>2 hours</td><td>111.1 (10.1)</td><td>109 (10.2)</td><td></td><td></td></tr>
<tr><td>4 hours</td><td>109.1 (9.5)</td><td>108.8 (11.7)</td><td></td><td></td></tr>
</table>
<u>Diastolic blood pressure</u>
<table>
<tr><th></th><th>ENDS</th><th>ENNDS</th><th>P (time)</th><th>P (time x exposure)</th></tr>
<tr><td>Baseline</td><td>70.3 (5.7)</td><td>70.2 (5.8)</td><td></td><td></td></tr>
<tr><td>0 mins</td><td>78.9 (5.9†)</td><td>74.5 (6.9†)</td><td></td><td></td></tr>
<tr><td>10 mins</td><td>77.7 (6.6†)</td><td>72.7 (8.2†)</td><td></td><td></td></tr>
<tr><td>20 mins</td><td>76.5 (6.6†)</td><td>71.1 (8.1†)</td><td><0.001</td><td>0.062</td></tr>
<tr><td>30 mins</td><td>74.9 (5.8†)</td><td>72.2 (8†)</td><td></td><td></td></tr>
<tr><td>2 hours</td><td>72.6 (5.4)</td><td>72 (6.5)</td><td></td><td></td></tr>
<tr><td>4 hours</td><td>70.5 (6.6)</td><td>69.8 (6.6)</td><td></td><td></td></tr>
</table>
<u>Pulse wave velocity</u>
<table>
<tr><th></th><th>ENDS</th><th>ENNDS</th><th>P (time)</th><th>P (time x exposure)</th></tr>
<tr><td>Baseline</td><td>5.8 (0.8)</td><td>6.2 (0.9)</td><td></td><td></td></tr>
<tr><td>0 mins</td><td>6.4 (0.8*)</td><td>6.4 (1)</td><td></td><td></td></tr>
<tr><td>10 mins</td><td>6.3 (0.9*)</td><td>6.2 (0.9)</td><td></td><td></td></tr>
<tr><td>20 mins</td><td>6.1 (0.9*)</td><td>6.1 (0.8)</td><td><0.001</td><td>0.037</td></tr>
<tr><td>30 mins</td><td>6 (0.8)</td><td>6.1 (0.9)</td><td></td><td></td></tr>
<tr><td>2 hours</td><td>5.8 (0.8)</td><td>6.1 (0.8)</td><td></td><td></td></tr>
<tr><td>4 hours</td><td>5.8 (0.9)</td><td>6 (0.8)</td><td></td><td></td></tr>
</table>
<u>Heart-rate corrected augmentation index</u>
<table>
<tr><th></th><th>ENDS</th><th>ENNDS</th><th>P (time)</th><th>P (time x exposure)</th></tr>
</table></td>
<td>High methodological quality

Very small study size

<u>Conflicts of interest</u>
None declared

<u>Funding</u>
Supported by the Swedish Heart and Lung Association, the Swedish Society of Medicine, the Swedish Heart-Lung Foundation and Stockholm County Council</td>
</tr>
</table>

<table>
<tr><th>Study details (author, year, location, study type time frame, [data source])</th><th>Sample characteristics</th><th>Intervention/exposure and comparator</th><th>Outcome measure</th><th>Results</th><th>Quality assessment, study size conflicts of interest, funding</th></tr>
<tr><td></td><td></td><td></td><td></td><td>
<table>
<tr><td>Baseline</td><td>− 5.1% (9.5)</td><td>− 2% (9.2)</td><td></td><td></td></tr>
<tr><td>0 mins</td><td>5.7% (11*)</td><td>0.6% (12.8)</td><td></td><td></td></tr>
<tr><td>10 mins</td><td>3.9% (13.2*)</td><td>0% (10.7)</td><td></td><td></td></tr>
<tr><td>20 mins</td><td>2% (11.1*)</td><td>− 0.7% (12.9)</td><td><0.001</td><td>0.006</td></tr>
<tr><td>30 mins</td><td>1.9% (10.1)</td><td>− 0.3% (10.7)</td><td></td><td></td></tr>
<tr><td>2 hours</td><td>− 2.6% (11*)</td><td>− 3.9% (10.7)</td><td></td><td></td></tr>
<tr><td>4 hours</td><td>− 3.8% (10.4)</td><td>− 2% (9.5)</td><td></td><td></td></tr>
</table>
*Denotes significant change from baseline due to exposure (contrast for ‘time × exposure’)
†Denotes significant change from baseline, not influenced by exposure (contrast for ‘time’)</td><td></td></tr>
</table>

<table>
<tr><th>Study details (author, year, location, study type time frame, [data source])</th><th>Sample characteristics</th><th>Intervention/exposure and comparator</th><th>Outcome measure</th><th>Results</th><th>Quality assessment, study size conflicts of interest, funding</th></tr>
<tr>
<td>Kerr et al., 2019

UK

Single-centre, prospective, randomised crossover study

June-December 2016

Laboratory study</td>
<td>Study size
20 participants

Sample
Habitual tobacco smokers of one or more CPD

Gender - n (%)
Male: 20/20 (100%)
Female: 0/20 (0%)

Age - mean (SD) years
31.6 (10.5)</td>
<td>Intervention 1
ENDS: 18mg/mL nicotine, tobacco flavoured

Intervention 2
Conventional cigarette

Comparator
Before session

Materials
ENDS: SmokeMax, second generation; 1300mAh variable voltage rechargeable battery
Conventional cigarette: participant's own type

Pattern of exposure
15 puffs</td>
<td>Haemodynamic parameters
Heart rate (beats/min)

Systolic blood pressure (mm Hg)

Diastolic blood pressure (mm Hg)

Reactive hyperaemia index (RHI)

Pulse wave amplitude (PWA)-occluded and control arms

Arterial stiffness
Augmentation index (%)

Augmentation index corrected for heart rate (AIx75) (%)</td>
<td>Heart rate - mean (SD)
<table><tr><th></th><th>Pre</th><th>Post</th><th>Change</th><th>P</th></tr>
<tr><td>ENDS</td><td>65 (9)</td><td>73 (8)</td><td>8 (5)</td><td><0.001</td></tr>
<tr><td>Cigarette</td><td>64 (8)</td><td>86 (13)</td><td>23 (12)</td><td><0.001</td></tr></table>
Systolic blood pressure - mean (SD)
<table><tr><th></th><th>Pre</th><th>Post</th><th>Change</th><th>P</th></tr>
<tr><td>ENDS</td><td>124 (12)</td><td>123 (11)</td><td>-1 (6)</td><td>0.431</td></tr>
<tr><td>Cigarette</td><td>121 (14)</td><td>125 (14)</td><td>4 (9)</td><td>0.058</td></tr></table>
Diastolic blood pressure - mean (SD)
<table><tr><th></th><th>Pre</th><th>Post</th><th>Change</th><th>P</th></tr>
<tr><td>ENDS</td><td>80 (11)</td><td>80 (10)</td><td>0 (5)</td><td>0.950</td></tr>
<tr><td>Cigarette</td><td>75 (11)</td><td>77 (10)</td><td>2 (5)</td><td>0.167</td></tr></table>
Reactive hyperaemia index - mean (SD)
<table><tr><th></th><th>Pre</th><th>Post</th><th>Change</th><th>P</th></tr>
<tr><td>ENDS</td><td>1.68 (0.33)</td><td>1.96 (0.44)</td><td>0.28 (0.38)</td><td>0.006</td></tr>
<tr><td>Cigarette</td><td>1.86 (0.47)</td><td>1.96 (0.51)</td><td>0.10 (0.44)</td><td>0.156</td></tr></table>
Pulse wave amplitude - occluded arm - mean (SD)
<table><tr><th></th><th>Pre</th><th>Post</th><th>Change</th><th>P</th></tr>
<tr><td>ENDS</td><td>860 (397)</td><td>465 (359)</td><td>-395 (310)</td><td><0.001</td></tr>
<tr><td>Cigarette</td><td>895 (392)</td><td>437 (387)</td><td>-458 (324)</td><td><0.001</td></tr></table>
Pulse wave amplitude - control arm - mean (SD)
<table><tr><th></th><th>Pre</th><th>Post</th><th>Change</th><th>P</th></tr>
<tr><td>ENDS</td><td>906 (434)</td><td>5070 (399)</td><td>-399 (353)</td><td>0.001</td></tr>
<tr><td>Cigarette</td><td>966 (451)</td><td>475 (396)</td><td>-492 (340)</td><td><0.001</td></tr></table>
Augmentation index - mean (SD)
<table><tr><th></th><th>Pre</th><th>Post</th><th>Change</th><th>P</th></tr>
<tr><td>ENDS</td><td>-10.5% (13.2)</td><td>-6.9% (13.5)</td><td>3.7% (5.7)</td><td>0.010</td></tr>
<tr><td>Cigarette</td><td>-9.0% (10.0)</td><td>-10.9% (13.5)</td><td>-1.9% (7.4)</td><td>0.265</td></tr></table>
Augmentation index corrected for heart rate - mean (SD)
<table><tr><th></th><th>Pre</th><th>Post</th><th>Change</th><th>P</th></tr>
<tr><td>ENDS</td><td>-16.6% (14.5)</td><td>-14.3% (14.6)</td><td>2.3% (6.5)</td><td>0.131</td></tr>
<tr><td>Cigarette</td><td>-15.6% (10.4)</td><td>-16.2% (13.9)</td><td>0.7% (7.8)</td><td>0.709</td></tr></table>
</td>
<td>Moderate methodological quality

Very small study size

Conflicts of interest
None declared

Funding
Authors supported by British Heart Foundation Centre of Research Excellence</td>
</tr>
</table>

Study details (author, year, location, study type time frame, [data source])	Sample characteristics	Intervention/exposure and comparator	Outcome measure	Results	Quality assessment, study size conflicts of interest, funding
Chaumont et al., 2018<br><br>Belgium<br><br>Randomised, single-blinded, placebo controlled, three period crossover study<br><br>2017	Study size<br>25 participants<br><br>Sample<br>Healthy occasional tobacco smokers<br><br>Gender - n (%)<br>Male: 18/25 (72%)<br>Female: 7/25 (28%)<br><br>Age - mean (SD) years<br>23 (0.4)	Intervention 1<br>ENDS: 3mg/mL nicotine<br><br>Intervention 2<br>ENNDS: 0mg/mL nicotine<br><br>Comparator<br>Sham vaping (device with power off)<br><br>Materials<br>Last generation high-power vaping device, 60 watts (0.4Ω dual coils)<br><br>Pattern of exposure<br>4 second puffs at 30 second intervals, 25 times, order randomised	Haemodynamics<br>Heart rate (beats/min)<br><br>Humeral systolic blood pressure (mm Hg)<br><br>Humeral diastolic blood pressure (mm Hg)<br><br>Arterial stiffness<br>Aortic systolic blood pressure (mm Hg)<br><br>Aortic diastolic blood pressure (mm Hg)<br><br>Aortic pulse pressure (mm Hg)<br><br>Augmentation index corrected for heart rate (AIx75) (%)<br><br>Carotid-femoral Pulse Wave Velocity (PWV) (m/s)<br><br>Subendocardial viability ratio (SEVR)	See tables below	Moderate methodological quality<br><br>Very small study size<br><br>Conflicts of interest<br>None declared<br><br>Funding<br>Supported by the "Fonds Erasme pour la Recherche Médicale"; "Fondation pour la Chirurgie Cardiaque"; "Fondation Emile Saucez-René Van Poucke"; "Prix Docteur & Mrs Rene Tagnon"; "Fondation IRIS"; the "Prix de l'Association André Vésale"; Astra Zeneca; "Fonds Fruit de Deux Vies'; "Fond David and Alice Van Buuren"

Haemodynamic parameters - mean (SEM)

	ENNDS	ENDS	Sham	P
Heart rate	60 (2)	59 (2)	60 (2)	>0.7
Systolic blood pressure	110 (2)	109 (1)	110 (2)	>0.8
Diastolic blood pressure	68 (2)	68 (1)	68 (1)	>0.9

Arterial stiffness indices - mean (SEM)

	ENNDS	ENDS	Sham	P
Aortic systolic blood pressure	95 (2)	94 (1)	94 (2)	>0.8
Aortic diastolic blood pressure	69 (1)	69 (1)	68 (1)	>0.6
Aortic pulse pressure	26 (1)	26 (1)	26 (1)	>0.9
AIx75	−4.5% (1.9)	−3.5% (1.5)	−3.4% (2.1)	>0.6
Carotid-femoral PWV	4.9 (0.1)	4.9 (0.1)	5 (0.1)	>0.6
SEVR	184 (8)	193 (7)	184 (8)	>0.3

Study details (author, year, location, study type time frame, [data source])	Sample characteristics	Intervention/exposure and comparator	Outcome measure	Results	Quality assessment, study size conflicts of interest, funding
Franzen et al., 2018<br><br>Germany<br><br>Single-centre pilot, randomised, double-blinded, crossover study<br><br>Study date not reported	Study size<br>15 participants<br><br>Sample<br>Active traditional cigarette smokers; average (SD) pack years 2.9 (1.5)<br><br>Gender - n (%)<br>Male: 5/15 (33%)<br>Female: 10/15 (67%)<br><br>Age - mean (SD) years<br>22.9 (3.5)	Intervention 1<br>ENDS: 24mg/mL nicotine, 55% propylene glycol and 35% glycerin, tobacco flavour<br><br>Intervention 2<br>ENNDS: 0mg/mL nicotine, 55% propylene glycol and 35% glycerin, tobacco flavour<br><br>Intervention 3<br>Conventional cigarette<br><br>Comparator<br>Before session<br><br>Materials<br>Tobacco cigarette: Philip Morris<br>ENDS and ENNDS: DIPSE, eGo-T CE4 vaporizer (third generation), 3.3 volts, 1.5 ohms and 7.26 watts<br><br>Pattern of exposure<br>Minimum one puff every 30 seconds for 10 puffs. Puff had to last 4 seconds. Order randomised.	Haemodynamic parameters<br>Heart rate (beats/min)<br><br>Systolic blood pressure (mm Hg)<br><br>Diastolic blood pressure (mm Hg)<br><br>Peripheral pulse pressure (mm Hg)<br><br>Arterial stiffness<br>Central systolic blood pressure (mm Hg)<br><br>Central diastolic blood pressure (mm Hg)<br><br>Augmentation index corrected for heart rate (AIx75) (%)<br><br>Pulse wave velocity (m/s)	Heart Rate<br>ENDS: significant increase (>12%; p<0.05) 45-minute follow-up<br>ENNDS: significant decrease (p<0.05) 110-minute follow-up<br><br>Systolic Blood Pressure<br>ENDS: significant increase (>3%; p<0.05) 40-minute follow-up<br>ENNDS: no change from baseline (p>0.05)<br><br>Diastolic Blood Pressure<br>ENDS: no change from baseline (p>0.05)<br>ENNDS: decreased (>4%, p<0.05) 30-minute follow-up<br><br>Peripheral Pulse Pressure<br>ENDS: significant increase (p<0.05) 30-minute follow-up<br>ENNDS: no change from baseline (p>0.05)<br><br>Central Systolic Blood Pressure<br>ENDS: no change from baseline (p>0.05)<br>ENNDS: no change from baseline (p>0.05)<br><br>Central Diastolic Blood Pressure<br>ENDS: no change from baseline (p>0.05)<br>ENNDS: significantly decreased (p<0.05) 30-minute follow-up<br><br>Augmentation index corrected for heart rate<br>ENDS: significantly increased (p<0.05) 90-minute follow-up<br>ENNDS: no change from baseline (p>0.05)<br><br>Pulse Wave Velocity<br>ENDS: significant increase (p<0.05) 15-minute follow-up<br>ENNDS: no change from baseline (p>0.05)	Moderate methodological quality<br><br>Very small study size<br><br>Conflicts of interest<br>None declared<br><br>Funding<br>Medizinische Klinik III of the Universitaetsklinikum Schleswig-Holstein

Study details (author, year, location, study type time frame, [data source])	Sample characteristics	Intervention/exposure and comparator	Outcome measure	Results	Quality assessment, study size conflicts of interest, funding
Staudt et al., 2018<br><br>US<br><br>Randomised (unequal), before-and-after study<br><br>Study date not reported	Study size<br>10 participants<br><br>Sample<br>Never smokers, self-reported history and confirmed by absence of tobacco metabolites in urine<br><br>Gender - n (%)<br>Male: 5/10 (50%)<br>Female: 5/10 (50%)<br><br>Age - mean (SD) years<br>40.2 (9.7)	Intervention 1 (n=7)<br>ENDS: nicotine concentration unknown<br><br>Intervention 2 (n=3)<br>ENNDS<br><br>Comparator<br>Before session<br><br>Materials<br>Blu branded ENDS and ENNDS<br><br>Pattern of exposure<br>10 puffs, 30 minutes rest, 10 puffs	Haemodynamics<br>Heart rate (beats/min)<br><br>Mean Arterial Pressure (MAP) (mm Hg)	Heart Rate<br>| | 1st inhalation - baseline | 2nd inhalation - baseline | | ENDS | -0.1 (4.0) | 0.1 (7.8) | | ENNDS | -0.3 (2.5) | -3.7 (10.4) | | P | 0.9 | 0.6 | <br>Mean Arterial Pressure (MAP)<br>| | 1st inhalation - baseline | 2nd inhalation - baseline | | ENDS | 1.3 (4.7) | 4.6 (5.1) | | ENNDS | 1.6 (3.7) | 5.6 (4.5) | | P | 0.2 | 0.3 |	Moderate methodological quality<br><br>Very small study size<br><br>Conflicts of interest<br>None declared<br><br>Funding<br>Supported by NIH and the Family Smoking Prevention and Tobacco Control Act

<table>
<tr><th>Study details (author, year, location, study type time frame, [data source])</th><th>Sample characteristics</th><th>Intervention/exposure and comparator</th><th>Outcome measure</th><th>Results</th><th>Quality assessment, study size conflicts of interest, funding</th></tr>
<tr>
<td>Moheimani et al., 2017

US

Randomised, open-label, crossover study

Study date not reported</td>
<td><u>Study size</u>
39 participants enrolled, 33 included, 4 lost to follow-up

<u>Sample</u>
No current (within 1 year) e-cigarette or combustible cigarette use

<u>Gender - n (%)</u>
Male: 13/33 (39%)
Female: 20/33 (61%)

<u>Age - mean (SD) years</u>
26.3 (0.9)</td>
<td><u>Intervention 1</u>
ENDS: 1.2% nicotine

<u>Intervention 2</u>
ENNDS: 0% nicotine

<u>Comparator</u>
E-cigarette without e-liquid (sham)

<u>Materials</u>
Greensmoke cigalike with tobacco-flavoured liquid or 1.0 Ω eGo-One by Joyetech with strawberry flavouring

<u>Pattern of exposure</u>
Three x 30 minute (60 puffs) sessions separated by a 4-week washout. Order randomised.</td>
<td><u>Heart rate variability</u>
Heart rate (HR) (beats/min)

High frequency component (HF)

Low frequency component (LF)

<u>Haemodynamics</u>
Systolic Blood Pressure (SBP) (mm Hg)

Diastolic Blood Pressure (DBP) (mm Hg)

Mean Arterial Pressure (MAP) (mm Hg)</td>
<td><u>Heart rate variability after use</u>
<table>
<tr><th></th><th>ENDS vs. Sham</th><th>ENDS vs. ENNDS</th><th>ENNDS vs. Sham</th></tr>
<tr><td>Δ HR</td><td>Increase (p=0.01)</td><td>Increase (p=0.05)</td><td>No difference (p=0.54)</td></tr>
<tr><td>Δ HF, nu</td><td>Decrease (p=0.02)</td><td>Decrease (p=0.03)</td><td>No difference (p=0.9)</td></tr>
<tr><td>Δ LF, nu</td><td>Increase (p=0.003)</td><td>No difference (p=0.08)</td><td>No difference (p=0.17)</td></tr>
<tr><td>Δ LF/HF</td><td>Increase (p=0.02)</td><td>No difference (p=0.06)</td><td>No difference (p=0.6)</td></tr>
</table>
<u>Acute changes in haemodynamics (mean (SEM))</u>
<table>
<tr><th></th><th>Δ SBP</th><th>Δ DBP</th><th>Δ MAP</th></tr>
<tr><td>ENDS</td><td>1.2 (2.0)</td><td>1.3 (1.1)</td><td>1.3 (1.2)</td></tr>
<tr><td>ENNDS</td><td>-0.8 (1.9)</td><td>-1.0 (1.1)</td><td>-1.0 (1.2)</td></tr>
<tr><td>Sham</td><td>-1.7 (2.0)</td><td>-1.1 (1.1)</td><td>-0.8 (1.2)</td></tr>
<tr><td>P</td><td>0.59</td><td>0.23</td><td>0.37</td></tr>
</table></td>
<td>Moderate methodological quality

Very small study size

<u>Conflicts of interest</u>
None declared

<u>Funding</u>
Supported by the Tobacco-Related Disease Research Program, American Heart Association, the National Institute of Environmental Health Sciences, National Institutes of Health, and the UCLA Clinical and Translational Science Institute.</td>
</tr>
<tr><td colspan="6">Cohort studies</td></tr>
<tr>
<td>Polosa et al., 2017

Italy

Prospective cohort study

2013-2017

Online survey of regular vape shop customers</td>
<td><u>Study size</u>
31 never smoker regular vape shop customers enrolled, 21 included in analysis

<u>Sample</u>
Never smokers or <100 cigarettes smoked in lifetime, daily e-cigarette users for ≥3 months

<u>Gender (%)</u>
Male: 21/31 (68%)
Female: 10/31 (32%)

<u>Age - mean (SD) years</u>
ENDS: 29.7 (6.1)
Control: 32.5 (7.0)</td>
<td><u>Exposure (n=9)</u>
Daily e-liquid consumption - median (range): 4mL (2-5)

<u>Comparator (n=12)</u>
Non-smoker and non-e-cigarette user

<u>Materials - device type</u>
Advanced refillable: 44%
Standard refillable: 56%

<u>Materials - nicotine concentration</u>
0%: 33%
0.9%: 22%
1.2%: 22%
1.6%: 11%
1.8%: 11%

<u>Follow-up</u>
Follow-up at 12, 24 and 42 months</td>
<td>Systolic blood pressure (mm Hg)

Diastolic blood pressure (mm Hg)

Heart rate (beats/min)</td>
<td><u>Systolic blood pressure - Mean (SD)</u>
<table>
<tr><th></th><th>Baseline</th><th>12 months</th><th>24 months</th><th>42 months</th></tr>
<tr><td>E-cigarette</td><td>115 (9)</td><td>116 (5)</td><td>114 (9)</td><td>118 (10)</td></tr>
<tr><td>Control</td><td>117 (9)</td><td>117 (10)</td><td>116 (10)</td><td>116 (9)</td></tr>
<tr><td>P</td><td colspan="4">0.82</td></tr>
</table>
<u>Diastolic blood pressure - Mean (SD)</u>
<table>
<tr><th></th><th>Baseline</th><th>12 months</th><th>24 months</th><th>42 months</th></tr>
<tr><td>E-cigarette</td><td>79 (6)</td><td>78 (4)</td><td>73 (9)</td><td>76 (8)</td></tr>
<tr><td>Control</td><td>74 (9)</td><td>76 (6)</td><td>75 (9)</td><td>73 (9)</td></tr>
<tr><td>P</td><td colspan="4">0.50</td></tr>
</table>
<u>Heart rate - Mean (SD)</u>
<table>
<tr><th></th><th>Baseline</th><th>12 months</th><th>24 months</th><th>42 months</th></tr>
<tr><td>E-cigarette</td><td>72 (7)</td><td>71 (9)</td><td>71 (9)</td><td>71 (7)</td></tr>
<tr><td>Control</td><td>79 (9)</td><td>78 (8)</td><td>76 (8)</td><td>78 (9)</td></tr>
<tr><td>P</td><td colspan="4">0.15</td></tr>
</table>
P: E-cigarette vs. control</td>
<td>Moderate methodological quality

Very small study size

<u>Conflicts of interest</u>
Grants and consulting/speaking fees from pharmaceutical companies, and electronic cigarette industry and trade associations

<u>Funding</u>
Supported by Catania University</td>
</tr>
<tr><td colspan="6">Non-randomised intervention studies</td></tr>
</table>

<table>
<tr><th>Study details (author, year, location, study type time frame, [data source])</th><th>Sample characteristics</th><th>Intervention/exposure and comparator</th><th>Outcome measure</th><th>Results</th><th>Quality assessment, study size conflicts of interest, funding</th></tr>
<tr>
<td>Pywell et al., 2018

UK

Non-randomised before-and-after pilot crossover study

Study date not reported</td>
<td><u>Study size</u>
15 participants

<u>Participants</u>
Smokers (n=7): average cigarette consumption 1.5 packs per week.
Non-smokers (n=8)

<u>Gender</u>
Not reported

<u>Age - mean (range) years</u>
26 (25-27)</td>
<td><u>Intervention 1</u>
ENDS: 24mg nicotine

<u>Intervention 2</u>
ENNDS: 0mg nicotine

<u>Comparator</u>
Before session

<u>Materials - device type</u>
Not specified

<u>Pattern of exposure</u>
Baseline (5 mins), ENNDS one puff every 30 secs for 10 inhalations. Same protocol for ENDS</td>
<td>Hand microcirculation (superficial and deep)</td>
<td><u>Superficial blood flow - average % change in blood flow (SE)</u>
<table>
<tr><th></th><th>During</th><th>0-5</th><th>5-10</th><th>10-15</th><th>15-20</th></tr>
<tr><td>Non-smokers</td><td></td><td></td><td></td><td></td><td></td></tr>
<tr><td>ENNDS</td><td>-11.37% (16.28)</td><td>-4.76% (16.68)</td><td>-8.24% (16.92)</td><td>-11.47% (17.56)</td><td>-16.93% (23.60)</td></tr>
<tr><td>P</td><td>0.74</td><td>0.86</td><td>0.83</td><td>0.74</td><td>0.74</td></tr>
<tr><td>ENDS</td><td>-23.12% (16.28)</td><td>-3.05% (16.68)</td><td>7.42% (16.92)</td><td>-2.71% (17.56)</td><td>20.37% (23.63)</td></tr>
<tr><td>P</td><td>0.32</td><td>0.88</td><td>0.83</td><td>0.88</td><td>0.71</td></tr>
<tr><td>Smokers</td><td></td><td></td><td></td><td></td><td></td></tr>
<tr><td>ENNDS</td><td>37.15% (11.18)</td><td>56.07% (11.86)</td><td>49.81% (13.32)</td><td>39.27% (14.73)</td><td>69.70% (16.98)</td></tr>
<tr><td>P</td><td><0.05</td><td><0.05</td><td><0.05</td><td><0.05</td><td><0.05</td></tr>
<tr><td>ENDS</td><td>-4.27% (14.90)</td><td>-52.99% (16.79)</td><td>-66.37% (14.97)</td><td>-76.92% (13.74)</td><td>-4.73% (21.50)</td></tr>
<tr><td>P</td><td>0.86</td><td>< 0.05</td><td>< 0.05</td><td>< 0.05</td><td>0.09</td></tr>
</table>
<u>Deep blood flow - average % change in blood flow (SE)</u>
<table>
<tr><th></th><th>During</th><th>0-5</th><th>5-10</th><th>10-15</th><th>15-20</th></tr>
<tr><td>Non-smokers</td><td></td><td></td><td></td><td></td><td></td></tr>
<tr><td>ENNDS</td><td>1.98% (5.94)</td><td>-7.26% (6.31)</td><td>-8.46% (6.18)</td><td>-7.46% (6.82)</td><td>-0.21% (6.66)</td></tr>
<tr><td>P</td><td>0.82</td><td>0.61</td><td>0.58</td><td>0.61</td><td>0.97</td></tr>
<tr><td>ENDS</td><td>-4.73% (5.94)</td><td>-7.25% (6.31)</td><td>-3.64% (6.18)</td><td>-6.26% (6.82)</td><td>-1.84% (6.67)</td></tr>
<tr><td>P</td><td>0.75</td><td>0.61</td><td>0.75</td><td>0.72</td><td>0.82</td></tr>
<tr><td>Smokers</td><td></td><td></td><td></td><td></td><td></td></tr>
<tr><td>ENNDS</td><td>-3.42% (6.00)</td><td>3.02% (6.29)</td><td>2.88% (6.08)</td><td>3.33% (6.67)</td><td>3.86% (6.68)</td></tr>
<tr><td>P</td><td>0.75</td><td>0.75</td><td>0.75</td><td>0.75</td><td>0.75</td></tr>
<tr><td>ENDS</td><td>-19.31% (6.13)</td><td>-26.68% (6.05)</td><td>-27.83% (5.79)</td><td>-28.43% (6.51)</td><td>-24.01% (6.43)</td></tr>
<tr><td>P</td><td><0.05</td><td><0.05</td><td><0.05</td><td><0.05</td><td><0.05</td></tr>
</table>
P: value compared to baseline</td>
<td>High methodological quality

Very small study size

<u>Conflicts of interest</u>
Not reported

<u>Funding</u>
Not reported</td>
</tr>
</table>

Percentages and p-values are presented as reported in original studies.

Alx75 = augmentation index corrected for heart rate; CPD = cigarette(s) per day; DBP = diastolic blood pressure; ENDS = electronic nicotine delivery system; ENNDS = electronic non-nicotine delivery system; HF = high frequency; HR = heart rate; LF = low frequency; MAP = mean arterial pressure; max = maximum; NIH = National Institutes of Health; PWA = pulse wave amplitude; PWV = pulse wave velocity; RHI = reactive hyperaemia index; SBP = systolic blood pressure; SD = standard deviation; SE = standard error; SEM = standard error of the mean; SEVR = subendocardial viability ratio; UCLA = University of California, Los Angeles; UK = United Kingdom; US = United States.

Table 2.3. Study details: cardiovascular health outcomes – case reports

Study details (author, year, location, data source)	Demographics and medical history	Exposure	Presentation	Outcome	Quality assessment
Shea et al., 2020<br><br>US<br><br>Hospital record	Male<br><br>48 years<br><br>Medical history<br>History of cardiac sarcoidosis and symptomatic non-sustained ventricular tachycardia, underwent implantation of a primary-prevention implantable cardioverter-defibrillator (ICD), later upgraded to a dual-chamber ICD	E-cigarette (JUUL device with a magnetic USB charging dock) was frequently stored in his left breast pocket overlying the device	Reported "beep" several times from device. The JUUL device was held up to his ICD, which elicited the steady magnet tone<br><br>There were no symptoms associated with these episodes and the patient denied any clinical ICD shock. There had been no recent reprogramming of his device. A remote transmission demonstrated normal device function without any alert notifications	Educated about the importance of keeping any type of magnet at least 6 inches from the device	Moderate methodological quality<br><br>Conflicts of interest<br>Educational and research funding from medical device manufacturers<br><br>Funding<br>No specific funding

Percentages and p-values are presented as reported in original studies.
ICD = implantable cardioverter-defibrillator; US = United States; USB = universal serial bus.

3. Respiratory disease

Table 3.1. Study details: respiratory health outcomes – randomised controlled trials, cohort studies and non-randomised intervention studies

Study details (author, year location, study type, time frame, [data source])	Sample characteristics	Intervention and control	Outcome measure	Results	Quality assessment, study size, conflict of interest, funding
Randomised controlled trials					
**Antoniewicz et al., 2019**<br><br>Sweden<br><br>Randomised, double-blinded, crossover study<br><br>Study date not reported<br><br>Laboratory study	<u>Study size</u><br>15 participants<br><br><u>Sample</u><br>Occasional users of tobacco products (max 10 cigarettes/month), healthy<br><br><u>Gender - n (%)</u><br>Male: 6/15 (40%)<br>Female: 9/15 (60%)<br><br><u>Age - mean (SD) years</u><br>26 (3)	<u>Intervention 1</u><br>ENDS: 19mg/mL nicotine<br><br><u>Intervention 2</u><br>ENNDS: 0mg/mL nicotine<br><br><u>Comparator</u><br>Before session<br><br><u>Materials</u><br>Variable mod third generation e-cigarette with e-liquid base primarily 49.4% propylene glycol, 44.4% vegetable glycerin, 5% ethanol, without any added flavourings<br><br><u>Pattern of exposure</u><br>30 puffs from ENDS for 30 min, each puff lasting approximately three seconds; measurements up to 6h following exposure	<u>Impulse oscillometry</u><br>Flow resistance at 5Hz/11Hz/13Hz/17Hz/19 Hz (R5/11/13/17/19)<br><br>Reactance at 5Hz (X5)<br><br>Difference of R5Hz and R19Hz (R5-19Hz)<br><br><u>Spirometry</u><br>Reactance area (AX)<br><br>Resonance frequency (fres)<br><br>Vital capacity (VC)<br><br>Forced expiratory volume in one second (FEV_1)<br><br><u>Fractional exhaled nitric oxide (FeNO)</u>	See results tables below	Moderate methodological quality<br><br>Very small study size<br><br><u>Conflicts of interest</u><br>None declared<br><br><u>Funding</u><br>Supported by the Swedish Heart and Lung Association, the Swedish Society of Medicine, the Swedish Heart-Lung Foundation and Stockholm County Council

<u>Impulse oscillometry</u>

	Baseline	0.5h	2h	4h	6h
R5 Hz - p_{time} = 0.001; $p_{time\ x\ exposure}$ = 0.003					
ENDS	3.57 (0.73)	3.85 (0.93)	3.27 (0.88)	3.24 (0.66)	3.32 (0.80)
ENNDS	3.41 (0.75)	3.26 (0.70)	3.15 (0.64)	3.30 (0.73)	3.23 (0.72)
R11 Hz - p_{time} = 0.002; $p_{time\ x\ exposure}$ < 0.001					
ENDS	3.19 (0.55	3.52 (0.74*)	3.02 (0.72)	2.96 (0.54)	3.05 (0.67)
ENNDS	3.09 (0.67	2.95 (0.61)	2.92 (0.51)	3.02 (0.65)	2.95 (0.63)
R13 Hz - p_{time} = 0.002; $p_{time\ x\ exposure}$ = 0.003					
ENDS	3.18 (0.55)	3.51 (0.77*)	3.03 (0.70)	2.96 (0.53)	3.03 (0.64)
ENNDS	3.07 (0.67)	2.94 (0.60)	2.92 (0.53)	3.01 (0.65)	2.94 (0.64)
R17 Hz - p_{time} = 0.002; $p_{time\ x\ exposure}$ = 0.010					
ENDS	3.18 (0.55)	3.48 (0.75*)	3.03 (0.66)	2.96 (0.53)	3.03 (0.61)
ENNDS	3.05 (0.68)	2.97 (0.61)	2.91 (0.57)	3.00 (0.69)	2.95 (0.65)
R19 Hz - p_{time} = 0.004; $p_{time\ x\ exposure}$ = 0.002					
ENDS	3.23 (0.55)	3.55 (0.74*)	3.13 (0.67)	3.04 (0.56)	3.10 (0.61)
ENNDS	3.09 (0.69)	3.04 (0.64)	2.94 (0.58)	3.06 (0.71)	3.05 (0.68)
X5 Hz - p_{time} = 0.057; $p_{time\ x\ exposure}$ = 0890					
ENDS	– 0.91 (0.29)	– 0.85 (0.28)	– 0.83 (0.31)	– 0.81 (0.30)	– 0.82 (0.35)
ENNDS	– 0.92 (0.32)	– 0.85 (0.30)	– 0.81 (0.33	– 0.82 (0.3)	– 0.81 (0.28)
R5-R19 Hz - p_{time} = 0.058; $p_{time\ x\ exposure}$ = 0.314					
ENDS	0.34 (0.42)	0.30 (0.43)	0.14 (0.34)	0.20 (0.49)	0.22 (0.35)
ENNDS	0.32 (0.41)	0.22 (0.29)	0.22 (0.37)	0.24 (0.47)	0.18 (0.26)

<u>Spirometry</u>

	Baseline	0.5h	2h	4h	6h
AX - p_{time} = 0.155; $p_{time\ x\ exposure}$ = 0.281					
ENDS	3.48 (2.41)	3.27 (2.15)	2.70 (2.19)	2.87 (2.56)	3.02 (2.40)
ENNDS	3.64 (2.64)	3.03 (1.67)	2.90 (1.89)	4.27 (3.85)	2.57 (1.37)
Fres - p_{time} = 0.018; $p_{time\ x\ exposure}$ = 0.042					
ENDS	12.28 (3.97)	12.06 (3.18)	10.86 (2.57)	11.20 (3.19)	11.73 (3.36)
ENNDS	12.44 (3.66)	11.70 (2.70)	11.54 (2.99)	11.92 (3.35)	11.06 (2.19*)
VC - p_{time} = 0.020; $p_{time\ x\ exposure}$ = 0.636					
ENDS	5.01 (1.23)	4.92 (1.18†)	4.94 (1.22†)	4.96 (1.18)	4.96 (1.19)
ENNDS	5.02 (1.21)	4.98 (1.21†)	4.96 (1.20†)	5.00 (1.20)	4.97 (1.20)
FEV_1 - p_{time} = 0.0096; $p_{time\ x\ exposure}$ = 0.788					
ENDS	3.82 (0.76)	3.84 (0.79)	3.86 (0.82)	3.85 (0.81)	3.87 (0.80)
ENNDS	3.86 (0.76)	3.86 (0.78)	3.90 (0.77)	3.90 (0.77)	3.89 (0.80)

<u>Fractional exhaled nitric oxide</u>

	Baseline	0.5h	2h	4h	6h
FeNO - p_{time} = 0.00; $p_{time\ x\ exposure}$ = 0.002					
ENDS	12.36 (2.87)	12.00 (3.55)	13.91 (3.21†)	13.09 (3.36)	11.36 (2.98)
ENNDS	11.82 (3.87)	12.91 (4.04	12.91 (4.01†)	12.18 (3.25)	11.27 (3.77)

<table>
<tr><th>Study details (author, year location, study type, time frame, [data source])</th><th>Sample characteristics</th><th>Intervention and control</th><th>Outcome measure</th><th>Results</th><th>Quality assessment, study size, conflict of interest, funding</th></tr>
<tr><td></td><td></td><td></td><td></td><td>*Denotes significant change from baseline due to exposure (contrast for time × exposure)
†Denotes significant change from baseline, not influenced by exposure (contrast for time)</td><td></td></tr>
<tr><td>Kerr et al., 2019

UK

Single-centre, prospective, randomised crossover study

June-December 2016

Laboratory study</td><td><u>Study size</u>
20 participants

<u>Sample</u>
Habitual tobacco smokers of one or more CPD

<u>Gender - n (%)</u>
Male: 20/20 (100%)
Female: 0/20 (0%)

<u>Age - mean (SD) years</u>
31.6 (10.5)</td><td><u>Intervention 1</u>
ENDS: 18mg/mL nicotine, tobacco flavoured

<u>Intervention 2</u>
Conventional cigarette

<u>Comparator</u>
Before session

<u>Materials</u>
ENDS: SmokeMax, second generation; variable voltage rechargeable
Conventional cigarette: own type

<u>Pattern of exposure</u>
15 puffs</td><td><u>Spirometry</u>
Forced expiratory volume in one second (FEV_1) (l)

Forced vital capacity (FVC) (l)

FEV_1/FVC: Tiffeneau-Pinelli index (%)

Peak expiratory flow (PEF) (l/min)

<u>Exhaled breath</u>
Carbon monoxide (CO) (ppm)</td><td><u>Spirometry and exhaled breath</u>
<table>
<tr><th></th><th>Pre</th><th>Post</th><th>Change</th><th>P</th></tr>
<tr><td>FEV_1</td><td></td><td></td><td></td><td></td></tr>
<tr><td>ENDS</td><td>4.2 (0.6)</td><td>4.1 (0.7)</td><td>-0.1 (0.2)</td><td>0.132(a)</td></tr>
<tr><td>Cigarette</td><td>4.3 (0.7)</td><td>4.2 (0.6)</td><td>0.0 (0.2)</td><td>0.373(a)</td></tr>
<tr><td>FVC</td><td></td><td></td><td></td><td></td></tr>
<tr><td>ENDS</td><td>5.2 (0.7)</td><td>5.1 (0.7)</td><td>-0.1 (0.3)</td><td>0.433(b)</td></tr>
<tr><td>Cigarette</td><td>5.3 (0.9)</td><td>5.2 (0.8)</td><td>0.0 (0.3)</td><td>0.723(b)</td></tr>
<tr><td>FEV_1/FVC</td><td></td><td></td><td></td><td></td></tr>
<tr><td>ENDS</td><td>81.1% (6.8)</td><td>80.9% (7.3)</td><td>-0.2% (2.0)</td><td>0.629(b)</td></tr>
<tr><td>Cigarette</td><td>81.3% (7.0)</td><td>81.0% (7.2)</td><td>-0.3% (4.8)</td><td>0.501(b)</td></tr>
<tr><td>PEF</td><td></td><td></td><td></td><td></td></tr>
<tr><td>ENDS</td><td>562 (62)</td><td>531 (96)</td><td>-31 (54)</td><td>0.019(a)</td></tr>
<tr><td>Cigarette</td><td>567 (72)</td><td>545 (81)</td><td>-22 (53)</td><td>0.074(a)</td></tr>
<tr><td>CO</td><td></td><td></td><td></td><td></td></tr>
<tr><td>ENDS</td><td>9 (10)</td><td>7 (7)</td><td>-2 (3)</td><td>0.007(b)</td></tr>
<tr><td>Cigarette</td><td>9 (10)</td><td>20 (10)</td><td>11 (2)</td><td><0.001(b)</td></tr>
</table>
P derived from:
a) paired t-test
b) related-samples Wilcoxon signed ranked test</td><td>Moderate methodological quality

Very small study size

<u>Conflicts of interest</u>
None declared

<u>Funding</u>
Authors supported by British Heart Foundation Centre of Research Excellence</td></tr>
</table>

<table>
<tr><th>Study details (author, year location, study type, time frame, [data source])</th><th>Sample characteristics</th><th>Intervention and control</th><th>Outcome measure</th><th>Results</th><th>Quality assessment, study size, conflict of interest, funding</th></tr>
<tr>
<td>Chaumont et al., 2019

Belgium

Randomised, single-blinded, crossover study

2017

Laboratory study</td>
<td><u>Study size</u>
25 participants in whole study. 9 in pulmonary testing

<u>Sample</u>
Healthy occasional tobacco smokers (not smoke >20 combustible cigarettes per week)

<u>Gender</u>
Not reported for subset of 9

<u>Age - mean (SD) years</u>
Not reported for subset of 9</td>
<td><u>Intervention 1</u>
ENNDS session

<u>Intervention 2</u>
Sham 3mg/mL ENDS control session (device turned off)

<u>Comparator</u>
Before session

<u>Materials</u>
Fourth-generation ENNDS (50:50 PG/GLY, Alien 220 box mod, TFV8 baby beast tank)

<u>Pattern of exposure</u>
25 puffs - one every 30s (inhale for 4s, hold for 4s, exhale). Each session separated by minimum 1 week washout. Measurements within 5-10 minutes of exposure</td>
<td><u>Spirometry</u>
Forced expiratory volume in one second (FEV_1) (l)

FEV_1/FVC: Tiffeneau-Pinelli index (%)

Peak expiratory flow (PEF) (l/s)

Forced expiratory flow (FEF) at 75%, 50%, 25% and 25-75% of FVC (l/s)

Airway total resistance (ATR) (cm H_2O l^{-1} s^{-1})

Intrathoracic gas volume (IGV) (l)

Total lung capacity (TLC) (l)

Residual volume (RV) (l)

Residual volume/total lung capacity (RV/TLC) (%)

Diffusion capacity of carbon monoxide (DL_{CO}) (mL min^{-1} $mmHg^{-1}$)</td>
<td><u>Spirometry</u>
<table>
<tr><th></th><th></th><th>Sham Vaping</th><th>P</th><th>ENNDS</th><th>P</th></tr>
<tr><td>FEV_1</td><td>Before
After</td><td>4.5 (4-4.6)
4.2 (4-4.6)</td><td>0.592</td><td>4.4 (4.2-4.6)
4.3 (3.9-4.6)</td><td>0.021</td></tr>
<tr><td>FEV_1/FVC</td><td>Before
After</td><td>82.2% (77.5-84.1)
82% (77.7-84.8)</td><td>0.79</td><td>83.5% (76.3-85.7)
81% (74-82.6)</td><td>0.002</td></tr>
<tr><td>PEF</td><td>Before
After</td><td>7.8 (7.4-9.8)
9.2 (7.4-9.9)</td><td>0.538</td><td>8.5 (7.2-9.3)
7.85 (7-9.8)</td><td>0.633</td></tr>
<tr><td>$FEF_{75\%}$</td><td>Before
After</td><td>6.9 (6.1-8.6)
7.1 (5.5-8.8)</td><td>0.522</td><td>7.2 (6.1-8.8)
6.9 (5.9-8.2)</td><td>0.112</td></tr>
<tr><td>$FEF_{50\%}$</td><td>Before
After</td><td>5 (3.6-5.4)
4.8 (3.6-5.1)</td><td>0.588</td><td>4.8 (4-6.1
4.2 (3.7-5.5)</td><td>0.009</td></tr>
<tr><td>$FEF_{25\%}$</td><td>Before
After</td><td>2.2 (1.5-2.5)
2.1 (1.6-2.5)</td><td>0.764</td><td>2.5 (1.7-2.6)
2 (1.4-2.3)</td><td>0.002</td></tr>
<tr><td>$FEF_{25-75\%}$</td><td>Before
After</td><td>4.5 (3.1-4.7)
4.2 (3.1-4.6)</td><td>0.545</td><td>4.2 (3.5-5.4)
3.7 (3.1-4.9)</td><td>0.003</td></tr>
<tr><td>ATR</td><td>Before
After</td><td>3.75 (3.2-5)
3.9 (3.4-4.5)</td><td>0.661</td><td>4 (3.35-4.5)
4.5 (3.8-5.9)</td><td>0.089</td></tr>
<tr><td>IGV</td><td>Before
After</td><td>3.2 (2.9-4)
3.5 (3-3.8)</td><td>0.943</td><td>3.5 (2.7-4)
3.1 (2.7-3.7)</td><td>0.486</td></tr>
<tr><td>TLC</td><td>Before
After</td><td>6.9 (6.2-8)
6.9 (6.2-8)</td><td>0.649</td><td>6.7 (6.2-7.9)
6.6 (5.9-7.7)</td><td>0.517</td></tr>
<tr><td>RV</td><td>Before
After</td><td>1.5 (1.1-2.4)
1.8 (1.6-2.25)</td><td>0.57</td><td>1.4 (1.2-2.5)
1.5 (1.2-2.2)</td><td>0.59</td></tr>
<tr><td>RV/TLC</td><td>Before
After</td><td>26% (19-30)
27% (23.5-29.5)</td><td>0.452</td><td>21% (19.5-31)
23% (19.5-28)</td><td>0.657</td></tr>
<tr><td>DL_{CO}</td><td>Before
After</td><td>32.65 (28.4-38.3)
32.1(26.1-37.7)</td><td>0.401</td><td>34.1 (23.4-41)
30.7 (26.6-43.1)</td><td>0.398</td></tr>
</table>
Values are medians (interquartile ranges)</td>
<td>Moderate methodological quality

Very small study size

<u>Conflicts of interest</u>
None declared

<u>Funding</u>
Supported by the “Fonds Erasme pour la Recherche Médicale”; “Fondation pour la Chirurgie Cardiaque”; “Fondation Emile Saucez-René Van Poucke”; “Prix Docteur & Mrs Rene Tagnon”; “Fondation IRIS”; the “Prix de l’Association André Vésale”; Astra Zeneca; “Fonds Fruit de Deux Vies’; “Fond David and Alice Van Buuren”</td>
</tr>
</table>

<table>
<tr><th>Study details (author, year location, study type, time frame, [data source])</th><th>Sample characteristics</th><th>Intervention and control</th><th>Outcome measure</th><th>Results</th><th>Quality assessment, study size, conflict of interest, funding</th></tr>
<tr>
<td>Staudt et al., 2018

US

Randomised (unequal), before-and-after study

Study date not reported

Weill Cornell Medical College Clinical Translational and Science Center and the Department of Genetic Medicine Clinical Research Facility</td>
<td><u>Study size</u>
10 participants

<u>Sample</u>
Never smokers, self-reported history and confirmed by absence of tobacco metabolites in urine

<u>Gender - n (%)</u>
Male: 5/10 (50%)
Female: 5/10 (50%)

<u>Age - mean (SD) years</u>
40.2 (9.7)</td>
<td><u>Intervention 1 (n=7)</u>
ENDS: nicotine concentration unknown

<u>Intervention 2 (n=3)</u>
ENNDS

<u>Comparator</u>
Before session

<u>Materials</u>
Blu branded ENDS and ENNDS

<u>Pattern of exposure</u>
10 puffs, 30 minutes rest, 10 puffs. Assessed 1 week after session</td>
<td><u>Spirometry</u>
Forced vital capacity (FVC)

Forced expiratory volume in one second (FEV_1)

FEV_1/FVC: Tiffeneau-Pinelli index

Total lung capacity (TLC)

Diffusion capacity for carbon monoxide (DL_{CO})

O_2 saturation</td>
<td><u>Spirometry</u>
<table>
<tr><th></th><th>ENDS</th><th></th><th>ENNDS</th><th></th></tr>
<tr><th></th><th>Baseline</th><th>Post</th><th>Baseline</th><th>Post</th></tr>
<tr><td>FVC (% predicted)</td><td>112 (16)</td><td>112 (11)</td><td>105 (6)</td><td>98.3 (12)</td></tr>
<tr><td>FEV_1 (% predicted)</td><td>112 (15)</td><td>113 (11)</td><td>103 (9)</td><td>91 (8)</td></tr>
<tr><td>FEV_1/FVC (% observed)</td><td>81 (3)</td><td>83 (3)</td><td>81 (4)</td><td>76 (4)</td></tr>
<tr><td>TLC (% predicted)</td><td>91 (11)</td><td>92 (7)</td><td>94 (13)</td><td>91 (21)</td></tr>
<tr><td>DL_{CO} (% predicted)</td><td>88 (10)</td><td>85 (13)</td><td>92 (9)</td><td>87 (3)</td></tr>
<tr><td>O_2 saturation</td><td>99 (1)</td><td>99 (1)</td><td>99 (2)</td><td>98 (1)</td></tr>
</table></td>
<td>Moderate methodological quality

Very small study size

<u>Conflicts of interest</u>
None declared

<u>Funding</u>
Supported by NIH and the Family Smoking Prevention and Tobacco Control Act</td>
</tr>
<tr><td colspan="6">Cohort studies</td></tr>
<tr>
<td>Bhatta & Glantz, 2020

US

Nationally representative longitudinal study

2013-2016

PATH (Wave 1, 2 and 3)</td>
<td><u>Study size</u>
32,320 participants at baseline

<u>Sample</u>
Current: ever used/smoked (fairly regularly) every day or some days
Former: ever used/smoked, but do not currently use/smoke
Never: never used/smoked

<u>Gender (baseline) (%)</u>
Male: 48.1%
Female: 51.9%

<u>Age - mean (SD) at baseline (years)</u>
18–24: 13.1%
25–34: 17.7%
35–44: 16.5%
45–54: 17.9%
55–64: 16.6%
65–74: 11.1%
≥75: 7.1%</td>
<td><u>Exposure 1 - e-cigarette</u>
Current or former

<u>Exposure 2 - smoker</u>
Current or former

Note: e-cigarette and cigarette use were not exclusive, dual users are included in both populations

<u>Comparator 1 - e-cigarette</u>
Never e-cigarette or smoker

<u>Materials - device type</u>
Not reported

<u>Materials - nicotine concentration</u>
Not reported

<u>Follow-up</u>
1 and 2 years after baseline</td>
<td><u>Self-reported lung or respiratory disease</u>
Chronic obstructive pulmonary disease (COPD), chronic bronchitis, emphysema, asthma</td>
<td><u>Incident respiratory disease at wave 2 or 3 excluding people with respiratory disease at wave 1</u>
<table>
<tr><th></th><th>ENDS</th><th></th><th>Smoker</th><th></th></tr>
<tr><th></th><th>AOR (95% CI)</th><th>P</th><th>AOR (95% CI)</th><th>P</th></tr>
<tr><td>Former</td><td>1.31 (1.07-1.60)</td><td>0.009</td><td>1.16 (0.87-1.57)</td><td>0.315</td></tr>
<tr><td>Current</td><td>1.29 (1.03-1.61)</td><td>0.026</td><td>2.56 (1.92-3.41)</td><td><0.001</td></tr>
</table>
<u>Incident respiratory disease at wave 2 or 3 excluding people with respiratory disease at wave 1</u>
<table>
<tr><th></th><th>ENDS</th><th></th><th>Smoker</th><th></th></tr>
<tr><th></th><th>AOR (95% CI)</th><th>P</th><th>AOR (95% CI)</th><th>P</th></tr>
<tr><td>COPD</td><td></td><td></td><td></td><td></td></tr>
<tr><td>Former</td><td>1.82 (1.23-2.69)</td><td>0.004</td><td>1.47 (0.42-5.20)</td><td>0.550</td></tr>
<tr><td>Current</td><td>1.44 (0.79-2.62)</td><td>0.237</td><td>5.79 (1.64-20.44)</td><td>0.008</td></tr>
<tr><td>Chronic bronchitis</td><td></td><td></td><td></td><td></td></tr>
<tr><td>Former</td><td>1.43 (1.02-2.00)</td><td>0.039</td><td>0.95 (0.56-1.59)</td><td>0.844</td></tr>
<tr><td>Current</td><td>1.60 (1.13-2.27)</td><td>0.010</td><td>1.96 (1.23-3.12)</td><td>0.005</td></tr>
<tr><td>Emphysema</td><td></td><td></td><td></td><td></td></tr>
<tr><td>Former</td><td>1.40 (0.9-2.83)</td><td>0.348</td><td>0.85 (0.21-3.42)</td><td>0.831</td></tr>
<tr><td>Current</td><td>1.60 (0.75-3.44)</td><td>0.229</td><td>3.66 (0.98-13.60)</td><td>0.056</td></tr>
<tr><td>Asthma</td><td></td><td></td><td></td><td></td></tr>
<tr><td>Former</td><td>1.23 (0.90-1.69)</td><td>0.200</td><td>0.87 (0.53-1.42)</td><td>0.575</td></tr>
<tr><td>Current</td><td>1.56 (1.10-2.22)</td><td>0.015</td><td>1.57 (1.02-2.42)</td><td>0.046</td></tr>
</table>
Referent: never users/smokers
Controlled for combustible tobacco smoking (former and current), age, BMI, sex, poverty level, race/ethnicity, and clinical variables at Wave 1</td>
<td>Moderate methodological quality

Large study size

<u>Conflicts of interest</u>
None declared

<u>Funding</u>
None</td>
</tr>
</table>

<table>
<tr><th>Study details (author, year location, study type, time frame, [data source])</th><th>Sample characteristics</th><th>Intervention and control</th><th>Outcome measure</th><th>Results</th><th>Quality assessment, study size, conflict of interest, funding</th></tr>
<tr>
<td>Bowler et al., 2017

US

Prospective cohort study

2011-2016

Two longitudinal studies: COPDGene and SPIROMICS</td>
<td><u>Study size</u>
4595 participants;
COPDGene: 3,535
SPIROMICS: 1,060

<u>Sample</u>
Adults (45-80 years) who are current or former smokers

<u>Gender - male (%)</u>
COPDGene
Never: 51%
Current: 41%
Former: 43%
SPIROMICS
Never: 54%
Current: 55%
Former: 44%

<u>Age range (years)</u>
45-80</td>
<td><u>Exposure</u>
Ever ENDS use

<u>Comparator</u>
Non-users

<u>Materials - device type</u>
No details

<u>Materials - nicotine concentration</u>
No details

<u>Follow-up</u>
5 years</td>
<td><u>COPD exacerbations</u>

<u>COPD progression</u> (GOLD criteria)

<u>Lung function</u> (spirometry)

<u>Adverse COPD outcomes</u></td>
<td><u>COPD exacerbations</u>
History of ever using e-cigarettes was significantly predictive of COPD exacerbations in COPDGene (p=0.01) after adjustment. SPIROMICS: ever using e-cigarettes was associated with reported exacerbations in the year prior to enrolment (p=0.04).

<u>COPD progression</u>
COPDGene: ever e-cigarette users were more likely to have progression of lung disease (defined by worsening of GOLD stage) after 5 years (p<0.001) than never users. Non-significant after adjustment.

<u>Lung function</u>
COPDGene: ever e-cigarette users were more likely to have a more rapid decline in lung function (FEV$_1$) than never users (43mL/year vs. 34mL/year; p=0.003). Non-significant after adjustment.

<u>Adverse COPD outcomes</u>
Ever using e-cigarettes was associated with 8% (SD 2) increased prevalence of chronic bronchitis, after adjustment (p<0.001).</td>
<td>Moderate methodological quality

Large study size

<u>Conflicts of interest</u>
None declared

<u>Funding</u>
SPIROMICS: supported by contracts from the NIH/NHLBI, supplemented by Foundation for the NIH
COPDGene: supported by National Heart, Lung, and Blood Institute and COPD Foundation
Both contributions from pharmaceutical companies</td>
</tr>
<tr>
<td>Polosa et al., 2017

Italy

Prospective cohort study

2013-2017

Online survey, regular vape shop customers</td>
<td><u>Study size</u>
31 never smokers enrolled, 21 included in analysis

<u>Sample</u>
Never smokers or <100 cigarettes smoked in lifetime, daily e-cigarette users for ≥3 months

<u>Gender - n (%)</u>
Male: 21/31 (68%)
Female: 10/31 (32%)

<u>Age - mean (SD) years</u>
ENDS: 29.7 (6.1)
Control: 32.5 (7.0)</td>
<td><u>Exposure (n=9)</u>
Daily e-liquid consumption - median (range): 4mL (2-5)

<u>Comparator (n=12)</u>
Non-smoker and non-e-cigarette user

<u>Materials - device type</u>
Advanced refillable: 44%
Standard refillable: 56%

<u>Materials - nicotine concentration (%)</u>
0%: 33
0.9%: 22
1.2%: 22
1.6%: 11
1.8%: 11

<u>Follow-up</u>
Follow-up at 12, 24 and 42 months</td>
<td><u>Spirometry</u>
Forced expiratory volume in one second (FEV$_1$) (l)

Forced vital capacity (FVC) (l)

FEV$_1$/FVC: Tiffeneau-Pinelli index (%)

Maximum mid-expiratory flow (FEF$_{25-75\%}$) (l/min)

<u>Exhaled air</u>
Carbon monoxide (eCO) (ppm)

Fractional exhaled nitric oxide (FeNO) (ppb)

<u>High-resolution computed tomography (HRCT)</u></td>
<td><u>Spirometry and exhaled air at three follow-up visits</u>
<table>
<tr><th></th><th>Baseline</th><th>Follow-up 1</th><th>Follow-up 2</th><th>Follow-up 3</th></tr>
<tr><td colspan="5">FEV$_1$ (mean (SD)) - p=0.30</td></tr>
<tr><td>ENDS</td><td>3.8 (0.8)</td><td>3.8 (0.8)</td><td>3.8 (0.7)</td><td>3.9 (0.8)</td></tr>
<tr><td>Control</td><td>4.1 (0.3)</td><td>4.1 (0.3)</td><td>4.0 (0.3)</td><td>4.1 (0.3)</td></tr>
<tr><td colspan="5">FVC (mean (SD)) - p=0.61</td></tr>
<tr><td>ENDS</td><td>4.9 (1.0)</td><td>4.8 (0.8)</td><td>4.8 (0.9)</td><td>4.9 (0.8)</td></tr>
<tr><td>Control</td><td>5.0 (0.5)</td><td>5.0 (0.4)</td><td>5.0 (0.5)</td><td>5.0 (0.4)</td></tr>
<tr><td colspan="5">FEV$_1$/FVC (mean (SD)) - p=0.09</td></tr>
<tr><td>ENDS</td><td>78.5% (3.5)</td><td>79.0% (3.6)</td><td>78.5% (2.3)</td><td>79.1% (2.8)</td></tr>
<tr><td>Control</td><td>81.5% (5.0)</td><td>82.0% (4.7)</td><td>80.9% (6.2)</td><td>82.1% (4.3)</td></tr>
<tr><td colspan="5">FEF$_{25-75\%}$ (mean (SD)) - p=0.36</td></tr>
<tr><td>ENDS</td><td>3.3 (0.7)</td><td>3.3 (0.6)</td><td>3.3 (0.8)</td><td>3.3 (0.6)</td></tr>
<tr><td>Control</td><td>3.4 (0.6)</td><td>3.5 (0.6)</td><td>3.5 (0.6)</td><td>3.6 (0.6)</td></tr>
<tr><td colspan="5">eCO (median and IQR) - p=0.21</td></tr>
<tr><td>ENDS</td><td>5.0 [3.5-7.3]</td><td>4.0 [2.8-6.0]</td><td>3.0 [3.0-5.8]</td><td>4.0 [2.8-6.3]</td></tr>
<tr><td>Control</td><td>4.0 [3.5-7.5]</td><td>5.5 [4.0-6.5]</td><td>7.0 [3.5-8.0]</td><td>5.0 [5.5-6.0]</td></tr>
<tr><td colspan="5">FeNO (median and IQR) - p=0.89</td></tr>
<tr><td>ENDS</td><td>21.1 [16.2-24.5]</td><td>19.7 [17.2-22.3]</td><td>18.9 [18.2-24.7]</td><td>20.0 [18.2-22.7]</td></tr>
<tr><td>Control</td><td>18.6 [17.6-25.7]</td><td>19.4 [16.0-25.1]</td><td>18.7 [16.9-22.0]</td><td>20.0 [16.2-23.4]</td></tr>
</table>
<u>High-resolution computed tomography at 42 months</u>
HRCT scans obtained in 8/9 e-cigarette users. Visual assessment of the HRCT scans showed no pathological findings</td>
<td>Moderate methodological quality

Very small study size

<u>Conflicts of interest</u>
Grants and consulting/speaking fees from pharmaceutical companies and electronic cigarette industry and trade associations

<u>Funding</u>
Supported by Catania University</td>
</tr>
<tr><td colspan="6">Non-randomised intervention studies</td></tr>
</table>

Study details (author, year location, study type, time frame, [data source])	Sample characteristics	Intervention and control	Outcome measure	Results	Quality assessment, study size, conflict of interest, funding
Kotoulas et al., 2020<br><br>Greece<br><br>Pre-post-post intervention study<br><br>Study date not reported<br><br>Laboratory study	Study size<br>50 participants (25 mildly asthmatic smokers, 25 healthy smokers)<br><br>Sample<br>All participants were current daily smokers of combustible tobacco<br><br>Gender - n (%)<br>Male: 21/50 (42%)<br>Female: 29/50 (58%)<br><br>Age - mean (SD) years<br>Asthmatic smokers<br>40.6 (10.8)<br><br>Healthy smokers<br>39.9 (10.2)	Exposure (n=25)<br>E-cigarette<br><br>Comparator (n=25)<br>Before and after<br><br>Materials - Device type<br>NOBACCO (Halandri, Greece), powered by a lithium battery with 1.2 Ω coil resistance<br><br>Materials - nicotine concentration<br>“Medium nicotine content”<br><br>Pattern of exposure<br>Used e-cigarette for 5 mins (10 puffs with 30 second inter-puff intervals, 1.0-1.5mL of e-liquid)	Pulmonary function<br>Forced vital capacity (FVC) (l)<br><br>Forced expiratory volume in one second (FEV_1) (l)<br><br>FEV_1/FVC: Tiffeneau-Pinelli index (%)<br><br>Peak expiratory flow (PEF) (l/s)<br><br>Residual volume (RV) (l)<br><br>Expiratory reserve volume (ERV) (l)<br><br>Total lung capacity (TLC) (l)<br><br>Respiratory resistance<br>Respiratory impedance at 5Hz (Z5Hz) (kPa/L/s)<br><br>Respiratory resistance at 5 (R5Hz), 10 (R10Hz), and 20Hz (R20Hz) (kPa/L/s)<br><br>Exhaled air<br>Exhaled FeNO (ppb)	Pulmonary function, respiratory resistance and exhaled air before and after e-cigarette use (see results table below)	High methodological quality<br><br>Small study size<br><br>Conflicts of interest<br>Not reported<br><br>Funding<br>Supported by Hellenic Society of Respiratory and Occupational Chest Diseases

Pulmonary function, respiratory resistance and exhaled air before and after e-cigarette use

		Pre	Post	Diff	P	P*
FVC	Healthy	4.02 (0.91)	4.03 (0.90)	+0.01	0.696	0.480
	Asthma	4.45 (1.15)	4.43 (1.17)	−0.02	0.534	
FVC (predict)	Healthy	104.61 (15.17)	104.74 (13.62)	+0.13	0.873	0.977
	Asthma	104.61 (14.2)	103.88 (13.62)	−0.73	0.726	
FEV_1	Healthy	3.42 (0.79)	3.39 (0.79)	−0.03	0.267	0.628
	Asthma	3.43 (0.90)	3.39 (0.91)	−0.04	0.113	
FEV_1 (predict)	Healthy	105.20 (16.67)	104.06 (14.29)	−1.14	0.125	0.865
	Asthma	95.94 (13.18)	94.64 (14.29)	−1.30	0.067	
FEV_1/FVC	Healthy	82.63 (6.95)	81.80(6.38)	−0.83	0.169	0.677
	Asthma	75.19 (8.23)	74.58 (7.96)	−0.61	0.040	
FEV_1/FVC (predict)	Healthy	101.83 (7.60)	100.82 (6.98)	−1.01	0.175	0.684
	Asthma	93.26 (9.25)	92.52 (9.01)	−0.74	0.042	
PEF	Healthy	7.42 (1.75)	7.23 (2.17)	−0.19	0.321	0.467
	Asthma	7.58 (2.02)	7.12 (2.08)	−0.46	0.003	
PEF (predict)	Healthy	98.80 (21.51)	94.78 (22.40)	−4.02	0.141	0.600
	Asthma	92.03 (19.55)	84.84 (19.02)	−7.19	0.001	
RV	Healthy	1.51 (0.43)	1.53 (0.50)	+0.01	0.59	0.946
	Asthma	1.87 (0.53)	1.89 (0.44)	+0.02	0.772	
RV (predict)	Healthy	87.30 (14.91)	88.32 (18.03)	+1.02	0.757	0.900
	Asthma	100.43 (26.64)	101.69 (21.59)	+1.26	0.738	
ERV	Healthy	1.08 (0.48)	1.06 (0.49)	−0.02	0.818	0.157
	Asthma	1.44 (0.65)	1.29 (0.57)	−0.15	0.051	
ERV (predict)	Healthy	87.52 (36.43)	84.84 (32.09)	−2.68	0.583	0.221
	Asthma	108.88 (39.00)	96.69 (28.97)	−12.19	0.053	
TLC	Healthy	5.56 (0.95)	5.59 (0.97)	+0.03	0.277	0.066
	Asthma	6.20 (1.33)	6.13 (1.28)	−0.07	0.141	
TLC (predict)	Healthy	97.41 (9.60)	97.88 (8.08)	+0.47	0.426	0.126
	Asthma	97.52 (12.4)	96.58 (11.33)	−0.94	0.187	
Z5Hz	Healthy	0.440 (0.098)	0.461 (0.106)	+0.021	0.063	0.515
	Asthma	0.431 (0.121)	0.464 (0.149)	+0.033	0.040	
R5Hz	Healthy	0.426 (0.099)	0.450 (0.105)	+0.024	0.034	0.712
	Asthma	0.419 (0.115)	0.449 (0.142)	+0.030	0.054	
R10Hz	Healthy	0.382 (0.096)	0.402 (0.098)	+0.020	0.038	0.668
	Asthma	0.376 (0.104)	0.403 (0.128)	+0.027	0.043	
R20Hz	Healthy	0.367 (0.097)	0.388 (0.098)	+0.021	0.034	0.816
	Asthma	0.362 (0.101)	0.386 (0.114)	+0.024	0.026	
FeNO	Healthy	15.12 (6.48)	11.84 (5.19)	−3.28	<0.001	<0.001
	Asthma	14.88 (11.60)	18.48 (13.38)	+3.60	0.001	

*mean difference between asthmatic and healthy smokers

<table>
<tr><th>Study details (author, year location, study type, time frame, [data source])</th><th>Sample characteristics</th><th>Intervention and control</th><th>Outcome measure</th><th>Results</th><th>Quality assessment, study size, conflict of interest, funding</th></tr>
<tr>
<td>Brożek et al., 2019

Poland

Laboratory pre-post study

Study date not reported

YoUng People E-smoking Study (YUPESS) - multi-centre international project</td>
<td><u>Study size</u>
120 participants: 30 participants in each exposure group

<u>Sample</u>
1. Exclusive e-cigarette users
2. Dual users
3. Exclusive cigarette smokers
4. Non-smokers

<u>Gender (%)</u>
Male: 71/120 (59.2%)
Female: 49/120 (40.8%)

<u>Age - mean (SD) years</u>
22.6 2.2</td>
<td><u>Exposure 1 (n=30)</u>
Exclusive e-cigarette users

<u>Exposure 2 (n=30)</u>
Dual users

<u>Exposure 3 (n=30)</u>
Exclusive cigarette smokers

<u>Comparator (n=30)</u>
Non-smokers

<u>Materials - device type</u>
ENDS: own device, multi-fruit flavoured e-liquid
Cigarette: popular cigarette brand (0.6mg nicotine/cigarette)

<u>Pattern of exposure</u>
Everyday habits for 5 minutes</td>
<td><u>Spirometry</u>
Forced vital capacity (FVC) (l)

Forced expiratory volume in one second (FEV_1) (l)

Forced expiratory volume in one second to FVC (FEV_1/FVC) (%)

Peak expiratory flow (PEF) (l/s)

Maximal expiratory flow at 25% and 75% of FVC ($MEF_{25,75}$) (l/s)

Maximal expiratory flow between 25% and 75% of FVC (MEF_{25-75}) (l/s)

<u>Acute respiratory responses</u>
Exhaled nitric oxide (FeNO) (ppb)

O_2 saturation (%)

Exhaled air temperature (°C)

Exhaled carbon monoxide (CO) (ppm)</td>
<td><u>Relative difference since baseline - mean (SD)</u>
<table>
<tr><th></th><th>ENDS</th><th>Cigarette</th><th>Dual</th><th>Non-smoker</th><th>P</th></tr>
<tr><td>FVC (1 min)</td><td>1.0 (4.1)</td><td>1.5 (4.9)</td><td>−0.5 (6.8)</td><td>−0.8 (3.0)</td><td>0.2</td></tr>
<tr><td>FVC (30 mins)</td><td>−0.2 (3.9)</td><td>0.2 (5.4)</td><td>1.4 (4.4)</td><td>-</td><td>0.4</td></tr>
<tr><td>FEV_1 (1 min)</td><td>2.3 (5.7)</td><td>2.8 (7.2)</td><td>−0.2 (6.4)</td><td>−0.3 (3.7)</td><td>0.4</td></tr>
<tr><td>FEV_1 (30 mins)</td><td>1.0 (6.3)</td><td>1.7 (7.3)</td><td>0.4 (5.2)</td><td>-</td><td>0.8</td></tr>
<tr><td>FEV_1/FVC (1 min)</td><td>1.3% (4.3)</td><td>1.3% (5.9)</td><td>0.2% (2.7)</td><td>0.6% (2.4)</td><td>0.8</td></tr>
<tr><td>FEV_1/FVC (30 mins)</td><td>1.3% (4.4)</td><td>1.6% (5.2)</td><td>−1.0% (3.8)</td><td>-</td><td>0.09</td></tr>
<tr><td>PEF (1 min)</td><td>3.8 (12.0)</td><td>4.6 (13.5)</td><td>5.5 (9.9)</td><td>2.4 (13.0)</td><td>0.9</td></tr>
<tr><td>PEF (30 mins)</td><td>5.5 (15.3)</td><td>0.2 (17.0)</td><td>1.0 (17.0)</td><td>-</td><td>0.5</td></tr>
<tr><td>MEF_{25} (1 min)</td><td>5.3 (16.0)</td><td>4.3 (14.4)</td><td>−7.3 (19.1)</td><td>3.5 (15.6)</td><td>0.02</td></tr>
<tr><td>MEF_{25} (30 mins)</td><td>0.8 (19.3)</td><td>1.4 (13.3)</td><td>−2.8 (16.1)</td><td>-</td><td>0.6</td></tr>
<tr><td>MEF_{75} (1 min)</td><td>3.1 (10.5)</td><td>3.0 (15.7)</td><td>4.9 (10.7)</td><td>1.3 (13.6)</td><td>0.6</td></tr>
<tr><td>MEF_{75} (30 mins)</td><td>4.1 (14.6)</td><td>1.8 (16.4)</td><td>−0.2 (15.0)</td><td>-</td><td>0.9</td></tr>
<tr><td>MEF_{25-75} (1 min)</td><td>4.2 (11.8)</td><td>4.8 (12.5)</td><td>−0.5 (11.1)</td><td>0.9 (9.6)</td><td>0.7</td></tr>
<tr><td>MEF_{25-75} (30 mins)</td><td>2.7 (11.2)</td><td>3.8 (13.2)</td><td>−2.0 (10.6)</td><td>-</td><td>0.5</td></tr>
<tr><td>FeNO (1 min)</td><td>7.3 (13.4)</td><td>13.1 (11.2)</td><td>12.8 (16.7)</td><td>0.3 (13.4)</td><td>0.0002</td></tr>
<tr><td>FeNO (30 mins)</td><td>−8.4 (18.6)</td><td>−3.9 (11.9)</td><td>−5.6 (18.5)</td><td>-</td><td>0.5</td></tr>
<tr><td>O_2 saturation (1 min)</td><td>−0.1% (1.1)</td><td>0.6% (1.1)</td><td>0.2% (0.8)</td><td>0.2% (0.7)</td><td>0.09</td></tr>
<tr><td>O_2 saturation (30 mins)</td><td>−0.1% (0.9)</td><td>−0.0% (1.1)</td><td>0.1% (1.0)</td><td>-</td><td>0.6</td></tr>
<tr><td>Exhaled air temp (1 min)</td><td>−0.5 (1.2)</td><td>0.0 (1.1)</td><td>−0.5 (0.9)</td><td>−0.2 (1.1)</td><td>0.4</td></tr>
<tr><td>Exhaled air temp (30 mins)</td><td>−0.7 (1.3)</td><td>−0.9 (1.0)</td><td>−0.6 (1.0)</td><td>-</td><td>0.4</td></tr>
<tr><td>Exhaled CO (1 min)</td><td>−11.9 (27.7)</td><td>−154.4 (115.1)</td><td>−1.1 (13.8)</td><td>−11.1 (31.4)</td><td>0.0001</td></tr>
<tr><td>Exhaled CO (30 mins)</td><td>−8.9 (26.9)</td><td>−117.6 (90.5)</td><td>11.0 (19.2)</td><td>-</td><td>0.0001</td></tr>
</table>
In the control group, under direction of the Ethics Committee, the 30-minute measurement was not allowed since the first and second measurement results did not differ</td>
<td>Moderate methodological quality

Moderate study size

<u>Conflicts of interest</u>
None declared

<u>Funding</u>
Medical University of Silesia</td>
</tr>
</table>

Study details (author, year location, study type, time frame, [data source])	Sample characteristics	Intervention and control	Outcome measure	Results	Quality assessment, study size, conflict of interest, funding
Coppeta et al., 2018<br><br>Italy<br><br>Crossover study<br><br>Study date not reported<br><br>Laboratory study	Study size<br>30 participants<br><br>Sample<br>Healthy non-smoker volunteers<br><br>Gender - n (%)<br>Male: 17/30 (57%)<br>Female: 13/30 (43%)<br><br>Age - mean (SD) years<br>32.6 (2.75)	Exposure<br>ENDS: 1.8% (18mg/mL)<br><br>Comparator<br>Tobacco cigarette: 0.6mg nicotine, 8mg tar, 9mg CO<br><br>Materials - device type<br>eGo P (L) with manual start, Latakia tobacco flavour<br><br>Pattern of exposure<br>15 puffs of ENDS	Spirometry<br>Forced expiratory volume in one second (FEV$_1$) (l)<br><br>Forced expiratory volume in one second to forced vital capacity (FEV$_1$/FVC) (%)<br><br>Forced expiratory flow between 25% and 75% of FVC (FEF$_{25-75}$) (l/s)	Lung function parameters (baseline, 1 minute and 15 minutes) for the traditional cigarette and the e-cigarette (see table below)	Moderate methodological quality<br><br>Small study size<br><br>Conflicts of interest<br>None declared<br><br>Funding<br>Not reported

Lung function parameters (baseline, 1 minute and 15 minutes) for the traditional cigarette and the e-cigarette

	Mean					95% CI		
	Baseline	Post	Diff	SD	SE	Lower	Upper	P
FEV$_1$ (Post = 1 min)								
ENDS	3.55	3.51	0.04	0.11	0.02	0.00	0.09	0.03
Cigarette	3.53	3.48	0.04	0.10	0.028	0.01	0.08	0.00
FEV$_1$ (Post = 15 mins)								
ENDS	3.55	3.53	0.02	0.14	0.03	-0.03	0.07	0.36
Cigarette	3.53	3.51	0.02	0.054	0.016	0.01	0.04	0.05
FEV$_1$/FVC (Post = 1 min)								
ENDS	82.1%	81.6%	1.03%	2.00	0.37	0.29	1.78	0.01
Cigarette	82.2%	81.7%	0.5%	1.28	0.38	0.98	1.02	0.04
FEV$_1$/FVC (Post = 15 mins)								
ENDS	82.1%	81.5%	0.40%	2.49	0.46	-0.53	1.33	0.39
Cigarette	82.2%	81.0%	1.2%	1.16	0.35	0.75	1.68	0.01
FEF$_{25-75}$ (Post = 1 min)								
ENDS	3.44	3.30	0.23	0.31	0.06	0.12	0.35	0.00
Cigarette	3.45	3.38	0.06	0.13	0.04	0.01	0.11	0.01
FEF$_{25-75}$ (Post = 15 mins)								
ENDS	3.44	.35	0.09	0.32	0.06	0.02	0.25	0.03
Cigarette	3.45	3.31	0.14	0.14	0.04	0.08	0.12	0.00

Study details (author, year location, study type, time frame, [data source])	Sample characteristics	Intervention and control	Outcome measure	Results	Quality assessment, study size, conflict of interest, funding
Lappas et al., 2018<br><br>Greece<br><br>Pre-post intervention study<br><br>Study date not reported<br><br>Laboratory study	Study size<br>54 participants (27 asthmatic smokers, 27 healthy smokers)<br><br>Sample<br>Dual e-cigarettes and combustible cigarettes. Smokers were healthy or with mild intermittent well controlled asthma<br><br>Gender - n (%)<br>Male: 21/54 (39%)<br>Female: 33/54 (61%)<br><br>Age - mean (SD) years<br>23.0 (3.2)	Exposure<br>ENDS: 12mg/mL nicotine<br><br>Comparator<br>Before<br><br>Materials - device type<br>New-generation e-cigarette (adjustable voltage), propylene glycol 46.13% w/v, glycerol 34.3% w/v, nicotine 1.18% w/v and tobacco essence (<5% w/v)<br><br>Pattern of exposure<br>Use for five minutes (10 puffs). Follow-up immediately after, 15 and 30 minutes after session	Impulse oscillometry<br>Respiratory system total impedance at 5Hz (Z5) (kPa/(L/s))<br><br>Respiratory system resistance at 5Hz/10Hz/20Hz (R5/R10/R20) (kPa/(L/s))<br><br>Resonant frequency (f_{res}) (Hz)<br><br>Respiratory system reactance at 5Hz/20Hz (X5/X20) (kPa/(L/s))<br><br>Reactance area (AX) (kPa/L)	See results tables below	High methodological quality<br><br>Small study size<br><br>Conflicts of interest<br>None declared<br><br>Funding<br>Behrakis Foundation

Results (Lappas et al., 2018):

Impulse oscillometry parameters - mean difference at baseline

	Z5	R5	R10	R20	FRes	X5	X20	AX
Healthy	0.33 (0.07)	0.31 (0.06)	0.29 (0.06)	0.29 (0.06)	10.43 (2.01)	-0.10 (0.03)	0.10 (0.03)	0.24 (0.12)
Asthmatic	0.38 (0.08)	0.37 (0.08)	0.33 (0.07)	0.33 (0.06)	12.4 (4.2)	-0.11 (0.03)	0.08 (0.05)	0.36 (0.32)
P	0.011	0.009	0.008	0.043	0.032	0.435	0.094	0.065

Impulse oscillometry - mean (SD) difference baseline to follow-up

	Directly after	P	15 mins post	P	30 mins post	P
Z5						
Healthy	0.36 (0.09)	<0.001	0.34 (0.08)	0.154	0.33 (0.08)	>0.999
Asthma	0.44 (0.09)	<0.001	0.40 (0.08)	0.128	0.38 (0.06)	>0.999
R5						
Healthy	0.34 (0.08)	<0.001	0.33 (0.08)	0.183	0.31 (0.08)	>0.999
Asthma	0.42 (0.08)	<0.001	0.38 (0.07)	0.238	0.36 (0.06)	>0.999
R10						
Healthy	0.31 (0.07)	0.001	0.30 (0.07)	0.293	0.29 (0.08)	>0.999
Asthma	0.38 (0.07)	<0.001	0.35 (0.06)	0.184	0.33 (0.05)	>0.999
R20						
Healthy	0.31 (0.06)	0.033	0.30 (0.06)	0.465	0.30 (0.07)	>0.999
Asthma	0.36 (0.07)	<0.001	0.34 (0.06)	0.250	0.33 (0.05)	>0.999
F_{res}						
Healthy	11.61 (3.05)	0.001	11.04 (2.78)	0.389	10.38 (2.43)	>0.999
Asthma	14.07 (4.48)	<0.001	12.45 (3.82)	>0.999	11.77 (3.46)	0.339
X5						
Healthy	-0.10 (0.03)	>0.999	-0.10 (0.03)	>0.999	-0.09 (0.03)	>0.999
Asthma	-0.12 (0.04)	<0.001	-0.10 (0.03)	>0.999	-0.10 (0.03)	>0.999
X20						
Healthy	0.08 (0.04)	<0.001	0.09 (0.04)	0.076	0.12 (0.11)	0.616
Asthma	0.05 (0.05)	<0.001	0.08 (0.05)	>0.999	0.08 (0.05)	>0.999
AX						
Healthy	0.33 (0.23)	0.041	0.28 (0.2)	0.490	0.23 (0.15)	>0.999
Asthma	0.55 (0.53)	<0.001	0.37 (0.28)	>0.999	0.30 (0.22)	0.108

Percentages and p-values are presented as reported in original studies.

AOR = adjusted odds ratio; ATR = airway total resistance; AX = reactance area; CI = confidence interval; CO = carbon monoxide; COPD = chronic obstructive pulmonary disease; CPD = cigarette(s) per day; diff = difference; DL_{CO} = diffusion capacity of carbon monoxide; eCO = exhaled carbon monoxide; ENDS = electronic nicotine delivery system; ENNDS = electronic non-nicotine delivery system; ERV = expiratory reserve volume; FEF = forced expiratory flow; $FEF_{25-75\%}$ = maximum mid-expiratory flow; FeNO = fractional exhaled nitric oxide; FEV_1 = forced expiratory volume in one second; FEV_1/FVC = Tiffeneau-Pinelli index; fres = resonance frequency; FVC = forced vital capacity; GOLD = Global Initiative for Chronic Obstructive Lung Disease; gly = glycerin; HRCT = high-resolution computed tomography; IGV = intrathoracic gas volume; IQR = interquartile range; kPa = kilopascal(s); max = maximum; $MEF_{25,75}$ = maximal expiratory flow at 25% and 75% of FVC; MEF_{25-75} = maximal expiratory flow between 25% and 75% of FVC; NHLBI = National Heart, Lung, and Blood Institute; NIH = National Institutes of Health; PATH = Population Assessment of Tobacco and Health; PEF = peak expiratory flow; PG = propylene glycol; ppb = part(s) per billion; ppm = part(s) per million; R5/R10/R11/R13/R17/R19/R20 = respiratory or flow resistance at 5Hz/10Hz/11Hz/13Hz/17Hz/19Hz/20Hz; R5-19Hz = difference of R5Hz and R19Hz; RV = residual volume; SD = standard deviation; SE = standard error; SPIROMICS = SubPopulations and InteRmediate Outcome Measures In COPD Study; TLC = total lung capacity; UK = United Kingdom; US = United States; VC = vital capacity; w/v = weight by volume; X5/X20 = reactance at 5Hz/20Hz; YUPESS = YoUng People E-smoking Study; Z5Hz = respiratory impedance at 5Hz.

Table 3.2. Study details: respiratory health outcomes – surveillance reports

<table>
<tr><th>Study details (author, year, location, time frame, data source)</th><th>Demographics</th><th>Exposure (e-liquid description)</th><th>Presentation and symptoms</th><th>Treatment</th><th>Outcome</th><th>Quality assessment, conflict of interest, funding</th></tr>
<tr><td colspan="7">National surveillance systems</td></tr>
<tr><td>Adkins et al., 2020

US

August 2019 - December 17, 2019

CDC</td><td>EVALI cases: 2,155

<u>Gender (N=2,141) - n (%)</u>
Female: 671 (31.3%)
Male: 1,470 (68.7%)

<u>Age (N=2,155) - n (%)</u>
13-17 years: 360 (16.7%)
18-24 years: 859 (39.9%)
25-49 years: 936 (43.4%)</td><td><u>ENDS patterns of use in past 90 days - n</u>
Any ENDS or vaping: 1,793
Exclusive ENDS or vaping: 1,793
Daily ENDS or vaping: 603
ENDS and THC: 1,793</td><td><u>EVALI symptoms - n</u>
Respiratory: 1,532
Gastrointestinal: 1,452
Constitutional*: 1,523
Gastrointestinal or constitutional symptoms, but no respiratory symptoms: 1,477

* Fever, chills, malaise</td><td><u>EVALI clinical course and treatment - n</u>
Hospitalisation: 2,026
ICU admission: 1,300
Corticosteroids: 1,203
Intubated: 632</td><td>Not reported</td><td>High methodological quality

<u>Conflicts of interest</u>
None declared

<u>Funding</u>
Not reported</td></tr>
<tr><td>Ellington et al., 2020

US

August 2019 - January 7, 2020

CDC</td><td>EVALI cases: 2,602

<u>Gender (N=2,486) - n (%)</u>
Female: 828 (33%)
Male: 1,658 (67%)

<u>Age (N=2,497) - n (%)</u>
13-17 years: 383 (15%)
18-24 years: 931 (37%)
25-34 years: 605 (24%)
35-44 years: 322 (13%)
45-64 years: 213 (9%)
65-85 years: 43 (2%)</td><td><u>E-cigarette composition 3 months preceding symptom onset (N=1,979) - n (%)</u>
Any nicotine: 1,128 (57%)</td><td>Not reported</td><td><u>Clinical course - n (%)</u>
| | Severe* | Not severe |
All (N=2,533)	810 (32%)	1,723 (68%)
Any Nicotine (N=1,122)	409 (36%)	713 (64%)
Exclusive nicotine (N=262)	156 (60%)	106 (40%) | *Hospital stay ≥10 days, ICU admission, endotracheal intubation, continuous airway pressure, bilevel airway pressure or death</td><td><u>Outcome - n (%)</u> |	Died	Survived
All (N=2,533)	57 (2%)	2,298 (98%)
Any nicotine (N=1,060)	26 (2%)	1,034 (98%)
Only nicotine (N=244)	16 (7%)	228 (93%) |</td><td>Grey literature-no quality assessment <u>Conflicts of interest</u> None declared <u>Funding</u> Not reported</td></tr>
<tr><td>Evans et al., 2020

US

August 2019 - December 10, 2019

CDC</td><td>Hospitalised EVALI: 2,409

<u>Median age - years</u>
Died: 54
Rehospitalised: 27
Neither died nor rehospitalised: 23</td><td>Not reported</td><td>Not reported</td><td>Not reported</td><td>Deaths: 52/2,409 (2%)

<u>Outcomes after discharge (N=1,139) - n (%)</u>
Rehospitalised: 31 (2.7%)
Died: 7 (0.6%)</td><td>Grey literature-no quality assessment

<u>Conflicts of interest</u>
One member of the Lung Injury Response Clinical Working Group reported receiving grants and personal fees from the FDA/NIH and the pharmaceutical industry

<u>Funding</u>
Not reported</td></tr>
</table>

Study details (author, year, location, time frame, data source)	Demographics	Exposure (e-liquid description)	Presentation and symptoms	Treatment	Outcome	Quality assessment, conflict of interest, funding
Krishnasamy et al., 2020<br><br>US<br><br>August 2019 - January 14, 2020<br><br>CDC and the National Syndromic Surveillance Program (NSSP)	Hospitalised EVALI cases (N=2,668) - n (%)<br>Confirmed: 1,401 (53%)<br>Probable: 1,267 (47%)<br><br>Gender (N=2,606) - n (%)<br>Female: 875 (34%)<br>Male: 1,731 (66%)<br><br>Age (N=2,619) - n (%)<br>13-17 years: 404 (15%)<br>18-24 years: 979 (37%)<br>25-34 years: 631 (24%)<br>35-44 years: 335 (13%)<br>45-64 years: 223 (9%)<br>≥65 years: 47 (2%)<br><br>Median age (range) years: 24 (13-85)	E-cigarette composition 3 months preceding symptom onset (N=2,022) - n (%)<br>Any nicotine: 1,162 (57%)<br>Both THC and nicotine: 834 (41%)<br>Exclusive nicotine: 274 (14%)<br>No THC or nicotine: 44 (2%)	Not reported	Not reported	Not reported	Grey literature-no quality assessment<br><br>Conflicts of interest<br>None declared<br><br>Funding<br>Not reported
Mikosz et al., 2020<br><br>US<br><br>August 2019 - December 10, 2019<br><br>CDC	Hospitalised EVALI: 2,409<br><br>Gender (N=804) - n (%)<br>Female: 275 (34%)<br>Male: 528 (66%)<br>Other: 1 (0%)<br><br>Age (N=804) - n (%)<br>13-17 years: 136 (17%)<br>18-24 years: 309 (38%)<br>25-50 years: 309 (38%)<br>≥51 years: 50 (6%)	Not reported	Symptoms at first reported clinical encounter - n (%)<br>Any respiratory: 758/792 (96%)<br>Any constitutional*: 710/775 (92%)<br>Any gastrointestinal: 621/762 (81%)<br><br>*Fever, chills, malaise, fatigue, headache, body aches	Clinical course - n (%)<br>Corticosteroids: 577/653 (88%)<br>ICU admission: 299/702 (43%)<br>Respiratory failure necessitating intubation and mechanical ventilation: 60/360 (17%)<br>Extracorporeal membrane oxygenation: 5/479 (1%)	Outcome - n (%)<br>Deaths: 52/2,409 (2%)<br>Rehospitalisation: 31<br>Death after discharge: 7<br>No rehospitalisation nor death: 768	Grey literature-no quality assessment<br><br>Conflicts of interest<br>None declared<br><br>Funding<br>Not reported
Werner et al., 2020<br><br>US<br><br>August 2019 - January 7, 2020<br><br>CDC	Hospitalised EVALI cases (N=2,618) - n (%)<br>Confirmed: 1,378 (53%)<br>Probable: 1,240 (47%)<br><br>Gender (N=2,558) - n (%)<br>Female: 860 (34%)<br>Male: 1,698 (66%)<br><br>Age (N=2,574) - n (%)<br><35 years: 1,979 (77%)<br>≥35 years: 595 (23%)<br><br>Median age (range) years<br>Fatal cases: 51 (15-75)<br>Non-fatal cases: 24 (13-85)	E-cigarette composition and pattern of use 3 months preceding symptom onset (N=2,066) - n (%)<br>Nicotine (non-exclusive): 1,134 (55%)<br>Nicotine (exclusive): 292 (14%)<br>THC and nicotine: 815 (39%)<br>Neither THC nor nicotine: 124 (6%)	Symptoms - n (%)<br>Respiratory: 1,762/1,835 (96%)<br>Gastrointestinal: 1,369/1,730 (79%)	Clinical course - n (%)<br>Antibiotics: 1,211/1,240 (98%)<br>Glucocorticoids: 1,297/1,477 (88%)<br>ICU admission: 690/1,561 (44%)<br>Endotracheal intubation: 178/813 (22%)<br>Ventilatory support (CPAP or BiPAP): 211/1,124 (19%)	Deaths: 60/2,618 (2%)	High methodological quality<br><br>Conflicts of interest<br>None declared<br><br>Funding<br>Not reported

Study details (author, year, location, time frame, data source)	Demographics	Exposure (e-liquid description)	Presentation and symptoms	Treatment	Outcome	Quality assessment, conflict of interest, funding
Blount et al., 2019<br><br>US<br><br>August 2019 - October 15, 2019<br><br>CDC	EVALI cases: 867	Substances used in the 3 months preceding symptom onset - %<br>THC-containing products: 86%	Not reported	Not reported	Not reported	Grey literature-no quality assessment<br><br>Conflicts of interest<br>None declared<br><br>Funding<br>Not reported
Chatham-Stephens et al., 2019<br><br>US<br><br>August 2019 - November 5, 2019<br><br>CDC	EVALI case status (N=2,006) - n (%)<br>Confirmed: 1,052 (52%)<br>Probable: 954 (48%)<br><br>Gender (N=1,905) - n (%)<br>Female: 607 (32%)<br>Male: 1,298 (68%)<br><br>Age (N=1,906) - n (%)<br>13-17 years: 293 (15%)<br>18-24 years: 721 (38%)<br>25-34 years: 459 (24%)<br>35-44 years: 256 (13%)<br>45-64 years: 141 (7%)<br>≥65 years: 36 (2%)<br><br>Median age (range) years: 24 (13-78)	E-cigarette composition used 3 months preceding symptom onset (N=1,184) - n (%)<br>Any nicotine: 723 (61%)<br>Both THC and nicotine: 573 (48%)<br>Nicotine only: 150 (13%)<br>No THC or nicotine: 50 (4%)	Symptoms among non-hospitalised EVALI cases - n (%)<br>Any respiratory: 47/55 (85%)<br>Any constitutional: 41/54 (76%)<br>Any gastrointestinal: 27/47 (57%)<br><br>Symptoms (cases with complete information; N=47) - n (%)<br>Respiratory only: 4 (9%)<br>Gastrointestinal only: 0 (0%)<br>Constitutional only*: 1 (2%)<br><br>*Fever, chills, weight loss	Not reported	EVALI cases and hospitalisation status (N=2,016) - n (%)<br>Hospitalised: 1,906 (95%)<br>Non-hospitalised: 110 (5%)	Grey literature-no quality assessment<br><br>Conflicts of interest<br>None declared<br><br>Funding<br>Not reported
Jatlaoui et al., 2019<br><br>US<br><br>August 2019 - November 13, 2019<br><br>CDC	EVALI cases: 2,172	Not reported	Not reported	Not reported	Deaths: 42/2,172 (1.9%)	Grey literature-no quality assessment<br><br>Conflicts of interest<br>None declared<br><br>Funding<br>Not reported
Lozier et al., 2019[374]<br><br>US<br><br>August 2019 - December 3, 2019<br><br>CDC	Hospitalised EVALI cases: 2,291<br><br>EVALI status (N=2,288) - n (%)<br>Confirmed: 1,221 (53%)<br>Probable: 1,067 (47%)<br><br>Gender (N=2,155) - n (%)<br>Female: 706 (33%)<br>Male: 1,499 (67%)	E-cigarette composition and pattern of use 3 months preceding symptom onset (N=1,782) - n (%)<br>Any nicotine: 956 (54%)<br>Nicotine only: 227 (13%)<br>Daily nicotine: 482 (85%)<br>Both THC and nicotine: 713 (40%)	Not reported	Not reported	Deaths: 48/2,291 (2%)	Grey literature-no quality assessment<br><br>Conflicts of interest<br>None declared<br><br>Funding<br>Not reported

Study details (author, year, location, time frame, data source)	Demographics	Exposure (e-liquid description)	Presentation and symptoms	Treatment	Outcome	Quality assessment, conflict of interest, funding
	Age (N=2,159) - n (%)<br>13-17 years: 341 (16%)<br>18-24 years: 817 (38%)<br>25-34 years: 524 (24%)<br>35-44 years: 278 (13%)<br>45-64 years: 165 (8%)<br>≥65 years: 34 (2%)<br><br>Median age (range) years: 24 (13-77)					
Moritz et al., 2019<br><br>US<br><br>August 2019 - October 15, 2019<br><br>CDC	EVALI cases: 1,378<br><br>Gender (N=1,378) - n (%)<br>Female: 414 (30%)<br>Male: 964 (70%)<br><br>Age (N=1,364) - n (%)<br>13-17 years: 196 (14%)<br>18-24 years: 541 (40%)<br>25-34 years: 344 (25%)<br>35-44 years: 172 (13%)<br>45-64 years: 87 (6%)<br>65-75 years: 24 (2%)<br><br>Median age (range) years: 24 (13-75)	E-cigarette composition used 3 months preceding symptom onset (N=867) - n (%)<br>Any THC: 749 (86%)<br>Any nicotine: 522 (64%)<br>Both THC and nicotine: 455 (52%)<br>THC only: 294 (34%)<br>Nicotine only: 97 (11%)<br>No THC or nicotine: 21 (2%)	Not reported	Not reported	Not reported	Grey literature-no quality assessment<br><br>Conflicts of interest<br>None declared<br><br>Funding<br>Not reported
Perrine et al., 2019<br><br>US<br><br>August 2019 - September 24, 2019<br><br>CDC	EVALI cases: 805<br><br>Gender (N=771) - n (%)<br>Female: 234 (30%)<br>Male: 531 (69%)<br>Missing: 6 (1%)<br><br>Age (N=771) - n (%)<br><18 years: 125 (16%)<br>18-24 years: 293 (38%)<br>25-34 years: 184 (24%)<br>35-44 years: 93 (12%)<br>≥45 years: 42 (6%)<br>Missing: 34 (4%)	Product use (N=514) - n (%)<br>Any THC: 395 (77%)<br>Any nicotine: 292 (57%)<br>Nicotine only: 82 (16%)<br><br>E-cigarette composition used in the 3 months preceding symptom onset (N=514) - n (%)<br>(Yes / No / Missing)<br>Nicotine: 292 (57%) / 173 (34%) / 49 (10%)<br>Flavoured e-liquid: 102 (20%) / 132 (26%) / 280 (55%)	Not reported	Not reported	Deaths: 12/805 (2%)	Grey literature-no quality assessment<br><br>Conflicts of interest<br>None declared<br><br>Funding<br>Not reported
Schier et al., 2019<br><br>US<br><br>August 2019 - August 27, 2019<br><br>CDC	215 possible cases of severe pulmonary disease	Not reported	Not reported	Not reported	Not reported	Grey literature-no quality assessment<br><br>Conflicts of interest<br>None declared<br><br>Funding<br>Not reported

Study details (author, year, location, time frame, data source)	Demographics	Exposure (e-liquid description)	Presentation and symptoms	Treatment	Outcome	Quality assessment, conflict of interest, funding
Siegel et al., 2019<br><br>US<br><br>August 2019 - October 3, 2019<br><br>CDC	EVALI cases: 1,299*<br><br><u>Gender (N=1,043) - n (%)</u><br>Female: 313 (30%)<br>Male: 730 (70%)<br><br><u>Age (only where full medical chart available (N=338)</u><br>Median age (range) years: 22 (13-71)<br><br>*October 8, 2019	<u>E-cigarette composition used 3 months preceding symptom onset (N=573) - n (%)</u><br>Any THC: 435 (76%)<br>Any nicotine: 332 (58%)<br>THC only: 183 (32%)<br>Nicotine only: 74 (13%)	<u>Symptoms (only where full medical chart available) (N=339) - n (%)</u><br>Any respiratory: 323 (95%)<br>Any constitutional*: 289 (85%)<br>Any gastrointestinal: 262 (77%)<br><br>*Self-reported fever, chills, and unexpected weight loss	<u>Clinical course (only where full medical chart available) - n (%)</u><br>Corticosteroids: 252/287 (88%)<br>ICU admission: 159/342 (47%)<br>Intubation and mechanical ventilation: 74/338 (22%)<br>Average hospital stay [mean (median) days]: 6.7 (5)	Deaths: 26/1,299 (2%)*<br><br>*October 8, 2019	Grey literature-no quality assessment<br><br><u>Conflicts of interest</u><br>One member of the Lung Injury Response Clinical Working Group received grants and fees from the pharmaceutical industry<br><br><u>Funding</u><br>Not reported
State-based surveillance systems						
Armatas et al., 2020<br><br>California, US<br><br>2019-2020<br><br>California Department of Public Health (CDPH)	<u>Hospitalised EVALI cases</u><br>June 18, 2019-February 23, 2020: 210 patients<br>April 2020: 8 patients<br><br><u>Age range (April 2020) (N=8)</u><br>14-50 years (median: 17 years); n=7 aged <21 years	<u>April 2020 (N=8) - n (%)</u><br>THC: 6 (75%)<br>ENDS only: 1 (13%)<br>Unspecified: 1 (13%)	Not reported	<u>Clinical course, April 2020, (N=8) - n (%)</u><br>ICU admission: 4 (50%)<br>Mechanical ventilation: 2 (25%)<br>SARS-CoV-2 testing: all negative<br><br><u>Hospitalisation</u><br>Median (range) days: 4 (4-13)	Not reported	Grey literature-no quality assessment<br><br><u>Conflicts of interest</u><br>None declared<br><br><u>Funding</u><br>Not reported
Gaub et al., 2019<br><br>Indiana, US<br><br>August 8-October 28, 2019<br><br>Indiana State Department of Health (ISDH)	<u>Hospitalised EVALI cases (N=97) - n (%)</u><br>Confirmed: 41 (42%)<br>Probable: 56 (58%)<br><br><u>Gender (N=54) - n (%)</u><br>Male: 38 (70%)<br>Female: 16 (30%)<br><br><u>Age (N=54) - n (%)</u><br>13-17 years: 7 (13%)<br>18-29 years: 27 (50%)<br>30-39 years: 12 (22%)<br>40-49 years: 3 (6%)<br>50-59 years: 3 (6%)<br>≥60 years: 2 (4%)<br><br>Median age (range) years: 26 (16-68)	Not reported	<u>Symptoms on admission (N=54) - n (%)</u><br>Shortness of breath: 48 (89%)<br>Cough: 44 (81%)<br>Nausea: 27 (50%)<br>Vomiting: 27 (50%)<br>Chest pain: 17 (31%)<br>Diarrhea: 15 (28%)<br>Abdominal pain: 12 (22%)<br>Sweating: 11 (20%)<br>Weight loss: 8 (15%)	<u>Medical care - n (%)</u><br>Antibiotics: 44/51 (86%)<br>Steroids: 34/52 (65%)<br>Bronchoscopy: 13/44 (30%)<br>ICU admission: 13/51 (25%)<br>Lung biopsy: 7/45 (16%)<br>Intubation/mechanical ventilation: 7/50 (14%)	Deaths: 3/97 (3%)	Grey literature-no quality assessment<br><br><u>Conflicts of interest</u><br>None declared<br><br><u>Funding</u><br>Not reported

Study details (author, year, location, time frame, data source)	Demographics	Exposure (e-liquid description)	Presentation and symptoms	Treatment	Outcome	Quality assessment, conflict of interest, funding
Lewis et al., 2019<br><br>Utah, US<br><br>August 6-October 15, 2019<br><br>Utah Department of Health (UDOH)	Confirmed or probable cases of EVALI: 83<br><br><u>Gender (N=83) - n (%)</u><br>Female: 14 (17%)<br>Male: 69 (83%)<br><br><u>Age (N=83) - n (%)</u><br>14-19 years: 11 (13%)<br>20-29 years: 43 (52%)<br>30-39 years: 23 (28%)<br>40-66 years: 6 (7%)<br><br>Median age (range) years: 26 (14-66)	Not reported	Not reported	<u>Medical care (N=79) - n (%)</u><br>Hospitalisation: 70 (89%)<br>Steroids: 59 (75%)<br>ICU admission: 35 (44%)<br>CPAP/BiPAP support* (no intubation): 30 (38%)<br>Acute respiratory distress syndrome: 20 (25%)<br>Intubation and mechanical ventilation: 9 (11%)<br><br>*Continuous positive airway pressure/bilevel positive airway pressure	Not reported	Grey literature-no quality assessment<br><br><u>Conflicts of interest</u><br>None declared<br><br><u>Funding</u><br>Not reported
Taylor et al., 2019<br><br>Minnesota, US<br><br>August 9-October 31, 2019<br><br>Minnesota Department of Health (MDH)	Confirmed or probable EVALI cases: 96<br><br><u>Gender (N=96) - n (%)</u><br>Female: 38 (40%)<br>Male: 58 (60%)<br><br>Median age (range) years: 21 (15-71)	Not reported	Not reported	<u>Clinical course (N=96) - n (%)</u><br>Hospitalised: 87 (91%)<br>ICU admission: 26 (27%)	Deaths: 3/96 (3%)	Grey literature-no quality assessment<br><br><u>Conflicts of interest</u><br>None declared<br><br><u>Funding</u><br>Not reported

Percentages and p-values are presented as reported in original studies.
BiPAP = bilevel positive airway pressure; CDC = Centers for Disease Control and Prevention; CDPH = California Department of Public Health; CPAP = continuous positive airway pressure; ENDS = electronic nicotine delivery system; EVALI = e-cigarette or vaping product use-associated lung injury; FDA = Food and Drug Administration (US); ICU = Intensive Care Unit; ISDH = Indiana State Department of Health; MDH = Minnesota Department of Health; NIH = National Institutes of Health; NSSP = National Syndromic Surveillance Program; SARS-CoV-2 = severe acute respiratory syndrome coronavirus 2; THC = tetrahydrocannabinol; UDOH = Utah Department of Health; US = United States.

Table 3.3. Study details: respiratory health outcomes - case reports and case series

Study details (author, year, location, data source [time frame])	Demographics and medical history	Exposure	Presentation	Treatment	Outcome	Quality assessment, conflict of interest and funding
Case series						
**Ansari-Gilani et al., 2020**<br><br>US<br><br>Hospital record<br><br>Time frame: not reported	Female<br>20 years<br><br><u>Medical history</u><br>Never smoker, no past medical history	Nicotine e-cigarette use for 3 months, last used night before presentation	Dyspnoea, cough, intermittent diarrhea, nausea<br><br><u>EVALI diagnosis</u><br>Confirmed case (hypersensitivity pneumonitis)	Antibiotics, steroids, supplemental oxygen	Discharged after 11 days, significant improvement in follow-up clinic	High methodological quality<br><br><u>Conflicts of interest</u><br>None declared<br><br><u>Funding</u><br>Not reported

<table>
<tr><th>Study details (author, year, location, data source [time frame])</th><th>Demographics and medical history</th><th>Exposure</th><th>Presentation</th><th>Treatment</th><th>Outcome</th><th>Quality assessment, conflict of interest and funding</th></tr>
<tr><td>Corcoran et al., 2020

US

Hospital record

August-November 2019</td><td>Male
17 years

<u>Medical history</u>
Hypertension</td><td>2 years: daily nicotine-e-cigarette pods</td><td>Nausea, vomiting, cough, fever, dyspnoea for four days

<u>EVALI diagnosis</u>
Probable case</td><td>Nasal cannula, paediatric intensive care unit (PICU), antibiotics</td><td>Discharged after 6 days</td><td>Moderate methodological quality

<u>Conflicts of interest</u>
None declared

<u>Funding</u>
National Heart, Lung, and Blood Institute</td></tr>
<tr><td>Fryman et al., 2020

US

Hospital record

November 2018-August 2019</td><td>Female
62 years

<u>Medical history</u>
Mild intermittent asthma</td><td>6 months: nicotine-based products</td><td>Dyspnoea and abdominal pain for one month

<u>EVALI diagnosis</u>
Confirmed case (acute respiratory failure)</td><td>Antibiotics</td><td>Improved over 5 days without steroids, discharged home</td><td>Moderate methodological quality

<u>Conflicts of interest</u>
None declared

<u>Funding</u>
None declared</td></tr>
<tr><td rowspan="2">Isakov et al., 2020

US

Hospital record

Time frame: not reported

* Authors do not specify if the case is confirmed or probable EVALI</td><td>Male
36 years

<u>Medical history</u>
Previously healthy, nil tobacco/illicit drug use</td><td>Frequent e-cigarette use, variety of flavours</td><td>Fever, cough, weakness, weight loss for four weeks

<u>EVALI diagnosis</u>
Confirmed/probable case* (organising pneumonia)</td><td>Not reported</td><td>Not reported</td><td rowspan="2">Low methodological quality

<u>Conflicts of interest</u>
None declared

<u>Funding</u>
None received</td></tr>
<tr><td>Male
18 years

<u>Medical history</u>
History of opiate use</td><td>Not reported</td><td>Lower back pain, headache, dyspnoea, fever

<u>EVALI diagnosis</u>
Confirmed/probable case* (acute lung injury)</td><td>Paediatric intensive care unit (PICU), antibiotics</td><td>Discharged after 6 days</td></tr>
<tr><td rowspan="3">Kass et al., 2020

US

Hospital record

April 2019-January 2020</td><td>Male
16 years

<u>Medical history</u>
Appendicitis after surgical intervention</td><td>Intermittent use for 1 year</td><td>Dry cough, general malaise, decreased appetite, chills, fever, dyspnoea, vomiting

<u>EVALI diagnosis</u>
Confirmed case</td><td>Intubation, nasal cannula, antibiotics, steroids</td><td>Discharged after 23 days</td><td rowspan="3">Low methodological quality

<u>Conflicts of interest</u>
None declared

<u>Funding</u>
Not reported</td></tr>
<tr><td>Male
16 years

<u>Medical history</u>
Allergy-induced asthma, delayed puberty, small stature, renal diverticulum, penile adhesions</td><td>2 years: up to 3 times/week</td><td>Fever, nausea, vomiting, diarrhoea

<u>EVALI diagnosis</u>
Confirmed case</td><td>Antibiotics, nasal cannula</td><td>Discharged after 8 days</td></tr>
<tr><td>Female
15 years

<u>Medical history</u></td><td>Rare personal use of Juul and mod device (unknown brand), but frequent ‘hotboxing’ (filling closed</td><td>Cough, dyspnoea, sputum production</td><td>Antibiotics, steroids</td><td>Not reported</td></tr>
</table>

Study details (author, year, location, data source [time frame])	Demographics and medical history	Exposure	Presentation	Treatment	Outcome	Quality assessment, conflict of interest and funding
	Possible asthma, chronic joint pain, sinopulmonary infections	space (car) with e-cigarette exhalant)	EVALI diagnosis<br>Neither confirmed nor probable case (imaging is normal)			
**Temas & Meyer, 2020**<br><br>US<br><br>Hospital record<br><br>July-August 2019	Male<br>33 years<br><br>Medical history<br>Remote history of asthma as child, community-acquired pneumonia two years prior, current smoker (one pack/day)	Regular use and used "all night" prior to presentation	Cough, dyspnoea, fever for two days, hypoxia, tachycardia<br><br>EVALI diagnosis<br>Confirmed case	Nasal cannula, antibiotics, steroids	Discharged on day 6 with steroid taper	High methodological quality<br><br>Conflicts of interest<br>None declared<br><br>Funding<br>Not reported
**Thakrar et al., 2020**<br><br>US<br><br>Hospital record<br><br>June 2019-August 2019	Male<br>16.5 years<br><br>Medical history<br>Not reported	E-cigarette 6-8 months prior, daily use for several weeks prior to admission	Not reported per patient, no information<br><br>EVALI diagnosis<br>Confirmed case	Admitted to hospital and received high-dose steroids	Not reported	Moderate methodological quality<br><br>Conflicts of interest<br>None declared<br><br>Funding<br>Not reported
	Male<br>17.0 years<br><br>Medical history<br>Not reported	Use of nicotine e-cigarette 3-5 days/week for unknown duration	Not reported per patient, no information<br><br>EVALI diagnosis<br>Confirmed case	Admitted to hospital and received high-dose steroids	Not reported	
	Male<br>17.7 years<br><br>Medical history<br>Not reported	Daily use of nicotine e-cigarette for 2-3 months, most recent use five months prior to admission	Not reported per patient, no information<br><br>EVALI diagnosis<br>Confirmed case	Admitted to hospital and received high-dose steroids	Not reported	
	Male<br>17.5 years<br><br>Medical history<br>Not reported	Daily use of nicotine e-cigarette for unknown duration	Not reported per patient, no information<br><br>EVALI diagnosis<br>Confirmed case	Admitted to hospital and received high-dose steroids	Not reported	
	Male<br>17.7 years<br><br>Medical history<br>Not reported	Daily use of nicotine e-cigarettes for 4 months	Not reported per patient, no information<br><br>EVALI diagnosis<br>Confirmed case	Admitted to hospital and received high-dose steroids	Not reported	
Case reports						

Study details (author, year, location, data source [time frame])	Demographics and medical history	Exposure	Presentation	Treatment	Outcome	Quality assessment, conflict of interest and funding
Edmonds et al., 2020<br><br>US<br><br>Hospital record<br><br>Time frame not reported	Female<br>31 years<br><br>Medical history<br>Former smoker (pack/day), vaginal delivery five weeks prior, untreated hepatitis c virus, chronic pain, PTSD, family history (systemic lupus erythematosus and scleroderma), medications (buprenorphine/naloxone, prazosin, venlafaxine)	Switched to e-cigarettes four years prior to presentation: 17mL of 3mg/mL nicotine fiery cinnamon e-liquid daily	Productive cough, haemoptysis<br><br>EVALI diagnosis<br>Confirmed case (diffuse alveolar haemorrhage)	Antibiotics	Haemoptysis gradually resolved during hospitalisation/cessation of e-cigarette use	High methodological quality<br><br>Conflicts of interest<br>None declared<br><br>Funding<br>U.S. Department of Veterans Affairs
Farooq et al., 2020<br><br>US<br><br>Hospital record<br><br>Time frame not reported	Male<br>19 years<br><br>Medical history<br>Multiple emergency department visits over four months prior (diffuse abdominal pain, nausea, vomiting, diarrhoea)	1 year: intermittent use of nicotine e-cigarettes	Acute gastroenteritis, hypoxia<br><br>EVALI diagnosis<br>Confirmed case	Antibiotics, antifungal therapy, steroids	Hypoxia improved with treatment, asymptomatic at follow-up with e-cigarette abstinence	Moderate methodological quality<br><br>Conflicts of interest<br>None declared<br><br>Funding<br>None received
Patterson et al., 2020<br><br>UK<br><br>Hospital record<br><br>Time frame not reported	Male<br>"In his 40s"<br><br>Medical history<br>Former smoker (twenty-pack/year), appendectomy, marijuana use in distant past	Switched to e-cigarettes 6 weeks prior: 18mg/mL nicotine, peppermint flavour	Coryzal symptoms, pleuritic chest pain, dyspnoea, hypoxia, tachycardia<br><br>EVALI diagnosis<br>Confirmed case (severe acute respiratory distress syndrome)	Intubation, mechanical ventilation, veno-venous extracorporeal membrane oxygenation (ECMO)	Survived, repatriated to referring hospital	Low methodological quality<br><br>Conflicts of interest<br>None declared<br><br>Funding<br>Not reported
Sakla et al., 2020<br><br>US<br><br>Hospital record<br><br>Time frame not reported	Female<br>25 years<br><br>Medical history<br>Unremarkable medical history	One year: use two-three hours/day, three times/week	Pleuritic chest pain, dyspnoea, dry cough, hyperventilation<br><br>EVALI diagnosis<br>Confirmed case (acute respiratory distress syndrome)	Saline, antibiotics, intubation, veno-venous extracorporeal membrane oxygenation (ECMO)	ECMO for three weeks, currently under care of speech management to establish dietary goals	Moderate methodological quality<br><br>Conflicts of interest<br>None declared<br><br>Funding<br>Not reported
Venkatnarayan et al., 2020<br><br>India<br><br>Hospital record<br><br>Time frame not reported	Male<br>31 years<br><br>Medical history<br>Smoker of 6 years (unclear if still using), nil known comorbidities, nil history of fever, haemoptysis, chest pain, palpitations or orthopnoea	3 months nicotine e-cigarettes, multiple flavours: last exposure four days before symptom onset	Acute onset breathlessness, dry cough for 3 days<br><br>EVALI diagnosis<br>Confirmed case	Nebulised bronchodilators and beta-agonists (after initial acute bronchitis diagnosis), antibiotics, antivirals, steroids	Condition significantly improved with treatment, advised not to use e-cigarettes, given smoking cessation advice	Moderate methodological quality<br><br>Conflicts of interest<br>None declared<br><br>Funding<br>None received

Study details (author, year, location, data source [time frame])	Demographics and medical history	Exposure	Presentation	Treatment	Outcome	Quality assessment, conflict of interest and funding
Aftab et al., 2019<br><br>US<br><br>Hospital record<br><br>Time frame not reported	Female<br>46 years<br><br>Medical history<br>Asthma, remote history of using marijuana and cocaine, nil history of lung disease, recent travel or sick contact	E-cigarette use for 1 month prior to admission	Dyspnoea and dry cough for 2 days<br><br>EVALI diagnosis<br>Confirmed case (acute respiratory distress syndrome)	High flow nasal cannula, antibiotics, intubation, high-dose steroids	Recovered/discharged to rehabilitation centre after 12 days, participated in physical therapy	Moderate methodological quality<br><br>Conflicts of interest<br>None declared<br><br>Funding<br>None received
Casanova et al., 2019<br><br>Spain<br><br>Hospital record<br><br>Time frame not reported	Female<br>31 years<br><br>Medical history<br>Unremarkable medical history	Daily use of nicotine e-cigarettes (with e-liquid) for 3 months, used nicotine salts (same device) in week preceding admission	Fever, myalgia, dry cough, fatigue and dyspnoea for 3 days<br><br>EVALI diagnosis<br>Confirmed case	Antibiotics, steroids	Discharged after 12 days	High methodological quality<br><br>Conflicts of interest<br>None declared<br><br>Funding<br>Not reported
Sommerfeld et al., 2018<br><br>US<br><br>Hospital record<br><br>Time frame not reported	Female<br>18 years<br><br>Medical history<br>Mild intermittent exertional asthma, recent reaction to Brazil nut, nil recent travel or animal exposure	2-3 weeks e-cigarette use, used 1-2 days before symptom onset	Dyspnoea, cough, pleuritic chest pain, afebrile<br><br>EVALI diagnosis<br>Confirmed case (hypersensitivity pneumonitis)	Paediatric intensive care unit (PICU), antibiotics, intubation, norepinephrine therapy, bilateral chest tubes, steroids	Discharged on steroid taper	Moderate methodological quality<br><br>Conflicts of interest<br>None declared<br><br>Funding<br>No external funding

Percentages and p-values are presented as reported in original studies.
ECMO = extracorporeal membrane oxygenation; EVALI = e-cigarette or vaping product use-associated lung injury; PICU = paediatric intensive care unit; PTSD = post-traumatic stress disorder; UK = United Kingdom; US = United States.

4. Oral Health

Table 4.1. Study details: oral health – cohort studies

Study details (author, year, location, study type time frame, data source)	Sample characteristics	Intervention/ exposure and comparator	Outcome measure	Results	Quality assessment, study size, conflicts of interest, funding
**Atuegwu et al., 2019**<br><br>US<br><br>Longitudinal cohort study<br><br>2013-2016<br><br>Population Assessment of Tobacco and Health (PATH) waves 1-3	<u>Study size</u><br>32,320 adults without gum disease at baseline; 18,289 participants in analysis<br><br><u>Sample</u><br>Never electronic nicotine product user: no use<br>Regular electronic nicotine product user: regular (regularly every day or some days) across waves<br>Infrequent electronic product user: ever users that did not use electronic nicotine product regularly every day or some days across waves<br><br><u>Gender - male % (95% CI)</u><br>Never users: 44.4% (43.7-45.1)<br>Regular users: 53.2% (46.7-59.7)<br>Infrequent users: 52.3% (51.2-53.4)<br><br><u>Age - % (95% CI) years</u><br>18-24: / 25-34: / 35-44: / 45-54: / 55+<br>Never users<br>9.6% (9.2-10) / 15.7% (14.8-16.6) / 17.4% (16.5-18.3) / 19.3% (18. 5-20.1) / 38% (37-39)<br>Regular users<br>23.8% (19.5-28.2) / 30.8% (24.4-37.1) / 15.9% (10.5-21.3) / 14.4% (9.5-19.3) / 15.1% (11.6-18.5)<br>Infrequent users<br>30.8% (29.8-31.8) / 29% (27.8-30.3) / 16.6% (15.6-17.6) / 12.4% (11.6-13.3) / 11.1% (10.2-12)	<u>Exposure 1 (n=329)</u><br>Regular electronic nicotine product user<br><br><u>Exposure 2 (n=8,298)</u><br>Infrequent electronic nicotine product user<br><br><u>Comparator (n=9,632)</u><br>Never electronic nicotine product user<br><br><u>Materials</u><br>Device details unknown<br><br><u>Follow-up</u><br>3 years	<u>New cases of gum disease</u><br>Baseline to wave 2 or 3<br><br><u>Bone loss</u><br>Around teeth, baseline to wave 3<br><br><u>Any periodontal disease</u><br>Baseline to wave 2 or 3. Diagnosis past 12 months	<u>Oral health outcomes - n (%) [95% CI]</u><br>(Never users (N=9,632) / Regular users (N=329) / Infrequent users (N=8,298))<br>New cases of gum disease: 491 (5.1%) [4.5-5.6] / 32 (9.8%) [6.4-13.3] / 515 (6.2%) [5.6-6.7]<br>Bone loss around teeth: 809 (8.4%) [7.6-9.2] / 37 (11.2%) [7.6-14.8] / 606 (7.3%) [6.6-8.1]<br>Any periodontal disease: 1127 (11.7%) [10.8-12.6] / 55 (16.7%) [12.2-21.2] / 946 (11.4%) [10.6-12.2]<br><br>Results of the Multivariable Logistic Regression Models - OR (95% CI)<br>(New cases of gum disease / Bone loss around teeth / Any periodontal disease)<br>Never users: Reference / Reference / Reference<br>Regular users: 1.76 (1.12-2.76) / 1.67 (1.06-2.63) / 1.58 (1.06-2.34)<br>Infrequent users: 1.09 (0.87-1.35) / 1.10 (0.91-1.33) / 1.09 (0.93-1.29)<br>Adjusted for age, gender, race, education, income, history of illicit/prescription drug use, tobacco, alcohol and marijuana use history, history of ulcers, respiratory disease, diabetes, high blood pressure, high cholesterol, dental visits	High methodological quality<br><br>Large study size<br><br><u>Conflicts of interest</u><br>None declared<br><br><u>Funding</u><br>Support from the NIH

Percentages and p-values are presented as reported in original studies.
CI = confidence interval; NIH = National Institutes of Health; OR = odds ratio; PATH = Population Assessment of Tobacco and Health; US = United States.

5. Developmental and reproductive

Table 5.1. Study details: developmental and reproductive outcomes – cohort studies and cross-sectional surveys

<table>
<tr><th>Study details (author, year, location study type [time frame, data source])</th><th>Sample characteristics</th><th>Exposure/Comparison groups</th><th>Outcome measure</th><th>Results</th><th>Quality assessment, study size, conflicts of interest, funding</th></tr>
<tr><td colspan="6">Cohort studies</td></tr>
<tr>
<td>McDonnell et al., 2020

Ireland

Prospective cohort study

No data period provided

Large urban maternity hospital</td>
<td>Study size
620 participants who gave birth to live singleton infants

Sample
ENDS: e-cigarette use at any point during pregnancy excluding those that quit after conception and before first study visit
Never smokers: never smoked

Age - mean (SD) years
ENDS: 31 (5.3)
Never smokers: 33 (5.9)</td>
<td>Exposure (n=218)
Exclusive ENDS users

Comparator (n=108)
Never smokers

Materials
Device and nicotine concentrations not specified

Follow-up
13 months</td>
<td>Birthweight (g)

Mean birth centile

Incidence of birthweight < 10th centile

Mean gestation at delivery

Mean Apgar score

Neonatal Intensive Care Unit (NICU) admission

Breastfeeding at discharge</td>
<td>
<table>
<tr><th>Outcome</th><th>ENDS (N=218)
n (%)</th><th>Never smokers (N=108)
n (%)</th><th>ENDS compared to never smokers</th></tr>
<tr><td>Birthweight (g)</td><td>3470 (555)</td><td>3471 (504)</td><td>p=0.97</td></tr>
<tr><td>Mean birth centile</td><td>47th</td><td>47th</td><td></td></tr>
<tr><td>Incidence of birthweight <10th percentile</td><td>24 (11%)</td><td>14 (12.9%)</td><td>p=0.60</td></tr>
<tr><td>Mean gestation at delivery</td><td>39+3</td><td>39+4</td><td></td></tr>
<tr><td>Mean Apgar score</td><td>9, 10</td><td>9, 10</td><td></td></tr>
<tr><td>NICU admission</td><td>15 (6.9%)</td><td>5 (4.6%)</td><td>p=0.42</td></tr>
<tr><td>Breastfeeding at discharge</td><td>106 (48.6%)</td><td>66 (61.1%)</td><td>p=0.03</td></tr>
</table>
</td>
<td>High methodological quality

Small study size

Conflicts of interest
None declared

Funding
Friends of the Coombe' research charity and by Coombe Women and Infants University Hospital</td>
</tr>
</table>

<table>
<tr><th>Study details (author, year, location study type [time frame, data source])</th><th>Sample characteristics</th><th>Exposure/Comparison groups</th><th>Outcome measure</th><th>Results</th><th>Quality assessment, study size, conflicts of interest, funding</th></tr>
<tr>
<td>Cardenas et al., 2019

US

Prospective cohort study

2015-2017

University affiliated pregnancy centre in Little Rock, Arkansas</td>
<td><u>Study size</u>
248 participants who gave birth to live singleton infants

<u>Sample</u>
Exclusive ENDS: ENDS use within the previous month
Dual users: current ENDS and smoking
Smokers: smoking in the previous month
Unexposed: non-current smokers/non-current ENDS users not exposed to secondhand smoke or ENDS aerosols or other tobacco products

<u>Age - n (%) years</u>
18-22: 94/248 (37.9%)
23-27: 76/248 (30.6%)
≥28: 78/248 (31.5%)

<u>Ethnicity - n (%)</u>
Non-Hispanic Black: 112/248 (45.2%)
Non-Hispanic White: 95/248 (38.3%)
Hispanic: 30/248 (12.1%)
Other: 11/248 (4.4%)</td>
<td><u>Exposure 1 (n=6)</u>
Exclusive current ENDS

<u>Exposure 2 (n=17)</u>
Dual users

<u>Exposure 3 (n=56)</u>
Current smokers

<u>Comparator (n=97)</u>
Unexposed

<u>Materials</u>
Device and nicotine concentrations not specified

<u>Follow-up</u>
6 months</td>
<td>Birthweight

Smallness for gestational age (SGA)</td>
<td><u>Pregnancy outcomes (n=232)</u>
<table>
<tr><th></th><th>Multivariate* mean z-score birthweight difference (SE)</th><th>SGA - n (%)</th><th>SGA multivariate* risk ratio (95% CI)</th></tr>
<tr><td>Exclusive ENDS (n=6)</td><td>-0.498 (0.411)</td><td>2 (33.3%)</td><td>3.1 (0.8-11.7)</td></tr>
<tr><td>Current smoker (n=56)</td><td>-0.482 (0.177)**</td><td>13 (23.1%)</td><td>1.9 (0.9-4.3)</td></tr>
<tr><td>Dual (n=17)</td><td>-0.297 (0.266)</td><td>4 (23.5%)</td><td>1.9 (0.6-5.5)</td></tr>
<tr><td>Unexposed (n=97)</td><td>0 (Referent)</td><td>11 (11.3%)</td><td>1 (Referent)</td></tr>
</table>
* Model included maternal age and race/ethnicity as covariates
** p<0.05

<u>Pregnancy outcomes, excluding unexposed participants who returned positive cotinine or carbon monoxide tests (n=199)</u>
<table>
<tr><th></th><th>Multivariate* mean z-score birthweight difference (SE)</th><th>SGA - n (%)</th><th>SGA multivariate* risk ratio (95% CI)</th></tr>
<tr><td>Exclusive ENDS (n=6)</td><td>-0.540 (0.417)</td><td>2 (33.3%)</td><td>5.1 (1.2-22.2)</td></tr>
<tr><td>Current smoker (n=56)</td><td>0.490 (0.190)**</td><td>13 (23.1%)</td><td>2.6 (0.9-7.2)</td></tr>
<tr><td>Dual (n=17)</td><td>-0.303 (0.274)</td><td>4 (23.5%)</td><td>2.5 (0.7-8.8)</td></tr>
<tr><td>Unexposed (n=64)</td><td>0 (Referent)</td><td>5 (7.8%)</td><td>1 (Referent)</td></tr>
</table>
* Model included maternal age and race/ethnicity as covariates
** p<0.05</td>
<td>High methodological quality

Very small study size

<u>Conflicts of interest</u>
None declared

<u>Funding</u>
None received</td>
</tr>
<tr><td colspan="6">Cross-sectional surveys</td></tr>
</table>

<table>
<tr><th>Study details (author, year, location study type [time frame, data source])</th><th>Sample characteristics</th><th>Exposure/Comparison groups</th><th>Outcome measure</th><th>Results</th><th>Quality assessment, study size, conflicts of interest, funding</th></tr>
<tr>
<td>Wang et al., 2020

US

Cross-sectional

2016

Pregnancy Risk Assessment Monitoring System (PRAMS)</td>
<td><u>Study size</u>
31,793 participants who gave birth to live singleton infants

<u>Sample</u>
Exclusive ENDS, sole smokers, dual users and non-users as reported 3 months before and last 3 months of pregnancy

No demographic data reported</td>
<td><u>Exposure 1 (n=126)</u>
ENDS: ENDS and other electronic nicotine products (vape pens, e-hookahs, hookah pens, e-cigars, e-pipes) in the last 3 months of pregnancy

<u>Exposure 2 (n=2,632)</u>
Smokers: smoked cigarettes in the last 3 months of pregnancy

<u>Exposure 3 (n=265)</u>
Dual: concurrent ENDS and cigarette use in the last 3 months of pregnancy

<u>Comparator (n=28,770)</u>
Non-users

<u>Materials</u>
Not specified</td>
<td>Preterm

Small-for-gestational-age</td>
<td><u>Smoking and e-cigarette use 3 months before pregnancy and in the last 3 months of pregnancy</u>
<table>
<tr><th></th><th colspan="4">Status in the last 3 months of pregnancy (n)</th></tr>
<tr><th>Status 3 months pre-pregnancy</th><th>Neither</th><th>Smoker</th><th>ENDS</th><th>Dual user</th></tr>
<tr><td>Neither</td><td>25,501</td><td>17</td><td>3</td><td>0</td></tr>
<tr><td>Exclusive smoker</td><td>2,622</td><td>2342</td><td>18</td><td>47</td></tr>
<tr><td>Exclusive ENDS</td><td>215</td><td>3</td><td>49</td><td>0</td></tr>
<tr><td>Dual user</td><td>432</td><td>270</td><td>56</td><td>218</td></tr>
<tr><td>Total</td><td>28,770</td><td>2,632</td><td>126</td><td>265</td></tr>
</table>
<u>Adjusted odds ratios (95% CI) for pregnancy outcomes associated with tobacco use in the last 3 months of pregnancy</u>
<table>
<tr><th></th><th>ENDS</th><th>Smoker</th><th>Dual user</th></tr>
<tr><td>Preterm</td><td>1.6 (0.7-3.4)</td><td>1.5 (1.2-1.8)</td><td>1.2 (0.8-2.0)</td></tr>
<tr><td>Small-for-gestational-age</td><td>2.0 (0.8-4.7)</td><td>2.6 (2.2-3.1)</td><td>2.2 (1.3-3.8)</td></tr>
<tr><td colspan="4"><u>Adjusted for pre-pregnancy smoking/e-cigarette status</u></td></tr>
<tr><td>Preterm</td><td>1.2 (0.5-2.7)</td><td>1.6 (1.2-2.0)</td><td>1.3 (0.8-2.3)</td></tr>
<tr><td>Small-for-gestational-age</td><td>2.4 (1.0-5.7)</td><td>2.4 (1.8-2.9)</td><td>2.3 (1.3-4.1)</td></tr>
</table>
Adjusted for: mother’s age, education level, race/ethnicity, marital status, previous preterm history, plurality, Kotelchuck index of prenatal care, pre-pregnancy BMI, drinking alcohol before pregnancy, and gestational weight gain</td>
<td>High methodological quality

Large study size

<u>Conflicts of interest</u>
None declared

<u>Funding</u>
No specific funding</td>
</tr>
</table>

Percentages and p-values are presented as reported in original studies.
CI = confidence interval; ENDS = electronic nicotine delivery system; NICU = Neonatal Intensive Care Unit; PRAMS = Pregnancy Risk Assessment Monitoring System; SD = standard deviation; SE = standard error; SGA = smallness for gestational age; US = United States.

6. Burns and injuries

Table 6.1. Study details: burns and injuries – surveillance reports

Study details (author, year, location, time frame, data source)	Demographic characteristics	Circumstance of injury	Presentation or details of injuries	Treatment	Outcome and recovery	Quality assessment, study size, conflict of interest, funding
McFaull et al., 2020<br><br>Canada<br><br>2013-2019<br><br>Canadian Hospitals Injury Reporting and Prevention Program network	N=4<br><br>Demographic information not reported	Explosion or overheating of the device: 2<br>Swallowed part of device: 1<br>Crushing injury by piece of disassembled device: 1	Thigh burn: n=2<br>Foreign body in alimentary tract: n=1<br>Crushing injury to finger: n=1	Not reported	Not reported	Low methodological quality<br><br>Very small study size<br><br><u>Conflicts of interest</u><br>None declared<br><br><u>Funding</u><br>Not reported
Wang et al., 2020<br><br>US<br><br>2010-2019<br><br>National Poison Data System (NPDS)	N=69<br><br><u>Gender (N=69) - n (%)</u><br>Male: 39 (56.5%)<br>Female: 28 (40.6%)<br>Unknown: 2 (2.9%)<br><br><u>Age (N=69) - n (%) years</u><br><5: 2 (2.9%)<br>5-11: 0 (0.0%)<br>12-17: 8 (11.5%)<br>18-24: 20 (29.0%)<br>25+: 30 (43.5%)<br>Unknown: 9 (13.0%)	Not reported	<u>Type of Burn (N=69) - n (%)</u><br>Thermal: 42 (60.9%)<br>Chemical: 21 (30.4%)<br>Both Thermal and Chemical: 5 (7.2%)<br>Not Specified: 1 (1.4%)<br><br><u>Body Part Burned (N=69) - n (%)</u><br>More than One Body Part: 18 (26.1%)<br>Face Only: 23 (33.3%)<br>Leg/Thigh Only: 13 (18.8%)<br>Hand Only: 10 (14.5%)<br>Shoulder/Chest Only: 1 (1.4%)<br>Genitals Only: 1 (1.4%)<br>Not Specified: 3 (4.3%)<br><br><u>Severity of Burn - n (%)</u><br>Superficial burn: 40 (58.0%)<br>Second- or third-degree burn: 25 (36.2%)<br>Oral burn: 5 (7.3%)<br>Not specified: 7 (10.1%)	<u>Treatment (N=69) - n (%)</u><br>Admitted: 4 (5.8%)<br>Treated, evaluated, and released: 45 (65.2%)<br>Not referred: 11 (15.9%)<br>Refused referral: 3 (4.4%)<br>Lost to follow-up: 6 (8.7%)	<u>Outcome (N=69) - n (%)</u><br>Minor, resolved rapidly: 21 (30.4%)<br>Moderate: 33 (47.8%)<br>Major, life-threatening: 2 (2.9%)<br>Not followed-up: 13 (18.9%)	High methodological quality<br><br><u>Conflicts of interest</u><br>None declared<br><br><u>Funding</u><br>Supported by the Center for Tobacco Products, U.S. Food and Drug Administration

Study details (author, year, location, time frame, data source)	Demographic characteristics	Circumstance of injury	Presentation or details of injuries	Treatment	Outcome and recovery	Quality assessment, study size, conflict of interest, funding
Dohnalek & Harley, 2019<br><br>US<br><br>2007-2017<br><br>National Electronic Injury Surveillance System (NEISS)	N=49<br><br>Sex unweighted (N=49) - n (%)<br>Male: 47 (95.9%)<br>Female: 2 (4.1%)<br><br>Age unweighted (N=49) - n (%) years<br><18: 3 (6.1%)<br>18-29: 26 (53.1%)<br>30-44: 14 (28.6%)<br>45-60: 5 (10.2%)<br>60+: 1 (2.0%)<br><br>Ethnicity unweighted (N=49) - n (%)<br>Non-Hispanic white: 20 (40.8%)<br>Black: 3 (6.1%)<br>Hispanic: 1 (2.0%)<br>Not stated: 25 (51.1%)	No information available on the e-cigarette used nor the exposure circumstances	Affected body part (2008-2017) (N=49) - n (%)<br>Head: 2 (4.1%)<br>Shoulder: 1 (2.0%)<br>Lower arm: 3 (6.1%)<br>Hand: 8 (16.3%)<br>Lower abdomen: 4 (8.2%)<br>Upper leg: 29 (59.2%)<br>Lower leg: 2 (4.1%)<br><br>Events (n)<br>2007-2012: 0<br>2013: 1<br>2014: 0<br>2015: 5<br>2016: 25<br>2017: 18	Required hospitalisation: 13/49 (26.5%)	Not reported	High methodological quality<br><br>Small study size<br><br>Conflicts of interest<br>Not reported<br><br>Funding<br>Not reported
Corey et al., 2018<br><br>US<br><br>2016<br><br>National Electronic Injury Surveillance System (NEISS)	Unweighted N=26<br><br>Sex unweighted (N=26) - n (%)<br>Male: 25 (96.2%)<br>Female: 1 (3.8%)<br><br>Age unweighted (N=26) - n (%) years<br><18: 3 (11.3%)<br>18-24: 4 (15.4%)<br>25-54: 18 (69.2%)<br>≥55: 1 (3.8%)	Device batteries in pocket: 20/26 (76.9%)<br><br>Details of e-cigarette devices used were not reported	Burn type (N=26) - unweighted n (%)<br>Thermal burn: 22 (84.6%)<br>Chemical burn: 3 (11.5%)<br>Electric burn: 1 (3.4%)<br><br>Affected body part (N=26) - unweighted n (%)<br>Upper leg/lower trunk: 19 (73.1%)<br>Hand/lower arm: 5 (19.2%)<br>Other body parts: 2 (7.7%)	Unweighted (N=26) - n (%)<br>Treated/discharged: 13 (50.0%)<br>Hospitalised: 12 (46.2%)<br>Other: 1 (3.8%)	Not reported	High methodological quality<br><br>Very small study size<br><br>Conflicts of interest<br>None declared<br><br>Funding<br>Supported by Center for Tobacco Products, U.S. Food and Drug Administration
	National estimate: N=1007<br><br>Sex national estimate (N=1007) - n (%; 95% CI)<br>Male: 992 (98.5%; 95.1-100.0)<br>Female: 15 (1.5%; 0.0-4.9)<br><br>Age national estimate (N=1007) - n (%; 95% CI)<br><18: 190 (18.9%; 12.2-25.6)<br>18-24: 109 (10.8%; 0.0-24.8)<br>25-54: 693 (68.8%; 58.7-78.9)<br>≥55: 15 (1.5%; 0.0-5.1)	Not reported	Burn type (N=1007) - national estimate n (%; 95% CI)<br>Thermal burn: 809 (80.3%; 53.2-100.0)<br>Chemical burn: 134 (13.3%; 0.0-38.3)<br>Electric burn: 64 (6.4%; 0.0-19.9)<br><br>Affected body part (N=1007) - national estimate n (%; 95% CI)<br>Upper leg/lower trunk: 778 (77.3%; 60.4-94.2)<br>Hand/lower arm: 198 (19.7%; 2.0-373)<br>Other body parts: 31 (3.1%; 0.0-7.3)	National estimate (N=1007) - n (%; 95% CI)<br>Treated/discharged: 626 (62.2%; 28.9-95.5)<br>Hospitalised: 278 (27.6%; 2.6-52.5)<br>Other: 103 (10.2%; 0.0-34.7)	Not reported	
	National estimate N=1,866	Not reported	National estimate - n	Not reported	Not reported	

Study details (author, year, location, time frame, data source)	Demographic characteristics	Circumstance of injury	Presentation or details of injuries	Treatment	Outcome and recovery	Quality assessment, study size, conflict of interest, funding
	Average per year - national estimate<br>N=835		2007 - 2012: 0<br>2013: 25<br>2014: 0<br>2015: 171<br>2016: 944<br>2017: 726			
Rossheim et al., 2018<br><br>US<br><br>2015-2017<br><br>US Consumer Product Safety Commission's (CPSC) National Electronic Injury Surveillance System (NEISS)	Unweighted N=52<br><br>National estimate - n (95% CI)<br>N=2,035 (1107-2964)<br><br>Sex national estimate - % (95% CI)<br>Male: 94% (85-100)<br><br>Age national estimate - years (95% CI)<br>Median: 26 (22-30)<br><br>Ethnicity national estimate - % (95% CI)<br>White: 87% (72-100)	Not reported	Burn location - national estimate % (95% CI)<br>Burns: 97% (93-100)<br>Upper leg: 61% (45-77)<br>Hand/fingers: 25% (9-42)	National estimate - % (95% CI)<br>Treated/released same visit: 69% (47-91)<br>Admitted: ~26% (5-47)<br>Left without being seen: 5% (0-15)	Not reported	High methodological quality<br><br>Conflicts of interest<br>None declared<br><br>Funding<br>Supported by the National Institute on Drug Abuse of the National Institutes of Health
Saxena et al., 2018<br><br>US<br><br>(1) 2009-2016<br>(2) 2009-2017<br><br>(1) National Fire Data Center<br>(2) Blog reports (Ecigone Blog)	Total cases N=636<br>(1) 195<br>(2) 243<br><br>No demographic information reported	Not reported	Not reported	Not reported	Not reported	Low methodological quality<br><br>Conflicts of interest<br>Not reported<br><br>Funding<br>Not reported

Percentages and p-values are presented as reported in original studies.
CI = confidence interval; CPSC = Consumer Product Safety Commission; NEISS = National Electronic Injury Surveillance System; NPDS = National Poison Data System; US = United States.

Table 6.2. Study details: burns and injuries – case reports and case series

Study details (author, year, location, [time frame], data source)	Demographics and medical history	Exposure (location of device, circumstance)	Presentation	Treatment	Outcome	Quality assessment, conflicts of interest, funding
Case series and burn centre reports						
Boissiere et al., 2020<br><br>France<br><br>2014-2019<br><br>Montpellier University Hospital Burn Centre	N=16<br><br>Male: 16/16 (100%)<br><br>Age mean: 41 years	Exposure (N=16) - n (%)<br>Device or battery in pocket: 16 (100%)<br>One battery in pocket possibly in contact with other objects: 9 (56%)<br>Presence of flame: 16 (100%)<br>Overheating before the fire: 8 (50%)	Second or third-degree burns: 16/16 (100%)<br><br>Average TBSA: 5% burned<br>Affected body area: buttocks, pelvis, genitals and/or thigh areas	Treatment (N=16) - n (%)<br>Hospitalisation: 6 (38%)<br>Surgery: 6 (38%)	Average healing length<br>46.25 days	Moderate methodological quality<br><br>Conflicts of interest<br>None declared<br><br>Funding<br>Not reported
Claes et al., 2020<br><br>Belgium<br><br>No time frame reported<br><br>Ghent Burn Center	Case 1<br>Male<br>45 years<br><br>Case 2<br>Male<br>47 years	Case 1<br>Spontaneous ignition of device in jeans pocket<br><br>Case 2<br>Spare battery went into thermal runaway in pocket	Case 1<br>Superficial partial and deep partial thickness burn on his right upper leg - 9% TBSA<br><br>Case 2<br>Superficial partial thickness, deep partial thickness and full thickness burn to upper leg and superficial burn to his fingers - 9% TBSA	Case 1<br>Cleaned and covered with allograft<br><br>Case 2<br>Cleaned and covered with allograft	Case 1<br>Complete wound healing 35 days after the initial injury. Scarring<br><br>Case 2<br>Complete wound healing 61 days after the initial injury. Scarring	High methodological quality<br><br>Conflicts of interest<br>None declared<br><br>Funding<br>No specific funding
Isakov et al., 2020<br><br>US<br><br>No time frame reported<br><br>Hospital record	Male<br>22 years	Device exploded during use	Lower lip laceration, multiple displaced teeth, and fractured maxilla	Lacerations repaired and dentoalveolar splint placed	Not reported	Moderate methodological quality<br><br>Conflicts of interest<br>None declared<br><br>Funding<br>None received
Gibson et al., 2019<br><br>US<br><br>2012-2016<br><br>Hospital electronic medical record (EMR) system-Oregon Clinic and Legacy Emmanuel Hospital	N=14<br><br>Male: 13/14 (92.9%)<br>Female: 1/14 (7.1%)<br><br>Age range: 16-49 years	Exposure (N=14) - n (%)<br>Device or battery exploded in pocket: 12 (85.7%)<br>Device exploded in hand: 2 (14.3%)<br><br>Details of device (N=14) - n (%)<br>Loose battery: 7 (50.0%)<br>E-cigarette device: 6 (42.9%)<br>Vape pen: 1 (7.1%)	Location of burn injury (N=14) - n (%)<br>Burns to thighs only: 6 (42.9%)<br>Burns to thigh and hand: 6 (42.9%)<br>Burn to hand: 1 (7.1%)<br>Burn to hand and lip: 1 (7.1%)<br><br>Degree of burn injury (N=14) - n (%)<br>Full thickness burns: 3 (21.4%)<br>Partial thickness burns: 10 (71.4%)<br>Mixed partial/full thickness burns: 1 (7.1%)<br><br>TBSA range: 1%-6%	3/14 (21.4%) of patients required excision and autografting	Average recovery time was 24.5 days<br><br>2/14 (14.3%) lost to follow-up	Moderate methodological quality<br><br>Conflicts of interest<br>None declared<br><br>Funding<br>None received

Study details (author, year, location, [time frame], data source)	Demographics and medical history	Exposure (location of device, circumstance)	Presentation	Treatment	Outcome	Quality assessment, conflicts of interest, funding
Quiroga et al., 2019<br><br>US<br><br>2018<br><br>Johns Hopkins Bayview Burn Center	N=6<br><br>Male: 6/6 (100%)<br><br>Age range: 27-46 years	Exposure - n (%)<br>Device or battery exploded in pants pocket: 6/6 (100%)	Side and degree of burn injury - n (%)<br>Burns to thigh: 3/6 (50.0%)<br>Burns to thigh and hand: 2/6 (33.3%)<br>Burns to thigh, knee and hand: 1/6 (16.7%)<br>Superficial partial thickness burn: 5/6 (83.3%)<br>Intermediate burn: 1/6 (16.7%)<br><br>TBSA range: 2%-6%	Treatment - n (%)<br>Tangential excision and skin grafting: 1/6 (16.7%)<br>Complex wound care: 5/6 (83.3%)	Discharged within a week: 5/6 (83.3%)<br><br>Stayed for 8 days: 1/6 (16.7%)	Moderate methodological quality<br><br>Conflicts of interest<br>None declared<br><br>Funding<br>Not reported
Hickey et al., 2018<br><br>US<br><br>2015-2017<br><br>Massachusetts General Hospital Burn Center	N=14<br><br>Male: 13/14 (93%)<br>Female: 1/14 (7%)<br><br>Age - mean (SD): 28.6 (8.6) years<br><br>Age range: 19-50 years	Location of device - n (%)<br>Pant pocket: 12/14 (86%)<br>Hand: 1/14 (7%)<br>Purse: 1/14 (7%)<br><br>Details of e-cigarettes used were not reported	Side and degrees of burn injury - n (%)<br>Isolated upper extremity burns: 1/14 (7%)<br>Multiple burns at thigh, buttock, genitalia and/ or hand: 4/14 (29%)<br>Second- and third-degree burns: 8/14 (57%)<br>Deep second-degree burns: 4/14 (29%)<br>Superficial second-degree burn: 2/14 (14%)<br>Average TBSA: 4.7% (SD=2.4%)	Treatment - n (%)<br>Admitted: 12/14 (86%)<br>Discharged, local wound care only: 1/14 (7%)<br>Refused admission: 1/14 (7%)<br>Split-thickness skin graft (STSG): 8/14 (57%)<br>Local wound care only: 4/14 (29%)<br>Xenograft and local wound care: 1/14 (7%)<br>Enzymatic debridement and wound care: 1/14 (7%)<br>Lost to follow-up: 1/14 (7%)	Average hospital stay length<br>6.6 days (SD=4.7)<br>Range: 0-15 days<br><br>Time to 95% wound closure<br>18.4 days (SD=10.8)<br>Range: 8-40 days	High methodological quality<br><br>Conflicts of interest<br>Not reported<br><br>Funding<br>Not reported
Maraqa et al., 2018<br><br>US<br><br>No time frame reported<br><br>Trauma Services Hurley Medical Center/Michigan State University, College of Human Medicine, Flint	N=8<br><br>Male: 8/8 (100%)<br><br>Age range: 17-47 years	Exposure - n (%)<br>Device or battery exploded in pants pocket: 7/8 (87.5%)<br>Device exploded in their breast pocket: 1/8 (12.5%)	Side and degrees of burn injury - n (%)<br>Burns to lower extremity: 7/8 (87.5%)<br>Burns to hand: 3/8 (37.5%)<br>Burns to scrotum/penis: 2/8 (25%)<br>Burns to chest: 1/8 (12.5%)<br>Partial thickness burns: 5/8 (62.5%)<br>Mixed partial and full: 3/8 (37.5%)<br>TBSA range: 4%-16%	Skin grafting: 2/8 (25.0%)	Time to discharge<br>Few hours to 6 days	Moderate methodological quality<br><br>Conflicts of interest<br>Not reported<br><br>Funding<br>Not reported
Harshman et al., 2017<br><br>US<br><br>No time frame reported<br><br>Burn centre	Case 1<br>Male<br>31 years<br><br>Case 2<br>Male<br>36 years	Case 1<br>Spontaneous ignition of device in jeans pocket while driving<br><br>Case 2<br>Spare battery in pocket that spontaneous ignited	Case 1<br>Mixed partial thickness and full thickness flame burns to right anterolateral thigh, buttock, leg, and inner thigh. 10% TBSA<br><br>Case 2<br>Deep partial and full thickness burns to thigh and superficial partial thickness burns to hand. 3% TBSA. Part of the battery case embedded in thigh	Case 1<br>Irrigated and dressed<br><br>Case 2<br>Irrigated and dressed. Skin infection two days after injury treated with antibiotics. Skin allograft	Case 1<br>Full recovery within 2 months<br><br>Case 2<br>In hospital for 12 days, returned to full function within 2 months	Moderate methodological quality<br><br>Conflicts of interest<br>None declared<br><br>Funding<br>Not reported

Study details (author, year, location, [time frame], data source)	Demographics and medical history	Exposure (location of device, circumstance)	Presentation	Treatment	Outcome	Quality assessment, conflicts of interest, funding
Serror et al., 2017<br><br>France<br><br>2016-2017<br><br>Saint Louis Hospital Burn Center, Paris	N=10<br><br>Male: 10/10 (100%)<br><br>Age - mean (range): 39 (26-55) years	Exposure - n (%)<br>Exploded in pocket: 8/10 (80%)<br>Exploded in hands: 2/10 (20%)	Affected body parts - n (%)<br>Thigh: 8/10 (80%)<br>Hands: 5/10 (50%)<br>Partial thickness: 5/10 (50%)<br>Full thickness: 3/10 (30%)<br>Mixed partial and full thickness: 2/10 (20%)<br><br>Average TBSA: 3% (0.5%-5%)	Treatment - n (%)<br>Non-operative management: 7/10 (70%)<br>Surgery: 3/10 (30%)	Spontaneously healed within 21 days: 7/10 (70%)	High methodological quality<br><br>Conflicts of interest<br>None declared<br><br>Funding<br>Not reported
Smith et al., 2017<br><br>US<br><br>2015-2016<br><br>Single burn centre	N=10<br><br>Male: 10/10 (100%)<br><br>Age range: 20-47 years	Exposure - n (%)<br>Device/battery exploded in pants pocket: 7/10 (70%)<br>Device exploded in hand: 1/10 (10%)<br>Device exploded while driving tractor trailer and fell into lap: 1/10 (10%)<br>Pouring liquid nicotine then engulfed in flames: 1/10 (10%)	Affected body part - n (%)<br>Thigh, hand, buttock: 1/10 (10%)<br>Hand, foot, thigh: 1/10 (10%)<br>Face, trunk, arms, hands, ankles, feet: 1/10 (10%)<br>Fingers, thigh, knee: 1/10 (10%)<br>Thigh, fingers: 1/10 (10%)<br>Hand, fingers: 1/10 (10%)<br>Thigh, hand: 3/10 (30%)<br>Thigh: 1/10 (10%)<br>Average TBSA: 4.2%	Treatment - n (%)<br>Skin graft: 8/10 (80%)<br>Not reported: 2/10 (20%)	Average length of hospital stay<br>4.9 days<br>Range: 0-11 days<br><br>Return to work - n (%)<br>3 weeks: 1/10 (10%)<br>4 weeks: 3/10 (30%)<br>5 weeks: 1/10 (10%)<br>No time taken off: 3/10 (30%)<br>Unknown: 2/10 (20%)	Moderate methodological quality<br><br>Conflicts of interest<br>None declared<br><br>Funding<br>Not reported
Case reports						
Beining et al., 2020<br><br>US<br><br>District Six Medical Examiner's Office	Male<br>38 years	Modified device exploded during use	Burns covering 80% of body and wound to face/mouth<br><br>Projectile wound to the head present to face	N/A	Death	Moderate methodological quality<br><br>Conflicts of interest<br>Not reported<br><br>Funding<br>Not reported
Hagarty & Luo, 2020<br><br>US<br><br>University of Illinois College of Medicine at Rockford, OSF St Anthony Medical Centre	Female<br>30 years<br><br>Recent tonsillar and ear infection	Device unable to be identified by emergency responders<br><br>Modified device exploded upon activation	Superficial partial thickness burn and a full thickness complex laceration of the lower lip<br><br>Tongue, hand and finger lacerations, teeth extensively broken, comminuted spinal fracture and evidence of left vertebral artery dissection	Fracture stabilised<br><br>Artery dissection treated with aspirin and low-molecular-weight heparin<br><br>Soft tissue injuries reconstructed after extensive irrigation	Discharged, healing well	Moderate methodological quality<br><br>Conflicts of interest<br>None declared<br><br>Funding<br>Not reported
Sedaghat & Morgan, 2020<br><br>US<br><br>Hospital record	Male<br>16 years	Inadvertent aspiration of the cartridge cap	Foreign body in the right main stem bronchus	Foreign body removed	Not reported	Low methodological quality<br><br>Conflicts of interest<br>Not reported<br><br>Funding<br>Not reported

Study details (author, year, location, [time frame], data source)	Demographics and medical history	Exposure (location of device, circumstance)	Presentation	Treatment	Outcome	Quality assessment, conflicts of interest, funding
Ashburn et al., 2019<br><br>US<br><br>Level 1 trauma/burn centre	Male<br>28 years	Device exploded during use	Two fractured teeth, tongue laceration, stellate upper lip laceration and foreign bodies in lower lip	Lacerations repaired	Discharged	Low methodological quality<br><br>Conflicts of interest<br>Not reported<br><br>Funding<br>Not reported
Katz & Russell, 2019<br><br>US<br><br>Unknown data source	Male<br>17 years	Device exploded during use	Puncture to the chin, extensive lacerations to mouth, multiple disrupted teeth and mandibular fracture	Internal fixation of the fracture, dental extraction, and debridement of devitalised tissue	6-week follow-up<br>Recovered well	Moderate methodological quality<br><br>Conflicts of interest<br>Not reported<br><br>Funding<br>Not reported
Michael et al., 2019<br><br>US<br><br>Hospital burn unit	Male<br>40 years	Spontaneous combustion of device in pant pocket	Severe burns on the left posterior thigh	Split thickness autograft and additional use of an allograft matrix 4 days after injury	Graft incorporated<br><br>One-month post-injury<br>Intermittent pain, irritation, and a mildly antalgic gait. Loss of terminal extension of the knee joint. Clinical evidence of iliotibial band tightness<br><br>The cosmetic appearance of his graft and donor site is of great emotional concern to the patient	Moderate methodological quality<br><br>Conflicts of interest<br>None declared<br><br>Funding<br>Not reported
Sangani et al., 2019<br><br>US<br><br>Hospital record	Male<br>40 years<br><br>Patient denied any medical history	Combustion of device spare batteries in pant pocket	Superficial and deep partial thickness burns to thigh, 9% TBSA	Wound irrigated	Not reported	Low methodological quality<br><br>Conflicts of interest<br>Not reported<br><br>Funding<br>Not reported
Ackley et al., 2018<br><br>US<br><br>Hospital record	Male<br>17 years	Device exploded when about to take a puff	Burnt left thumb with sensory loss, decreased motor control, heavy bleeding	Immediate irrigation, debridement, and a left-hand carpal tunnel release	Post-operative day 2<br>Discharged<br><br>Post-operative day 8<br>Blackened thumb without capillary refill or sensation and limited motor function. Required 6 additional operative procedures	Moderate methodological quality<br><br>Conflicts of interest<br>Not reported<br><br>Funding<br>Not reported

Study details (author, year, location, [time frame], data source)	Demographics and medical history	Exposure (location of device, circumstance)	Presentation	Treatment	Outcome	Quality assessment, conflicts of interest, funding
Chi et al., 2018<br><br>US<br><br>Emergency Dental Clinic, Medical University of South Carolina	Male<br>20 years	Device exploded during use	Burns and lacerations of the upper and lower lips, dislodgement of teeth	Lacerations sutured, teeth extracted. Antibiotic and pain medication prescribed	Lost to follow-up	Moderate methodological quality<br><br><u>Conflicts of interest</u><br>None declared<br><br><u>Funding</u><br>None received
Satteson et al., 2018<br><br>US<br><br>Emergency Department, Trauma Centre, Wake Forest University of Medicine	Male<br>35 years	Device (Dark Horse atomiser with a SMPL Mec Mod battery) rapidly heated and suddenly exploded after battery was changed	Significant for deep partial and full thickness burns to thumb and embedded foreign body	Surgery and debridement of devitalised tissue and carpal tunnel release	<u>15 months after initial injury</u><br>Thumb interphalangeal joint is fixed in 30° of flexion with no ability to actively or passively flex or extend	High methodological quality<br><br><u>Conflicts of interest</u><br>None declared<br><br><u>Funding</u><br>None received
Anderson et al., 2017<br><br>US<br><br>University of Kentucky Emergency Department	Female<br>30 years	Device exploded during use	2% TBSA burns to face, forearm, and thigh and bilateral corneal burns	Treated with erythromycin to corneal burns, Silvadene to the extremities, and bacitracin to the face	Discharged, healing well	Low methodological quality<br><br><u>Conflicts of interest</u><br>Not reported<br><br><u>Funding</u><br>Not reported

Percentages and p-values are presented as reported in original studies.
EMR = electronic medical record; SD = standard deviation; STSG = split-thickness skin graft; TBSA = total body surface area; US = United States.

7. Poisonings

Table 7.1. Study details: poisoning – surveillance reports

Study details (author, year, country, time frame, data source)	Demographics	Exposure (e-liquid description, route of administration, cause of exposure)	Presentation and symptoms	Treatment	Outcome	Quality assessment, study size, conflict of interest, funding
Chang et al., 2020<br><br>US<br><br>2013-2017<br><br>National Center for Injury Prevention under the NEISS All Injury Program (NEISS-AIP)	Unweighted sample<br>n=39<br><br>Gender (N=39) - n (%)<br>Male: 14 (35.9%)<br>Female: 25 (64.1%)<br><br>Age (N=39) - n (%)<br>5-11 years: 4 (10.3%)<br>12-17 years: 10 (25.6%)<br>18-24 years: 10 (25.6%)<br>≥25 years: 15 (38.5%)	All cases aged 5-11 years experienced unintentional liquid ingestions or chemical exposure	Symptoms (N=39) - n (%)<br>Cardiovascular: 11 (28.2%)<br>Allergic reaction: 7 (17.9%)<br>Other: 7 (17.9%)<br>Unspecified: 6 (15.4%)<br>Gastroenteric: 5 (12.8%)<br>Chemical exposure: 3 (7.7%)	Treatment (N=39) - n (%)<br>Treated and released: 33 (84.6%)<br>Left without being seen: 3 (7.7%)<br>Treated and admitted to a hospital: 3 (7.7%)	Not reported	High methodological quality<br><br>Conflicts of interest<br>Not reported. No financial disclosures<br><br>Funding<br>Center for Tobacco Products, U.S. Food and Drug Administration
	National estimates (weighted)<br>n=2,718<br><br>Gender - n (%; 95% CI)<br>Male: 1,410 (51.9%; 29.1-74.6)<br>Female: 1,309 (48.1%; 25.4-70.9)<br><br>Age - n (%; 95% CI)<br>5-11 years: 127 (4.7%; 0.0-10.7)<br>12-17 years: 449 (16.5%; 0.0-36.1)<br>18-24 years: 737 (27.1%; 11.1-43.2)<br>≥25 years: 1,405 (51.7%; 30.8-72.5)	Not reported	National estimates (weighted) - n (%; 95% CI)<br>Cardiovascular: 808 (29.7%; 10.8-48.6)<br>Allergic reaction: 700 (25.7%; 2.7-48.7)<br>Other: 587 (21.6%; 1.5-41.7)<br>Unspecified: 308 (11.3%; 0.0-28.6)<br>Gastroenteric: 249 (9.2%; 0.0-19.3)<br>Chemical exposure: 68 (2.5%; 0.18-4.8)	National estimates (weighted) - n (%; 95% CI)<br>Treated and released: 2,082 (76.6%; 54.3-98.9)<br>Left without being seen: 423 (15.9%; 0.0-40.1)<br>Treated and admitted to a hospital: 203 (7.5%; 0.0-23.7)	Not reported	
McFaull et al., 2020<br><br>Canada<br><br>2011-2019<br><br>The electronic Canadian Hospitals Injury Reporting and Prevention Program network	Total cases n=55<br><br>Age (N=55) - n (%)<br>0-4 years: 36 (65.5%)<br>5-14 years: 12 (21.8%)<br>15-19 years: 7 (12.7%)<br>20-29 years: 0 (0%)<br>30-49 years: 0 (0%)	Route of administration - n (%)<br>Unintentional ingestion of vaping solution: 36/55 (65.5%)	Not reported	Not reported	Not reported	Moderate methodological quality<br><br>Conflicts of interest<br>None declared<br><br>Funding<br>Not reported

Study details (author, year, country, time frame, data source)	Demographics	Exposure (e-liquid description, route of administration, cause of exposure)	Presentation and symptoms	Treatment	Outcome	Quality assessment, study size, conflict of interest, funding
Obertova et al., 2020<br><br>Czech Republic<br><br>2012-2018<br><br>Toxicological Information Centre (TIC)<br><br>*The Centre recorded 148 phone calls in total (three animal exposures and 145 human)	Total human cases n=145*<br><br>Gender (N=145) - n (%)<br>Male: 95 (65.5%)<br>Female: 48 (33.1%)<br>Unknown: 2 (1.4%)<br><br>Age (%)<br>≤2 years: 37%<br>2-18 years: 25%<br>18+ years: 35%<br>Unknown age: 1%	Volume<br>Range (mL): 10-30<br><br>Nicotine concentration<br>Range (mg/mL): 1-24<br><br>Dosage (N=148)* - n (%)<br>Severe/lethal: 6 (4%)<br>Toxic: 53 (36%)<br>Low-to-moderate: 35 (24%)<br>Unknown: 54 (36%)<br><br>Cause of exposure (N=148)* - n (%)<br>Accidental: 110 (74%)<br>Incorrect application: 10 (7%)<br>Abuse: 6 (4%)<br>Suicide attempt: 6 (4%)<br>Other/unknown reasons: 16 (11%)<br><br>Route of administration (%)<br>Ingestion: 67%<br>Licking: 14%<br>Suspected ingestion: 7%<br>Inhalation: 6%<br>Ocular: 4%<br>Intravenous: 2%	Symptoms - n (%)<br>Asymptomatic: 82/148 (55%)<br>Symptomatic (60/148; 41%) post-exposure:<br><1 hour: 42/60 (70%)<br>1-4 hours: 14/60 (24%)<br>>4 hours: 4/60 (6%)<br>Symptoms not stated: 6/148 (4%)<br><br>Symptoms included: nausea, feeling of burning in the mouth and throat, salivation, repeated vomiting, diarrhea, abdominal pain, tachycardia, tremor and respiratory irritation	Treatment - n (%)<br>Medical examination recommended: 115/148 (78%)<br>Hospitalisation/medical observation: 106/148 (72%)<br>Home observation: 33/148 (22%)<br><br>Recommended treatment measures for hospitalised patients (N=106) - n (%)<br>Activated charcoal: 57 (54%)<br>Symptomatic treatment: 75 (70%)<br>Atropine: 2 (2%)<br>Gastric lavage: 1 (1%)<br>Not stated: 9 (9%)<br><br>In one 33-year-old patient with coma and general convulsions, intubation was performed, and benzodiazepines were applied	Prognosis (time of consultation) (N=148)* - n (%)<br>Good: 15 (10%)<br>Probably good: 62 (42%)<br>Uncertain: 65 (44%)<br>Unknown: 6 (4%)	High methodological quality<br><br>Conflicts of interest<br>None declared<br><br>Funding<br>First Faculty of Medicine, Charles University; Ministry of Health Czech Republic

Study details (author, year, country, time frame, data source)	Demographics	Exposure (e-liquid description, route of administration, cause of exposure)	Presentation and symptoms	Treatment	Outcome	Quality assessment, study size, conflict of interest, funding
Wang et al., 2020<br><br>US<br><br>2010-2018<br><br>National Poison Data System (NPDS)	Total cases n=17,358<br><br>Gender (N=17,358) - n (%)<br>Male: 9,631 (55.5%)<br>Female: 7,648 (44.1%)<br>Unknown: 79 (0.5%)<br><br>Age (N=17,358) - n (%)<br><5 years: 11,250 (64.8%)<br>5-11 years: 525 (3.0%)<br>12-17 years: 596 (3.4%)<br>18-24 years: 1,443 (8.3%)<br>25+ years: 2,667 (15.4%)<br>Missing: 877 (5.1%)	Quantity (mL) of e-liquid by medical outcome - mean (min-max)<br>No effect (n=37): 7.5mL (0.2-60.0)<br>Minor (n=22): 13.1mL (0.6-60.0)<br>Moderate (n=5): 56.2mL (1.0-200.0)<br><br>Quantity (mg) of nicotine by medical outcome - mean (min-max)<br>No effect (n=11): 19.3 (3.0-96.0)<br>Minor (n=11): 49.7 (6.0-240.0)<br><br>Route of administration (N=17,358) - n (%)<br>Ingestion: 13,456 (77.5%)<br>Dermal: 2,258 (13.0%)<br>Inhalation/nasal: 1,807 (10.4%)<br>Ocular: 1,232 (7.1%)<br>Other: 60 (0.3%)<br>Unknown: 31 (0.2%)<br><br>Number of events by year<br>2010: 57<br>2011: 237<br>2012: 415<br>2013: 1,435<br>2014: 3,742<br>2015: 3,500<br>2016: 2,751<br>2017: 2,320<br>2018: 2,901	Symptoms - n (%)<br>Vomiting: 2,297 (25.4%)<br>Nausea: 1,070 (11.8%)<br>Ocular irritation/pain: 1,022 (11.3%)<br>Red eye conjunctivitis: 494 (5.5%)<br>Dizziness/vertigo: 463 (5.1%)	Level of care at health care facility (N=17,358) - n (%)<br>Admitted (critical unit): 99 (0.6%)<br>Admitted (noncritical unit): 130 (0.8%)<br>Admitted (psychiatric facility): 54 (0.3%)<br>Lost to follow-up/left: 1,079 (6.2%)<br>Treated, evaluated and released: 4,752 (27.4%)<br>Refused referral/no show: 679 (3.9%)<br>Not referred to facility: 10,565 (60.9%)	Medical outcome (N=17,358) - n (%)<br>Minor: 3,918 (22.6%)<br>Moderate: 578 (3.3%)<br>Major: 24 (0.1%)<br>Death: 2 (0.01%)<br>No effect: 6,068 (35.0%)<br>Missing: 6,768 (39.0%)	High methodological quality<br><br>Conflicts of interest<br>None declared<br><br>Funding<br>Not reported
Chang & Rostron, 2019<br><br>US<br><br>2018<br><br>National Emergency Injury Surveillance System (NEISS)	Unweighted sample n=26<br><br>Gender - n (%)<br>Male: 15/26 (58%)<br>Female: 11/26 (42%)<br><br>Age - n (%)<br><2 years: 17/26 (65%)<br>2-4 years: 9/26 (45%)	Nicotine concentration, unweighted sample - n<br>0.6mg: 2<br><br>E-liquid volume, unweighted sample - n<br>60mL: 2<br>10mL: 1<br><br>Route of exposure - unweighted sample - n (%)<br>Ingestion: 25/26 (96%)<br>Other/not stated: 1/26 (4%)<br><br>Ingested cotton filters: 3/26 (12%)	Symptoms, unweighted sample - n<br>Vomiting: 3<br>Emesis: 2	Treatment, unweighted sample - n (%)<br>Admitted to hospital: 2/26 (8%)<br>Treated and released: 23/26 (88%)<br>Left without being seen: 1/26 (4%)	Not reported	High methodological quality<br><br>Conflicts of interest<br>None declared<br><br>Funding<br>Center for Tobacco Products, U.S. Food and Drug Administration

Study details (author, year, country, time frame, data source)	Demographics	Exposure (e-liquid description, route of administration, cause of exposure)	Presentation and symptoms	Treatment	Outcome	Quality assessment, study size, conflict of interest, funding
	National estimates n=885<br><br><u>Gender (national estimates) (N=885) - n (%)</u><br>Male: 267 (30.1%)<br>Female: 618 (69.9%)<br><br><u>Age (national estimates) (N=885) - n (%)</u><br><2 years: 526 (59.4%)<br>2-4 years: 359 (40.6%)	<u>Route of exposure (national estimates) (N=885) - n (%)</u><br>Ingestion: 880 (99.4%)<br>Other/not stated: 5 (0.56%)	Not reported	<u>Treatment (national estimates) (N=885) - n (%)</u><br>Treated and admitted to a hospital: 10 (1.1%)<br>Treated and released: 797 (90.0%)<br>Left without being seen: 78 (8.9%)	Not reported	
Chang et al., 2019<br><br>US<br><br>2013-2017<br><br>National Emergency Injury Surveillance System (NEISS)	Unweighted sample n=116<br><br><u>Gender (N=116) - n (%)</u><br>Male: 67 (57.8%)<br>Female: 49 (42.2%)<br><br><u>Age (N=116) - n (%)</u><br><2 years: 62 (53.4%)<br>2-4 years: 54 (46.6%)	<u>Nicotine concentration, unweighted - mean (min-max)</u><br>mg (n=6): 3 (1.8-100)<br><br><u>E-liquid volume, unweighted - mean (min-max)</u><br>mL (n = 19): 16.8 (0.2-118.3)<br>bottle (n = 26): 0.875 (0.5-1.0)<br><br><u>Route of administration, unweighted (N=116) - n (%)</u><br>Ingestion: 111 (95.7%)<br>Dermal: 3 (2.6%)<br>Ingestion + ocular: 1 (0.9%)<br>Unknown: 1 (0.9%)	<u>Symptoms, unweighted (N=11) - n (%)</u><br>Vomiting, nausea, emesis: 7 (63.6%)<br>Crying, eye redness: 2 (18.2%)<br>Cough: 1 (9.1%)<br>Sleepy: 1 (9.1%)<br>Oral cyanosis/unresponsive: 1 (9.1%)	<u>Treatment, unweighted (N=116) - n (%)</u><br>Treated and admitted to a hospital: 11 (9.5%)<br>Treated and released: 103 (88.8%)<br>Left without being seen: 2 (1.7%)	Not reported	High methodological quality<br><br><u>Conflicts of interest</u><br>None declared<br><br><u>Funding</u><br>Center for Tobacco Products, U.S. Food and Drug Administration
	National estimates n=4,745<br><br><u>Gender (N=4,745) - n (%)</u><br>Male: 2,574 (54.3%)<br>Female: 2,171 (45.7%)<br><br><u>Age (N=4,745) - n (%)</u><br><2 years: 2,667 (56.2%)<br>2-4 years: 2,078 (43.8%)	<u>Route of administration - national estimates (N=4,745) - n (%)</u><br>Ingestion: 4,597 (96.9%)<br>Dermal: 858 (2.6%)<br>Ingestion + ocular: 6 (0.12%)<br>Unknown: 100 (2.1%)	Not reported	<u>National estimates (N=4,745) - n (%)</u><br>Treated and admitted to a hospital: 194 (4.1%)<br>Treated and released: 4,530 (95.5%)<br>Left without being seen: 21 (0.43%)	Not reported	

Study details (author, year, country, time frame, data source)	Demographics	Exposure (e-liquid description, route of administration, cause of exposure)	Presentation and symptoms	Treatment	Outcome	Quality assessment, study size, conflict of interest, funding
Choi et al., 2019<br><br>Canada<br><br>2012-2017<br><br>The British Columbia Drug and Poison Information Centre (DPIC)	Total cases n=186<br><br>Gender (N=186) - n (%)<br>Male: 108 (58.1%)<br>Female: 76 (40.9%)<br>Unknown: 2 (1.1%)<br><br>Age (N=186) - n (%)<br>≤4 years: 81 (43.5%)<br>5-14 years: 7 (3.8%)<br>15-19 years: 18 (9.7%)<br>20-24 years: 7 (3.8%)<br>≥25 years: 31 (16.7%)<br>Not recorded: 42 (22.6%)	Nicotine concentration (N=97) - n (%)<br>0mg/mL: 4 (4.1%)<br>0.1-5mg/mL: 18 (18.6%)<br>6-17mg/mL: 53 (54.6%)<br>18-23mg/mL: 15 (15.5%)<br>≥24mg/mL: 7 (7.2%)<br><br>Route of administration (N=186) - n (%)<br>Ingestion: 122 (65.6%)<br>Inhalation: 28 (15.0%)<br>Dermal: 22 (11.8%)<br>Ocular: 12 (6.4%)<br>Nasal: 1 (0.5%)<br>Vaginal: 1 (0.5%)<br><br>Cause of exposure (N=186) - n (%)<br>Accidental access: 85 (45.7%)<br>Usual e-cigarette use: 25 (13.4%)<br>E-cigarette malfunction: 17 (9.1%)<br>Other/not recorded: 16 (8.6%)<br>Spill: 13 (7.0%)<br>Mistaken identity: 12 (6.4%)<br>Handling device: 10 (5.4%)<br>Intentional inappropriate use: 7 (3.8%)<br>Making e-juice: 1 (0.5%)	Symptoms present (N=186) - n (%)<br>Yes: 87 (46.8%)<br>No: 70 (37.6%)<br>Not recorded: 29 (15.6%)<br><br>Symptoms (local) (N=186) - n (%)<br>Ocular: 11 (5.9%)<br>Oral/pharyngeal: 9 (4.8%)<br>Dermal: 5 (2.7%)<br>Respiratory: 3 (1.6%)<br>Vaginal: 1 (0.5%)<br><br>Symptoms (systemic) (N=186) - n (%)<br>Not typical for nicotine exposure: 45 (24.2%)<br>Typical for low nicotine exposure: 42 (22.6%)<br>Typical for high nicotine exposure: 2 (1.1%)	Care trajectory (N=186) - n (%)<br>Managed outside of health facility: 131 (70.4%)<br>Treated at health facility and released: 32 (17.2%)<br>Admitted (noncritical unit): 8 (4.3%)<br>Admitted (critical unit): 1 (0.5%)<br>Lost to follow-up: 14 (7.5%)	Not reported	High methodological quality<br><br>Conflicts of interest<br>None declared<br><br>Funding<br>Internal funding at the BC Centre for Disease Control
Hughes & Hendrickson, 2019<br><br>US<br><br>2014-2017<br><br>Oregon Poison Centre	Total cases n=265<br><br>Age (N=265) - n (%)<br>Children: 193 (72.8%)<br>Adults: 72 (27.2%)<br><br>Median (range): 2 years (0.5-65)	Route of administration: children (N=193) - n (%)<br>Ingestion: 108 (56%)<br>Exposures by handling device: 29 (15%)<br>Oral mucosal exposures: 23 (12%)<br>Dermal exposures: 23 (12%)<br>Inhalational exposures: 10 (5%)<br><br>Route of administration: adults (N=72) - n (%)<br>Ingestion exposures: 23 (32%)<br>Mucosal exposures: 15 (21%)<br>Ocular exposures: 14 (19%)<br>Dermal exposures: 13 (18%)<br>Inhalational exposure: 7 (10%)	Asymptomatic on initial call - n (%)<br>Children: 138/193 (72%)<br>Adults: 14/72 (19%)<br><br>Symptomatic on initial call - n (%)<br>Children: 55/193 (28%)<br>Adults: 58/72 (81%)	Not reported	Asymptomatic on follow-up call - n (%)<br>Children: 185/193 (96%)<br>Adults: 24/72 (33%)<br><br>Symptomatic on follow-up call - n (%)<br>Children: 8/193 (4%)<br>Adults: 13/72 (18%)<br><br>Unable to follow (children): 31/193 (16%)<br>Unable to follow (adults): 35/72 (49%)	Moderate methodological quality<br><br>Conflicts of interest<br>None declared<br><br>Funding<br>Not reported
Ang et al., 2018<br><br>UK<br><br>2008-2016	Total cases n=278<br><br>Gender (N=278) - n (%)<br>Male: 165 (59.4%)<br>Female: 112 (40.3%)<br>Unknown: 1 (0.3%)	Not reported	Symptoms - n (%)<br>Present: 63/278 (22.7%)<br><br>Most incidents were accidental and asymptomatic	Not reported	Not reported	Low methodological quality<br><br>Conflicts of interest<br>None declared

Study details (author, year, country, time frame, data source)	Demographics	Exposure (e-liquid description, route of administration, cause of exposure)	Presentation and symptoms	Treatment	Outcome	Quality assessment, study size, conflict of interest, funding
UK National Poisons Information Service (NPIS) Database	Age (N=278) - n (%)<br><4 years: 222 (79.9%)<br>5-16 years: 56 (20.1%)		Common clinical features (%)<br>Vomiting: 9.5%<br>Tachycardia: 2%<br>Dysesthesia: 1%<br>Irritation: 1%<br>Increased creatine kinase: 1%			Funding<br>No specific funding
Govindarajan et al., 2018<br><br>US<br><br>2012-2017<br><br>National Poison Data System (NPDS)	Total cases n=8,269<br><br>Gender - n (%)<br>Male: 4,572 (55.3%)<br><br>Age - n (%)<br><3 years: 6,940 (83.9%)<br><br>Median (IQR): 2.0 years (1.3 - 2.0)	Route of administration - n (%)<br>Ingestion: 7,649 (92.5%)	Clinical effects - n (%)<br>≥1 clinical effects: 2,032 (24.6%)<br>Severe clinical effects: 12<br>Coma: 4<br>Seizure: 4<br>Respiratory arrest: 3<br>Cardiac arrest: 1<br><br>Medical outcome - n (%)<br>Minor: 1,677 (20.3%)<br>Moderate: 132 (1.6%)<br>Major: 8 (0.1%)<br>Death: 1	Treatment - n (%)<br>Treated and released: 2,902 (35.1%)<br>Admitted: 115 (1.4%)	Not reported	Moderate methodological quality<br><br>Conflicts of interest<br>None declared<br><br>Funding<br>Centers for Disease Control and Prevention and the Child Injury Prevention Alliance stipend
Wylie et al., 2018<br><br>Australia<br><br>2009-2016<br><br>Australian Poisons Information Centres (PICs)	Total cases n=202<br><br>Age (N=202) - n (%)<br>Children: 76 (37.6%)<br>Adults and adolescents: 126 (62.4%)	Nicotine concentration of e-liquid - median (range)<br>20.2mg/mL (0.06-200mg/mL)<br><br>Route of administration - children<br>Uncapped vials, sucking the mouthpiece, drinking from separated liquid containers, inhaling the liquid, eating the cartridge, or having splashed liquid in their eyes<br><br>Route of administration, adults and adolescents - deliberate self-harm - n<br>Ingestion: 10<br>Injection: 2	12 had moderate symptoms, usually a gastrointestinal disturbance combined with sedation	Not reported	Not reported	Low methodological quality<br><br>Conflicts of interest<br>Consultancy fees from pharmaceutical company<br><br>Funding<br>Not reported

Study details (author, year, country, time frame, data source)	Demographics	Exposure (e-liquid description, route of administration, cause of exposure)	Presentation and symptoms	Treatment	Outcome	Quality assessment, study size, conflict of interest, funding
Vardavas et al., 2017<br><br>Europe (Sweden, Ireland, The Netherlands, Portugal, Austria, Slovakia, Lithuania and Hungary)<br><br>2012-2015<br><br>National Poisons Centers	Total incidents n=277<br><br>By country (N=277) - n (%)<br>Sweden: 121 (43.7%)<br>Netherlands: 78 (28.2%)<br>Ireland: 37 (13.4%)<br>Portugal: 25 (9.0%)<br>Austria: 8 (2.9%)<br>Slovakia: 5 (1.8%)<br>Lithuania: 2 (0.7%)<br>Hungary: 1 (0.4%)<br><br>Gender (N=233) - n (%)<br>Male: 118 (50.6%)<br>Female: 115 (49.4%)<br><br>Age (N=277) - n (%)<br>5 years: 92 (33.2%)<br>6-18 years: 27 (9.8%)<br>≥19 years: 158 (57.0%)	Cause of exposure (N=275) - n (%)<br>Unintentional: 196 (71.3%)<br>Intentional: 49 (17.8%)<br>Abuse: 15 (5.5%)<br>Misuse: 6 (2.2%)<br>Suspected suicide: 3 (1.1%)<br>Unknown: 6 (2.2%)<br><br>Route of administration (N=277) - n (%)<br>Ingestion: 187 (67.5%)<br>Respiratory/inhalation: 46 (16.6%)<br>Dermal: 25 (9.0%)<br>Ocular: 21 (7.6%)<br>Other: 6 (2.2%)	Symptoms (N=277) - n (%)<br>Vomiting: 56 (20.3%)<br>Dizziness: 40 (14.5%)<br>Nausea: 38 (13.8%)<br>Throat Conditions: 25 (9.1%)<br>Throat irritation: 9 (3.3%)<br>Burning throat: 5 (1.8%)<br>Oral mucosal: 8 (2.9%)<br>Salivation: 2 (0.7%)<br>Pharyngitis: 1 (0.4%)<br>Abdominal Conditions: 17 (6.2%)<br>Eye Conditions: 14 (5.0%)<br>Headache: 11 (4.0%)<br>Diarrhea: 8 (2.9%)<br>Breathing Conditions: 8 (2.9%)<br>Tremor: 4 (1.4%)<br>Other: 75 (27.3%)	Management of incident (N=237) - n (%)<br>Residence/on site: 166 (70.0%)<br>Hospital: 56 (23.6%)<br>Ambulance: 4 (1.7%)<br>Other/unknown: 11 (4.6%)	Medical outcome (N=208) - n (%)<br>Minor effect: 112 (53.8%)<br>Moderate effect: 13 (6.3%)<br>Major effect: 1 (0.5%)<br>No effect: 82 (39.4%)<br>Death: 0 (0.0%)	Moderate methodological quality<br><br>Conflicts of interest<br>None declared<br><br>Funding<br>EU Health Programme

Percentages and p-values are presented as reported in original studies.

BC = British Columbia; CI = confidence interval; DPIC = Drug and Poison Information Centre; EU = European Union; IQR = interquartile range; max = maximum; min = minimum; NEISS = National Electronic Injury Surveillance System; NEISS-AIP = National Electronic Injury Surveillance System All Injury Program; NPDS = National Poison Data System; NPIS = National Poisons Information Service; PIC = Poisons Information Centre; TIC = Toxicological Information Centre; UK = United Kingdom; US = United States.

Table 7.2. Study details: poisoning – case reports and case series

Study details (author, year, location, data source [time frame])	Demographics and medical history	Exposure (e-liquid description, route of administration, cause of exposure)	Presentation	Treatment	Outcome	Quality assessment, conflict of interest, funding
Case series						
Isakov et al., 2020<br><br>US<br><br>No time frame reported<br><br>Hospital record	Female<br>13 years<br><br>Not reported	Ingestion of a vape pen containing nicotine. Concern for a potentially lethal dose of nicotine if the vape pen were to leak<br><br>Ingestion<br><br>Accidental	The patient was taken for an exploratory laparotomy for removal of the pen<br><br>At the time of the laparotomy, the vape tip was in the colon	The colon was repaired primarily with colostomy closure and the patient tolerated the procedure well	She was subsequently discharged without complications	Low methodological quality<br><br>Conflicts of interest<br>None declared<br><br>Funding<br>None received
Park & Min, 2018<br><br>South Korea<br><br>Dec 2015-April 2016<br><br>Emergency department	Case 1<br>Male<br>27 years<br><br>Not reported<br><br>Case 2<br>Female<br>17 years<br><br>Not reported	Case 1<br>DIY Flavor Shack® 16mg/mL nicotine concentration and Halo® 18mg/mL nicotine concentration<br><br>Ingestion<br><br>Suicide attempt<br><br>Case 2<br>10mL e-cigarette liquid named 'Pure Nicotine®' with a nicotine concentration of 210mg/mL<br><br>Ingestion<br><br>Suicide attempt	Case 1<br>Showing seizure-like movements, cardiac arrest, comatose with fixed pupil size of 3mm<br><br>Case 2<br>Cardiac arrest, generalised tonic clonic movement for 5 minutes. Comatose with a fixed pupil size of 3mm	Case 1<br>Cardiac arrest care, targeted temperature management (TTM)<br><br>Case 2<br>Cardiac arrest care, targeted temperature management (TTM)	Case 1<br>24-hour after TTM: alert and aware<br>Day 13: discharged<br><br>Case 2<br>24-hour after TTM: alert and aware<br>Day 32: transferred to a rehabilitation facility	Moderate methodological quality<br><br>Conflicts of interest<br>None declared<br><br>Funding<br>Not reported
Case reports						
De Pieri et al., 2020<br><br>Italy<br><br>Emergency department	Female<br>4 years<br><br>Not reported	Approx. 10mL of 6mg/mL nicotine containing e-liquid<br><br>Ingestion<br><br>Accidental substituted for ibuprofen syrup	Vomiting but was alert and general condition remained stable	N/A	Full recovery	Low methodological quality<br><br>Conflicts of interest<br>None declared<br><br>Funding<br>Not reported
Lee et al., 2020<br><br>South Korea	Male<br>26 years	Approximately 10mL of 99% liquid nicotine (990mg/mL)<br><br>Ingestion	No pulse identified and performed cardiopulmonary resuscitation	Cardiopulmonary resuscitation and transferred to ICU	Hypoxic ischemic encephalopathy (brain death) caused by lethal nicotine intoxication	Moderate methodological quality<br><br>Conflicts of interest

Study details (author, year, location, data source [time frame])	Demographics and medical history	Exposure (e-liquid description, route of administration, cause of exposure)	Presentation	Treatment	Outcome	Quality assessment, conflict of interest, funding
Emergency department	Severe depression, medicated	Suicide attempt				None declared<br><br><u>Funding</u><br>None received
**Scarpino et al., 2020**<br><br>Italy, Florence<br><br>Emergency department	Male<br>23 years<br><br>Not reported	2 e-cigarettes refills<br><br>Ingestion<br><br>Unknown	Sudden loss of consciousness with vomiting, followed by bradycardia and respiratory muscle paralysis. Patient was in coma	Not reported	<u>Day nine of coma</u><br>Loss of respiratory drive and evolved toward brain death	Moderate methodological quality<br><br><u>Conflicts of interest</u><br>None declared<br><br><u>Funding</u><br>Not reported
**Aoki et al., 2019**<br><br>Japan<br><br>Emergency department	Female<br>19 years<br><br>Non-smoker, history of depression	Nicotine containing e-liquid<br><br>Intravenous injection<br><br>Unknown: suggestive of suicide, but no conclusive evidence	Cardiorespiratory arrest and was confirmed dead upon arrival at emergency department<br><br>The nicotine concentration was extremely high in the tissues around the injection mark on the right upper arm and reached a lethal level in the blood	N/A	Death due to high concentration of injected nicotine	Low methodological quality<br><br><u>Conflicts of interest</u><br>None declared<br><br><u>Funding</u><br>Not reported
**Belkoniene et al., 2019**<br><br>Switzerland<br><br>Emergency department	Male<br>51 years<br><br>Active e-cigarette user, history of cigarette smoking, type 2 diabetes mellitus and a personality disorder	10mL of 100mg/mL nicotine e-liquid<br><br>Injection<br><br>Suicide attempt	Abdominal cramps; psychomotor agitation and mydriatic pupils followed by bradypnea and coma<br><br>Developed a transitory neurological impairment with the appearance of tetraparesis, gaze palsy and myoclonus due to nicotinic syndrome<br><br>Lactic acidosis	Intubated in ICU using rapid sequence induction (etomidate, succinylcholine and fentanyl)	<u>7-10 hours post-injection</u>: woke up and answered simple questions. Pupils were still mydriatic and poorly responsive to light<br><br><u>11 hours post-injection:</u> complete recovery of motor response and normalisation of deep tendon reflexes allowing extubation<br><br><u>24 hours later:</u> discharged	Moderate methodological quality<br><br><u>Conflicts of interest</u><br>None declared<br><br><u>Funding</u><br>No funding provided
**Demir & Topal, 2018**<br><br>Turkey<br><br>Pediatric emergency department	Female<br>6 years<br><br>Not reported	7mL liquid and 8.4mg nicotine with nicotine ratio 1.2mg/mL that was storage in an e-liquid bottle. The estimated nicotine intake of the whole bottle was 8.4mg<br><br>Ingestion<br><br>Accidental	Nausea and vomiting<br><br>Bilateral sudden sensorineural hearing loss (SSNHL) after 24-hour fluid intake	Gastric lavage	<u>6th month of follow-up</u>: audiometric test results same as the results at the 10th day. Patient started using bilateral conventional hearing devices	High methodological quality<br><br><u>Conflicts of interest</u><br>None declared<br><br><u>Funding</u><br>Not reported

Study details (author, year, location, data source [time frame])	Demographics and medical history	Exposure (e-liquid description, route of administration, cause of exposure)	Presentation	Treatment	Outcome	Quality assessment, conflict of interest, funding
Paik et al., 2018<br><br>South Korea<br><br>Emergency department	Male<br>53 years<br><br>No known medical illness	3mL of e-liquid, brand name 'Pure Nicotine', concentration unknown<br><br>Ingestion<br><br>Suicide attempt	Immediately after ingestion<br>The patient exhibited tachycardia, vomiting, diarrhea, and sweating without hypotension<br><br>One hour after ingestion<br>Bradycardia, hypotension, and severe weakness	Administered dopamine	Blood pressure normalised within 18 hours of admission, discharged after 3 days	Moderate methodological quality<br><br>Conflicts of interest<br>None declared<br><br>Funding<br>Inha University Research Grant
Morely et al., 2017<br><br>United Kingdom<br><br>Hospital record	Male<br>32 years<br><br>Not reported	Approximately 20mL from an e-liquid bottle containing 72mg/mL nicotine liquid<br><br>Ingestion<br><br>Accidental, inebriated at the time	Agitated, collapsed then went into cardiac arrest prior to reaching hospital	Cardiopulmonary resuscitation and transferred to ICU	Death due to brain hypoxia, attributed to prolonged cardiopulmonary resuscitation	Moderate methodological quality<br><br>Conflicts of interest<br>Not reported<br><br>Funding<br>None received
van der Meer et al., 2017<br><br>The Netherlands<br><br>ICU	Male<br>42 years<br><br>Bipolar disorder	Nicotine containing e-liquid 450mg/mL<br><br>Ingestion<br><br>Unknown	No heart rhythm. Poor neurological status. High nicotine level in body: 3.0mg/L<br><br>(Reference values for a smoker are 0.01-0.05mg/L)	Cardiac massage and symptomatic treatment	Died of post anoxic encephalopathy	Moderate methodological quality<br><br>Conflicts of interest<br>Not reported<br><br>Funding<br>Not reported

Percentages and p-values are presented as reported in original studies.
Dec = December; ICU = Intensive Care Unit; SSNHL = sudden sensorineural hearing loss; TTM = targeted temperature management; US = United States.

Table 8.1. Study details: mental health effects – cohort studies

<table>
<tr><th>Study details (author, year, location, time frame, [data source])</th><th>Sample characteristics</th><th>Exposure/Comparator</th><th>Outcome measure</th><th>Results</th><th>Quality assessment, study size, conflicts of interest, funding</th></tr>
<tr>
<td>Marsden et al., 2019

US

2014-2017

Marketing and Promotions across Colleges in Texas project (M-PACT)</td>
<td><u>Study size</u>
5,236 participants

<u>Sample</u>
Past 30-day user

<u>Gender - n (%)</u>
Male: 1,919/5,236 (36.7%)
Female: 3,317,5,236 (63.3%)

<u>Age - mean (SD) years</u>
21.0 (2.3)
Range: 18-29</td>
<td><u>Exposure 1 (n=768)</u>
Refillable e-cigarettes

<u>Exposure 2 (n=303)</u>
Disposable e-cigarettes

<u>Comparator</u>
Within person

<u>Materials</u>
No information

<u>Follow-up</u>
Six waves of data from October 2014 through June 2017; approximate 6-monthly follow-up</td>
<td>Depressive symptoms (measured with the Center for Epidemiologic Studies Depression 10 scale - CES-D-10)</td>
<td><u>Hierarchical Poisson model - includes multiple product user</u>
<table>
<tr><th>Frequency of use[1]</th><th>Rate ratio</th><th>95% CI</th><th>P</th></tr>
<tr><td>Refillable e-cigarette</td><td>1.01</td><td>1.00-1.03</td><td>0.02</td></tr>
<tr><td>Disposable e-cigarette</td><td>1.00</td><td>0.98-1.03</td><td>0.92</td></tr>
<tr><td>Cigarettes</td><td>1.03</td><td>1.02-1.04</td><td><0.001</td></tr>
<tr><td>Past 30-day use[2]</td><td></td><td></td><td></td></tr>
<tr><td>Refillable e-cigarette</td><td>1.03</td><td>1.00-1.05</td><td>0.04</td></tr>
<tr><td>Disposable e-cigarette</td><td>1.05</td><td>0.99-1.11</td><td>0.13</td></tr>
<tr><td>Cigarettes</td><td>1.04</td><td>1.01-1.06</td><td><0.01</td></tr>
</table>
-Adjusted for race/ethnicity, sex, baseline age, two- vs. four-year college, father's education and survey wave
[1]The number of days of tobacco product use in the past 30 days was scaled so that each one-unit increase represents an additional 5 days of use.
[2]Past 30-day use was adjusted for frequency of use

<u>Model-based estimates of the associations - include single product users</u>
<table>
<tr><th>5 days of use in the past 30 days</th><th>Rate ratio</th><th>95% CI</th></tr>
<tr><td>Refillable e-cigarette</td><td>1.04</td><td>1.02-1.07</td></tr>
<tr><td>Disposable e-cigarette</td><td>1.05</td><td>0.99-1.11</td></tr>
<tr><td>Cigarettes</td><td>1.07</td><td>1.04 -1.09</td></tr>
<tr><td>15 days of use in the past 30 days</td><td></td><td></td></tr>
<tr><td>Refillable e-cigarette</td><td>1.07</td><td>1.04-1.11</td></tr>
<tr><td>Disposable e-cigarette</td><td>1.05</td><td>0.98-1.13</td></tr>
<tr><td>Cigarettes</td><td>1.13</td><td>1.10-1.16</td></tr>
</table>
Estimates account for frequency of use, past 30-day use and relevant interactions. All associations are adjusted for race/ethnicity, sex, baseline age, two- vs. four-year college, father's education and survey wave</td>
<td>Low methodological quality

Large study size, number of events not reported

<u>Conflicts of interest</u>
None declared

<u>Funding</u>
National Cancer Institute at the National Institutes of Health and the US Food and Drug Administration</td>
</tr>
</table>

Percentages and p-values are presented as reported in original studies.
CES-D-10 = Center for Epidemiologic Studies Depression 10 Scale; CI = confidence interval; M-PACT = Marketing and Promotions Across Colleges in Texas; SD = standard deviation; US = United States.

9. Environmental hazards with health implications

Table 9.1 Study details: environmental hazards with health implications – controlled and natural experiments

<table>
<tr><th>Study details (author, year, study design)</th><th>Setting</th><th>Experimental conditions</th><th>Outcome measure</th><th>Results</th><th>Quality assessment, conflict of interest, funding</th></tr>
<tr><td colspan="6">Controlled experiments</td></tr>
<tr>
<td>Protano et al., 2020

Italy

Open-label, single-centre, controlled study</td>
<td>Room with closed window and door, single occupant, 3 participants

<u>Area size</u>
52.7m^3

<u>Temperature</u>
20-23°C

<u>Relative humidity</u>
36%-40%</td>
<td><u>Experimental</u>
During vaping session; 12 puffs were made for each session (approximately 5-6 minutes); 2 blocks, 15 sessions in each

<u>Control</u>
Before vaping session

<u>Device</u>
JUUL, 4 flavours (Golden Tobacco, Mango, Mint, Royal Crème)</td>
<td><u>Particulate matter</u>
PM1 ($\mu g/m^3$)

Average session time 5.5 minutes</td>
<td><u>PM1</u> - mean (SD)
<table>
<tr><th>Flavour</th><th>Experimental</th><th>Control</th></tr>
<tr><td>Golden Tobacco</td><td>1637.9 (6387.6)</td><td>8.3 (2.3)</td></tr>
<tr><td>Mango</td><td>37.7 (208.3)</td><td>10.9 (1.5)</td></tr>
<tr><td>Mint</td><td>16.7 (5.4)</td><td>13.8 (1.9)</td></tr>
<tr><td>Royal Crème</td><td>16.0 (5.0)</td><td>13.3 (1.5)</td></tr>
</table>
Statistically significant difference (<0.001) before and after vaping session for all tests. Median also published. Mean and median approximately equal in control condition. Mean notably higher than median in experimental condition for Golden Tobacco and Mango flavours only</td>
<td>High methodological quality

<u>Conflicts of interest</u>
None declared

<u>Funding</u>
No external funding</td>
</tr>
<tr>
<td rowspan="2">Savdie et al., 2020

Portugal

Open-label, single-centre, controlled studies</td>
<td>Sitting room occupied by 2 people

<u>Area size</u>
73m^3</td>
<td><u>Experimental</u>
During vaping session; one participant took 10 puffs for 5 minutes, 10-minute rest, repeated 8 times

<u>Control</u>
Non-smoking/vaping (“background” not further specified)

<u>Device</u>
1. JUUL (Slate JUUL, 4.5V, 8W, 5% nicotine pods)
2. Vape (IStick TC40W, nicotine free liquid) (ENNDS)</td>
<td><u>Particulate matter</u>
1. PM1 ($\mu g/m^3$)
2. PM2.5 ($\mu g/m^3$)
3. PM10 ($\mu g/m^3$)
4. Ultrafine particles (UFP) (#/cm^3)

<u>5. Black carbon ($\mu g/m^3$)</u>

<u>Gases</u>
6. Carbon monoxide (CO) (mg/m^3)
7. Carbon dioxide (CO_2) (mg/m^3)

Average session time
5 minutes</td>
<td><u>Particulate matter, black carbon and gases</u> - mean
<table>
<tr><th></th><th>Control</th><th>Experimental</th></tr>
<tr><td>PM1</td><td>21.0</td><td>1,350*</td></tr>
<tr><td>PM2.5</td><td>22.6</td><td>1,370*</td></tr>
<tr><td>PM10</td><td>25.4</td><td>1,380*</td></tr>
<tr><td>UFP</td><td>4,690</td><td>37,800*</td></tr>
<tr><td>Black carbon</td><td>0.21</td><td>4.3</td></tr>
<tr><td>CO</td><td>1.66</td><td>1.00</td></tr>
<tr><td>CO_2</td><td>1,810</td><td>2,890</td></tr>
</table>
* Statistically different to control (p<0.05)
^ Approximate from graphed data
Statistical significance test results not reported for black carbon, CO, CO_2

Note: JUUL and ENNDS not reported separately</td>
<td rowspan="2">High methodological quality

<u>Conflicts of interest</u>
None declared

<u>Funding</u>
Supported by LIFE Index-Air project and Portuguese Foundation for Science and Technology</td>
</tr>
<tr>
<td>Medium volume car (Diesel Opel Corsa, from 2007) occupied by 2 people</td>
<td><u>Experimental</u>
During vaping session; one participant took 10 puffs for 3 minutes, 7-minute rest, repeated 3 times

<u>Control</u>
Non-smoking/vaping (test drive)

<u>Device</u>
1. JUUL (Slate JUUL, 4.5V, 8W, 5% nicotine pods)</td>
<td><u>Particulate matter</u>
1. PM1 ($\mu g/m^3$)
2. PM2.5 ($\mu g/m^3$)
3. PM10 ($\mu g/m^3$)
4. Ultrafine particles (UFP) (#/cm^3)

<u>5. Black carbon ($\mu g/m^3$)</u>

<u>Gases</u>
6. Carbon monoxide (CO) (mg/m^3)</td>
<td><u>Particulate matter, black carbon and gases</u> - mean
<table>
<tr><th></th><th colspan="2">JUUL</th><th colspan="2">ENNDS</th></tr>
<tr><th></th><th>Experimental</th><th>Control</th><th>Experimental</th><th>Control</th></tr>
<tr><td>PM1</td><td>129</td><td>19.2</td><td>1,150</td><td>21.0</td></tr>
<tr><td>PM2.5</td><td>131</td><td>21.1</td><td>1,170</td><td>21.8</td></tr>
<tr><td>PM10</td><td>134*</td><td>24.5</td><td>1,170*</td><td>23.3</td></tr>
<tr><td>UFP</td><td>47,800</td><td>28,500</td><td>56,300</td><td>17,600</td></tr>
<tr><td>Black carbon</td><td>1.15</td><td>0.57</td><td>0.70</td><td>0.59</td></tr>
<tr><td>CO</td><td>0.82</td><td>0.43</td><td>1.09</td><td>0.43</td></tr>
<tr><td>CO_2</td><td>982</td><td>883</td><td>1,090</td><td>956</td></tr>
</table>
* Statistically different to control (p<0.05)</td>
</tr>
</table>

<table>
<thead>
<tr><th>Study details (author, year, study design)</th><th>Setting</th><th>Experimental conditions</th><th>Outcome measure</th><th>Results</th><th>Quality assessment, conflict of interest, funding</th></tr>
</thead>
<tbody>
<tr><td></td><td></td><td>2. Vape (IStick TC40W, nicotine free liquid) (ENNDS)</td><td>7. Carbon dioxide (CO_2) (mg/m^3)

Average session time
3 minutes</td><td>Statistical significance test results not reported for PM1, PM2.5, UFP, black carbon, CO, CO_2</td><td></td></tr>
<tr><td>Loupa et al., 2019

Greece

Open-label, single-centre study</td><td>Residential living room, with wall-mounted air conditioner, occupied by two people

<u>Area size</u>
$126m^3$</td><td><u>Experimental</u>
During vaping session, one participant vaped for 10 minutes, approximately 2 puffs per minute with 1-minute interval between puffs

<u>Control</u>
Tobacco cigarettes

<u>Device</u>
E-cigarette, no nicotine (ENNDS)</td><td><u>Particulate matter</u>
1. PM2.5 ($\mu g/cm^3$)
2. PM10 ($\mu g/cm^3$)

Average session time
10 minutes</td><td><u>Particulate matter</u>
<table>
<tr><th></th><th colspan="2">PM2.5</th><th colspan="2">PM10</th></tr>
<tr><th></th><th>Mean (SD)</th><th>Min-Max</th><th>Mean (SD)</th><th>Min-Max</th></tr>
<tr><td>ENDS</td><td>74.78 (96.12)</td><td>1.44-288.72</td><td>82.06 (98.95)</td><td>2.02-294.76</td></tr>
<tr><td>Cigarettes</td><td>55.32 (31.32)</td><td>2.37-97.25</td><td>62.19 (31.11)</td><td>3.67-106.83</td></tr>
</table>
</td><td>High methodological quality

<u>Conflicts of interest</u>
Not reported

<u>Funding</u>
University funds</td></tr>
</tbody>
</table>

<table>
<tr><th>Study details (author, year, study design)</th><th>Setting</th><th>Experimental conditions</th><th>Outcome measure</th><th>Results</th><th>Quality assessment, conflict of interest, funding</th></tr>
<tr>
<td>Schober et al., 2019

Germany

Open-label, multi-centre, controlled study</td>
<td>1. Large (4-5m^3): Skoda Octavia (Skoda), Volvo S (Volvo)
2. Medium (3-4m^3): VW Golf (2001,-05,-06) (Golf 01, Golf 05, Golf 06)
3. Small (2-3m^3): Smart ForFour (Smart), Fiat Punto (Fiat)

Each occupied by 2 people

Passenger window 2cm or 5cm open</td>
<td><u>Experimental</u>
During vaping session; passenger used e-cigarette, four second inhalation twice per minute

<u>Control</u>
No vaping/smoking (test drive)

<u>Device</u>
SubTwin Neo; tobacco-flavoured liquid, nicotine content 18mg/mL</td>
<td><u>Particulate matter</u>
1. Nano particle concentration (PNC diameter 25-300nm) (#/cm^3)
2. Fine particle concentration (PNC diameter >300nm) (#/cm^3)
3. PM2.5 (μg/m^3)

<u>4. Propylene glycol</u>

<u>5. Nicotine</u>

<u>Volatile organic and organic compounds</u> (μg/m^3)
6. Benzene
7. Toluene
8. Furfural
9. 3-Ethenylpyridine

<u>Carbonyls</u>
10. Formaldehyde
11. Acetaldehyde
12. Propionaldehyde
13. Acetone
14. 2-Butanone

Average session time 20-23 minutes</td>
<td><u>Particulate matter</u> - mean
<table>
<tr><th></th><th>PNC (25-300nm)</th><th></th><th>PNC (>300nm)</th><th></th><th>PM2.5</th><th></th></tr>
<tr><th></th><th>Experimental</th><th>Control</th><th>Experimental</th><th>Control</th><th>Experimental</th><th>Control</th></tr>
<tr><td>Skoda</td><td>53,579</td><td>10,491</td><td>2,145</td><td>20</td><td>490</td><td>6</td></tr>
<tr><td>Volvo</td><td>14,209</td><td>20,231</td><td>659</td><td>41</td><td>170</td><td>10</td></tr>
<tr><td>Golf 06</td><td>33,014</td><td>20,675</td><td>1,362</td><td>22</td><td>262</td><td>7</td></tr>
<tr><td>Golf 05</td><td>73,954</td><td>73,941</td><td>1,188</td><td>40</td><td>269</td><td>11</td></tr>
<tr><td>Golf 01</td><td>10,248</td><td>8,434</td><td>289</td><td>18</td><td>75</td><td>7</td></tr>
<tr><td>Smart</td><td>13,543</td><td>17,716</td><td>90</td><td>14</td><td>18</td><td>4</td></tr>
<tr><td>Fiat</td><td>19,901</td><td>18,626</td><td>28</td><td>19</td><td>8</td><td>9</td></tr>
</table>
<u>Propylene glycol</u> passenger window: 2cm open/5cm open - mean
<table>
<tr><th></th><th>Skoda</th><th>Volvo</th><th>Golf 06</th><th>Golf 05</th><th>Golf 01</th><th>Smart</th><th>Fiat</th></tr>
<tr><td>ENDS</td><td>262/502</td><td>196/226</td><td>341/370</td><td>762/611</td><td>50/59</td><td><LD</td><td><LD</td></tr>
<tr><td>Control</td><td><LD</td><td><LD</td><td><LD</td><td><LD</td><td><LD</td><td><LD</td><td><LD</td></tr>
</table>
<u>Nicotine</u> passenger window: 2cm open/5cm open - mean
<table>
<tr><th></th><th>Skoda</th><th>Volvo</th><th>Golf 06</th><th>Golf 05</th><th>Golf 01</th><th>Smart</th><th>Fiat</th></tr>
<tr><td>ENDS</td><td>4/5</td><td><LD</td><td>4/<LD</td><td>10/7</td><td>5/<LD</td><td><LD</td><td><LD</td></tr>
<tr><td>Control</td><td><LD</td><td><LD</td><td><LD</td><td><LD</td><td><LD</td><td><LD</td><td><LD</td></tr>
</table>
<LD: measurement was below the limit of detection (LD)
No significance testing was reported
No correlation found between the different ventilation conditions (passenger window 2cm vs. 5cm open)

<u>Other compounds</u>
No effect reported

<u>Carbonyls</u>
No effect reported</td>
<td>High methodological quality

<u>Conflicts of interest</u>
None declared

<u>Funding</u>
Not reported</td>
</tr>
</table>

Study details (author, year, study design)	Setting	Experimental conditions	Outcome measure	Results	Quality assessment, conflict of interest, funding
Coppeta et al., 2018<br><br>Italy<br><br>Open-label, single-centre, controlled study	Unknown setting with single occupant, 30 participants	Experimental<br>Active vaping; one participant performing 15 puffs over 5 minutes, and temporal variation during the subsequent 60 minutes<br><br>Control<br>Before vaping session<br><br>Device<br>EGO P (L) with manual start; Latakia tobacco flavour containing nicotine 1.8% (18mL/L)	Particulate matter<br>Concentration of airborne particles (#/cm^3)<br><br>Average session time: approximately 5 minutes (time to return to baseline particle concentration)	Particulate matter - mean (range)<br>ENDS: 49,690pp/cm^3 (5,040-50,000)<br>Control: 42,645pp/cm^3 (2,310-50,000)<br><br>No statistical tests conducted	Moderate methodological quality<br><br>Conflicts of interest<br>None declared<br><br>Funding<br>Not reported
van Drooge et al., 2019<br><br>Spain<br><br>Open-label, single-centre, controlled study	Closed room without direct contact with external air, occupied by 10 people<br><br>Area size<br>146m^3	Experimental<br>During vaping; 5 active vapers ab libium use during 12-hour period<br><br>Control<br>Non-vaping (day prior)<br><br>Device<br>E-liquid composition: Power (W), Nicotine (mg/mL), Proportion glycerine/propylene glycol<br>1. 50, 3, 70/30<br>2. 70, 3, 80/20<br>3. 45, 6, 50/50<br>4. 20, 3, 40/60<br>5. 15, 12, 30/70	Particulate matter<br>1. PM10 (μg/m^3)<br>2. PM2.5 (μg/m^3)<br>3. PM1 (μg/m^3)<br>4. Particle number concentration (PNC) (#/cm^3)<br><br>Organic compounds<br>5. Nicotine (μg/m^3)<br><br>Average session time 12 hours	Particulate matter - mean<br>(columns: PM10 / PM2.5 / PM1 / PNC)<br>ENDS: 60 / 20 / 14 / 9.6×10^3<br>Control: 25 / 10 / 6 / 5.2×10^3<br><br>Nicotine - mean<br>ENDS 16<br>Control 0.1	High methodological quality<br><br>Conflicts of interest<br>Not reported<br><br>Funding<br>Partial funding from EU projects HEALS, NEUROSOME, and EPPA S.A

<table>
<tr><th>Study details (author, year, study design)</th><th>Setting</th><th>Experimental conditions</th><th>Outcome measure</th><th>Results</th><th>Quality assessment, conflict of interest, funding</th></tr>
<tr>
<td>Protano et al., 2018

Italy

Open-label, single-centre, controlled study</td>
<td>Room with closed window and door, unspecified number of occupant participants

<u>Area size</u>
52.7m^3</td>
<td><u>Experimental</u>
During vaping session; 12 puffs were made for each session lasting approximately 5.5 minutes (1 puff about each thirty seconds); unknown number of active vapers

<u>Control</u>
Before vaping session

<u>Device</u>
1: First generation e-cigarettes (Young Category®)
2: Second generation e-cigarettes (Smooke®)
3: Third generation e-cigarettes (JustFog Q16 Kit®, voltage 3.4V - 4.8V, resistance 1.6 Ohm)
4: Fourth generation e-cigarettes (G 150 Smok Kit® with V8 Baby-Q2 Smok atomizer®, wattage variation from 25 to 150W, and the resistance of either 0.15 and 0.4 Ohm) ENDS and ENNDS
0.15Ω and 0.4Ω 25W, 50W, 55W, 80W, 100W and 150W</td>
<td><u>Particulate matter</u>
1. PM1 ($\mu g/m^3$)

Average session time 5.5 minutes</td>
<td><u>PM1</u> - mean (SD)
<table>
<tr><th></th><th>Experimental</th><th>Control</th></tr>
<tr><td>First generation</td><td></td><td></td></tr>
<tr><td>ENNDS</td><td>79.69 (80.13)</td><td>41.27 (19.09)</td></tr>
<tr><td>ENDS</td><td>105.52 (117.10)</td><td>43.86 (18.75)</td></tr>
<tr><td>Second generation</td><td></td><td></td></tr>
<tr><td>ENNDS</td><td>534.00 (1266.88)</td><td>21.34 (7.67)</td></tr>
<tr><td>ENDS</td><td>3428.85 (5857.54)</td><td>18.33 (6.74)</td></tr>
<tr><td>Third generation</td><td></td><td></td></tr>
<tr><td>ENNDS (3.4V)</td><td>789.48 (2300.46)</td><td>21.56 (6.31)</td></tr>
<tr><td>ENDS (3.4V)</td><td>54.39 (179.23)</td><td>26.22 (6.58)</td></tr>
<tr><td>ENNDS (4.8V)</td><td>522.29 (1729.70)</td><td>21.45 (6.75)</td></tr>
<tr><td>ENDS (4.8V)</td><td>1005.81 (4405.06)</td><td>26.22 (13.58)</td></tr>
<tr><td>Fourth generation</td><td></td><td></td></tr>
<tr><td>ENNDS (0.15Ω, 25W)</td><td>384.53 (1327.67)</td><td>20.96 (2.74)</td></tr>
<tr><td>ENDS (0.15Ω, 25W)</td><td>963.24 (4605.46)</td><td>35.44 (6.32)</td></tr>
<tr><td>ENNDS (0.4Ω, 55W)</td><td>74.50 (40.70)</td><td>31.67 (8.79)</td></tr>
<tr><td>ENDS (0.4Ω, 55W)</td><td>472.93 (1181.44)</td><td>43.87 (6.23)</td></tr>
<tr><td>ENNDS (0.4Ω, 80W)</td><td>2238.34 (3931.00)</td><td>35.44 (6.32)</td></tr>
<tr><td>ENDS (0.4Ω, 80W)</td><td>14887.00 (25725.24)</td><td>41.66 (7.36)</td></tr>
<tr><td>ENNDS (0.15Ω, 50W)</td><td>177.69 (80.61)</td><td>41.27 (19.09)</td></tr>
<tr><td>ENDS (0.15Ω, 50W)</td><td>5949.16 (15452.17)</td><td>43.55 (7.73)</td></tr>
<tr><td>ENNDS (0.15Ω, 100W)</td><td>5637.34 (19136.38)</td><td>39.28 (17.21)</td></tr>
<tr><td>ENDS (0.15Ω, 100W)</td><td>2572.72 (4301.85)</td><td>43.55 (7.73)</td></tr>
<tr><td>ENNDS (0.15Ω, 150W)</td><td>12925.34 (31590.92)</td><td>41.27 (19.09)</td></tr>
<tr><td>ENDS (0.15Ω, 150W)</td><td>14640.47 (32776.91)</td><td>44.67 (8.59)</td></tr>
</table>
Statistically significant difference (<0.001) before and after vaping session for all tests. Median also published. Mean and median approximately equal in control condition. Mean notably higher than median in experimental condition</td>
<td>High methodological quality

<u>Conflicts of interest</u>
None declared

<u>Funding</u>
No external funding</td>
</tr>
</table>

<table>
<tr><th>Study details (author, year, study design)</th><th>Setting</th><th>Experimental conditions</th><th>Outcome measure</th><th>Results</th><th>Quality assessment, conflict of interest, funding</th></tr>
<tr>
<td>Volesky et al., 2018

Canada

Open-label, single-centre, controlled study</td>
<td>Closed room with two occupants, volunteer e-cigarette user situated near the centre facing the measurement devices either 0.5m or 1m away

<u>Area size</u>
~$38m^3$</td>
<td><u>Experimental</u>
During vaping; one active vaper took 4-second puffs 7 times, repeated 3 times

<u>Control</u>
No vaping (before and after vaping session)

<u>Device</u>
1. Cigalike e-cigarette (cigalike)
2. Tank e-cigarette (tank)
3. Adjustable voltage e-cigarette (adjustable)

E-liquid: Gold Seal™ brand “sweetish berry”, 12mg/mL nicotine, 70% propylene glycol, 30% vegetable glycerin</td>
<td><u>Particulate matter</u>
1. Particulate matter size <2.5µm (PM2.5) (µg/m^3)
2. Ultrafine particles (UFP) (#/cm^3)

Average session time 6.5 minutes</td>
<td><u>PM2.5</u>
<table>
<tr><th></th><th></th><th>0.5 metres from user</th><th></th><th></th><th>1 metre from user</th><th></th></tr>
<tr><th>Mean</th><th>C(B)</th><th>ENDS</th><th>C(A)</th><th>C(B)</th><th>ENDS</th><th>C(A)</th></tr>
<tr><td>Cigalike</td><td>2</td><td>709</td><td>2</td><td>3</td><td>168</td><td>31</td></tr>
<tr><td>Tank</td><td>2</td><td>1,117</td><td>7</td><td>2</td><td>1,193</td><td>152</td></tr>
<tr><td>Adjust</td><td>2</td><td>364</td><td>2</td><td>2</td><td>235</td><td>3</td></tr>
<tr><td></td><td>p=0.665</td><td></td><td></td><td>p<0.001</td><td></td><td></td></tr>
<tr><td>Maximum</td><td></td><td></td><td></td><td></td><td></td><td></td></tr>
<tr><td>Cigalike</td><td>48</td><td>174,160</td><td>514</td><td>369</td><td>20,333</td><td>24</td></tr>
<tr><td>Tank</td><td>46</td><td>164,164</td><td>20</td><td>7</td><td>28,288</td><td>1,683</td></tr>
<tr><td>Adjust</td><td>87</td><td>77,181</td><td>88</td><td>92</td><td>28,991</td><td>186</td></tr>
</table>
C(B) = Control (before); C(A) = Control (after)

<u>Ultrafine particles</u>
<table>
<tr><th></th><th></th><th>0.5 metres from user</th><th></th><th></th><th>1 metre from user</th><th></th></tr>
<tr><th>Mean</th><th>C(B)</th><th>ENDS</th><th>C(A)</th><th>C(B)</th><th>ENDS</th><th>C(A)</th></tr>
<tr><td>Cigalike</td><td>1,173</td><td>11,106</td><td>4,353</td><td>2,828</td><td>10,366</td><td>6,326</td></tr>
<tr><td>Tank</td><td>922</td><td>14,541</td><td>4,736</td><td>4,522</td><td>26,424</td><td>9,990</td></tr>
<tr><td>Adjust</td><td>2,073</td><td>8,060</td><td>4,499</td><td>3,124</td><td>9,699</td><td>5,910</td></tr>
<tr><td></td><td>p=0.710</td><td></td><td></td><td>p<0.001</td><td></td><td></td></tr>
<tr><td>Maximum</td><td></td><td></td><td></td><td></td><td></td><td></td></tr>
<tr><td>Cigalike</td><td>4,801</td><td>284,260</td><td>14,044</td><td>5,879</td><td>255,713</td><td>11,015</td></tr>
<tr><td>Tank</td><td>1,182</td><td>270,368</td><td>10,551</td><td>6,533</td><td>232,524</td><td>37,628</td></tr>
<tr><td>Adjust</td><td>3,064</td><td>235,840</td><td>8,992</td><td>4,832</td><td>249,281</td><td>12,190</td></tr>
</table>
C(B) = Control (before); C(A) = Control (after)</td>
<td>High methodological quality

<u>Conflicts of interest</u>
Not reported

<u>Funding</u>
No specific funding. Health Canada provided measurement devices and technical expertise and Carleton University’s covered material costs</td>
</tr>
<tr><td colspan="6">Natural experiments</td></tr>
<tr>
<td>Cammalleri et al., 2020

Italy

Open-label, single-centre, controlled study</td>
<td>Outdoors of the “Del Vecchio” library of the Department of Public Health and Infectious Diseases of Sapienza University of Rome, unknown number of participants

No known other sources of PM1</td>
<td><u>Experimental</u>
During vaping session; one participant vaping one e-cigarette or JUUL

<u>Control</u>
Before vaping session

<u>Device</u>
Electronic cigarette (not further defined) and JUUL (no description)</td>
<td><u>Particulate matter</u>
PM1 (µg/m^3)</td>
<td><u>PM1</u>
<table>
<tr><th></th><th>During vaping session</th><th></th><th>Before vaping session</th><th></th><th></th></tr>
<tr><th></th><th>Mean (SD)</th><th>Median (IQR)</th><th>Mean (SD)</th><th>Median (IQR)</th><th>P</th></tr>
<tr><td>E-cigarette</td><td>394.82 (1317.66)</td><td>23.00 (29.00)</td><td>28.81 (1.94)</td><td>23.00 (2.00)</td><td><0.023</td></tr>
<tr><td>JUUL</td><td>159.13 (304.74)</td><td>34.00 (107.00)</td><td>29.25 (1.59)</td><td>29.00 (2.00)</td><td>0.003</td></tr>
</table></td>
<td>High methodological quality

<u>Conflicts of interest</u>
None declared

<u>Funding</u>
No external funding</td>
</tr>
</table>

Study details (author, year, study design)	Setting	Experimental conditions	Outcome measure	Results	Quality assessment, conflict of interest, funding
Khachatoorian et al., 2019<br><br>US<br><br>Two site, natural experiment	Living room with adequate ventilation<br><br>Area size<br>187.5ft^2 (47.78m^3)	Experimental<br>Vaping use approximately 3 hours/day; average 15 days per month; fabric hung on a desk located near a window<br><br>Control<br>Non-smokers home (no further information provided)<br><br>Device<br>Innokin iTaste MVP and Wotofo ZNA 30 clone by A-mod Technology Co., LTD with Aspire Nautilus tank; e-liquid nicotine concentration of 6mg/mL<br><br>Duration<br>1-6 months	1. Nicotine (ng/g)<br>2. Cotinine (ng/g)<br><br>Collected on polyester or cotton fabric sample<br><br>Fabrics were collected after 1, 2, 3, 4, 5, and 6 months of exposure	Nicotine - total<br>Most abundant marker of e-cigarette exhaled aerosol residue contamination<br><br>Maximum: 5100ng/gram on cotton fabric at month 3<br>Range: 2000-3000ng/gram on cotton fabric (excluding month 3)<br><br>Only detected month 5 and 6 on polyester samples<br><br>Cotinine - total<br>Detected at all months for cotton sample, only detected at month 1, 3 and 4 for polyester sample	Moderate methodological quality<br><br>Conflicts of interest<br>None declared<br><br>Funding<br>Supported by Tobacco-Related Disease Research Program of California; the National Institute on Drug Abuse, USA and the National Center for Research Resources
Mock & Hendlin, 2019<br><br>US<br><br>July 2018-April 2019<br><br>Garbology study (ethno-archaeological study of a community or cultural group by analysing its waste)	Purposively selected, non-random sample of 12 public high schools in Alameda, Contra Costa, Marin, and San Francisco counties in California; student parking lots and exterior school perimeter	N/A	1. JUUL or JUUL-compatible pods<br>2. JUUL or JUUL-compatible caps<br>3. JUUL 4-packs<br>4. Total number of JUUL and JUUL-compatible items	Count of product waste items (#)<br>Total<br>Pods 47<br>Caps 123<br>4-Packs 3<br>Total* 173<br>*Reported total=172	Moderate methodological quality<br><br>Conflicts of interest<br>None declared<br><br>Funding<br>Not reported

Study details (author, year, study design)	Setting	Experimental conditions	Outcome measure	Results	Quality assessment, conflict of interest, funding
Nguyen et al., 2019<br><br>US<br><br>Multi-centre, natural experiment	Vape shop (location, ventilation type)<br>1. Storefront, A/C<br>2. Storefront, Central<br>3. Plaza, Natural<br>4. Storefront, Natural<br>5. Plaza, None<br>6. Storefront, A/C<br><br>Area size (m^3)<br>1. 318<br>2. 262<br>3. 244<br>4. 323<br>5. 168<br>6. 175	Experimental dimensions<br>Indoor<br><br>Control dimensions<br>Outdoor<br><br>Pattern of use<br>Total vaping frequency (TVF) #/30 minutes<br>(average across all conditions)<br>1. 88 (96)<br>2. 19 (16)<br>3. 16 (15)<br>4. 9 (5)<br>5. 91 (25)<br>6. 13 (3)	Particulate matter<br>1. Particle number (#/cm^3)<br>2. PM2.5 (µg/m^3)<br><br>Average session time<br>8-10 hours	Particle number - range<br>Indoor - no active e-cigarette use: 5.5×10^{3} to 3.3×10^{4} particles/cm^3<br>Indoor - active e-cigarette use: 1.3×10^{4} to 4.8×10^{5} particles/cm^3<br>Outdoor - 8.5×10^{3} to 5.6×10^{4} particles/cm^3<br><br>PM2.5 - range<br>Indoor - no active e-cigarette use: 3.2 to 39 µg/m^3<br>Indoor - active e-cigarette use: 15.5 to 37,500 µg/m^3<br>Outdoor - 7.5 to 72µg/m^3<br><br>Due to a small number of sampled vape shops, significant linear correlations between real-time PM concentrations could not be observed	Moderate methodological quality<br><br>Conflicts of interest<br>Not reported<br><br>Funding<br>Supported by the Tobacco-Related Disease Research Program and the Center for Occupational and Environmental Health at the University of California, Los Angeles
Khachatoorian et al., 2018<br><br>US<br><br>One site, natural experiment	Actively operated shop located on basement floor of two-story mall next to active vape shop<br><br>Area size<br>Vape shop:<br>405ft^2 (37m^2)<br><br>Study site-adjacent shop<br>311ft^2 (28m^2)	Experimental<br>Fabric placement inside shop located next to vape shop;<br>Filter placement<br>- in the return vent towards the back of suite<br>- in the middle of the suite<br><br>Control<br>Unexposed samples plus<br>Control fabrics (terrycloth) placed<br>- in a hallway outside the field site<br>- in a non-smoker home in the same community<br><br>Duration<br>Short-term exposure: 1 day (24 hours), 4 days (96 hours) and 8 days (192 hours)<br>Long-term exposure: 1, 2 and 3 months	1. Nicotine (ng/g)<br>2. Cotinine (ng/g)<br><br>Collected on cotton towels, paper towels, terrycloth towels samples and air filters<br><br>Samples were collected after 1, 4, and 8 days and after 1, 2 and 3 months	Nicotine - total<br>Nicotine was the most abundant marker of e-cigarette aerosol contamination (highest concentration=23,260ng/g of fabric). Its concentration generally increased with exposure time<br><br>Cotinine - total<br>Cotinine concentrations generally increased as exposure time increased. The air filters appeared to trap cotinine<br><br>Frequency of nicotine and cotinine<br>(columns: Cotton towel / Paper towel)<br>Nicotine: 100% / 92%<br>Cotinine: 22% / 83%<br><br>Control samples of paper towels and terrycloth towels exposed both in the home of a non-smoker and in the mall had no detectable nicotine or cotinine except for a low nicotine level (107ng/g and 93ng/g) in two samples	Moderate methodological quality<br><br>Conflicts of interest<br>None declared<br><br>Funding<br>Supported by Tobacco-Related Disease Research Program of California; the National Institute on Drug Abuse, USA and the National Center for Research Resources

Percentages and p-values are presented as reported in original studies.

A/C = air conditioner; C(A) = control (after); C(B) = control (before); ENDS = electronic nicotine delivery system; ENNDS = electronic non-nicotine delivery system; EPPA = Environment Partnership Programme for Accession; EU = European Union; HEALS = Health and Environment-wide Associations based on Large population Surveys; IQR = interquartile range; LD = limit of detection; max = maximum; min = minimum; PM = particulate matter; PNC = particle number concentration; pp = part(s) per; SD = standard deviation; TVF = total vaping frequency; UFP = ultrafine particle(s); US = United States; USA = United States of America.

Table 9.2 Study details: environmental hazards with health implications – surveillance reports

Study details (author, publication year)	Context (country, time frame, data source)	Number of fires/explosions	Circumstance of e-cigarette fire/explosion	Loss of property/fire spread	Quality assessment, conflict of interest, funding
Saxena et al., 2018	US<br><br>January 2009 to December 31, 2016<br><br>National Fire Data Center*<br><br>*Same data sources as below	Total fires/explosions* n=195<br><br>*Same data sources as below	Battery operating conditions during occurrence of e-cigarette fire incidents*:<br>Usage: 31%<br>Spare battery: 31%<br>Charging: 25%<br>Transport/storage/unknown: 13%<br><br>*Same data sources as below	Not reported	Low methodological quality<br><br>Conflicts of interest<br>Not reported<br><br>Funding<br>Not reported
	Various<br><br>August 2009 to April 2017<br><br>Blog reports (Ecigone Blog)	Total fires/explosions n=243	Battery operating conditions during occurrence of e-cigarette fire incidents:<br>Usage: 26%<br>Spare battery: 18%<br>Charging: 35%<br>Transport/storage/unknown: 21%	Not reported	
US Fire Administration, 2017	US<br><br>January 2009 to December 31 2016<br><br>National Fire Incident Reporting System (NFIRS)	Total fires/explosions n=195	Battery operating conditions during occurrence of e-cigarette fire incidents (N=195) - n (%)<br>In pocket: 61 (31.3%)<br>In use: 60 (30.8%)<br>Charging: 48 (24.6%)<br>Storage: 18 (9.2%)<br>Not reported: 7 (3.6%)<br>Transport: 1 (0.5%)	Resulted in ignition of nearby contents: 128/195 (66%)<br><br>Fire spread (N=195) - n (%)<br>Minor: 91 (46.7%)<br>None reported: 67 (34.4%)<br>Moderate: 27 (13.8%)<br>Major: 10 (5.1%)	Grey literature -no quality assessment<br><br>Conflicts of interest<br>Not reported<br><br>Funding<br>Not reported

Percentages and p-values are presented as reported in original studies.
NFIRS = National Fire Incident Reporting System; US = United States.

10. Neurological outcomes

Table 10.1. Study details: neurological outcomes – surveillance reports

Study details (author, publication year, country, time frame, data source)	Demographics	Exposure (e-liquid description, route of administration, cause of exposure)	Presentation and symptoms	Treatment	Outcome	Quality assessment, conflict of interest, funding
**Obertova et al., 2020**<br><br>Czech Republic<br><br>2012-2018<br><br>Toxicological Information Centre (TIC)<br><br>*The Centre recorded 148 phone calls in total (three animal exposures and 145 human)	Total human cases in surveillance report n=145*<br><br>Cases with neurological outcomes n=6	Not reported	Symptoms (N=148)* - n (%)<br>Tremor: 2 (1.4%)<br>Convulsion: 3 (2.0%)<br>Auditory hallucination: 1 (0.7%)	Not reported	Not reported	High methodological quality<br><br>Conflicts of interest<br>None declared<br><br>Funding<br>First Faculty of Medicine, Charles University; Ministry of Health Czech Republic
**Faulcon et al., 2019**<br><br>US<br><br>2010-2019<br><br>The Food and Drug Administration (FDA) Center for Tobacco Products	Sample size<br>123 new and experienced e-cigarette users, 82 (67%) in 14-24 year olds<br><br>Gender (14-24 years) - n (%)<br>Male: 54/82 (66%)<br><br>Age - median (IQR) years<br>20 (17-27)<br><br>Prior history of seizures - n (%)<br>14-24 years: 5/82 (6%)<br><br>Ethnicity (14-24 years) - n (%)<br>White: 74/82 (90%)	JUUL, Suorin, SMOK, and Vuse brands were the most commonly named	Symptoms - total sample (N=123) - n (%)<br>Seizure: 114 (93%)<br>Syncope: 8 (7%)<br>Tremor: 1 (1%)<br><br>Symptoms - 14-24 years (N=82) - n (%)<br>Seizure: 77 (94%)<br>Syncope: 4 (5%)<br>Tremor: 1 (1%)<br><br>Timing - total sample - n (%)<br>After first use: 8<br>Seizure within 30 minutes of last use*: 49/79 (62%)<br>Seizure within 2 hours of last use*: 5/79 (6%)<br>Seizure within 24 hours of last use*: 67/79 (85%)<br>*Information available for 79 reports<br><br>Seizures occurred immediately after one puff, all-day use, and with use weeks before the event	Not reported	Continued use after seizure - n (%)<br>14-24 years: 45/82 (55%)<br><br>Repeat seizures with continued ENDS use - n (%)<br>14-24 years: 33/45 (73%)	Low methodological quality<br><br>Conflicts of interest<br>None declared<br><br>Funding<br>Not reported
**Govindarajan et al., 2018**<br><br>US<br><br>2012-2017<br><br>National Poison Data System (NPDS)	Total cases in surveillance report n=8,269<br><br>Cases with neurological outcomes n=8	Not reported	Neurological effects (n)<br>Coma: 4<br>Seizure: 4	Not reported	Not reported	Moderate methodological quality<br><br>Conflicts of interest<br>None declared<br><br>Funding<br>Centers for Disease Control and Prevention and the Child Injury Prevention Alliance stipend

Percentages and p-values are presented as reported in original studies.
ENDS = electronic nicotine delivery system; FDA = Food and Drug Administration (US); IQR = interquartile range; NPDS = National Poison Data System; TIC = Toxicological Information Centre; US = United States.

11. Less serious adverse events

Table 11.1. Study details: less serious adverse events – randomised controlled trials and cohort studies

<table>
<tr><th>Study details (author, year, location, study type, time frame)</th><th>Sample characteristics</th><th>Intervention and control</th><th>Outcome measure</th><th>Results</th><th>Quality assessment, study size, conflicts of interest, funding</th></tr>
<tr><td colspan="6">Randomised controlled trials</td></tr>
<tr>
<td>Myers Smith et al., 2021

UK

Randomised controlled trial

2017-2018</td>
<td>Study size
135 smokers

Sample
Smokers

Gender (male) - n (%)
ENDS: 36/68 (53%)
NRT: 33/67 (49%)

Age - median (IQR) years
ENDS: 41 (16)
NRT: 40 (19)</td>
<td>Intervention (n=68)
ENDS: concentration of choice

Comparator (n=67)
Nicotine replacement therapy

Materials
ENDS of choice

Follow-up
6 months</td>
<td>Adverse events</td>
<td>Frequency of adverse events at week 1-24 - n
<table>
<tr><th></th><th>ENDS</th><th>NRT</th></tr>
<tr><td>Throat irritation</td><td>2</td><td>0</td></tr>
<tr><td>Nausea</td><td>1</td><td>2</td></tr>
<tr><td>Cough</td><td>3</td><td>1</td></tr>
<tr><td>Itchiness/skin irritation</td><td>0</td><td>11</td></tr>
<tr><td>Vivid dreams</td><td>0</td><td>1</td></tr>
<tr><td>Hiccups</td><td>0</td><td>1</td></tr>
<tr><td>Cough/throat/chest irritation</td><td>7</td><td>0</td></tr>
<tr><td>Dry mouth/throat</td><td>2</td><td>1</td></tr>
<tr><td>Indigestion</td><td>0</td><td>2</td></tr>
<tr><td>Sleep problems</td><td>0</td><td>1</td></tr>
<tr><td>Sore glands</td><td>0</td><td>1</td></tr>
</table>
</td>
<td>Moderate methodological quality

Moderate study size

Conflicts of interest
Research funding from and provided consultancy to pharmaceutical companies

Funding
Tobacco Advisory Group project grant, Cancer Research UK</td>
</tr>
</table>

<table>
<tr><th>Study details (author, year, location, study type, time frame)</th><th>Sample characteristics</th><th>Intervention and control</th><th>Outcome measure</th><th>Results</th><th>Quality assessment, study size, conflicts of interest, funding</th></tr>
<tr>
<td>Eisenberg et al., 2020

Canada

Multi-centre randomised controlled trial

2016-2019</td>
<td><u>Study size</u>
376 smokers

<u>Sample</u>
Current smoker who smoked a mean of 10 cigarettes or more per day

<u>Gender (male) - n (%)</u>
ENDS: 63/128 (49%)
ENNDS: 71/127 (56%)
Control: 64/121 (53%)

<u>Age - mean (SD) years</u>
ENDS: 53 (13)
ENNDS: 53 (13)
Control: 53 (12)</td>
<td><u>Intervention 1 (n=128)</u>
ENDS: 15mg/mL nicotine, and behavioural counselling

<u>Intervention 2 (n=127)</u>
ENNDS: 0mg/mL nicotine, and behavioural counselling

<u>Comparator (n=121)</u>
Counselling only

<u>Materials</u>
Rechargeable e-cigarette with prefilled, disposable, tobacco-flavoured liquid cartridges

<u>Follow-up</u>
Telephone call at weeks 1, 2, 8 and 18. Laboratory visit at weeks 4, 12, and 24</td>
<td>Serious and mild adverse events</td>
<td><u>Serious Adverse Events - n (%)</u>
<table>
<tr><th></th><th>ENDS (n=128)</th><th>ENNDS (n=127)</th><th>Control (n=121)</th></tr>
<tr><td>Participants</td><td>1 (0.8%)</td><td>4 (3.1%)</td><td>2 (1.7%)</td></tr>
<tr><td>Death</td><td>0</td><td>0</td><td>0</td></tr>
<tr><td>Respiratory</td><td>1 (0.8%)</td><td>0</td><td>0</td></tr>
<tr><td>Cardiovascular</td><td>0</td><td>1 (0.8%)</td><td>1 (0.8%)</td></tr>
<tr><td>Neuropsychiatric</td><td>0</td><td>0</td><td>0</td></tr>
<tr><td>Other</td><td>0</td><td>3 (2.4%)</td><td>1 (0.8%)</td></tr>
</table>
<u>Mild Adverse Events - n (%)</u>
<table>
<tr><th></th><th>ENDS (n=128)</th><th>ENNDS (n=127)</th><th>Control (n=121)</th></tr>
<tr><td>Participants</td><td>120 (94%)</td><td>118 (93%)</td><td>88 (73%)</td></tr>
<tr><td>Cough</td><td>95 (74%)</td><td>81 (64%)</td><td>66 (55%)</td></tr>
<tr><td>Dry mouth</td><td>72 (56%)</td><td>74 (58%)</td><td>55 (46%)</td></tr>
<tr><td>Headache</td><td>70 (55%)</td><td>69 (54%)</td><td>46 (38%)</td></tr>
<tr><td>Rhinitis</td><td>70 (55%)</td><td>67 (53%)</td><td>51 (42%)</td></tr>
<tr><td>Throat irritation</td><td>70 (55%)</td><td>53 (42%)</td><td>30 (25%)</td></tr>
<tr><td>Dyspnoea</td><td>53 (41%)</td><td>61 (48%)</td><td>43 (36%)</td></tr>
<tr><td>Sore throat</td><td>44 (34%)</td><td>39 (31%)</td><td>21 (17%)</td></tr>
<tr><td>Light headedness</td><td>42 (33%)</td><td>34 (27%)</td><td>28 (23%)</td></tr>
<tr><td>Dizziness</td><td>39 (31%)</td><td>31 (24%)</td><td>37 (31%)</td></tr>
<tr><td>Mouth irritation</td><td>38 (30%)</td><td>24 (19%)</td><td>15 (12%)</td></tr>
<tr><td>Nausea</td><td>37 (29%)</td><td>30 (24%)</td><td>20 (17%)</td></tr>
<tr><td>Indigestion</td><td>31 (24%)</td><td>33 (26%)</td><td>28 (23%)</td></tr>
<tr><td>Mouth ulcers</td><td>19 (15%)</td><td>16 (13%)</td><td>7 (6%)</td></tr>
<tr><td>Vertigo</td><td>16 (13%)</td><td>11 (9%)</td><td>9 (7%)</td></tr>
</table></td>
<td>High methodological quality

Moderate study size

<u>Conflict of interest</u>
Grants and compensation from pharmaceutical companies

<u>Funding</u>
Canadian Institutes of Health Research</td>
</tr>
</table>

<table>
<tr><th>Study details (author, year, location, study type, time frame)</th><th>Sample characteristics</th><th>Intervention and control</th><th>Outcome measure</th><th>Results</th><th>Quality assessment, study size, conflicts of interest, funding</th></tr>
<tr>
<td>Hajek et al., 2019

UK

Two-group, pragmatic, multi-centre, individually randomised, controlled trial

2015-2018</td>
<td><u>Study size</u>
886 smokers

<u>Sample</u>
Adult smokers attending UK National Health Service stop-smoking services

<u>Gender (N=884) - n (%)</u>
Male: 460/884 (52%)
Female: 424/884 (48%)

<u>Age - median (IQR) years</u>
41 (33-52)</td>
<td><u>Intervention (n=438)</u>
ENDS and behavioural support including weekly one-on-one session with local clinicians

<u>Comparator (n=446)</u>
Nicotine replacement therapies (NRTs) and behavioural support including weekly one-on-one session with local clinicians

<u>Materials</u>
ENDS: nicotine 18mg/mL
NRTs: range of nicotine replacement products

<u>Follow-up</u>
52 weeks, phone call at 26 and 52 weeks and trial visit at 52 weeks</td>
<td>Adverse events</td>
<td><u>Respiratory symptoms at baseline and 52 weeks - n (%)</u>
<table>
<tr><th></th><th>ENDS (n=315)</th><th></th><th>NRTs (n=279)</th><th></th><th>Relative risk (95% CI)</th></tr>
<tr><th></th><th>Baseline</th><th>52 weeks</th><th>Baseline</th><th>52 weeks</th><th></th></tr>
<tr><td>Shortness of breath</td><td>120 (38.1%)</td><td>66 (21.0%)</td><td>92 (33.0%)</td><td>64 (22.9%)</td><td>0.9 (0.7-1.1)</td></tr>
<tr><td>Wheezing</td><td>102 (32.4%)</td><td>74 (23.5%)</td><td>86 (30.8%)</td><td>59 (21.1%)</td><td>1.1 (0.8-1.4)</td></tr>
<tr><td>Cough</td><td>173 (54.9%)</td><td>97 (30.8%)</td><td>144 (51.6%)</td><td>111 (39.8%)</td><td>0.8 (0.6-0.9)</td></tr>
<tr><td>Phlegm</td><td>137 (43.5%)</td><td>79 (25.1%)</td><td>121 (43.4%)</td><td>103 (36.9%)</td><td>0.7 (0.6-0.9)</td></tr>
</table>
<u>Serious Adverse Events - n</u>
ENDS: 27
NRT: 22

No serious adverse event in either group was classified by the trial clinician as being related to product use</td>
<td>Moderate methodological quality

Moderate study size

<u>Conflicts of interest</u>
Grants and personal fees from pharmaceutical companies outside current study

<u>Funding</u>
National Institute for Health Research and Cancer Research UK Prevention Trials Unit</td>
</tr>
<tr>
<td>Holliday et al., 2019

UK

Single-centre, two-arm, parallel group, individually randomised controlled pilot trial

2016-2017</td>
<td><u>Study size</u>
80 smokers

<u>Sample</u>
Smoker of burnt tobacco (≥10 factory-made CPD or 7g loose tobacco/day or 14 hand-rolled CPD), diagnosed with periodontitis

<u>Gender - n (%)</u>
Male: 38/80 (47.5%)
Female: 42/80 (52.5%)

<u>Age - mean (SD) years</u>
44.3 (10.7)</td>
<td><u>Intervention (n=40)</u>
ENDS

<u>Comparator (n=40)</u>
Usual care (behavioural therapy)

<u>Materials</u>
ENDS: Vype eTank clearomiser (tank), Flavour options: Blended Tobacco, Crisp Mint, Dark Cherry and Vpure (flavourless)*. Nicotine strength concentrations: 0mg/mL, 6mg/mL, 12mg/mL, 18mg/mL

<u>Follow-up</u>
6 months</td>
<td>Dental adverse events</td>
<td><u>Dental adverse events - n</u>
<table>
<tr><th></th><th>Control</th><th></th><th>ENDS</th><th></th></tr>
<tr><th></th><th>AEs (n)</th><th>No. participants</th><th>AEs (n)</th><th>No. participants</th></tr>
<tr><td>Toothache</td><td>4</td><td>4</td><td>11</td><td>9</td></tr>
<tr><td>Dentine hypersensitivity</td><td>3</td><td>3</td><td>3</td><td>3</td></tr>
<tr><td>Tooth/teeth loss</td><td>5 (6 teeth)</td><td>4</td><td>5 (9 teeth)</td><td>3</td></tr>
<tr><td>Dental/ periodontal abscess</td><td>2</td><td>2</td><td>3</td><td>3</td></tr>
<tr><td>Mouth ulceration</td><td>0</td><td>0</td><td>2</td><td>2</td></tr>
<tr><td>Soreness of intra-oral soft tissue</td><td>0</td><td>0</td><td>3</td><td>3</td></tr>
<tr><td>Fractured/carious filling or tooth</td><td>3</td><td>3</td><td>2</td><td>2</td></tr>
<tr><td>Other</td><td>3</td><td>2</td><td>6</td><td>5</td></tr>
</table></td>
<td>Moderate methodological quality

Small study size

<u>Conflicts of interest</u>
None declared

<u>Funding</u>
National Institute for Health Research</td>
</tr>
</table>

<table>
<tr><th>Study details (author, year, location, study type, time frame)</th><th>Sample characteristics</th><th>Intervention and control</th><th>Outcome measure</th><th>Results</th><th>Quality assessment, study size, conflicts of interest, funding</th></tr>
<tr><td>Lee et al., 2019

Korea

Single-centre, prospective, open-label, randomised controlled, clinical pilot trial

2012</td><td><u>Study size</u>
150 smokers

<u>Sample</u>
Current smoker who smoked at least 10 CPD during the preceding year, had smoked for at least 3 years

<u>Gender - n (%)</u>
Male: 150/150 (100%)
Female: 0/150 (0%)

<u>Age - mean (SD) years</u>
42.3 (8.3)</td><td><u>Intervention (n=75)</u>
ENDS: 16mg/mL nicotine

<u>Comparator (n=75)</u>
Nicotine gum

<u>Materials</u>
ENDS: eGO-C Ovale, nicotine 0.01mg/mL; Janty-Korea Co.
Gum: Nicoman, nicotine 2mg/tablet

<u>Follow-up</u>
Laboratory visits at 12 and 24 weeks</td><td>Tolerability</td><td><u>Adverse events - n (%)</u>
ENDS: 5/75 (6.7%)
Gum: 13/75 (17.3%)
p=0.044

<u>Frequency of adverse events - n (%)</u>
<table>
<tr><th></th><th>ENDS</th><th>Gum</th><th>P</th></tr>
<tr><td>Subjects with any AE</td><td>5 (6.7%)</td><td>13 (7.3%)</td><td>0.044</td></tr>
<tr><td>Total AEs</td><td>9 (100%)</td><td>27 (100%)</td><td>-</td></tr>
<tr><td>Sore throat</td><td>-</td><td>2 (7.4%)</td><td>0.497</td></tr>
<tr><td>Oral pain</td><td>2 (22.2%)</td><td>5 (18.5%)</td><td>0.442</td></tr>
<tr><td>Cough</td><td>3 (3.33%)</td><td>3 (11.1%)</td><td>1.000</td></tr>
<tr><td>Dry mouth</td><td>2 (22.2%)</td><td>2 (7.4%)</td><td>1.000</td></tr>
<tr><td>Oral ulcer</td><td>-</td><td>-</td><td>-</td></tr>
<tr><td>Dizziness</td><td>-</td><td>5 (18.5%)</td><td>0.058</td></tr>
<tr><td>Headache</td><td>1 (11.1%)</td><td>2 (7.4%)</td><td>1.000</td></tr>
<tr><td>Nausea/vomiting</td><td>1 (11.1%)</td><td>8 (29.6%)</td><td>0.034</td></tr>
<tr><td>Other</td><td>-</td><td>-</td><td>-</td></tr>
</table>
No serious adverse events were reported</td><td>Moderate methodological quality

Moderate study size

<u>Conflicts of interest</u>
None declared

<u>Funding</u>
None</td></tr>
<tr><td>Lucchiari et al., 2019

Italy

Double-blind randomised controlled trial

2015-2016</td><td><u>Study size</u>
210 smokers

<u>Sample</u>
Smoker who smoked an average of 10 cigarettes or more a day for at least the past 10 years

<u>Gender - n (%)</u>
Male: 132/210 (62.9%)
Female: 78/210 (37.1%)

<u>Age - mean (SD) years</u>
62.8 (4.58)</td><td><u>Intervention 1 (n=70)</u>
ENDS

<u>Intervention 2 (n=70)</u>
ENNDS

<u>Comparator (n=70)</u>
Counselling

<u>Materials</u>
ENDS: e-cigarette kit and 12 x 10mL liquid cartridges (8mg/mL nicotine concentration)
ENNDS: e-cigarette kit and 12 x 0mL liquid cartridges (8mg/mL nicotine concentration)

<u>Follow-up</u>
3 and 6 months</td><td>Adverse events</td><td><u>Adverse events at 3 and 6 months (%)</u>
<table>
<tr><th></th><th colspan="2">3 months</th><th colspan="2">6 months</th></tr>
<tr><th></th><th>ENDS</th><th>ENNDS</th><th>ENDS</th><th>ENNDS</th></tr>
<tr><td>Burning throat</td><td>5.7%</td><td>2.9%</td><td>15.9%</td><td>5.6%</td></tr>
<tr><td>Cough</td><td>10.0%</td><td>2.9%</td><td>5.8%</td><td>2.8%</td></tr>
<tr><td>Nausea</td><td>1.4%</td><td>2.9%</td><td>5.8%</td><td>7.0%</td></tr>
<tr><td>Headache</td><td>-</td><td>-</td><td>-</td><td>1.4%</td></tr>
<tr><td>Insomnia</td><td>1.4%</td><td>-</td><td>1.4%</td><td>-</td></tr>
<tr><td>Stomach ache</td><td>-</td><td>-</td><td>4.3%</td><td>4.2%</td></tr>
<tr><td>Confusion</td><td>1.4%</td><td>-</td><td>1.4%</td><td>-</td></tr>
</table></td><td>Moderate methodological quality

Moderate study size

<u>Conflicts of interest</u>
None declared

<u>Funding</u>
Fondazione Umberto Veronesi</td></tr>
</table>

Study details (author, year, location, study type, time frame)	Sample characteristics	Intervention and control	Outcome measure	Results	Quality assessment, study size, conflicts of interest, funding
Baldassarri et al., 2018<br><br>US<br><br>Double-blinded, randomised controlled trial<br><br>Study date not reported	Study size<br>40 smokers<br><br>Sample<br>Current smokers: smoking 1 or more CPD<br><br>Gender male - n (%)<br>ENDS + patch: 12/20 (60%)<br>ENNDS + patch: 7/20 (35%)<br>Total: 19/40 (48%)<br><br>Age - mean (SD) years<br>ENDS + patch: 52.2 (12.2)<br>ENNDS + patch: 53.8 (7.8)<br>Total: 53.0 (10.1)	Intervention (n=20)<br>ENDS: 24mg/mL nicotine, nicotine patch and counselling<br><br>Comparator (n=21)<br>ENNDS, nicotine patch and counselling<br><br>Materials<br>2nd generation eGO style device (650 mAh battery, EVOD clearomiser, 3.7V, 1.8Ω single bottom coil), e-liquid: 70/30 propylene glycol/vegetable glycerin, tobacco flavour)<br>Nicotine patch: 21mg or 14mg nicotine<br><br>Follow-up<br>Laboratory visits 24 weeks	Adverse events	Commonly reported side effects-all participants (%)<br>Cough: 30%<br>Sore throat: 22.5%<br>Increased appetite: 17.5%<br>Vivid dreams: 17.5%<br><br>No significant differences by treatment group	Moderate methodological quality<br><br>Small study size<br><br>Conflict of interest<br>Grants and consulting/speaking fees from pharmaceutical companies and funding as an expert witness in litigation filed against the tobacco industry<br><br>Funding<br>Yale University and the National Heart, Lung, and Blood Institute
Carpenter et al., 2017<br><br>US<br><br>Randomised controlled trial<br><br>Study date not reported	Study size<br>68 smokers<br><br>Sample<br>Current smoker of ≥5 CPD for ≥1 year<br><br>Gender - male (%)<br>ENDS 16mg: 28%<br>ENDS 24mg: 57%<br>Control: 36%<br><br>Age - mean (SD) years<br>ENDS 16mg: 43.3 (14.4)<br>ENDS 24mg: 40.9 (12.3)<br>Control: 42.3 (14.2)	Intervention 1 (n=25)<br>ENDS: 16mg/mL nicotine<br><br>Intervention 2 (n=21)<br>ENNDS: 24mg/mL nicotine<br><br>Comparator (n=22)<br>No intervention<br><br>Materials<br>Blu Starter Pack or BluPlus+, traditional tobacco or menthol flavour<br><br>Follow-up<br>Laboratory visits at 8, 12, 16 weeks	Adverse events	Total number of Adverse Events - % participants, number of AEs<br>ENDS 16mg: 36%, 17 AEs<br>ENDS 24mg: 52%, 21 AEs<br>Control: none<br><br>Adverse Events (%) - both ENDS groups<br>Cough: 32%<br>Nausea: 24%<br>Mouth/throat irritation: 16%<br><br>Adverse Events (%) - control<br>Headache: 24%<br>Cough: 21%<br>Mouth/throat irritation: 17%	Low methodological quality<br><br>Small study size<br><br>Conflict of interest<br>Consultant/advisory board members for and grants from pharmaceutical companies and expert witness testimony against cigarette manufacturers<br><br>Funding<br>Not reported
Cohort studies					

<table>
<tr><th>Study details (author, year, location, study type, time frame)</th><th>Sample characteristics</th><th>Intervention and control</th><th>Outcome measure</th><th>Results</th><th>Quality assessment, study size, conflicts of interest, funding</th></tr>
<tr>
<td>Walele et al., 2018

UK

Prospective cohort study

Study date not reported</td>
<td><u>Study size</u>
209 smokers

<u>Sample</u>
Healthy smokers (5-30 CPD for at least one year), aged between 21 and 65 years, BMI 18-35kg/m^2, all from a previous randomised controlled trial (only compliant participants included)

<u>Gender - n (%)</u>
Male: 115/209 (55%)
Female: 94/209 (45%)

<u>Age - mean (SD) years</u>
36.6 (10.2)</td>
<td><u>Intervention (n=209)</u>
ENDS: 1.6% (16mg/g) nicotine Puritane™ device, in tobacco or menthol flavour

<u>Comparator</u>
None

<u>Materials</u>
Puritane™ (closed system ENDS)

<u>Follow-up</u>
Two years</td>
<td>Adverse events (AEs)

Serious adverse events (SAEs)

Analysed as whole sample, and subgroups:

‘EVP-compliant’ - abstinent from conventional cigarettes for at least 80% of the completed study days

‘Completers’ - completed the study</td>
<td><table>
<tr><th></th><th>All subjects (n=209)</th><th>EVP-compliant subjects (n=110)</th><th>Completers (n=102)</th></tr>
<tr><td>Total</td><td>971 (100%)</td><td>575 (100%)</td><td>640 (100%)</td></tr>
<tr><td>SAEs</td><td>7 (0.7%)</td><td>3 (0.5%)</td><td>1 (0.2%)</td></tr>
<tr><td>AEs leading to study withdrawal</td><td>11 (1.1%)</td><td>6 (1.0%)</td><td>0</td></tr>
<tr><td colspan="4">AEs by severity (% of AEs)</td></tr>
<tr><td>Mild</td><td>323 (33.3%)</td><td>222 (38.6%)</td><td>236 (36.9%)</td></tr>
<tr><td>Moderate</td><td>503 (51.8%)</td><td>292 (50.8%)</td><td>318 (49.7%)</td></tr>
<tr><td>Severe</td><td>145 (14.9%)</td><td>61 (10.6%)</td><td>86 (13.4%)</td></tr>
<tr><td colspan="4">AEs by relationship to study product (% of AEs)</td></tr>
<tr><td>Almost definitely related</td><td>11 (1.1%)</td><td>7 (1.2%)</td><td>3 (0.5%)</td></tr>
<tr><td>Probably related</td><td>32 (3.3%)</td><td>27 (4.7%)</td><td>17 (2.7%)</td></tr>
<tr><td>Possibly related</td><td>401 (41.3%)</td><td>192 (33.4%)</td><td>259 (40.5%)</td></tr>
<tr><td>Unlikely related</td><td>207 (21.3%)</td><td>114 (19.8%)</td><td>122 (19.1%)</td></tr>
<tr><td>Unrelated</td><td>320 (33.0%)</td><td>235 (40.9%)</td><td>239 (37.3%)</td></tr>
</table></td>
<td>Low methodological quality

Moderate study size

<u>Conflicts of interest</u>
Personal fees or ‘other’ from Fontem Ventures and/or the tobacco and pharmaceutical industries

<u>Funding</u>
Funded and supported by Fontem Ventures (parent company is Imperial Brands Group)</td>
</tr>
<tr>
<td>Polosa et al., 2017

Italy

Prospective cohort study

Online survey, regular vape shop customers

2013-2017</td>
<td><u>Study size</u>
31 never smokers enrolled, 21 included in analysis

<u>Sample</u>
Never smokers or <100 cigarettes smoked in lifetime, daily e-cigarette users for ≥3 months

<u>Gender - n (%)</u>
Male: 21/31 (68%)
Female: 10/31 (32%)

<u>Age - mean (SD) years</u>
ENDS: 29.7 (6.1)
Control: 32.5 (7.0)</td>
<td><u>Exposure (n=9)</u>
Daily e-liquid consumption - median (range): 4mL (2-5)

<u>Comparator (n=12)</u>
Non-smoker and non-e-cigarette user

<u>Materials - device type</u>
Advanced refillable: 44%
Standard refillable: 56%

<u>Materials - nicotine concentration (%)</u>
0%: 33
0.9%: 22
1.2%: 22
1.6%: 11
1.8%: 11

<u>Follow-up</u>
Follow-up at 12, 24 and 42 months</td>
<td><u>Self-reported adverse events at baseline and each study visit</u>
Cough, wheeze, shortness of breath, tight chest</td>
<td>None of the participants in this study reported any wheezing, shortness of breath, or chest tightness. Cough was reported by one e-cigarette user at baseline and by another at second follow-up. In the control group, three participants reported cough on three separate occasions. Of note, study participants reported no severe adverse reactions.</td>
<td>Moderate methodological quality

Very small study size

<u>Conflicts of interest</u>
Grants and consulting/speaking fees from pharmaceutical companies and electronic cigarette industry and trade associations

<u>Funding</u>
Supported by Catania University</td>
</tr>
</table>

Percentages and p-values are presented as reported in original studies.
AE = adverse event; BMI = body mass index; CI = confidence interval; CPD = cigarette(s) per day; ENDS = electronic nicotine delivery system; ENNDS = electronic non-nicotine delivery system; EVP = electronic vaping product; IQR = interquartile range; NRT = nicotine replacement therapy; SAE = serious adverse event; SD = standard deviation; UK = United Kingdom; US = United States.

Table 11.2. Study details: less serious adverse events - surveillance reports

Study details (author, year, country, time frame, data source)	Demographics (sample size, sex, age)	Exposure (details of device)	Presentation	Treatment	Outcome	Quality assessment, conflict of interest, funding
Motooka et al., 2018<br><br>US<br><br>2004-2016<br><br>Food and Drug Administration Adverse Event Reporting System database	N=27<br><br>Sex: unknown<br><br>Age: unknown	Not reported	Adverse events (n)<br>Dizziness: 4<br>Dyspnoea: 4<br>Nausea: 2<br>Chest pain: 2<br>Increased heart rate: 2<br>Tremor: 2<br>Disorientation: 2<br>Cough: 2<br>Wheezing: 2<br>Thermal burn: 1<br>Pulmonary edema: 1<br>Throat irritation: 1<br>Altered visual depth perception: 1<br>Chills: 1<br>Device component issue: 1<br>Device deposit issue: 1<br>Device malfunction: 2<br>Device physical property issue: 1<br>Fear: 1<br>Headache: 1<br>Insomnia: 1<br>Lung disorder: 1<br>Malaise: 1<br>Migraine: 1<br>Pain: 2<br>Product label issue: 1<br>Productive cough: 1<br>Panic reaction: 1<br>Sensation of heaviness: 1<br>VIIth nerve paralysis: 1	Not reported	Not reported	Low methodological quality<br><br>Very small study size<br><br>Conflicts of interest<br>Author is an employee of Micron Inc (technology company)<br><br>Funding<br>Japan Society for the Promotion of Science

Percentages and p-values are presented as reported in original studies.
US = United States.

Table 12.1. Study details: optical health – non-randomised intervention studies

<table>
<tr><th>Study details (author, year, location, study design)</th><th>Sample characteristics</th><th>Exposure/ Comparison groups</th><th>Outcome measure</th><th>Results</th><th>Quality assessment study size, conflict of interest, funding</th></tr>
<tr>
<td>Munsamy et al., 2019

South Africa

Non-randomised, pre-post study

Study date not reported</td>
<td><u>Study size</u>
64 enrolled, 58 analysed

<u>Sample</u>
E-cigarette naïve subjects

<u>Gender - n (%)</u>
Male: 43/64 (67%)
Female: 21/64 (33%)

<u>Age mean (years)</u>
21

<u>Setting</u>
Designated smoker area (4.67m by 2.25m), air-conditioning turned off</td>
<td><u>Exposure - dose</u>
0.05mL of 8mg/mL nicotine containing e-liquid

<u>Comparator</u>
Within subject

<u>Materials</u>
Not specified

<u>Pattern of use</u>
10 puffs</td>
<td>Corneal epithelial thickness (microns) of the 5 zones: central, superior, inferior, nasal and temporal

Tear film stability (seconds) measured by Non-Invasive Keratograph Break-Up Time (NIKBUT)</td>
<td><u>Mean change for corneal epithelial thickness, n=58 (microns)</u>
<table>
<tr><th></th><th>Pre</th><th>Post</th><th>Mean change</th><th>SD</th><th>P</th></tr>
<tr><td>Central</td><td>52.44</td><td>52.76</td><td>-0.3448</td><td>1.5955</td><td>0.105</td></tr>
<tr><td>Superior</td><td>52.38</td><td>52.56</td><td>-0.2414</td><td>1.5138</td><td>0.230</td></tr>
<tr><td>Inferior</td><td>52.97</td><td>53.19</td><td>-0.2931</td><td>1.6005</td><td>0.169</td></tr>
<tr><td>Nasal</td><td>52.63</td><td>52.81</td><td>-0.2069</td><td>1.4112</td><td>0.269</td></tr>
<tr><td>Temporal</td><td>51.64</td><td>51.87</td><td>-0.2759</td><td>1.3218</td><td>0.117</td></tr>
</table>
All the mean changes for corneal epithelial thickness were statistically insignificant

<u>Tear film stability, n=57 (seconds)</u>
<table>
<tr><th></th><th>Pre</th><th>Post</th><th>Mean change</th><th>SD</th><th>P</th></tr>
<tr><td>Pre Post</td><td>12.72</td><td>14.12</td><td>-1.40</td><td>6.11</td><td>0.089</td></tr>
</table>
Negative reading implies an increase, therefore non-significant increase in tear film stability</td>
<td>Moderate methodological quality

Very small study size

<u>Conflicts of interest</u>
None declared

<u>Funding</u>
Not reported</td>
</tr>
</table>

Percentages and p-values are presented as reported in original studies.
ENIKBUT = Non-Invasive Keratograph Break-up Time; SD = standard deviation.

13. Olfactory outcomes

Table 13.1. Study details: olfactory outcomes – cross-sectional surveys

<table>
<tr><th>Study details (author, year, study design, time frame [data source])</th><th>Sample characteristics</th><th>Exposure/ Comparison groups</th><th>Outcome measure</th><th>Results</th><th>Quality assessment, study size, conflict of interest, funding</th></tr>
<tr>
<td>Majchrzak et al., 2020

Austria

Cross-sectional

July-October 2017

Students of the University of Vienna, Vienna University of Economics and Business, vapour bars - recruited via social media, personal contacts</td>
<td><u>Study size</u>
181 participants total
Never smokers: 70
Smokers: 66
Exclusive e-cigarette: 45

<u>Sample</u>
Never smokers: non-smokers that never smoked
Smokers: no definition
Exclusive e-cigarette: ex-smokers abstinent from smoking for approximately 2 years

<u>Age - mean (SD) years</u>
Never smokers: 25.2 (5.4)
Smokers: 27.2 (5.7)
Exclusive e-cigarette: 26.8 (6.3)

<u>Gender - n (%)</u>
Never smokers: 40/70 (57%) females, 30/70 (43%) males
Smokers: 32/66 (48%) females, 34/66 (52%) males
Exclusive e-cigarette: 18/45 (40%) females, 27/45 (60%) males</td>
<td><u>Exposure - dose</u>
Average 10.8mL liquid/day for an average of 2.3 years

<u>Comparators</u>
Never smokers

<u>Materials</u>
Not specified

<u>Follow-up</u>
Used e-cigarettes approximately 2 years</td>
<td><u>Olfactory sensitivity</u>
1. Threshold test (score out of 16)

2. Discrimination test (score out of 16)

3. Identification test (score out of 16)

4. Olfactory test result - TDI (score out of 48)</td>
<td>Exclusive e-cigarette users and never smokers
<table>
<tr><th></th><th>Exclusive e-cigarette users Mean (SD)</th><th>Never smokers Mean (SD)</th><th>P</th></tr>
<tr><td>Threshold test</td><td>10.19 (1.76)</td><td>9.96 (2.03)</td><td>0.349</td></tr>
<tr><td>Pearson correlation</td><td></td><td></td><td></td></tr>
<tr><td>Years of e-cigarette use</td><td colspan="2">r = −0.099</td><td>0.517</td></tr>
<tr><td>Volume consumed (mL)</td><td colspan="2">r = −0.204</td><td>0.180</td></tr>
<tr><td>Discrimination test</td><td>11.67 (1.38)</td><td>12.73 (1.46)</td><td>≤0.001</td></tr>
<tr><td>Pearson correlation</td><td></td><td></td><td></td></tr>
<tr><td>Years of e-cigarette use</td><td>r = 0.091</td><td></td><td>0.553</td></tr>
<tr><td>Volume consumed (mL)</td><td>r = −0.013</td><td></td><td>0.932</td></tr>
<tr><td>Identification test</td><td>11.34 (1.44)</td><td>12.06 (1.82)</td><td>0.033</td></tr>
<tr><td>Pearson correlation</td><td></td><td></td><td></td></tr>
<tr><td>Years of e-cigarette use</td><td>r = −0.075</td><td></td><td>0.626</td></tr>
<tr><td>Volume consumed (mL)</td><td>r = −0.038,</td><td></td><td>0.803</td></tr>
<tr><td>TDI-score</td><td>33.20 (2.23)</td><td>34.74 (3.60)</td><td><0.05</td></tr>
</table>
Exclusive e-cigarette users and smokers
<table>
<tr><th></th><th>Exclusive e-cigarette users Mean (SD)</th><th>Smokers Mean (SD)</th><th>P</th></tr>
<tr><td>Threshold test</td><td>10.19 (1.76)</td><td>6.07 (1.36)</td><td>≤0.001</td></tr>
<tr><td>Discrimination test</td><td>11.67 (1.38)</td><td>9.23 (1.38)</td><td>≤0.001</td></tr>
<tr><td>Identification test</td><td>11.34 (1.44)</td><td>10.53 (1.38)</td><td>0.001</td></tr>
<tr><td>TDI-score</td><td>33.20 (2.23)</td><td>25.83 (2.26)</td><td><0.05</td></tr>
</table>
</td>
<td>Moderate methodological quality

Small study size

<u>Conflicts of interest</u>
None declared

<u>Funding</u>
No specific funding</td>
</tr>
</table>

Percentages and p-values are presented as reported in original studies.
SD = standard deviation; TDI = threshold-discrimination-identification.

4. Studies in combined evidence synthesis - systematic umbrella and top-up review

Dependence and Abuse liability (52 studies)

Meta-analyses

No studies identified

Randomised controlled trials (13) [1]

1. Adriaens K, Van Gucht D, Baeyens F. IQOS™ vs. e-cigarette vs. tobacco cigarette: a direct comparison of short-term effects after overnight-abstinence. *Int J Environ Res Public Health* 2018; 15: 2902.
2. De La Garza R, Shuman SL, Yammine L, et al. A pilot study of e-cigarette naïve cigarette smokers and the effects on craving after acute exposure to e-cigarettes in the laboratory. *Am J Addict* 2019; 28: 361-366.
3. Hiler M, Breland A, Spindle T, et al. Electronic cigarette user plasma nicotine concentration, puff topography, heart rate, and subjective effects: influence of liquid nicotine concentration and user experience. *Exp Clin Psychopharmacol* 2017; 25: 380-392.
4. Meier E, Wahlquist AE, Heckman BW, et al. A pilot randomized crossover trial of electronic cigarette sampling among smokers. *Nicotine Tob Res* 2017; 19: 176-182.
5. O'Connell G, Pritchard JD, Prue C, et al. A randomised, open-label, cross-over clinical study to evaluate the pharmacokinetic profiles of cigarettes and e-cigarettes with nicotine salt formulations in US adult smokers. *Intern Emerg Med* 2019; 14: 853-861.
6. Palmer AM, Brandon TH. How do electronic cigarettes affect cravings to smoke or vape? Parsing the influences of nicotine and expectancies using the balanced-placebo design. *J Consult Clin Psychol* 2018; 86: 486-491.
7. Rosbrook K, Green BG. Sensory effects of menthol and nicotine in an e-cigarette. *Nicotine Tob Res* 2016; 18: 1588-1595.
8. Steinberg MB, Zimmermann MH, Delnevo CD, et al. E-cigarette versus nicotine inhaler: comparing the perceptions and experiences of inhaled nicotine devices. *J Gen Intern Med* 2014; 29: 1444-1450.
9. Stiles MF, Campbell LR, Graff DW, et al. Pharmacodynamic and pharmacokinetic assessment of electronic cigarettes, combustible cigarettes, and nicotine gum: implications for abuse liability. *Psychopharmacology* 2017; 234: 2643-2655.
10. Stiles MF, Campbell LR, Jin T, et al. Assessment of the abuse liability of three menthol Vuse Solo electronic cigarettes relative to combustible cigarettes and nicotine gum. *Psychopharmacology* 2018; 235: 2077-2086.
11. Strasser AA, Souprountchouk V, Kaufmann A, et al. Nicotine replacement, topography, and smoking phenotypes of e-cigarettes. *Tob Regul Sci* 2016; 2: 352-362.
12. Vansickel AR, Weaver MF, Eissenberg T. Clinical laboratory assessment of the abuse liability of an electronic cigarette. *Addiction* 2012; 107: 1493-1500.

Cohort studies (1)

1. Du P, Fan TY, Yingst J, et al. Changes in e-cigarette use behaviors and dependence in long-term e-cigarette users. *Am J Prev Med* 2019; 57: 374-383.

Non-randomised intervention studies (17)

1. Audrain-McGovern J, Strasser AA, Wileyto EP. The impact of flavoring on the rewarding and reinforcing value of e-cigarettes with nicotine among young adult smokers. *Drug Alcohol Depend* 2016; 166: 263-267.

[1] Described two separate randomised controlled trials both of which are accounted for in table count

2. Baldassarri SR, Hillmer AT, Anderson JM, et al. Use of electronic cigarettes leads to significant beta2-nicotinic acetylcholine receptor occupancy: evidence from a PET imaging study. *Nicotine Tob Res* 2018; 20: 425-433.
3. Cobb CO, Lopez AA, Soule EK, et al. Influence of electronic cigarette liquid flavors and nicotine concentration on subjective measures of abuse liability in young adult cigarette smokers. *Drug Alcohol Depend* 2019; 203: 27-34.
4. Dawkins LE, Kimber CF, Doig M, et al. Self-titration by experienced e-cigarette users: blood nicotine delivery and subjective effects. *Psychopharmacology* 2016; 233: 2933-2941.
5. Dowd AN, Tiffany ST. Comparison of tobacco and electronic cigarette reward value measured during a cue-reactivity task: an extension of the choice behavior under cued conditions procedure. *Nicotine Tob Res* 2019; 21: 1394-1400.
6. Goldenson NI, Kirkpatrick MG, Barrington-Trimis JL, et al. Effects of sweet flavorings and nicotine on the appeal and sensory properties of e-cigarettes among young adult vapers: application of a novel methodology. *Drug Alcohol Depend* 2016; 168: 176-180.
7. Hobkirk AL, Nichols TT, Foulds J, et al. Changes in resting state functional brain connectivity and withdrawal symptoms are associated with acute electronic cigarette use. *Brain Res Bull* 2018; 138: 56-63.
8. Hughes JR, Peters EN, Callas PW, et al. Withdrawal symptoms from e-cigarette abstinence among former smokers: a pre-post clinical trial. *Nicotine Tob Res* 2020; 22: 734-739.
9. Hughes JR, Peters EN, Callas PW, et al. Withdrawal symptoms from e-cigarette abstinence among adult never-smokers: a pilot experimental study. *Nicotine Tob Res* 2020; 22: 740-746.
10. Maloney SF, Breland A, Soule EK, et al. Abuse liability assessment of an electronic cigarette in combustible cigarette smokers. *Exp Clin Psychopharmacol* 2019; 27: 443-454.
11. Nichols TT, Foulds J, Yingst JM, et al. Cue-reactivity in experienced electronic cigarette users: novel stimulus videos and a pilot fMRI study. *Brain Research Bull* 2016; 123: 23-32.
12. Perkins KA, Karelitz JL, Michael VC. Reinforcement enhancing effects of acute nicotine via electronic cigarettes. *Drug Alcohol Depend* 2015; 153: 104-108.
13. Rüther T, Hagedorn D, Schiela K, et al. Nicotine delivery efficiency of first- and second-generation e-cigarettes and its impact on relief of craving during the acute phase of use. *Int J Hyg Environ Health* 2018; 221: 191-198.
14. Spindle TR, Talih S, Hiler MM, et al. Effects of electronic cigarette liquid solvents propylene glycol and vegetable glycerin on user nicotine delivery, heart rate, subjective effects, and puff topography. *Drug Alcohol Depend* 2018; 188: 193-199.
15. St.Helen G, Dempsey DA, Havel CM, et al. Impact of e-liquid flavors on nicotine intake and pharmacology of e-cigarettes. *Drug Alcohol Depend* 2017; 178: 391-398.
16. St.Helen G, Nardone N, Addo N, et al. Differences in nicotine intake and effects from electronic and combustible cigarettes among dual users. *Addiction* 2020; 115: 757-767.
17. Vansickel AR, Cobb CO, Weaver MF, et al. A clinical laboratory model for evaluating the acute effects of electronic "cigarettes": nicotine delivery profile and cardiovascular and subjective effects. *Cancer Epidemiol Biomarkers Prev* 2010; 19: 1945-1953.

Case-control studies

No studies identified

Surveillance reports

No studies identified

Cross-sectional surveys (21)

1. Boykan R, Goniewicz ML, Messina CR. Evidence of nicotine dependence in adolescents who use Juul and similar pod devices. *Int J Environ Res Public Health* 2019; 16: 2135.
2. Browne M, Todd DG. Then and now: consumption and dependence in e-cigarette users who formerly smoked cigarettes. *Addict Behav* 2018; 76: 113-121.

3. Camara-Medeiros A, Diemert L, O'Connor S, et al. Perceived addiction to vaping among youth and young adult regular vapers. *Tob Control* 2021; 30: 273-278.
4. Case K, Mantey D, Creamer M, et al. E-cigarette-specific symptoms of nicotine dependence among Texas adolescents. *Addict Behav* 2018; 84: 57-61.
5. Dawkins L, Turner J, Roberts A, et al. 'Vaping' profiles and preferences: an online survey of electronic cigarette users. *Addiction* 2013; 108: 1115-1125.
6. Etter JF, Eissenberg T. Dependence levels in users of electronic cigarettes, nicotine gums and tobacco cigarettes. *Drug Alcohol Depend* 2015; 147: 68-75.
7. Etter JF. Explaining the effects of electronic cigarettes on craving for tobacco in recent quitters. *Drug Alcohol Depend* 2015; 148: 102-108.
8. Etter JF. Throat hit in users of the electronic cigarette: an exploratory study. *Psychol Addict Behav* 2016; 30: 93-100.
9. Farsalinos KE, Romagna G, Tsiapras D, et al. Evaluating nicotine levels selection and patterns of electronic cigarette use in a group of "vapers" who had achieved complete substitution of smoking. *Subst Abuse* 2013; 7: 139-146.
10. Foulds J, Veldheer S, Yingst JM, et al. Development of a questionaire for assessing dependence on electronic cigarettes among a large sample of ex-smoking e-cigarette users. *Nicotine Tob Res* 2015; 17: 186-192.
11. González-Roz A, Secades-Villa R, Weidberg S. Evaluating nicotine dependence levels in e-cigarette users. *Adicciones* 2017; 29: 136-138.
12. Hughes JR, Callas PW. Prevalence of withdrawal symptoms from electronic cigarette cessation: a cross-sectional analysis of the US Population Assessment of Tobacco and Health. *Addict Behav* 2019; 91: 234-237.
13. Jankowski M, Krzystanek M, Zejda JE, et al. E-cigarettes are more addictive than traditional cigarettes-a study in highly educated young people. *Int J Environ Res Public Health* 2019; 16: 2279.
14. Johnson JM, Muilenburg JL, Rathbun SL, et al. Elevated nicotine dependence scores among eletronic cigarette users at an electronic cigarette convention. *J Community Health* 2018; 43: 164-174.
15. Leavens ELS, Smith TT, Natale N, et al. Electronic cigarette dependence and demand among pod mod users as a function of smoking status. *Psychol Addict Behav* 2020; 34: 804-810.
16. Liu G, Wasserman E, Kong L, et al. A comparison of nicotine dependence among exclusive e-cigarette and cigarette users in the PATH study. *Prev Med* 2017; 104: 86-91.
17. Morean M, Krishnan-Sarin S, O'Malley S. Assessing nicotine dependence in adolescent e-cigarette users: the 4-Item Patient-Reported Outcomes Measurement Information System (PROMIS) Nicotine Dependence Item Bank for electronic cigarettes. *Drug Alcohol Depend* 2018; 188: 60-63.
18. Rostron BL, Schroeder MJ, Ambrose BK. Dependence symptoms and cessation intentions among US adult daily cigarette, cigar, and e-cigarette users, 2012-2013. *BMC Public Health* 2016; 16: 814.
19. Shiffman S, Sembower MA. Dependence on e-cigarettes and cigarettes in a cross-sectional study of US adults. *Addiction* 2020; 115: 1924-1931.
20. Strong DR, Pearson J, Ehlke S, et al. Indicators of dependence for different types of tobacco product users: descriptive findings from Wave 1 (2013-2014) of the Population Assessment of Tobacco and Health (PATH) Study. *Drug Alcohol Depend* 2017; 178: 257-266.
21. Yingst JM, Veldheer S, Hrabovsky S, et al. Factors associated with electronic cigarette users' device preferences and transition from first generation to advanced generation devices. *Nicotine Tob Res* 2015; 17: 1242-1246.

Case series

No studies identified

Case reports

No studies identified

Cardiovascular health outcomes (21 studies)

Meta-analyses (1)

1. Skotsimara G, Antonopoulos AS, Oikonomou E, et al. Cardiovascular effects of electronic cigarettes: a systematic review and meta-analysis. *Eur J Prev Cardiol* 2019; 26: 1219-1228.

Randomised controlled trials (11)

1. Antoniewicz L, Brynedal A, Hedman L, et al. Acute effects of electronic cigarette inhalation on the vasculature and the conducting airways. *Cardiovasc Toxicol* 2019; 19: 441-450.
2. Chaumont M, De Becker B, Zaher W, et al. Differential effects of e-cigarette on microvascular endothelial function, arterial stiffness and oxidative stress: a randomized crossover trial. *Sci Rep* 2018; 8: 10378.
3. Cooke WH, Pokhrel A, Dowling C, et al. Acute inhalation of vaporized nicotine increases arterial pressure in young non-smokers: a pilot study. *Clin Auton Res* 2015; 25: 267-270.
4. Cossio R, Cerra ZA, Tanaka H. Vascular effects of a single bout of electronic cigarette use. *Clin Exp Pharmacol Physiol* 2020; 47: 3-6.
5. Fogt DL, Levi MA, Rickards CA, et al. Effects of acute vaporized nicotine in non-tobacco users at rest and during exercise. *Int J Exerc Sci* 2016; 9: 607-615.
6. Franzen KF, Willig J, Cayo Talavera S, et al. E-cigarettes and cigarettes worsen peripheral and central hemodynamics as well as arterial stiffness: a randomized, double-blinded pilot study. *Vasc Med* 2018; 23: 419-425.
7. Ikonomidis I, Katogiannis K, Kostelli G, et al. Effects of electronic cigarette on platelet and vascular function after four months of use. *Food Chem Toxicol* 2020; 141: 111389.
8. Kerr DM, Brooksbank KJ, Taylor RG, et al. Acute effects of electronic and tobacco cigarettes on vascular and respiratory function in healthy volunteers: a cross-over study. *J Hypertens* 2019; 37: 154-166.
9. Moheimani RS, Bhetraratana M, Peters KM, et al. Sympathomimetic effects of acute e-cigarette use: role of nicotine and non-nicotine constituents. *J Am Heart Assoc* 2017; 6: e006579.
10. Staudt MR, Salit J, Kaner RJ, et al. Altered lung biology of healthy never smokers following acute inhalation of e-cigarettes. *Respir Res* 2018; 19: 78.
11. Yan XS, D'Ruiz C. Effects of using electronic cigarettes on nicotine delivery and cardiovascular function in comparison with regular cigarettes. *Regul Toxicol Pharmacol* 2015; 71: 24-34.

Cohort studies (1)

1. Polosa R, Cibella F, Caponnetto P, et al. Health impact of e-cigarettes: a prospective 3.5-year study of regular daily users who have never smoked. *Sci Rep* 2017; 7: 13825.

Non-randomised intervention studies (7)

1. Carnevale R, Sciarretta S, Violi F, et al. Acute impact of tobacco vs electronic cigarette smoking on oxidative stress and vascular function. *Chest* 2016; 150: 606-612.
2. Czogała J, Cholewiński M, Kutek A, et al. Evaluation of changes in hemodynamic parameters after the use of electronic nicotine delivery systems among regular cigarette smokers. *Przegl Lek* 2012; 69: 841-845.
3. Farsalinos KE, Tsiapras D, Kyrzopoulos S, et al. Acute effects of using an electronic nicotine-delivery device (electronic cigarette) on myocardial function: comparison with the effects of regular cigarettes. *BMC Cardiovasc Disord* 2014; 14: 78.
4. Pywell MJ, Wordsworth M, Kwasnicki RM, et al. The effect of electronic cigarettes on hand microcirculation. *J Hand Surg Am* 2018; 43: 432-438.
5. Spindle TR, Hiler MM, Breland AB, et al. The influence of a mouthpiece-based topography measurement device on electronic cigarette user's plasma nicotine concentration, heart rate,

and subjective effects under directed and ad libitum use conditions. *Nicotine Tob Res* 2017; 19: 469-476.
6. St.Helen G, Ross KC, Dempsey DA, et al. Nicotine delivery and vaping behavior during ad libitum e-cigarette access. *Tob Regul Sci* 2016; 2: 363-376.
7. Vlachopoulos C, Ioakeimidis N, Abdelrasoul M, et al. Electronic cigarette smoking increases aortic stiffness and blood pressure in young smokers. *J Am Coll Cardiol* 2016; 67: 2802-2803.

Case-control studies
No studies identified

Surveillance reports
No studies identified

Cross-sectional surveys
Not appropriate study design, not included in evidence synthesis

Case series
No studies identified

Case reports (1)
1. Shea JB, Aguilar M, Sauer WH, et al. Unintentional magnet reversion of an implanted cardiac defibrillator by an electronic cigarette. *HeartRhythm Case Rep* 2020; 6: 121-123.

Cancer (1 study)

Meta-analyses
No studies identified

Randomised controlled trials
No studies identified

Cohort studies (1)

1. Manzoli L, Flacco ME, Ferrante M, et al. Cohort study of electronic cigarette use: effectiveness and safety at 24 months. *Tob Control* 2017; 26: 284-292.

Non-randomised intervention studies
No studies identified

Case-control studies
No studies identified

Surveillance reports
No studies identified

Cross-sectional surveys
Not appropriate study design, not included in evidence synthesis

Case series
No studies identified

Case reports
Not appropriate study design, not included in evidence synthesis

Respiratory disease (58 studies)

Meta-analyses

No studies identified

Randomised controlled trials (10)

1. Antoniewicz L, Brynedal A, Hedman L, et al. Acute effects of electronic cigarette inhalation on the vasculature and the conducting airways. *Cardiovasc Toxicol* 2019; 19: 441-450.
2. Boulay M-È, Henry C, Bossé Y, et al. Acute effects of nicotine-free and flavour-free electronic cigarette use on lung functions in healthy and asthmatic individuals. *Respir Res* 2017; 18: 33.
3. Campagna D, Cibella F, Caponnetto P, et al. Changes in breathomics from a 1-year randomized smoking cessation trial of electronic cigarettes. *Eur J Clin Invest* 2016; 46: 698-706.[2]
4. Chaumont M, van de Borne P, Bernard A, et al. Fourth generation e-cigarette vaping induces transient lung inflammation and gas exchange disturbances: results from two randomized clinical trials. *Am J Physiol Lung Cell Mol Physiol* 2019; 316: L705-L719.
5. Cibella F, Campagna D, Caponnetto P, et al. Lung function and respiratory symptoms in a randomized smoking cessation trial of electronic cigarettes. *Clin Sci (Lond)* 2016; 130: 1929-1937.[3]
6. Cravo A, Bush J, Sharma G, et al. A randomised, parallel group study to evaluate the safety profile of an electronic vapour product over 12 weeks. *Regul Toxicol Pharmacol* 2016; 81 Suppl 1: S1-S14.
7. D'Ruiz CD, O'Connell G, Graff DW, et al. Measurement of cardiovascular and pulmonary function endpoints and other physiological effects following partial or complete substitution of cigarettes with electronic cigarettes in adult smokers. *Regul Toxicol Pharmacol* 2017; 87: 36-53.
8. Ferrari M, Zanasi A, Nardi E, et al. Short-term effects of a nicotine-free e-cigarette compared to a traditional cigarette in smokers and non-smokers. *BMC Pulm Med* 2015; 15: 120.
9. Kerr DM, Brooksbank KJ, Taylor RG, et al. Acute effects of electronic and tobacco cigarettes on vascular and respiratory function in healthy volunteers: a cross-over study. *J Hypertens* 2019; 37: 154-166.
10. Kumral TL, Saltürk Z, Yildirim G, et al. How does electronic cigarette smoking affect sinonasal symptoms and nasal mucociliary clearance? *B-ENT* 2016; 12: 17-21.
11. Staudt MR, Salit J, Kaner RJ, et al. Altered lung biology of healthy never smokers following acute inhalation of e-cigarettes. *Respir Res* 2018; 19: 78.

Cohort studies (6)

1. Bhatta DN, Glantz SA. Association of e-cigarette use with respiratory disease among adults: a longitudinal analysis. *Am J Prev Med* 2020; 58: 182-190.
2. Bowler RP, Hansel NN, Jacobson S, et al. Electronic cigarette use in US adults at risk for or with COPD: analysis from two observational cohorts. *J Gen Intern Med* 2017; 32: 1315-1322.
3. Polosa R, Caponnetto P, Morjaria JB, et al. Effect of an electronic nicotine delivery device (e-cigarette) on smoking reduction and cessation: a prospective 6-month pilot study. *BMC Public Health* 2011; 11: 786.
4. Polosa R, Cibella F, Caponnetto P, et al. Health impact of e-cigarettes: a prospective 3.5-year study of regular daily users who have never smoked. *Sci Rep* 2017; 7: 13825.
5. Polosa R, Morjaria J, Caponnetto P, et al. Effect of smoking abstinence and reduction in asthmatic smokers switching to electronic cigarettes: evidence for harm reversal. *Int J Environ Res Public Health* 2014; 11: 4965-4977.[4]

[2] Duplicated data, combined in evidence synthesis with Cibella et al. 2016
[3] Duplicated data, combined in evidence synthesis with Campangna et al. 2016
[4] Duplicated data, combined in evidence synthesis with Polosa et al. 2016

6. Polosa R, Morjaria JB, Caponnetto P, et al. Evidence for harm reduction in COPD smokers who switch to electronic cigarettes. *Respir Res* 2016; 17: 166.
7. Polosa R, Morjaria JB, Caponnetto P, et al. Persisting long term benefits of smoking abstinence and reduction in asthmatic smokers who have switched to electronic cigarettes. *Discov Med* 2016; 21: 99-108.[5]

Non-randomised intervention studies (8)

1. Brożek GM, Jankowski M, Zejda JE. Acute respiratory responses to the use of e-cigarette: an intervention study. *Sci Rep* 2019; 9: 6844.
2. Coppeta L, Magrini A, Pietroiusti A, et al. Effects of smoking electronic cigarettes on pulmonary function and environmental parameters. *Open Public Health J* 2018; 11: 360-368.
3. Flouris AD, Chorti MS, Poulianiti KP, et al. Acute impact of active and passive electronic cigarette smoking on serum cotinine and lung function. *Inhal Toxicol* 2013; 25: 91-101.
4. Kotoulas SC, Pataka A, Domvri K, et al. Acute effects of e-cigarette vaping on pulmonary function and airway inflammation in healthy individuals and in patients with asthma. *Respirology* 2020; 25: 1037-1045.
5. Lappas AS, Tzortzi AS, Konstantinidi EM, et al. Short-term respiratory effects of e-cigarettes in healthy individuals and smokers with asthma. *Respirology* 2018; 23: 291-297.
6. Marini S, Buonanno G, Stabile L, et al. Short-term effects of electronic and tobacco cigarettes on exhaled nitric oxide. *Toxicol Appl Pharmacol* 2014; 278: 9-15.
7. Palamidas A, Tsikrika S, Katsaounou P, et al. Acute effects of short term use of e-cigarettes on airways physiology and respiratory symptoms in smokers with and without airways obstructive diseases and in healthy non smokers. *Tob Prev Cessat* 2017; 3: 5.
8. Vardavas CI, Anagnostopoulos N, Kougias M, et al. Short-term pulmonary effects of using an electronic cigarette: impact on respiratory flow resistance, impedance, and exhaled nitric oxide. *Chest* 2012; 141: 1400-1406.

Case-control studies

No studies identified

Surveillance reports (18)

1. Adkins SH, Anderson KN, Goodman AB, et al. Demographics, substance use behaviors, and clinical characteristics of adolescents with e-cigarette, or vaping, product use-associated lung injury (EVALI) in the United States in 2019. *JAMA Pediatr* 2020; 174: e200756.
2. Armatas C, Heinzerling A, Wilken JA. Notes from the field: e-cigarette, or vaping, product use-associated lung injury cases during the COVID-19 response - California, 2020. *MMWR Morb Mortal Wkly Rep* 2020; 69: 801-802.
3. Blount BC, Karwowski MP, Morel-Espinosa M, et al. Evaluation of bronchoalveolar lavage fluid from patients in an outbreak of e-cigarette, or vaping, product use-associated lung injury - 10 states, August-October 2019. *MMWR Morb Mortal Wkly Rep* 2019; 68: 1040-1041.
4. Chatham-Stephens K, Roguski K, Jang Y, et al. Characteristics of hospitalized and nonhospitalized patients in a nationwide outbreak of e-cigarette, or vaping, product use-associated lung injury - United States, November 2019. *MMWR Morb Mortal Wkly Rep* 2019; 68: 1076-1080.
5. Ellington S, Salvatore P, Ko J, et al. Update: product, substance-use, and demographic characteristics of hospitalized patients in a nationwide outbreak of e-cigarette, or vaping, product use–associated lung injury - United States, August 2019-January 2020. *MMWR Morb Mortal Wkly Rep* 2020; 69: 44-49.
6. Evans ME, Twentyman E, Click ES, et al. Update: interim guidance for health care professionals evaluating and caring for patients with suspected e-cigarette, or vaping, product

[5] Duplicated data, combined in evidence synthesis with Polosa et al. 2014

use-associated lung injury and for reducing the risk for rehospitalization and death following hospital discharge - United States, December 2019. *MMWR Morb Mortal Wkly Rep* 2020; 68: 1189-1194.

7. Gaub K, Hallyburton S, Samanic C, et al. Patient charactertistics and product use behaviors among persons with e-cigarette, or vaping, product use-associated lung injury-Indiana, June-October 2019. *MMWR Morb Mortal Wkly Rep* 2019; 68: 1139-1141.
8. Jatlaoui T, Wiltz J, Kabbani S, et al. Update: interim guidance for health care providers for managing patients with suspected e-cigarette, or vaping, product use-associated lung injury - United States, November 2019. *MMWR Morb Mortal Wkly Rep* 2019; 68: 1081-1086.
9. Krishnasamy VP, Hallowell BD, Ko JY, et al. Update: characteristics of a nationwide outbreak of e-cigarette, or vaping, product use-associated lung injury - United States, August 2019-January 2020. *MMWR Morb Mortal Wkly Rep* 2020; 69: 90-94.
10. Lewis N, McCaffrey K, Sage K, et al. E-cigarette use, or vaping, practices and characteristics among persons with associated lung injury - Utah, April-October 2019. *MMWR Morb Mortal Wkly Rep* 2019; 68: 953-956.
11. Lozier MJ, Wallace B, Anderson K, et al. Update: demographic, product, and substance-use characteristics of hospitalized patients in a nationwide outbreak of e-cigarette, or vaping, product use-associated lung injuries - United States, December 2019. *MMWR Morb Mortal Wkly Rep* 2019; 68: 1142-1148.
12. Mikosz CA, Danielson M, Anderson KN, et al. Characteristics of patients experiencing rehospitalization or death after hospital discharge in a nationwide outbreak of e-cigarette, or vaping, product use-associated lung injury - United States, 2019. *MMWR Morb Mortal Wkly Rep* 2020; 68: 1183-1188.
13. Moritz ED, Zapata LB, Lekiachvili A, et al. Update: characteristics of patients in a national outbreak of e-cigarette, or vaping, product use-associated lung injuries - United States, October 2019. *MMWR Morb Mortal Wkly Rep* 2019; 68: 985-989.
14. Perrine C, Pickens C, Boehmer T, et al. Characteristics of a multistate outbreak of lung injury associated with e-cigarette use, or vaping - United States, 2019. *MMWR Morb Mortal Wkly Rep* 2019; 68: 860-864.
15. Schier JG, Meiman JG, Layden J, et al. Severe pulmonary disease associated with electronic-cigarette-product use - interim guidance. *MMWR Morb Mortal Wkly Rep* 2019; 68: 787-790.
16. Siegel DA, Jatlaoui TC, Koumans EH, et al. Update: interim guidance for health care providers evaluating and caring for patients with suspected e-cigarette, or vaping, product use associated lung injury - United States, October 2019. *MMWR Morb Mortal Wkly Rep* 2019; 68: 919-927.
17. Taylor J, Wiens T, Peterson J, et al. Characteristics of e-cigarette, or vaping, products used by patients with associated lung injury and products seized by law enforcement - Minnesota, 2018 and 2019. *MMWR Morb Mortal Wkly Rep* 2019; 68: 1096-1100.
18. Werner AK, Koumans EH, Chatham-Stephens K, et al. Hospitalizations and deaths associated with EVALI. *N Engl J Med* 2020; 382: 1589-1598.

Cross-sectional surveys

Not appropriate study design, not included in evidence synthesis

Case series (7)

1. Ansari-Gilani K, Petraszko AM, Teba CV, et al. E-cigarette use related lung disease, review of clinical and imaging finding in 3 cases. *Heart Lung* 2020; 49: 139-143.
2. Corcoran A, Carl JC, Rezaee F. The importance of anti-vaping vigilance-EVALI in seven adolescent pediatric patients in Northeast Ohio. *Pediatr Pulmonol* 2020; 55: 1719-1724.
3. Fryman C, Lou B, Weber AG, et al. Acute respiratory failure associated with vaping. *Chest* 2020; 157: e63-e68.

4. Isakov KMM, Legasto AC, Hossain R, et al. A case-based review of vaping-induced injury-pulmonary toxicity and beyond. *Curr Probl Diagn Radiol* 2020; 50: 401-409.
5. Kass AP, Overbeek DL, Chiel LE, et al. Case series: adolescent victims of the vaping public health crisis with pulmonary complications. *Pediatr Pulmonol* 2020; 55: 1224-1236.
6. Temas D, Meyer A. E-cigarette- and vaping-related lung injury (EVALI) at a regional hospital system in South Carolina. *Case Rep Pulmonol* 2020; 2020: 5370606.
7. Thakrar PD, Boyd KP, Swanson CP, et al. E-cigarette, or vaping, product use-associated lung injury in adolescents: a review of imaging features. *Pediatr Radiol* 2020; 50: 338-344.

Case reports (9)

1. Aftab G, Ahmad M, Frenia D. Vaping-associated lung injury. *Cureus* 2019; 11: e6216.
2. Casanova GS, Amaro R, Soler N, et al. An imported case of e-cigarette or vaping associated lung injury in Barcelona. *Eur Respir J* 2020; 55: 1902076.
3. Edmonds PJ, Copeland C, Conger A, et al. Vaping-induced diffuse alveolar hemorrhage. *Respir Med Case Rep* 2020; 29: 100996.
4. Farooq U, Anwar M, Alcantar D, et al. Gastroenteritis and miliary lung opacities: an interesting combination of findings. *Cureus* 2020; 12: e8848.
5. Patterson CM, Valchanov K, Barker A, et al. Severe acute respiratory distress syndrome requiring extracorporeal membrane oxygenation support: a consequence of vaping. *ERJ Open Res* 2020; 6: 00013-2020.
6. Sakla NM, Gattu R, Singh G, et al. Vaping-associated acute respiratory distress syndrome. *Emerg Radiol* 2020; 27: 103-106.
7. Sommerfeld CG, Weiner DJ, Nowalk A, et al. Hypersensitivity pneumonitis and acute respiratory distress syndrome from e-cigarette use. *Pediatrics* 2018; 141: e20163927.
8. Thota D, Latham E. Case report of electronic cigarettes possibly associated with eosinophilic pneumonitis in a previously healthy active-duty sailor. *J Emerg Med* 2014; 47: 15-17.
9. Venkatnarayan K, Rajamuri NKR, Krishnaswamy UM, et al. E-cigarettes: out of the frying pan into the fire? *Lung India* 2020; 37: 329-332.

Oral Health (4 studies)

Meta-analyses
No studies identified

Randomised controlled trials
No studies identified

Cohort studies (2)

1. Atuegwu NC, Perez MF, Oncken C, et al. Association between regular electronic nicotine product use and self-reported periodontal disease status: Population Assessment of Tobacco and Health Survey. *Int J Environ Res Public Health* 2019; 16: 1263.
2. Tatullo M, Gentile S, Paduano F, et al. Crosstalk between oral and general health status in e-smokers. *Medicine (Baltimore)* 2016; 95: e5589.

Non-randomised intervention studies (2)

1. Reuther WJ, Hale B, Matharu J, et al. Do you mind if I vape? Immediate effects of electronic cigarettes on perfusion in buccal mucosal tissue-a pilot study. *Br J Oral & Maxillofac Surg* 2016; 54: 338-341
2. Wadia R, Booth V, Yap HF, et al. A pilot study of the gingival response when smokers switch from smoking to vaping. *Br Dent J* 2016; 221: 722-726.

Case-control studies
No studies identified

Surveillance reports
No studies identified

Cross-sectional surveys
Not appropriate study design, not included in evidence synthesis

Case series
No studies identified

Case reports
Not appropriate study design, not included in evidence synthesis

Developmental and reproductive (3 studies)

Meta-analyses
No studies identified

Randomised controlled trials
No studies identified

Cohort studies (2)

1. Cardenas VM, Cen RQ, Clemens MM, et al. Use of electronic nicotine delivery systems (ENDS) by pregnant women I: risk of small-for-gestational-age birth. *Tob Induc Dis* 2019; 17: 44.
2. McDonnell BP, Dicker P, Regan CL. Electronic cigarettes and obstetric outcomes: a prospective observational study. *BJOG* 2020; 127: 750-756.

Non-randomised intervention studies
No studies identified

Case-control studies
No studies identified

Surveillance reports
No studies identified

Cross-sectional surveys (1)

1. Wang X, Lee NL, Burstyn I. Smoking and use of electronic cigarettes (vaping) in relation to preterm birth and small-for-gestational-age in a 2016 U.S. national sample. *Prev Med* 2020; 134: 106041.

Case series
No studies identified

Case reports
No studies identified

Burns and injuries (67 studies)

Meta-analyses
No studies identified

Randomised controlled trials
No studies identified

Cohort studies
No studies identified

Non-randomised intervention studies
No studies identified

Case-control studies
No studies identified

Surveillance reports (7)

1. Corey CG, Chang JT, Rostron BL. Electronic nicotine delivery system (ENDS) battery-related burns presenting to US emergency departments, 2016. *Inj Epidemiol* 2018; 5: 4.
2. Dohnalek HM, Harley EH. Analysis of electronic cigarette-related injury presenting to U.S. emergency departments, 2008-2017. *J Emerg Med* 2019; 57: 399-404.
3. McFaull SR, Do MT, Champagne A, et al. Injuries and poisonings associated with e-cigarettes and vaping substances, electronic Canadian Hospitals Injury Reporting and Prevention Program, 2011-2019. *Health Promot Chronic Dis Prev Can* 2020; 40: 250-254.
4. Rossheim ME, Livingston MD, Soule EK, et al. Electronic cigarette explosion and burn injuries, US emergency departments 2015-2017. *Tob Control* 2019; 28: 472-474.
5. Rudy SF, Durmowicz EL. Electronic nicotine delivery systems: overheating, fires and explosions. *Tob Control* 2017; 26: 10-18.
6. Saxena S, Kong LX, Pecht MG. Exploding e-cigarettes: a battery safety issue. *IEEE Access* 2018; 6: 21442-21466.
7. Wang B, Liu ST, Rostron B, et al. Burn injuries related to e-cigarettes reported to poison control centers in the United States, 2010-2019. *Inj Epidemiol* 2020; 7: 36.

Cross-sectional surveys
No studies identified

Case series (26)

1. Arnaout A, Dewi F, Nguyen D. Re: burn injuries from exploding electronic cigarette batteries: an emerging public health hazard. *J Plast Reconstr Aesthet Surg* 2017; 70: 981-982.
2. Bauman ZM, Roman J, Singer M, et al. Canary in the coal mine-initial reports of thermal injury secondary to electronic cigarettes. *Burns* 2017; 43: e38-e42.
3. Boissiere F, Bekara F, Luca-Pozner V, et al. Thermal and chemical burns caused by e-cigarette battery explosions. *Ann Chir Plast Esthet* 2020; 65: 24-30.
4. Brownson EG, Thompson CM, Goldsberry S, et al. Explosion injuries from e-cigarettes. *N Engl J Med* 2016; 375: 1400-1402.
5. Claes KEY, Vyncke T, De Wolf E, et al. Enzymatic debridement as an effective treatment for combined flame and chemical burns caused by e-cigarettes. *Am J Emerg Med* 2020; 38: 1199-1202.
6. Colaianni CA, Tapias LF, Cauley R, et al. Injuries caused by explosion of electronic cigarette devices. *Eplasty* 2016; 16: ic9.
7. Gibson CJS, Eshraghi N, Kemalyan NA, et al. Electronic cigarette burns: a case series. *Trauma* 2019; 21: 103-106.

8. Harshman J, Vojvodic M, Rogers AD. Burns associated with e-cigarette batteries: a case series and literature review. *CJEM* 2018; 20: S20-S28.
9. Hassan S, Anwar M, Muthayya P, et al. Burn injuries from exploding electronic cigarette batteries: an emerging public health hazard. *J Plast Reconstr Aesthet Surg* 2016; 69: 1716-1718.
10. Herlin C, Bekara F, Bertheuil N, et al. Deep burns caused by electronic vaping devices explosion. *Burns* 2016; 42: 1875-1877.
11. Hickey S, Goverman J, Friedstat J, et al. Thermal injuries from exploding electronic cigarettes. *Burns* 2018; 44: 1294-1301.
12. Isakov KMM, Legasto AC, Hossain R, et al. A case-based review of vaping-induced injury-pulmonary toxicity and beyond. *Curr Probl Diagn Radiol* 2020; 50: 401-409.
13. Jiwani AZ, Williams JF, Rizzo JA, et al. Thermal injury patterns associated with electronic cigarettes. *Int J Burns Trauma* 2017; 7: 1-5.
14. Kite AC, Le BQ, Cumpston KL, et al. Blast injuries caused by vape devices: 2 case reports. *Ann Plast Surg* 2016; 77: 620-622.
15. Kumetz EA, Hurst ND, Cudnik RJ, et al. Electronic cigarette explosion injuries. *Am J Emerg Med* 2016; 34: 2252.
16. Maraqa T, Mohamed MAT, Salib M, et al. Too hot for your pocket! Burns from e-cigarette lithium battery explosions: a case series. *J Burn Care Res* 2018; 39: 1043-1047.
17. Nicoll K, Rose A, Khan M, et al. Thigh burns from exploding e-cigarette lithium ion batteries: first case series. *Burns* 2016; 42: e42-e46.
18. Paley GL, Echalier E, Eck TW, et al. Corneoscleral laceration and ocular burns caused by electronic cigarette explosions. *Cornea* 2016; 35: 1015-1018.
19. Patterson SB, Beckett AR, Lintner A, et al. A novel classification system for injuries after electronic cigarette explosions. *J Burn Care Res* 2017; 38: e95-e100.
20. Quiroga L, Asif M, Lagziel T, et al. E-cigarette battery explosions: review of the acute management of the burns and the impact on our population. *Cureus* 2019; 11: e5355.
21. Ramirez JI, Ridgway CA, Lee JG, et al. The unrecognized epidemic of electronic cigarette burns. *J Burn Care Res* 2017; 38: 220-224.
22. Serror K, Chaouat M, Legrand MM, et al. Burns caused by electronic vaping devices (e-cigarettes): a new classification proposal based on mechanisms. *Burns* 2018; 44: 544-548.
23. Sheckter C, Chattopadhyay A, Paro J, et al. Burns resulting from spontaneous combustion of electronic cigarettes: a case series. *Burns Trauma* 2016; 4: 35.
24. Smith SL, Smith C, Cheatham M, et al. Electronic cigarettes: a burn case series. *J Nurse Pract* 2017; 13: 693-699.
25. Toy J, Dong F, Lee C, et al. Alarming increase in electronic nicotine delivery systems-related burn injuries: a serious unregulated public health issue. *Am J Emerg Med* 2017; 35: 1781-1782.
26. Treitl D, Solomon R, Davare DL, et al. Full and partial thickness burns from spontaneous combustion of e-cigarette lithium-ion batteries with review of literature. *J Emerg Med* 2017; 53: 121-125.

Case reports (34)

1. Ackley E, Williams JTB, Kunrath C, et al. Too hot to handle? When vaporizers explode. *J Pediatr* 2018; 196: 320-320.e1.
2. Anderson H, Richie C, Bernard A. A surprisingly volatile smoking alternative: explosion and burns as risks of e-cigarette use. *J Burn Care Res* 2017; 38: e884.
3. Andresen NS, Lee DJ, Kowalski CE, et al. Fall with e-cigarette in mouth resulting in pharyngeal and esophageal burns. *JAMA Otolaryngol Head Neck Surg* 2018; 144: 385-386.

4. Archambeau BA, Young S, Lee C, et al. E-cigarette blast injury: complex facial fractures and pneumocephalus. *Western J Emerg Med* 2016; 17: 805-807.
5. Ashburn NP, Smith ZP, Bozeman WP. Thermal blast injuries from an exploding e-cigarette. *Vis J Emerg Med* 2019; 14: 9-10.
6. Ban C, Krishnan DG, Abdallah Y. Ballistic trauma from an exploding electronic cigarette: case report. *Oral Maxillofac Surg Cases* 2017; 3: 61-63.
7. Beining T, Thogmartin JR, Kurz W. Projectile wound to head from modified electronic cigarette explosion. *J Forensic Sci* 2020; 65: 1365-1367.
8. Benowitz NL. E-cigarette explosion in a patient room. Rockville (MD), USA: US Department of Health and Human Services, AHRQ Patient Safety Network, 2019. https://psnet.ahrq.gov/web-mm/e-cigarette-explosion-patient-room (viewed June 2021).
9. Bohr S, Almarzouqi F, Pallua N. Extensive burn injury caused by fundamental electronic cigarette design flaw. *Ann Burns Fire Disasters* 2016; 29: 231-233.
10. Brooks JK, Kleinman JW, Brooks JB, et al. Electronic cigarette explosion associated with extensive intraoral injuries. *Dent Traumatol* 2017; 33: 149-152.
11. Cant A, Collard B, Cunliffe D. Electronic cigarettes: necrotic ulcer. *Br Dent J* 2017; 222: 226.
12. Cason DE, Morgan DE, Pietryga JA. Injuries from an exploding e-cigarette: a case report. *Ann Intern Med* 2016; 165: 678-679.
13. Chi AC, Neville BW, Ravenel M. Electronic cigarette explosion: case report of an emerging cause of orofacial trauma. *Trauma* 2018; 20: 62-67.
14. Foran I, Oak NR, Meunier MJ. High-pressure injection injury caused by electronic cigarette explosion: a case report. *JBJS Case Connect* 2017; 7: e36.
15. Goverman J, Schulz JT. Thigh burns from exploding e-cigarette. *Burns* 2016; 42: 1618.
16. Hagarty S, Luo J. E-cigarette "vape" device explosion causing C spine fracture. *Plast Reconstr Surg Glob Open* 2020; 8: e2745.
17. Harrison R, Hicklin D. Electronic cigarette explosions involving the oral cavity. *J Am Dent Assoc* 2016; 147: 891-896.
18. Jablow LM, Sexton RJ. Spontaneous electronic cigarette explosion: a case report. *Am J Med Case Rep* 2015; 3: 93-94.
19. Jamison A, Lockington D. Ocular chemical injury secondary to electronic cigarette liquid misuse. *JAMA Ophthalmol* 2016; 134: 1443.
20. Jordan KS, McCague Y. Ocular chemical burns secondary to accidental administration of e-cigarette liquid. *Adv Emerg Nurs J* 2018; 40: 104-109.
21. Katz MG, Russell KW. Injury from e-cigarette explosion. *N Engl J Med* 2019; 380: 2460.
22. Khairudin MN, Mohd Zahidin AZ, Bastion M-LC. Front to back ocular injury from a vaping-related explosion. *BMJ Case Rep* 2016; 2016: bcr2016214964.
23. Michael R, Ebraheim N, Maier J, et al. Electronic cigarette burns: a case report and review of current literature. *Case Rep Orthop* 2019; 2019: 4231764.
24. Moore J, Mihalache G, Messahel A. " Exploding" electronic cigarette: a case report. *Br J Oral Maxillofac Surg* 2016; 54: 1056-1057.
25. Norii T, Plate A. Electronic cigarette explosion resulting in a C1 and C2 fracture: a case report. *J Emerg Med* 2017; 52: 86-88.
26. Rogér JM, Abayon M, Elad S, et al. Oral trauma and tooth avulsion following explosion of e-cigarette. *J Oral Maxillofac Surg* 2016; 74: 1181-1185.
27. Sangani R, Chalhoub A, Dela Cruz M. Caustic burn after spontaneous explosion of lithium ion battery. *Vis J Emerg Med* 2019; 16: 100615.
28. Satteson ES, Walker NJ, Tuohy CJ, et al. Extensive hand thermal and blast injury from electronic cigarette explosion: a case report. *Hand (NY)* 2018; 13: NP1-NP5.
29. Schroeder N, Beavers K, Yu A, et al. 2016: electronic cigarettes and thermal injury: a case report on the dangers of vaping. *Crit Care Med* 2016; 44: 579.

30. Sedaghat F, Morgan RH. Incidentally discovered thyroid nodule following vape aspiration. *Vis J Emerg Med* 2020; 20: 100741.
31. Serror K, Chaouat M, De Runz A, et al. Thigh deep burns caused by electronic vaping devices (e-cigarettes): a new mechanism. *Burns* 2017; 43: 1133-1135.
32. Shastry S, Langdorf MI. Electronic vapor cigarette battery explosion causing shotgun-like superficial wounds and contusion. *West J Emerg Med* 2016; 17: 177-180.
33. Vaught B, Spellman J, Shah A, et al. Facial trauma caused by electronic cigarette explosion. *Ear Nose Throat J* 2017; 96: 139-142.
34. Walsh K, Sheikh Z, Johal K, et al. Rare case of accidental fire and burns caused by e-cigarette batteries. *BMJ Case Rep* 2016; 2016: bcr2015212868.

Poisonings (59 studies)

Meta-analyses
No studies identified

Randomised controlled trials
No studies identified

Cohort studies

No studies identified

Non-randomised intervention studies
No studies identified

Case-control studies
No studies identified

Surveillance reports (28)

1. Ang E, Tuthill D, Thompson J. E-cigarette liquid ingestion: a fast growing accidental issue in children. *Arch Dis Child* 2018; 103: 1091.
2. Anonymous. Electronic cigarettes: poisoning in children. *Prescrire* 2015; 24: 21.
3. Cantrell FL, Clark RF. More on nicotine poisoning in infants. *N Engl J Med* 2014; 371: 880.
4. Cantrell FL. Adverse effects of e-cigarette exposures. *J Community Health* 2014; 39: 614-616.
5. Chang JT, Rostron BL. Electronic nicotine delivery system (ENDS) liquid nicotine exposure in young children presenting to US emergency departments, 2018. *Inj Epidemiol* 2019; 6: 43.
6. Chang JT, Wang B, Chang CM, et al. National estimates of poisoning events related to liquid nicotine in young children treated in US hospital emergency departments, 2013-2017. *Inj Epidemiol* 2019; 6: 10.
7. Chang JT, Wang B, Rostron BL, et al. National estimates of ENDS liquid nicotine exposures, U.S., 2013-2017. *Am J Prev Med* 2020; 59: 742-745.
8. Chatham-Stephens K, Law R, Taylor E, et al. Exposure calls to U.S. poison centers involving electronic cigarettes and conventional cigarettes-September 2010-December 2014. *J Med Toxicol* 2016; 12: 350-357.
9. Chatham-Stephens K, Law R, Taylor E, et al. Notes from the field: calls to poison centers for exposures to electronic cigarettes-United States, September 2010-February 2014. *MMWR Morb Mortal Wkly Rep* 2014; 63: 292-293.
10. Choi A, Le M, Rahim T, et al. Electronic cigarette exposures reported to the British Columbia Drug and Poison Information Centre: an observational case series. *CMAJ Open* 2019; 7: E462-e471.
11. De La Oliva U, Conejo Menor J. Exposures to electronic cigarettes: calls to the poison center in Spain. *Rev Espanola de Medicina Leg* 2014; 40: 146-149.
12. Forrester MB. Pediatric exposures to electronic cigarettes reported to Texas poison centers. *J Emerg Med* 2015; 49: 136-142.
13. Govindarajan P, Spiller HA, Casavant MJ, et al. E-Cigarette and liquid nicotine exposures among young children. *Pediatrics* 2018; 141: e20173361.
14. Hughes A, Hendrickson RG. An epidemiologic and clinical description of e-cigarette toxicity. *Clin Toxicol (Phila)* 2019; 57: 287-293.
15. Kamboj A, Spiller HA, Casavant MJ, et al. Pediatric exposure to e-cigarettes, nicotine, and tobacco products in the United States. *Pediatrics* 2016; 137: e20160041.
16. LoVecchio F, Zoph O. Incidence of electronic cigarette exposures in children skyrockets in Arizona. *Am J Emerg Med* 2015; 33: 834-835.

17. McFaull SR, Do MT, Champagne A, et al. Injuries and poisonings associated with e-cigarettes and vaping substances, electronic Canadian Hospitals Injury Reporting and Prevention Program, 2011-2019. *Health Promot Chronic Dis Prevent Can* 2020; 40: 250-254.
18. Mowry JB, Spyker DA, Brooks DE, et al. 2014 annual report of the American Association of Poison Control Centers' National Poison Data System (NPDS): 32nd annual report. *Clin Toxicol (Phila)* 2015; 53: 962-1147.
19. Mowry JB, Spyker DA, Cantilena LR, Jr., et al. 2013 annual report of the American Association of Poison Control Centers' National Poison Data System (NPDS): 31st annual report. *Clin Toxicol (Phila)* 2014; 52: 1032-1283.
20. Obertova N, Navratil T, Zak I, et al. Acute exposures to e-cigarettes and heat-not-burn products reported to the Czech Toxicological Information Centre over a 7-year period (2012-2018). *Basic Clin Pharmacol Toxicol* 2020; 127: 39-46.
21. Ordonez JE, Kleinschmidt KC, Forrester MB. Electronic cigarette exposures reported to Texas poison centers. *Nicotine Tob Res* 2015; 17: 209-211.
22. Thomas E, Spears RA, Alldridge G, et al. E-cigarette liquid refills-a safe beverage? Analysis of enquiries to the UK National Poisons Information Service from 2007 to 2013. Brussels, Belgium: XXXIV International Congress of the European Association of Poisons Centres and Clinical Toxicologists (EAPCCT), 2014. https://www.tandfonline.com/doi/full/10.3109/15563650.2014.906213 (viewed June 2021).
23. Vakkalanka JP, Hardison LS, Holstege CP. Epidemiological trends in electronic cigarette exposures reported to U.S. poison centers. *Clin Toxicol (Phila)* 2014; 52: 542-548.
24. Valentine N, McClelland E, Bryant J, et al. Electronic cigarettes in Mississippi: issues facing healthcare providers and policy makers. *J Miss State Med Assoc* 2016; 57: 181-189.
25. Vardavas CI, Girvalaki C, Filippidis FT, et al. Characteristics and outcomes of e-cigarette exposure incidents reported to 10 European poison centers: a retrospective data analysis. *Tob Induc Dis* 2017; 15: 36.
26. Wang BG, Liu S, Persoskie A. Poisoning exposure cases involving e-cigarettes and e-liquid in the United States, 2010-2018. *Clin Toxicol (Phila)* 2020; 58: 488-494.
27. Weiss D, Tomasallo CD, Meiman JG, et al. Electronic cigarette exposure: calls to Wisconsin poison control centers, 2010-2015. *WMJ* 2016; 115: 306-310.
28.
29. Wylie C, Heffernan A, Brown JA, et al. Exposures to e-cigarettes and their refills: calls to Australian Poisons Information Centres, 2009-2016. *Med J Aust* 2019; 210: 126.

Cross-sectional surveys
No studies identified

Case series (4)
1. Christensen LB, van't Veen T, Bang J. Three cases of attempted suicide by ingestion of nicotine liquid used in e-cigarettes. *Clin Toxicol (Phila)* 2013; 51: 290.
2. Isakov KMM, Legasto AC, Hossain R, et al. A case-based review of vaping-induced injury-pulmonary toxicity and beyond. *Curr Probl Diagn Radiol* 2020; 50: 401-409.
3. Park EJ, Min YG. The emerging method of suicide by electronic cigarette liquid: a case report. *J Korean Med Sci* 2018; 33: e52.
4. Sommerfeld K, Łukasik-Głębocka M, Kulza M, et al. Intravenous and oral suicidal e-liquid poisonings with confirmed nicotine and cotinine concentrations. *Forensic Sci Int* 2016; 262: e15-e20.

Case reports (27)
1. Aoki Y, Ikeda T, Tani N, et al. Evaluation of the distribution of nicotine intravenous injection: an adult autopsy case report with a review of literature. *Int J Legal Med* 2020; 134: 243-249.

2. Bartschat S, Mercer-Chalmers-Bender K, Beike J, et al. Not only smoking is deadly: fatal ingestion of e-juice-a case report. *Int J Legal Med* 2015; 129: 481-486.
3. Bassett RA, Osterhoudt K, Brabazon T. Nicotine poisoning in an infant. *N Engl J Med* 2014; 370: 2249-2250.
4. Belkoniene M, Socquet J, Njemba-Freiburghaus D, et al. Near fatal intoxication by nicotine and propylene glycol injection: a case report of an e-liquid poisoning. *BMC Pharmacol Toxicol* 2019; 20: 28.
5. Cervellin G, Luci M, Bellini C, et al. Bad news about an old poison. A case of nicotine poisoning due to both ingestion and injection of the content of an electronic cigarette refill. *Emerg Care J* 2013; 9: 53-54.
6. Chen BC, Bright SB, Trivedi AR, et al. Death following intentional ingestion of e-liquid. *Clin Toxicol (Phila)* 2015; 53: 914-916.
7. De Pieri C, Brisotto S, Marzona F, et al. Liquid nicotine intoxication due to dangerous packaging. *Pediatr Emerg Care* 2020; 36: e425.
8. Demir E, Topal S. Sudden sensorineural hearing loss associated with electronic cigarette liquid: the first case in the literature. *Int J Pediatr Otorhinolaryngol* 2018; 114: 26-28.
9. Eberlein CK, Frieling H, Köhnlein T, et al. Suicide attempt by poisoning using nicotine liquid for use in electronic cigarettes. *Am J Psychiatry* 2014; 171: 891.
10. Eggleston W, Nacca N, Stork CM, et al. Pediatric death after unintentional exposure to liquid nicotine for an electronic cigarette. *Clin Toxicol (Phila)* 2016; 54: 890-891.
11. Garat A, Nisse P, Kauv M, et al. Lactic acidosis due to voluntary e-liquid ingestion. *Toxicol Anal Clin* 2016; 28: 329-332.
12. Gill N, Sangha G, Poonai N, et al. E-cigarette liquid nicotine ingestion in a child: case report and discussion. *CJEM* 2015; 17: 699-703.
13. Gupta S, Gandhi A, Manikonda R. Accidental nicotine liquid ingestion: emerging paediatric problem. *Arch Dis Child* 2014; 99: 1149.
14. Howard C. A new source for nicotine exposures in pediatric patients: electronic cigarettes. *J Emerg Nurs* 2016; 42: 451-453.
15. Jamison A, Lockington D. Ocular chemical injury secondary to electronic cigarette liquid misuse. *JAMA Ophthalmol* 2016; 134: 1443.
16. Lam CN, Goldenson NI, Burner E, et al. Cultural buffering as a protective factor against electronic cigarette use among Hispanic emergency department patients. *Addict Behav* 2016; 63: 155-160.
17. Lee J, You Y, Park JS, et al. Liver donation after brain death following intentional ingestion of 99% e-cigarette liquid nicotine 10 mL. *Exp Clin Transplant* 2020; 18: 120-122.
18. Morley S, Slaughter J, Smith PR. Death from ingestion of e-liquid. *J Emerg Med* 2017; 53: 862-864.
19. Noble MJ, Longstreet B, Hendrickson RG, et al. Unintentional pediatric ingestion of electronic cigarette nicotine refill liquid necessitating intubation. *Ann Emerg Med* 2017; 69: 94-97.
20. Paik JH, Kang S, Durey A, et al. Symptomatic bradycardia due to nicotine intoxication. *Rev Bras Ter Intensiva* 2018; 30: 121-126.
21. Räsänen M, Helanterä I, Kalliomäki J, et al. A case report of successful kidney donation after brain death following nicotine intoxication. *Transplant Proc* 2017; 49: 229-231.
22. Scarpino M, Bonizzoli M, Lanzi C, et al. Brain death following ingestion of e-cigarette liquid nicotine refill solution. *Brain Behav* 2020; 10: e01744.
23. Schipper EM, de Graaff LCG, Koch BCP, et al. A new challenge: suicide attempt using nicotine fillings for electronic cigarettes. *Br J Clin Pharmacol* 2014; 78: 1469-1471.
24. Seo AD, Kim DC, Yu HJ, et al. Accidental ingestion of e-cigarette liquid nicotine in a 15-month-old child: an infant mortality case of nicotine intoxication. *Korean J Pediatr* 2016; 59: 490-493.

25. Thornton SL, Oller L, Sawyer T. Fatal intravenous injection of electronic nicotine delivery system refilling solution. *J Med Toxicol* 2014; 10: 202-204.
26. van der Meer DH, Pranger AD, Jansen I, et al. Fatal intoxication with nicotine for e-cigarette. *Ned Tijdschr Geneeskd* 2017; 161: D1591.
27. You G, Rhee J, Park Y, et al. Determination of nicotine, cotinine and trans-3′-hydroxycotinine using LC/MS/MS in forensic samples of a nicotine fatal case by oral ingestion of e-cigarette liquid. *J Forensic Sci* 2016; 61: 1149-1154.

Mental Health (3 studies)

Meta-analyses
No studies identified

Randomised controlled trials
No studies identified

Cohort studies
1. Bandiera FC, Loukas A, Li X, et al. Depressive symptoms predict current e-cigarette use among college students in Texas. *Nicotine Tob Res* 2017; 19: 1102-1106.
2. Lechner WV, Janssen T, Kahler CW, et al. Bi-directional associations of electronic and combustible cigarette use onset patterns with depressive symptoms in adolescents. *Prev Med* 2017; 96: 73-78.
3. Marsden DG, Loukas A, Chen BJ, et al. Associations between frequency of cigarette and alternative tobacco product use and depressive symptoms: a longitudinal study of young adults. *Addict Behav* 2019; 99: 106078.

Non-randomised intervention studies
No studies identified

Case-control studies
No studies identified

Surveillance reports
No studies Identified

Cross-sectional surveys
Not appropriate evidence, not included in evidence synthesis

Case series
No studies identified

Case reports
No studies identified

Environmental hazards with health implications (25 studies)

Meta-analyses
No studies identified

Randomised controlled trials
No studies identified

Cohort studies
No studies identified

Non-randomised intervention study/controlled experimental studies (17)

1. Ballbè M, Martínez-Sánchez JM, Sureda X, et al. Cigarettes vs. e-cigarettes: passive exposure at home measured by means of airborne marker and biomarkers. *Environ Res* 2014; 135: 76-80.
2. Chen R, Aherrera A, Isichei C, et al. Assessment of indoor air quality at an electronic cigarette (vaping) convention. *J Expo Sci Environ Epidemiol* 2018; 28: 522-529.
3. Coppeta L, Magrini A, Pietroiusti A, et al. Effects of smoking electronic cigarettes on pulmonary function and environmental parameters. *Open Public Health J* 2018; 11: 360-368.
4. Czogała J, Goniewicz ML, Fidelus B, et al. Secondhand exposure to vapors from electronic cigarettes. *Nicotine Tob Res* 2014; 16: 655-662.
5. Fernández E, Ballbè M, Sureda X, et al. Particulate matter from electronic cigarettes and conventional cigarettes: a systematic review and observational study. *Curr Environ Health Rep* 2015; 2: 423-429.
6. Liu JM, Liang QW, Oldham MJ, et al. Determination of selected chemical levels in room air and on surfaces after the use of cartridge- and tank-based e-vapor products or conventional cigarettes. *Int J Environ Res Public Health* 2017; 14: 969.
7. Loupa G, Karali D, Rapsomanikis S. The trace of airborne particulate matter from smoking e-cigarette, tobacco heating system, conventional and hand-rolled cigarettes in a residential environment. *Air Qual Atmos Health* 2019; 12: 1449-1457.
8. Melstrom P, Koszowski B, Thanner MH, et al. Measuring PM2.5, ultrafine particles, nicotine air and wipe samples following the use of electronic cigarettes. *Nicotine Tob Res* 2017; 19: 1055-1061.
9. Protano C, Avino P, Manigrasso M, et al. Environmental electronic vape exposure from four different generations of electronic cigarettes: airborne particulate matter levels. *Int J Environ Res Public Health* 2018; 15: 2172.
10. Protano C, Manigrasso M, Avino P, et al. Second-hand smoke generated by combustion and electronic smoking devices used in real scenarios: ultrafine particle pollution and age-related dose assessment. *Environ Int* 2017; 107: 190-195.
11. Protano C, Manigrasso M, Cammalleri V, et al. Impact of electronic alternatives to tobacco cigarettes on indoor air particular matter levels. *Int J Environ Res Public Health* 2020; 17: 2947.
12. Savdie J, Canha N, Buitrago N, et al. Passive exposure to pollutants from a new generation of cigarettes in real life scenarios. *Int J Environ Res Public Health* 2020; 17: 3455.
13. Schober W, Fembacher L, Frenzen A, et al. Passive exposure to pollutants from conventional cigarettes and new electronic smoking devices (IQOS, e-cigarette) in passenger cars. *Int J Hyg Environ Health* 2019; 222: 486-493.
14. Schober W, Szendrei K, Matzen W, et al. Use of electronic cigarettes (e-cigarettes) impairs indoor air quality and increases FeNO levels of e-cigarette consumers. *Int J Hyg Environ Health* 2014; 217: 628-637.
15. Soule EK, Maloney SF, Spindle TR, et al. Electronic cigarette use and indoor air quality in a natural setting. *Tob Control* 2017; 26: 109-112.

16. van Drooge BL, Marco E, Perez N, et al. Influence of electronic cigarette vaping on the composition of indoor organic pollutants, particles, and exhaled breath of bystanders. *Environ Sci Pollut Res* 2019; 26: 4654-4666.
17. Volesky KD, Maki A, Scherf C, et al. The influence of three e-cigarette models on indoor fine and ultrafine particulate matter concentrations under real-world conditions. *Environ Pollut* 2018; 243: 882-889.

Case-control studies

No studies identified

Surveillance reports (3)

1. McKenna LA, Jr. Electronic cigarette fires and explosions in the United States 2009-2016. National Fire Data Center, US Fire Administration, 2017. https://permanent.fdlp.gov/gpo121499/electronic_cigarettes.pdf (viewed June 2021).
2. Saxena S, Kong LX, Pecht MG. Exploding e-cigarettes: a battery safety issue. *IEEE Access* 2018; 6: 21442-21466.
3. UK Government. Fire statistics data tables. Updated 19 January 2023. https://www.gov.uk/government/statistical-data-sets/fire-statistics-data-tables (viewed June 2021).

Cross-sectional surveys

No studies identified

Case series/natural experimental studies (5)

1. Cammalleri V, Marotta D, Protano C, et al. How do combustion and non-combustion products used outdoors affect outdoor and indoor particulate matter levels? A field evaluation near the entrance of an italian university library. *Int J Environ Res Public Health* 2020; 17: 5200.
2. Khachatoorian C, Jacob P, Benowitz NL, et al. Electronic cigarette chemicals transfer from a vape shop to a nearby business in a multiple-tenant retail building. *Tob Control* 2019; 28: 519-525.
3. Khachatoorian C, Jacob P, Sen A, et al. Identification and quantification of electronic cigarette exhaled aerosol residue chemicals in field sites. *Environ Res* 2019; 170: 351-358.
4. Mock J, Hendlin YH. Notes from the field: environmental contamination from e-cigarette, cigarette, cigar, and cannabis products at 12 high schools-San Francisco Bay area, 2018-2019. *MMWR Morb Mortal Wkly Rep* 2019; 68: 897-899.
5. Nguyen C, Li LQ, Sen CA, et al. Fine and ultrafine particles concentrations in vape shops. *Atmos Environ* 2019; 211: 159-169.

Case reports

No studies identified

Neurological outcomes (13 studies)

Meta-analyses
No studies identified

Randomised controlled trials
No studies identified

Cohort studies
No studies identified

Non-randomised intervention studies
No studies identified

Case-control studies
No studies identified

Surveillance reports (3)
1. Faulcon LM, Rudy S, Limpert J, et al. Adverse experience reports of seizures in youth and young adult electronic nicotine delivery systems users. *J Adolesc Health* 2020; 66: 15-17.
2. Govindarajan P, Spiller HA, Casavant MJ, et al. E-cigarette and liquid nicotine exposures among young children. *Pediatrics* 2018; 141: e20173361.
3. Obertova N, Navratil T, Zak I, et al. Acute exposures to e-cigarettes and heat-not-burn products reported to the Czech Toxicological Information Centre over a 7-year period (2012-2018). *Basic Clin Pharmacol Toxicol* 2020; 127: 39-46.

Cross-sectional surveys
No studies identified

Case series (2)
1. Liu EMN, McIntosh A. First seizure in adolescent immediately following e-cigarette use: two patient cases. *Neurol Clin Neurosci* 2020; 8: 158-159.
2. Park EJ, Min YG. The emerging method of suicide by electronic cigarette liquid: a case report. *J Korean Med Sci* 2018; 33: e52.

Case reports (8)
1. Ackley E, Williams JTB, Kunrath C, et al. Too hot to handle? When vaporizers explode. *J Pediatr* 2018; 196: 320-320.e1.
2. Belkoniene M, Socquet J, Njemba-Freiburghaus D, et al. Near fatal intoxication by nicotine and propylene glycol injection: a case report of an e-liquid poisoning. *BMC Pharmacol Toxicol* 2019; 20: 28.
3. Demir E, Topal S. Sudden sensorineural hearing loss associated with electronic cigarette liquid: the first case in the literature. *Int J Pediatr Otorhinolaryngol* 2018; 114: 26-28.
4. Hughes A, Hendrickson RG. An epidemiologic and clinical description of e-cigarette toxicity. *Clin Toxicol (Phila)* 2019; 57: 287-293.
5. Noble MJ, Longstreet B, Hendrickson RG, et al. Unintentional pediatric ingestion of electronic cigarette nicotine refill liquid necessitating intubation. *Ann Emerg Med* 2017; 69: 94-97.
6. Satteson ES, Walker NJ, Tuohy CJ, et al. Extensive hand thermal and blast injury from electronic cigarette explosion: a case report. *Hand (NY)* 2018; 13: NP1-NP5.
7. Vannier S, Ronziere T, Ferre J, et al. Reversible cerebral vasoconstriction syndrome triggered by an electronic cigarette: case report. *Eur J Neurol* 2015; 22: e64-e65.
8. Wharton JD, Kozek LK, Carson RP. Increased seizure frequency temporally related to vaping: where there's vapor, there's seizures? *Pediatr Neurol* 2020; 104: 66-67.

Sleep (0 studies)

Meta-analyses
No studies identified

Randomised controlled trials
No studies identified

Cohort studies
No studies identified

Non-randomised intervention studies
No studies identified

Case-control studies
No studies identified

Surveillance reports
No studies identified

Cross-sectional surveys
Not appropriate evidence, not included in evidence synthesis

Case series
No studies identified

Case reports
No studies identified

Less serious adverse events (47 studies)

Meta-analyses (1)

1. Hartmann-Boyce J, McRobbie H, Lindson N, et al. Electronic cigarettes for smoking cessation. *Cochrane Database Syst Rev* 2021; 4: CD010216.

Randomised controlled trials (24)

1. Adriaens K, Van Gucht D, Declerck P, et al. Effectiveness of the electronic cigarette: an eight-week Flemish study with six-month follow-up on smoking reduction, craving and experienced benefits and complaints. *Int J Environ Res Public Health* 2014; 11: 11220-11248.
2. Baldassarri SR, Bernstein SL, Chupp GL, et al. Electronic cigarettes for adults with tobacco dependence enrolled in a tobacco treatment program: a pilot study. *Addict Behav* 2018; 80: 1-5.
3. Bonevski B, Manning V, Wynne O, et al. QuitNic: a pilot randomized controlled trial comparing nicotine vaping products with nicotine replacement therapy for smoking cessation following residential detoxification. *Nicotine Tob Res* 2021; 23: 462-470.
4. Boulay M-È, Henry C, Bossé Y, et al. Acute effects of nicotine-free and flavour-free electronic cigarette use on lung functions in healthy and asthmatic individuals. *Respir Res* 2017; 18: 33.
5. Bullen C, Howe C, Laugesen M, et al. Electronic cigarettes for smoking cessation: a randomised controlled trial. *Lancet* 2013; 382: 1629-1637.
6. Campagna D, Cibella F, Caponnetto P, et al. Changes in breathomics from a 1-year randomized smoking cessation trial of electronic cigarettes. *Eur J Clin Invest* 2016; 46: 698-706.[6]
7. Caponnetto P, Campagna D, Cibella F, et al. EffiCiency and safety of an eLectronic cigAreTte (ECLAT) as tobacco cigarettes substitute: a prospective 12-month randomized control design study. *PLoS One* 2013; 8: e66317.
8. Carpenter MJ, Heckman BW, Wahlquist AE, et al. A naturalistic, randomized pilot trial of e-cigarettes: uptake, exposure, and behavioral effects. *Cancer Epidemiol Biomarkers Prev* 2017; 26: 1795-1803.
9. Cibella F, Campagna D, Caponnetto P, et al. Lung function and respiratory symptoms in a randomized smoking cessation trial of electronic cigarettes. *Clin Sci (Lond)* 2016; 130: 1929-1937.[7]
10. Cravo A, Bush J, Sharma G, et al. A randomised, parallel group study to evaluate the safety profile of an electronic vapour product over 12 weeks. *Regul Toxicol Pharmacol* 2016; 81 Suppl 1: S1-S14.
11. Dawkins L, Bauld L, Ford A, et al. A cluster feasibility trial to explore the uptake and use of e-cigarettes versus usual care offered to smokers attending homeless centres in Great Britain. *PLoS One* 2020; 15: e0240968.
12. Eisenberg MJ, Hébert-Losier A, Windle SB, et al. Effect of e-cigarettes plus counseling vs counseling alone on smoking cessation: a randomized clinical trial. *JAMA* 2020; 324: 1844-1854.
13. Felicione NJ, Enlow P, Elswick D, et al. A pilot investigation of the effect of electronic cigarettes on smoking behavior among opioid-dependent smokers. *Addict Behav* 2019; 91: 45-50.
14. George J, Hussain M, Vadiveloo T, et al. Cardiovascular effects of switching from tobacco cigarettes to electronic cigarettes. *J Am Coll Cardiol* 2019; 74: 3112-3120.
15. Hajek P, Phillips-Waller A, Przulj D, et al. A randomized trial of e-cigarettes versus nicotine-replacement therapy. *N Engl J Med* 2019; 380: 629-637.

[6] Duplicated data, combined in evidence synthesis with Cibella et al. 2016

[7] Duplicated data, combined in evidence synthesis with Campangna et al. 2016

16. Holliday R, Preshaw PM, Ryan V, et al. A feasibility study with embedded pilot randomised controlled trial and process evaluation of electronic cigarettes for smoking cessation in patients with periodontitis. *Pilot Feasibility Stud* 2019; 5: 74.
17. Lee S-H, Ahn S-H, Cheong Y-S. Effect of electronic cigarettes on smoking reduction and cessation in Korean male smokers: a randomized controlled study. *J Am Board Fam Med* 2019; 32: 567-574.
18. Lee SM, Tenney R, Wallace AW, et al. E-cigarettes versus nicotine patches for perioperative smoking cessation: a pilot randomized trial. *PeerJ* 2018; 6: e5609.
19. Lucchiari C, Masiero M, Mazzocco K, et al. Benefits of e-cigarettes in smoking reduction and in pulmonary health among chronic smokers undergoing a lung cancer screening program at 6 months. *Addict Behav* 2020; 103: 106222.
20. Masiero M, Lucchiari C, Mazzocco K, et al. E-cigarettes may support smokers with high smoking-related risk awareness to stop smoking in the short run: preliminary results by randomized controlled trial. *Nicotine Tob Res* 2019; 21: 119-126.
21. Meier E, Wahlquist AE, Heckman BW, et al. A pilot randomized crossover trial of electronic cigarette sampling among smokers. *Nicotine Tob Res* 2017; 19: 176-182.
22. Myers Smith K, Phillips-Waller A, Pesola F, et al. E-cigarettes versus nicotine replacement treatment as harm reduction interventions for smokers who find quitting difficult: Randomised controlled trial. *Addiction* 2022; 117: 224-233.
23. Ozga-Hess JE, Felicione NJ, Ferguson SG, et al. Piloting a clinical laboratory method to evaluate the influence of potential modified risk tobacco products on smokers' quit-related motivation, choice, and behavior. *Addict Behav* 2019; 99: 106105.
24. Pulvers K, Nollen NL, Rice M, et al. Effect of pod e-cigarettes vs cigarettes on carcinogen exposure among African American and Latinx smokers: a randomized clinical trial. *JAMA Netw Open* 2020; 3: e2026324.
25. Tseng T-Y, Ostroff JS, Campo A, et al. A randomized trial comparing the effect of nicotine versus placebo electronic cigarettes on smoking reduction among young adult smokers. *Nicotine Tob Res* 2016; 18: 1937-1943.

Cohort studies (17)

1. Bell S, Dean J, Gilks C, et al. Tobacco harm reduction with vaporised nicotine (THRiVe): the study protocol of an uncontrolled feasibility study of novel nicotine replacement products among people living with HIV who smoke. *Int J Environ Res Public Health* 2017; 14: 799.
2. Caponnetto P, Maglia M, Cannella MC, et al. Impact of different e-cigarette generation and models on cognitive performances, craving and gesture: a randomized cross-over trial (CogEcig). *Front Psychol* 2017; 8: 127.
3. Goniewicz ML, Gawron M, Smith DM, et al. Exposure to nicotine and selected toxicants in cigarette smokers who switched to electronic cigarettes: a longitudinal within-subjects observational study. *Nicotine Tob Res* 2017; 19: 160-167.
4. Hajek P, Corbin L, Ladmore D, et al. Adding e-cigarettes to specialist stop-smoking treatment: City of London pilot project. *J Addict Res Ther* 2015; 6: 244.
5. Hickling LM, Perez-Iglesias R, McNeill A, et al. A pre-post pilot study of electronic cigarettes to reduce smoking in people with severe mental illness. *Psychol Med* 2019; 49: 1033-1040.
6. Humair JP, Tango R. Can e-cigarette help patients to reduce or stop smoking in primary care practice? *J Gen Intern Med* 2014; 29: S480.
7. Nides MA, Leischow SJ, Bhatter M, et al. Nicotine blood levels and short-term smoking reduction with an electronic nicotine delivery system. *Am J Health Behav* 2014; 38: 265-274.
8. Oncken CA, Litt MD, McLaughlin LD, et al. Nicotine concentrations with electronic cigarette use: effects of sex and flavor. *Nicotine Tob Res* 2015; 17: 473-478.
9. Polosa R, Caponnetto P, Maglia M, et al. Success rates with nicotine personal vaporizers: a prospective 6-month pilot study of smokers not intending to quit. *BMC Public Health* 2014a; 14: 1159.

10. Polosa R, Caponnetto P, Morjaria JB, et al. Effect of an electronic nicotine delivery device (e-cigarette) on smoking reduction and cessation: a prospective 6-month pilot study. *BMC Public Health* 2011; 11: 786.[8]
11. Polosa R, Cibella F, Caponnetto P, et al. Health impact of e-cigarettes: a prospective 3.5-year study of regular daily users who have never smoked. *Sci Rep* 2017; 7: 13825.
12. Polosa R, Morjaria JB, Caponnetto P, et al. Effectiveness and tolerability of electronic cigarette in real-life: a 24-month prospective observational study. *Intern Emerg Med* 2014b; 9: 537-546.
13. Pratt SI, Sargent J, Daniels L, et al. Appeal of electronic cigarettes in smokers with serious mental illness. *Addict Behav* 2016; 59: 30-34.
14. Stein MD, Caviness C, Grimone K, et al. An open trial of electronic cigarettes for smoking cessation among methadone-maintained smokers. *Nicotine Tob Res* 2016; 18: 1157-1162.
15. Valentine GW, Hefner K, Jatlow PI, et al. Impact of e-cigarettes on smoking and related outcomes in veteran smokers with psychiatric comorbidity. *J Dual Diagn* 2018; 14: 2-13.
16. Van Staden SR, Groenewald M, Engelbrecht R, et al. Carboxyhaemoglobin levels, health and lifestyle perceptions in smokers converting from tobacco cigarettes to electronic cigarettes. *S Afr Med J* 2013; 103: 865-868.
17. Wadia R, Booth V, Yap HF, et al. A pilot study of the gingival response when smokers switch from smoking to vaping. *Br Dent J* 2016; 221: 722-726.
18. Walele T, Bush J, Koch A, et al. Evaluation of the safety profile of an electronic vapour product used for two years by smokers in a real-life setting. *Regul Toxicol Pharmacol* 2018; 92: 226-238.

Non-randomised intervention studies (4)

1. Caponnetto P, Auditore R, Russo C, et al. Impact of an electronic cigarette on smoking reduction and cessation in schizophrenic smokers: a prospective 12-month pilot study. *Int J Environ Res Public Health* 2013; 10: 446-461.
2. Dicpinigaitis PV, Lee Chang A, Dicpinigaitis AJ, et al. Effect of e-cigarette use on cough reflex sensitivity. *Chest* 2016; 149: 161-165.
3. Dicpinigaitis PV, Lee Chang A, Dicpinigaitis AJ, et al. Effect of electronic cigarette use on the urge-to-cough sensation. *Nicotine Tob Res* 2016; 18: 1763-1765.
4. Palamidas A, Tsikrika S, Katsaounou P, et al. Acute effects of short term use of e-cigarettes on airways physiology and respiratory symptoms in smokers with and without airways obstructive diseases and in healthy non smokers. *Tob Prev Cessat* 2017; 3: 5.

Case-control study

No studies identified

Surveillance reports (1)

1. Motooka Y, Matsui T, Slaton RM, et al. Adverse events of smoking cessation treatments (nicotine replacement therapy and non-nicotine prescription medication) and electronic cigarettes in the Food and Drug Administration Adverse Event Reporting System, 2004-2016. *SAGE Open Med* 2018; 6: 2050312118777953.
2. MHRA Yellow Card[9]

Cross-sectional surveys

Not appropriate study design, not included in evidence synthesis

Case series

[8] Duplicated data, combined in evidence synthesis with Polosa et al. 2014b

[9] No reference provided in Public Health England 2018 report

No studies identified

Case reports

No studies identified

Optical Health (1 study)

Meta-analyses

No studies identified

Randomised controlled trials

No studies identified

Cohort studies

No studies identified

Non-randomised intervention studies (1)

1. Munsamy A, Bhanprakash B, Sirkhot A, et al. A pre-test post-test assessment of non-invasive keratograph break up time and corneal epithelial thickness after vaping. *Afr Health Sci* 2019; 19: 2926-2933.

Case-control studies

No studies identified

Surveillance reports

No studies identified

Cross-sectional surveys

Not appropriate evidence, not included in evidence synthesis

Case series

No studies identified

Case reports

No studies identified

Wound healing (0 studies)

Meta-analyses
No studies identified

Randomised controlled trials
No studies identified

Cohort studies
No studies identified

Non-randomised intervention studies
No studies Identified

Case-control studies
No studies identified

Surveillance reports
No studies identified

Cross-sectional surveys
No studies identified

Case series
No studies identified

Case reports
Not appropriate evidence, not included in evidence synthesis

Olfactory outcomes (1 study)

Meta-analyses
No studies identified

Randomised controlled trials
No studies identified

Cohort studies
No studies identified

Non-randomised intervention studies
No studies identified

Case-control studies
No studies identified

Surveillance reports
No studies identified

Cross-sectional surveys (1)
1. Majchrzak D, Ezzo MC, Kiumarsi M. The effect of tobacco- and electronic cigarettes use on the olfactory function in humans. *Food Qual Prefer* 2020; 86: 103995.

Case series
No studies identified

Case reports
No studies identified

Endocrine outcomes (2 studies)

Meta-analyses
No studies identified

Randomised controlled trials
No studies identified

Cohort studies
No studies identified

Non-randomised intervention studies
No studies identified

Case-control studies
No studies identified

Surveillance reports
No studies identified

Cross-sectional surveys (2)
1. Atuegwu NC, Perez MF, Oncken C, et al. E-cigarette use is associated with a self-reported diagnosis of prediabetes in never cigarette smokers: results from the Behavioral Risk Factor Surveillance System Survey. *Drug Alcohol Depend* 2019; 205: 107692.
2. Orimoloye OA, Uddin SMI, Chen L-C, et al. Electronic cigarettes and insulin resistance in animals and humans: results of a controlled animal study and the National Health and Nutrition Examination Survey (NHANES 2013-2016). *PloS One* 2019; 14: e0226744.

Case series
No studies identified

Case reports
No studies identified

Allergic diseases (4 studies)

Meta-analyses
No studies identified

Randomised controlled trials
No studies identified

Cohort studies

No studies identified

Non-randomised intervention studies
No studies identified

Case-control studies
No studies identified

Surveillance reports
No studies identified

Cross-sectional surveys
No studies identified

Case series (1)

1. Shim TN, Kosztyuova T. Allergic contact dermatitis to electronic cigarette. *Dermatitis* 2018; 29: 94-95.

Case reports (3)

1. Azevedo A, Lobo I, Selores M. Allergic contact dermatitis and electronic cigarettes: is nickel to blame? *Contact Dermatitis* 2019; 81: 135-136.
2. Maridet C, Atge B, Amici J-M, et al. The electronic cigarette: the new source of nickel contact allergy of the 21st century? *Contact Dermatitis* 2015; 73: 49-50.
3. Ormerod E, Stone N. Contact allergy and electronic cigarettes (and eyelash curlers). *Clin Exp Dermatol* 2017; 42: 682-683.

Haematological outcomes (0 studies)

Meta-analyses
No studies identified

Randomised controlled trials
No studies identified

Cohort studies
No studies identified

Non-randomised intervention studies
No studies identified

Case-control studies
No studies identified

Surveillance reports
No studies identified

Cross-sectional surveys
No studies identified

Case series
No studies identified

Case reports
Not appropriate evidence, not included in evidence synthesis

Smoking uptake (28 studies)

Meta-analyses (3)

1. Aladeokin A, Haighton C. Is adolescent e-cigarette use associated with smoking in the United Kingdom?: A systematic review with meta-analysis. *Tob Prev Cessat* 2019; 5: 15.
2. Khouja JN, Suddell SF, Peters SE, et al. Is e-cigarette use in non-smoking young adults associated with later smoking? A systematic review and meta-analysis. *Tob Control* 2020; 30: 8-15.
3. Soneji S, Barrington-Trimis JL, Wills TA, et al. Association between initial use of e-cigarettes and subsequent cigarette smoking among adolescents and young adults: a systematic review and meta-analysis. *JAMA Pediatr* 2017; 171: 788-797.

Randomised controlled trials (2)

1. Conner M, Grogan S, Simms-Ellis R, et al. Evidence that an intervention weakens the relationship between adolescent electronic cigarette use and tobacco smoking: a 24-month prospective study. *Tob Control* 2020; 29: 425-431.
2. Pénzes M, Foley KL, Nădășan V, et al. Bidirectional associations of e-cigarette, conventional cigarette and waterpipe experimentation among adolescents: a cross-lagged model. *Addict Behav* 2018; 80: 59-64.

Cohort studies (23)

1. Aleyan S, Gohari MR, Cole AG, et al. Exploring the bi-directional association between tobacco and e-cigarette use among youth in Canada. *Int J Environ Res Public Health* 2019; 16: 4256.
2. Barrington-Trimis JL, Bello MS, Liu F, et al. Ethnic differences in patterns of cigarette and e-cigarette use over time among adolescents. *J Adolesc Health* 2019; 65: 359-365.
3. Barrington-Trimis JL, Kong G, Leventhal AM, et al. E-cigarette use and subsequent smoking frequency among adolescents. *Pediatrics* 2018; 142: e20180486.
4. Berry KM, Fetterman JL, Benjamin EJ, et al. Association of electronic cigarette use with subsequent initiation of tobacco cigarettes in US youths. *JAMA Netw Open* 2019; 2: e187794.
5. Best C, Haseen F, Currie D, et al. Relationship between trying an electronic cigarette and subsequent cigarette experimentation in Scottish adolescents: a cohort study. *Tob Control* 2018; 27: 373-378.
6. Bold KW, Kong G, Camenga DR, et al. Trajectories of e-cigarette and conventional cigarette use among youth. *Pediatrics* 2018; 141: e20171832.
7. Brose LS, Bowen J, McNeill A, et al. Associations between vaping and relapse to smoking: preliminary findings from a longitudinal survey in the UK. *Harm Reduct J* 2019; 16: 76.
8. Chien YN, Gao W, Sanna M, et al. Electronic cigarette use and smoking initiation in Taiwan: evidence from the first prospective study in Asia. *Int J Environ Res Public Health* 2019; 16: 1145.
9. Dai H, Leventhal AM. Association of electronic cigarette vaping and subsequent smoking relapse among former smokers. *Drug Alcohol Depend* 2019; 199: 10-17.
10. East K, Hitchman SC, Bakolis I, et al. The association between smoking and electronic cigarette use in a cohort of young people. *J Adolesc Health* 2018; 62: 539-547.
11. Kinnunen JM, Ollila H, Minkkinen J, et al. Nicotine matters in predicting subsequent smoking after e-cigarette experimentation: a longitudinal study among Finnish adolescents. *Drug Alcohol Depend* 2019; 201: 182-187.
12. Leventhal AM, Strong DR, Kirkpatrick MG, et al. Association of electronic cigarette use with initiation of combustible tobacco product smoking in early adolescence. *JAMA* 2015; 314: 700-707.
13. Loukas A, Marti CN, Cooper M, et al. Exclusive e-cigarette use predicts cigarette initiation among college students. *Addict Behav* 2018; 76: 343-347.

14. Lozano P, Barrientos-Gutierrez I, Arillo-Santillan E, et al. A longitudinal study of electronic cigarette use and onset of conventional cigarette smoking and marijuana use among Mexican adolescents. *Drug Alcohol Depend* 2017; 180: 427-430.
15. McMillen R, Klein JD, Wilson K, et al. E-cigarette use and future cigarette initiation among never smokers and relapse among former smokers in the PATH study. *Public Health Rep* 2019; 134: 528-536.
16. Miech R, Patrick ME, O'Malley PM, et al. E-cigarette use as a predictor of cigarette smoking: results from a 1-year follow-up of a national sample of 12th grade students. *Tob Control* 2017; 26: e106-e111.
17. Osibogun O, Bursac Z, Maziak W. E-cigarette use and regular cigarette smoking among youth: Population Assessment of Tobacco and Health study (2013-2016). *Am J Prev Med* 2020; 58: 657-665.
18. Primack BA, Shensa A, Sidani JE, et al. Initiation of traditional cigarette smoking after electronic cigarette use among tobacco-naïve US young adults. *Am J Med* 2018; 131: 443.e1-443.e9.
19. Primack BA, Soneji S, Stoolmiller M, et al. Progression to traditional cigarette smoking after electronic cigarette use among US adolescents and young adults. *JAMA Pediatr* 2015; 169: 1018-1023.
20. Spindle TR, Hiler MM, Cooke ME, et al. Electronic cigarette use and uptake of cigarette smoking: a longitudinal examination of US college students. *Addict Behav* 2017; 67: 66-72.
21. Treur JL, Rozema AD, Mathijssen JJP, et al. E-cigarette and waterpipe use in two adolescent cohorts: cross-sectional and longitudinal associations with conventional cigarette smoking. *Eur J Epidemiol* 2017; 33: 323-334.
22. Unger JB, Soto DW, Leventhal A. E-cigarette use and subsequent cigarette and marijuana use among Hispanic young adults. *Drug Alcohol Depend* 2016; 163: 261-264.
23. Wills TA, Knight R, Sargent JD, et al. Longitudinal study of e-cigarette use and onset of cigarette smoking among high school students in Hawaii. *Tob Control* 2017; 26: 34-39.

Non-randomised intervention studies
No studies identified

Case-control studies
No studies identified

Surveillance reports
No studies identified

Cross-sectional surveys
No studies identified

Case series
No studies identified

Case reports
No studies identified

Smoking cessation (11 studies)

Meta-analyses

Not appropriate study design, not included in evidence synthesis[10]

Randomised controlled trials (11)[11]

1. Baldassarri SR, Bernstein SL, Chupp GL, et al. Electronic cigarettes for adults with tobacco dependence enrolled in a tobacco treatment program: a pilot study. *Addict Behav* 2018; 80: 1-5.
2. Bullen C, Howe C, Laugesen M, et al. Electronic cigarettes for smoking cessation: a randomised controlled trial. *Lancet* 2013; 382: 1629-1637.
3. Caponnetto P, Auditore R, Russo C, et al. Impact of an electronic cigarette on smoking reduction and cessation in schizophrenic smokers: a prospective 12-month pilot study. *Int J Environ Res Public Health* 2013; 10: 446-461.
4. Carpenter MJ, Heckman BW, Wahlquist AE, et al. A naturalistic, randomized pilot trial of e-cigarettes: uptake, exposure, and behavioral effects. *Cancer Epidemiol Biomarkers Prev* 2017; 26: 1795-1803.
5. Eisenberg MJ, Hébert-Losier A, Windle SB, et al. Effect of e-cigarettes plus counseling vs counseling alone on smoking cessation: a randomized clinical trial. *JAMA* 2020; 324: 1844-1854.
6. Hajek P, Phillips-Waller A, Przulj D, et al. A randomized trial of e-cigarettes versus nicotine-replacement therapy. *N Engl J Med* 2019; 380: 629-637.
7. Halpern SD, Harhay MO, Saulsgiver K, et al. A pragmatic trial of e-cigarettes, incentives, and drugs for smoking cessation. *N Engl J Med* 2018; 378: 2302-2310.
8. Holliday R, Preshaw PM, Ryan V, et al. A feasibility study with embedded pilot randomised controlled trial and process evaluation of electronic cigarettes for smoking cessation in patients with periodontitis. *Pilot Feasibility Stud* 2019; 5: 74.
9. Lee S-H, Ahn S-H, Cheong Y-S. Effect of electronic cigarettes on smoking reduction and cessation in Korean male smokers: a randomized controlled study. *J Am Board Fam Med* 2019; 32: 567-574.
10. Lucchiari C, Masiero M, Mazzocco K, et al. Benefits of e-cigarettes in smoking reduction and in pulmonary health among chronic smokers undergoing a lung cancer screening program at 6 months. *Addict Behav* 2020; 103: 106222.
11. Walker N, Parag V, Verbiest M, et al. Nicotine patches used in combination with e-cigarettes (with and without nicotine) for smoking cessation: a pragmatic, randomised trial. *Lancet Respir Med* 2020; 8: 54-64.

Cohort studies

Not appropriate study design, not include in review

Non-randomised intervention studies

Not appropriate study design, not include in review

Case-control studies

Not appropriate study design, not include in review

---

[10] In additional to the major international reviews, five meta-analyses of randomised controlled trials were identified with findings on the efficacy of e-cigarettes for smoking cessation. Their results were considered in relation to the current review's findings but not included in evidence synthesis. Further details can be found in the original report.

[11]One additional study was identified after the search and data analysis. Further details can be found in the original report.

Surveillance reports
Not appropriate study design, not include in review

Cross-sectional surveys
Not appropriate study design, not include in review

Case series
Not appropriate study design, not include in review

Case reports
Not appropriate study design, not include in review

5. GRADE table (combined evidence from umbrella and top-up review)

Outcome	Risk of bias[1]	Indirectness	Inconsistency	Imprecision	Publication bias	Certainty of the evidence[2]
Clinical outcomes						
Randomised controlled trials						
Dependence<br>1 study	Serious concerns	Serious concerns	Not applicable	Very serious concerns	Not detected	Very low
Cardiovascular health outcomes	No studies identified					
Cancer	No studies identified					
Respiratory health outcomes	No studies identified					
Oral health	No studies identified					
Developmental and reproductive effects	No studies identified					
Burns and injuries	GRADE was not applied					
Poisoning	GRADE was not applied					
Mental health effects	No studies identified					
Environmental hazards with health implications	No studies identified					
Neurological outcomes	No studies identified					
Sleep outcomes	No studies identified					
Less serious adverse events<br>33 studies	Very serious concerns	Very serious concerns	Very serious concerns	Very serious concerns	Not detected	Very low
Optical health	No studies identified					
Wound healing	No studies identified					
Olfactory outcomes	No studies identified					
Endocrine outcomes	No studies identified					
Allergic diseases	No studies identified					
Haematological outcomes	No studies identified					
Smoking uptake	Not applicable					
Smoking cessation<br>(ENDS vs no intervention/usual care)<br>5 studies	Very serious concerns[3]	No concerns	No concerns	Serious concerns	Undetected	Very low
Smoking cessation<br>(ENDS vs ENNDS)<br>4 studies	Very serious concerns[3]	No concerns	No concerns	Serious concerns	Undetected	Very low
Smoking cessation<br>(ENDS nicotine >0.01mg/mL vs approved NRT)<br>2 studies	Serious concerns[3]	No concerns	No concerns	Serious concerns	Undetected	Low
Smoking cessation<br>(ENNDS vs usual care)	Serious concerns[3]	No concerns	No concerns	Very serious concerns	Undetected	Very low

Outcome	Risk of bias[1]	Indirectness	Inconsistency	Imprecision	Publication bias	Certainty of the evidence[2]
2 studies						
Smoking cessation (ENNDS vs other NRT) 1 study	Serious concerns[3]	No concerns	Not applicable, only one study	Very serious concerns	Undetected	Very low
**Non-randomised studies[4]**						
Dependence (1 cohort, 8 non-randomised intervention, 21 cross-sectional)	Very serious concerns	Very serious concerns	No concerns	Serious concerns	Not detected	Very low
Cardiovascular health outcomes	No studies identified					
Cancer 1 study (1 cohort)	Very serious concerns	Serious concerns	Not applicable	Serious concerns	Not detected	Very low
Respiratory health outcomes 4 studies (4 cohort)	Serious concerns	Very serious concerns	No concerns	Serious concerns	Not detected	Very low
Oral health 3 studies (2 cohort, 1 non-randomised intervention)	No concerns	Serious concerns	Serious concerns	Serious concerns	Not detected	Very low
Developmental and reproductive effects 3 studies (2 cohort, 1 cross-sectional)	No concerns	Serious concerns	Serious concerns	Very serious concerns	Not detected	Very low
Burns and injuries	GRADE was not applied					
Poisoning	GRADE was not applied					
Mental health effects	No studies identified					
Environmental hazards with health implications 22 studies (17 controlled, 5 natural experiment)	Serious concerns	Serious concerns	Very serious concerns	Very serious concerns	Not detected	Very low
Neurological outcomes	GRADE was not applied					
Sleep outcomes	No studies identified					
Less serious adverse events 21 studies (4 non-randomised intervention, 17 cohort)	Very serious concerns	Serious concerns	Serious concerns	Very serious concerns	Not detected	Very low
Optical health	No studies identified					
Wound healing	No studies identified					
Olfactory outcomes	No studies identified					
Endocrine outcomes	No studies identified					
Allergic diseases	No studies identified					
Haematological outcomes	No studies identified					
Smoking uptake	GRADE was not applied					
Smoking cessation	Not applicable					
**Subclinical/intermediate outcomes**						
**Randomised controlled trials**						

Outcome	Risk of bias[1]	Indirectness	Inconsistency	Imprecision	Publication bias	Certainty of the evidence[2]
Abuse liability<br>13 studies	Very serious concerns	Very serious concerns	Very serious concerns	Very serious concerns	Not detected	Very low
Cardiovascular health outcomes	No studies identified					
Cancer	No studies identified					
Respiratory health outcomes 9 studies	Serious concerns	Very serious concerns	Very serious concerns	Very serious concerns	Not detected	Very low
Oral health	No studies identified					
Developmental and reproductive effects	No studies identified					
Burns and injuries	GRADE was not applied					
Poisoning	GRADE was not applied					
Mental health effects	No studies identified					
Environmental hazards with health implications	No studies identified					
Neurological outcomes	Not applicable					
Sleep outcomes	Not applicable					
Less serious adverse events	Not applicable					
Optical health	No studies identified					
Wound healing	No studies identified					
Olfactory outcomes	No studies identified					
Endocrine outcomes	No studies identified					
Allergic diseases	Not applicable					
Haematological outcomes	Not applicable					
Smoking uptake	Not applicable					
Smoking cessation	Not applicable					
Non-randomised studies[4]						
Abuse liability<br>16 studies (15 non-randomised intervention, 1 cross-sectional)	Serious concerns	Serious concerns	Very serious concerns	Very serious concerns	Not detected	Very low
Cardiovascular health outcomes	No studies identified					
Cancer	No studies identified					
Respiratory health outcomes 9 studies (4 cohort, 8 non-randomised intervention)	Very serious concerns	Very serious concerns	Very serious concerns	Very serious concerns	Not detected	Very low
Oral health<br>2 studies (1 cohort, 1 non-randomised intervention)	No concerns	Very serious concerns	Very serious concerns	Very serious concerns	Not detected	Very low
Developmental and reproductive effects	No studies identified					
Burns and injuries	GRADE was not applied					
Poisoning	GRADE was not applied					
Mental health effects<br>3 studies (3 cohort)	Very serious concerns	Very serious concerns	Very serious concerns	Serious concerns	Not detected	Very low

Outcome	Risk of bias[1]	Indirectness	Inconsistency	Imprecision	Publication bias	Certainty of the evidence[2]
Environmental hazards with health implications	No studies identified					
Neurological outcomes	Not applicable					
Sleep outcomes	Not applicable					
Less serious adverse events	Not applicable					
Optical health<br>1 study (1 non-randomised intervention)	Serious concerns	Very serious concerns	Not applicable	Very serious concerns	Not detected	Very low
Wound healing	No studies identified					
Olfactory outcomes<br>1 study (1 non-randomised intervention)	Serious concerns	Serious concerns	Not applicable	Very serious concerns	Not detected	Very low
Endocrine outcomes<br>2 studies (2 cross-sectional)	Serious concerns	Serious concerns	Very serious concerns	Serious concerns	Not detected	Very low
Allergic diseases	Not applicable					
Haematological outcomes	Not applicable					
Smoking uptake	Not applicable					
Smoking cessation	Not applicable					

ENDS = electronic nicotine delivery system; ENNDS = electronic non-nicotine delivery system; GRADE = Grading of Recommendations Assessment, Development and Evaluation; NRT = nicotine replacement therapy.

[1]Risk of bias assessments (using the Joanna Briggs Institute's (JBI) critical appraisal checklists) were only available for studies included in the top-up review. Rating should be interpreted with caution.

[2]Certainty of evidence should be interpreted with caution as risk of bias was available only for studies in the top-up review.

[3]Risk of bias assessments were conducted using the Cochrane risk-of-bias tool for randomised controlled trials.[814]

[4]No studies were eligible for upgrading-criteria not presented in table.

References

1. Committee on the Review of the Health Effects of Electronic Nicotine Delivery Systems (National Academies of Sciences, Engineering, and Medicine). Public health consequences of e-cigarettes. Washington (DC): National Academies Press, 2018. https://www.ncbi.nlm.nih.gov/books/NBK507171/pdf/Bookshelf_NBK507171.pdf (viewed Nov 2019).
2. McNeill A, Brose LS, Calder R, et al. Evidence review of e-cigarettes and heated tobacco products 2018. A report commissioned by Public Health England. Feb 2018. https://assets.publishing.service.gov.uk/government/uploads/system/uploads/attachment_data/file/684963/Evidence_review_of_e-cigarettes_and_heated_tobacco_products_2018.pdf (viewed Nov 2019).
3. Byrne S, Brindal E, Williams G, et al. E-cigarettes, smoking and health: a literature review update. 22 June 2018. https://www.csiro.au/en/research/health-medical/diseases/Health-impacts-of-electronic-cigarettes (viewed Nov 2019).
4. The Joanna Briggs Institute. Critical appraisal tools. https://jbi.global/critical-appraisal-tools (viewed July 2020).
5. Schünemann H, Brozek J, Guyatt G, Oxman A (editors). GRADE handbook. Updated Oct 2013. https://gdt.gradepro.org/app/handbook/handbook.html (viewed July 2021).
